# Stabilization of Glucosyl Dioxolenium Ions by “Dual Participation” of the 2,2-Dimethyl-2-(*ortho*-nitrophenyl)acetyl (DMNPA) Protection Group for 1,2-*cis*-Glucosylation

**DOI:** 10.1021/acs.joc.2c00808

**Published:** 2022-06-24

**Authors:** Wouter A. Remmerswaal, Kas J. Houthuijs, Roel van de Ven, Hidde Elferink, Thomas Hansen, Giel Berden, Herman S. Overkleeft, Gijsbert A. van der Marel, Floris P. J. T. Rutjes, Dmitri V. Filippov, Thomas J. Boltje, Jonathan Martens, Jos Oomens, Jeroen D. C. Codée

**Affiliations:** † Leiden Institute of Chemistry, Leiden University, Einsteinweg 55, 2333 CC Leiden, The Netherlands; ‡ Institute for Molecules and Materials, FELIX Laboratory, Radboud University, Toernooiveld 7, 6525 ED Nijmegen, The Netherlands; § Institute for Molecules and Materials, Radboud University, Heyendaalseweg 135, 6525 AJ Nijmegen, The Netherlands; ∥ Departament de Química Inorgànica i Orgànica & IQTUB, Universitat de Barcelona, 08028 Barcelona, Spain

## Abstract

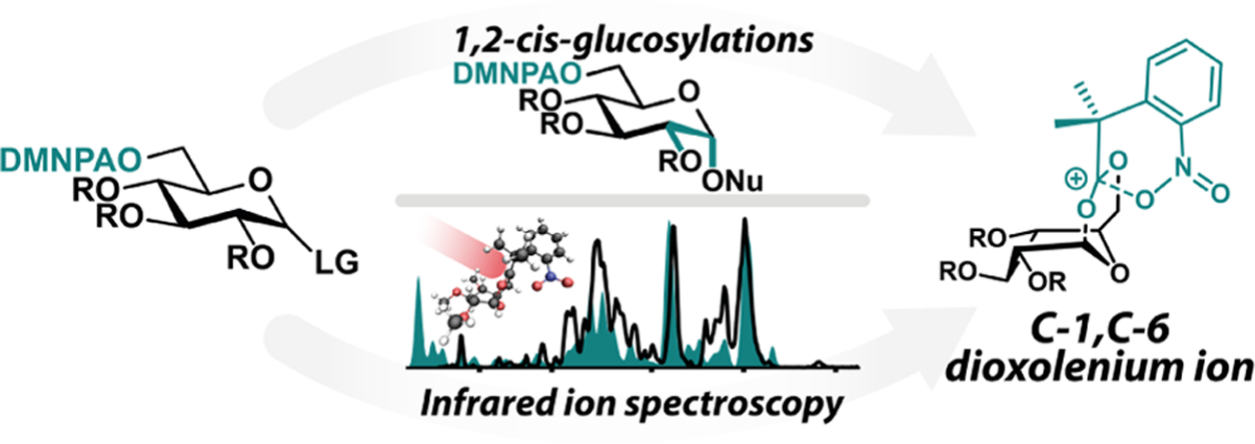

The stereoselective introduction of glycosidic bonds is of paramount importance to oligosaccharide synthesis. Among the various chemical strategies to steer stereoselectivity, participation by either neighboring or distal acyl groups is used particularly often. Recently, the use of the 2,2-dimethyl-2-(*ortho*-nitrophenyl)acetyl (DMNPA) protection group was shown to offer enhanced stereoselective steering compared to other acyl groups. Here, we investigate the origin of the stereoselectivity induced by the DMNPA group through systematic glycosylation reactions and infrared ion spectroscopy (IRIS) combined with techniques such as isotopic labeling of the anomeric center and isomer population analysis. Our study indicates that the origin of the DMNPA stereoselectivity does not lie in the direct participation of the nitro moiety but in the formation of a dioxolenium ion that is strongly stabilized by the nitro group.

## Introduction

**Figure 1:**
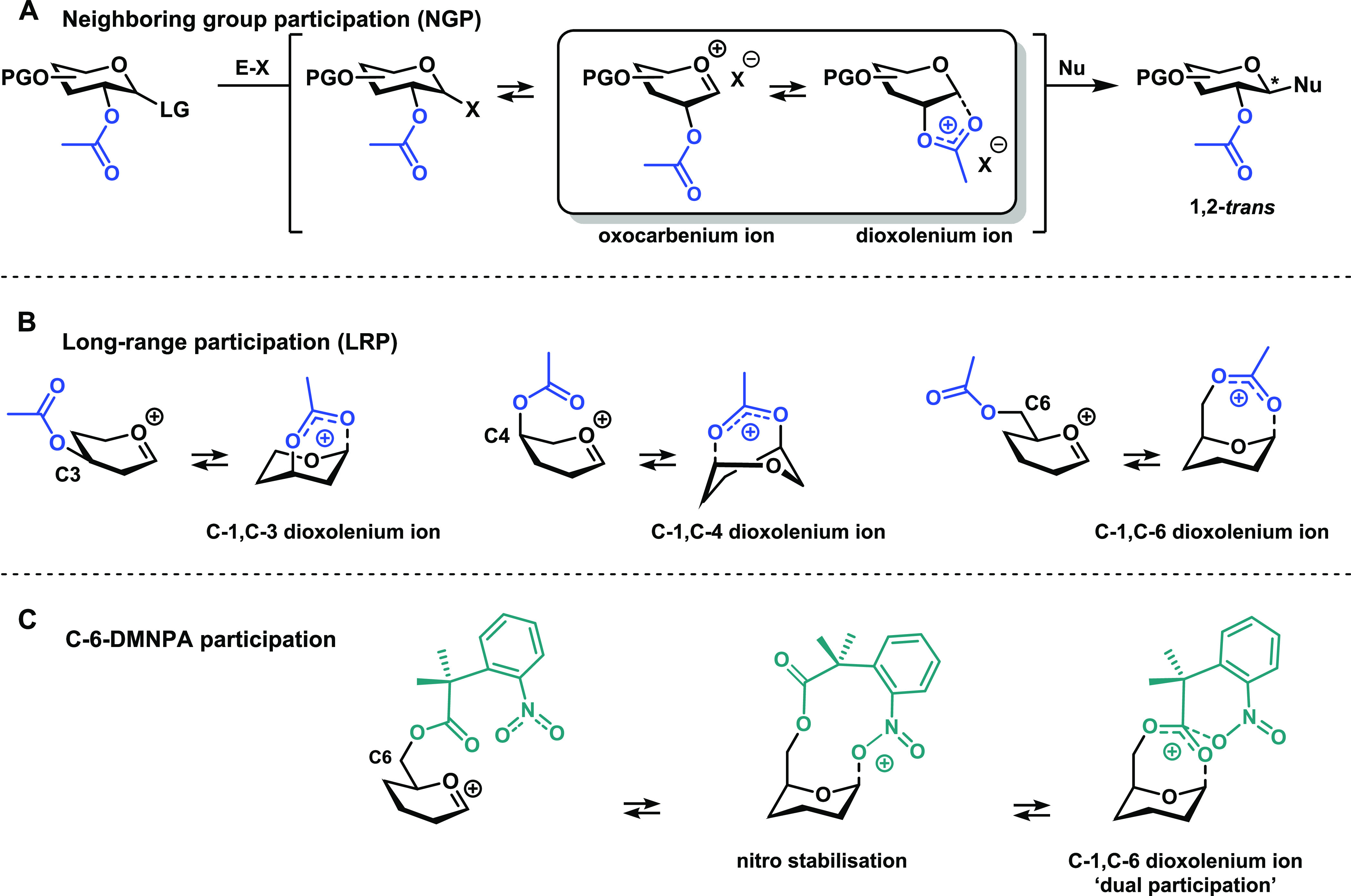
NGP (A) and LRP (B) in glycosylation reactions allow to control the stereoselectivity of glycosylation reactions. Schematic representation of the possible reactive intermediates in NGP and LRP. PG = protection group, E–X = promoter system, Nu = nucleophile. (C) LRP by the DMNPA group, mounted at C-6.

The stereoselective introduction of glycosidic bonds remains a major challenge in the chemical synthesis of oligosaccharides. The protecting group pattern on both reaction partners in the glycosylation reaction has a dramatic effect on the stereochemical outcome. Thus, careful selection of the protecting groups can enable one to steer the glycosylation reaction to the desired stereoisomer.^1−4^ The use of C-2 acyl-protecting groups results in neighboring group participation (NGP), by the formation of a bicyclic C-1,C-2-dioxolenium ion intermediate (Figure 1A), which reliably forms 1,2-*trans* glycosidic bonds.^5,6^ This strategy is one of the cornerstones of oligosaccharide synthesis.^7^ By definition, however, this only allows for the formation of 1,2-*trans* glycosides. In contrast, long-range participation (LRP) of acyl groups from distal positions (*i.e*., C-3, C-4, and C-6) can potentially enable the introduction of 1,2-*cis* linkages (Figure 1B). The origin and the strength of this stereodirecting effect remain poorly understood and are heavily debated.^8−11^ Evidence for the occurrence of LRP comes from the stereoselectivity of glycosylation reactions featuring remote acyl groups on the donor glycosides and the isolation of cyclic orthoesters.^12−25^ Dioxolenium ions formed by attack of the remote esters on the anomeric center of activated glycosyl donors have recently been detected in both the gas phase, by infrared ion spectroscopy (IRIS),^26−28^ and in solution by NMR experiments.^29^

Using a combination of IRIS, density functional theory (DFT) calculations,^30^ and model glycosylation experiments,^26^ we recently mapped how the strength of LRP depends on the position of the participating ester groups on the glycosyl donor ring as well as the relative stereochemistry of the donor glycoside. The strongest LRP was observed for C-3-*O*-acyl mannosyl donors. These provide excellent α-selectivity with a range of nucleophiles, and DFT calculations have indicated the bridged intermediate 1,3-dioxolenium ion to be significantly more stable than the corresponding oxocarbenium ion. Subsequently, we provided evidence for the existence of this bridged ion in solution using chemical exchange saturation transfer NMR experiments in which we could detect a cross-coupling peak between the anomeric carbon and a ^13^C labeled C-3-acyl group.^31^ Less prominent LRP effects were observed for other systems such as C-4-acyl galactosides, for which Crich and co-workers have argued that attack on the activated anomeric center of these donors is hampered by the orientation of the C-4-ester. They suggest that this group preferentially takes up a conformation in which the C=O nearly eclipses the C-4,H-4 bond, and that rotation along the C-4–O-4 axis is too unfavorable to allow for the formation of a dioxolenium ion.^10^ This is supported by recent findings in which this rotational barrier is lowered by placing a methyl at the C-4, thus creating a quaternary carbon atom. The methylated C-4-*O*-benzoyl group formed a C-4,C-1 dioxolenium ion and was observed by NMR spectroscopy.^32^

Although LRP has also been invoked to account for increased α-selectivity in C-6-acyl glycosyl donors, bridged 1,6-dioxolenium ions have not been observed experimentally. In contrast to the C-1,C-3 and C-1,C-4 dioxolenium ions observed by IRIS for C-3-acyl mannosides and C-4-acyl galactosides, ions generated from C-6-acyl-functionalized pyranosides showed ring-opened structures in which the C-6-acyl group attacks the C-5 to expel the O-5, forming a C-5,C-6-dioxolenium ion with concomitant generation of the C-1-aldehyde. DFT calculations did not reveal any stabilization of the parent oxocarbenium ions by C-1,C-6-dioxolenium ion formation.

To enhance LRP effects to control the stereoselectivity of glycosylation reactions, the 2,2-dimethyl-2-(*ortho*-nitrophenyl)acetyl (DMNPA) protection group was recently introduced.^33^ This protection group has been shown to steer the stereoselectivity from various distal positions on differently configured glycosyl donors, consistent with an LRP mechanism.^34^ The current hypothesis for the origin of the enhanced LRP effect of the DMNPA is the unique chemical structure that may enable a “dual-participation” mechanism. The intermediate dioxolenium ion can be stabilized through the donation of electron density from the aryl nitro group, which is brought into close proximity of the central carbon atom of the dioxolenium ion by the geminal dimethyl groups through the Thorpe–Ingold effect^35^ (Figure 1C). This hypothesis is supported by crystal structures that indicate the interaction of the nitro group with the DMNPA carbonyl in the parent donor molecules.

However, little direct experimental evidence is available for the proposed dual-participation mechanism and initial computational studies have shown that stabilization of the intermediate oxocarbenium ion may also take place by direct interaction of the nitro group with the anomeric center. IRIS experiments provide an excellent opportunity to probe the structure of intrinsically labile cations and discriminate dioxolenium and oxocarbenium ions. It has been proposed that the DMNPA group may be used to assist in the formation of α-glucosyl linkages, present in many biologically and structurally relevant polysaccharides. We therefore set out to unravel the possible mechanisms of LRP in DMNPA-functionalized glucosyl donors, and we here combine a set of model glycosylation reactions, employing a set of partially fluorinated alcohol acceptors of gradually increasing nucleophilicity, with the characterization of reactive intermediates by IRIS techniques. Isotope labeling has been used to gain additional information on the different isomers of the cations, generated upon ionization. An isomer population analysis was performed to probe the structures that were simultaneously present in the gas phase cation mixture. Altogether, our experiments show that mounting the DMNPA group at the C-3 or C-4 glucosyl alcohols does not affect the stereoselectivity of the glycosylation reactions, but the C-6-DMNPA ester may provide LRP to favor the formation of the α-glucosyl products. The C-6-DMNPA group may stabilize the glucosyl oxocarbenium ion through a dual participation mechanism in which the distal ester attacks the anomeric center and the DMNPA nitro group stabilizes the dioxolenium ion. Stabilization of the ionic intermediates can shift the glycosylation reaction mechanism from an S_N_2-type substitution on the α-anomeric glucosyl triflate, which leads to the β-linked product, to a mechanism involving the stabilized ionic species that provides the challenging α-glucosides.

## Results and Discussion

### Model Glycosylation Reactions

To systematically investigate the stereodirecting effect of the DMNPA group, a matrix of model glycosylation reactions was performed in which the stereoselectivity of glycosylations of different glucosyl donors is compared. To this end, we generated the C-3, C-4, and C-6 DMNPA-protected glucosyl donors (**3**, **5**, and **7**, respectively). The synthesis is depicted in Supporting Information Scheme S1 alongside their benzoyl counterparts (**2**, **4**, and **6**, respectively) and the benchmark glucosyl donor **1**, bearing solely benzyl ether protecting groups (Table 1). The acceptors used for the model glycosylation reactions consist of a set of model acceptors of systematically increasing nucleophilicity. Glycosylation reactions with these partially fluorinated ethanol derivatives (*i.e*., hexafluoro-2-propanol, HFIP; 2,2,2-trifluoroethanol, TFE; 2,2-difluoroethanol, DFE; 2-fluoroethanol, MFE; ethanol, EtOH) can be used to probe the effect of acceptor nucleophilicity on the stereoselectivity of the glycosylation reaction.^8^ The glycosylation reactions were performed under preactivation conditions using a slight excess of diphenyl sulfoxide (Ph_2_SO) and triflic anhydride (Tf_2_O) as an activator system (Table 1). As we previously reported, glycosylation reactions with the per-*O*-benzylated glucose donor exhibit a gradual shift from α- to β-stereoselectivity as the nucleophilicity of the acceptor increases.^36,37^ This can be explained by a shift in the reaction mechanism through which the glycosidic linkages are formed. The weaker nucleophiles require a more electrophilic glycosylating agent, such as a glycosyl oxocarbenium ion-like species, a related contact ion pair, or an equatorial anomeric triflate, while reactive nucleophiles can displace the more stable covalent anomeric axial triflate.

**Table 1:**
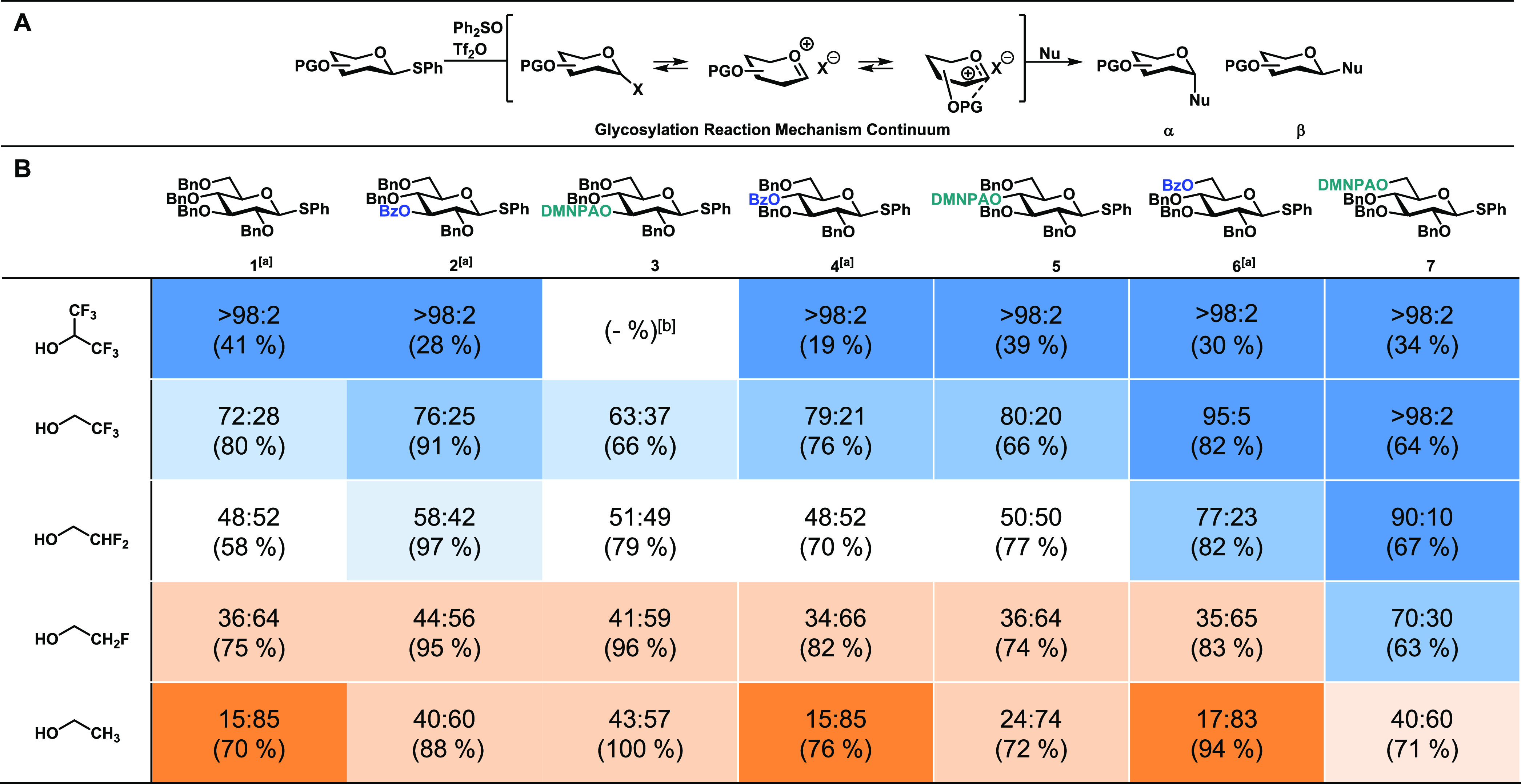
Model Glycosylation Reactions^a^^b^^c^

a Experimental data of the per-benzyl and benzoate donor glycosylation reactions from Hansen et al.^26^

b Product formation was not observed from crude NMR and could not be isolated.

c The stereoselectivity of the reaction is expressed as α:β and based on ^1^H-NMR of purified α/β-product mixtures. Blue-colored cells represent α-selectivity, while orange-colored cells represent β-selectivity. The percentage given in parentheses represents the yield after purification by column chromatography; preactivation-based glycosylation conditions: donor **1**–**7** (1 equiv), Tf_2_O (1.3 equiv), Ph_2_SO (1.3 equiv), TTBP (2.5 equiv), dichloromethane (DCM) (0.05 M), −80 to −60 °C, then add nucleophile (2 equiv) at −80 °C.

Table 1 summarizes the results of the model glycosylation reactions. All of these reactions were performed under the same preacitvation conditions (Tf_2_O (1.3 equiv), Ph_2_SO (1.3 equiv), TTBP (2.5 equiv), DCM (0.05 M), −80 to −60 °C, then add nucleophile (2 equiv) at −80 °C), with excess acceptor and high dilution to minimize the concentration change during the reaction.^38^ Previously, we reported that placing a benzoate on the C-3, C-4, or C-6 of a glucosyl donor had virtually no effect on the stereoselectivity of the glycosylation reactions compared to the per-benzylated glucosyl donor. Installation of the DMNPA group on either C-3 or C-4 did not affect the stereoselectivity trends either. However, a significant shift in stereoselectivity, toward the formation of more α-linked products, is observed when this group is mounted on C-6. Notably, the reactions of 2,2-difluoroethanol and 2,2,2-trifluoroethanol proceed with excellent selectivity and only the α-linked products were obtained. As the reactivity of carbohydrate alcohol acceptors roughly corresponds to the reactivity of these model alcohols, this indicates that C-6-DMNPA can be used to construct the challenging α-glucosyl glycosidic linkages in oligosaccharides. The difference in stereoselectivity between the C-6-OBn/Bz donors **1**/**6** and C-6-DMNPA donor **7** is indicative of a shift in the mechanisms of the glycosylation reaction. Specifically, the enhanced selectivity toward a product, with the glycosidic linkage trans with respect to the acyl group, may be indicative of a mechanism involving LRP. The absence of enhanced α-selectivity for the *O*-6-benzoate donor demonstrates the LRP-enhancing effect of the DMNPA group. Overall, the stereoselectivity trends in Table 1 indicate that the DMNPA group may enable LRP, but that participation critically depends on the position of the carbohydrate ring.

To investigate whether dual participation can play a role in the stabilization of the intermediate glycosyl cations, the structure of the DMNPA-containing glycosyl cations was studied by IRIS. Based on the observed selectivity in the model glycosylation experiments, we prepared the methylated (commonly used in IRIS and computational studies to minimize spectral congestion^26,39^ and computational cost,^26,30^ respectively) anomeric sulfoxide derivative of the α-selective glucosyl donor **7**, *i.e*., glucosyl sulfoxide **8** (Supporting Information Scheme S2). To obtain the glycosyl cations of this donor, the proton adduct was generated by electrospray ionization (ESI+) and isolated in a Bruker AmaZon Speed ion trap.^40^ Subsequently, the sulfoxide leaving group was expelled by collision-induced dissociation (CID) to generate the glycosyl cation. An IR spectrum of the isolated glycosyl cation was measured using the free-electron laser FELIX^41^ in the 600–1900 cm^–1^ range by monitoring the wavelength-dependent IR multiple photon-induced dissociation (IRMPD) yield.^42^ Structural assignment was achieved by comparison of the IR spectra to the DFT-calculated spectra (B3LYP/6–31++G(d,p)). This combination of functional and basis set has been shown to perform well for predicting vibrational spectra of the type of systems considered here, and for consistency, we have continued with this approach.^39,43,44^ Alternative basis sets and the inclusion of a dispersion correction^45^ have been evaluated to have a minimal effect on the computed vibrational spectra (see Supporting Information Figure S2). Higher-level energies are obtained by combining the B3LYP calculated Gibbs free energy with the electronic energy of an MP2/6-311++G(2d,2p) single point calculation. Candidate geometries of possible conformations were generated using an earlier reported workflow.^46^

**Figure 2:**
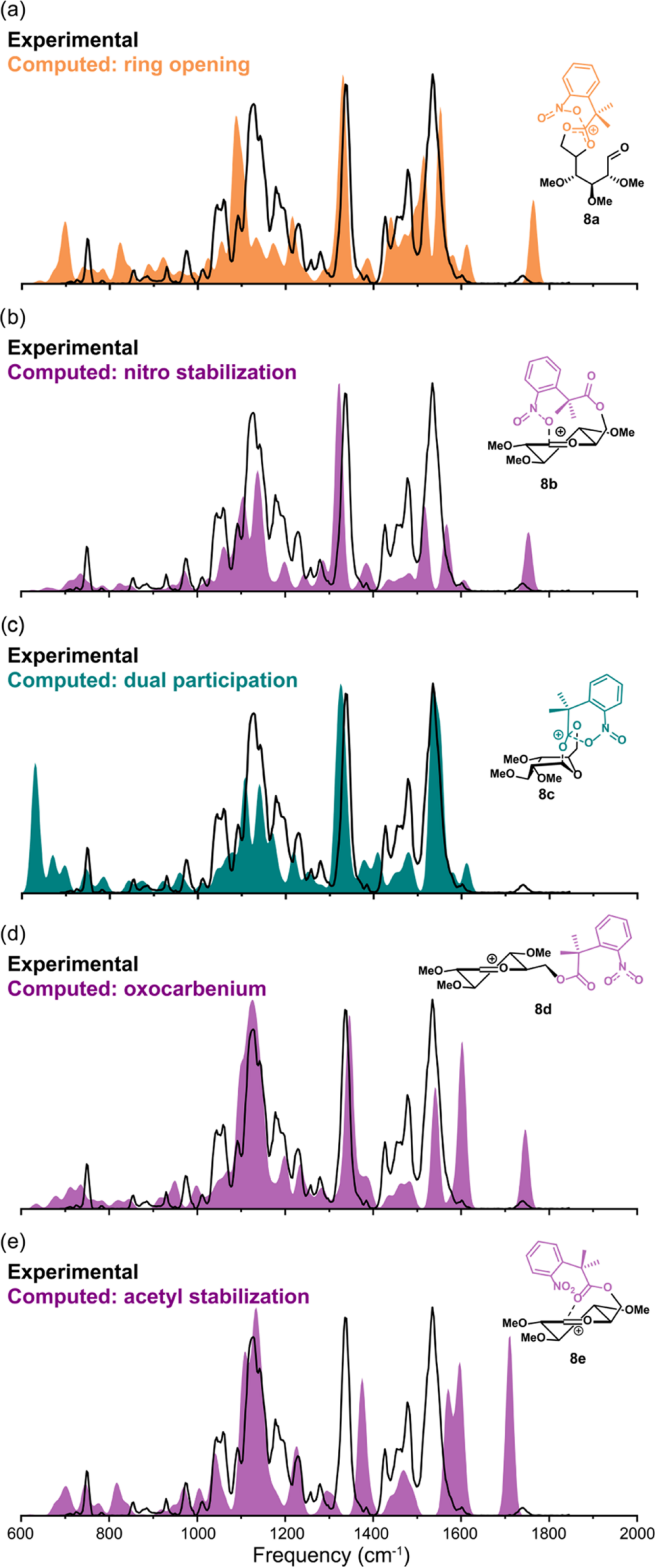
Comparison of the experimental IR spectrum of the glycosyl cation of **8** at *m*/*z* 396 (black) to the calculated spectra (filled, colored) of the ring-opened C-5,C-6-dioxolenium ion with nitro stabilization **8a** (a), the nitro-stabilized oxocarbenium ion **8b** (b), the C-1,C-6-dioxolenium ion with nitro stabilization **8c** (c), the oxocarbenium ion **8d** (d), and the acetyl-stabilized oxocarbenium ion **8e** (e).

The experimental IR spectrum of the glucosyl cation generated from **8** is presented in black in Figure 2, along with the computed spectra of different isomeric cation structures: the ring-opened C-5,C-6-dioxolenium ion with nitro stabilization (**8a**), the nitro-stabilized oxocarbenium ion (**8b**), the C-1,C-6-dioxolenium ion with nitro stabilization (**8c**), the oxocarbenium ion (**8d**), and the acetyl-stabilized oxocarbenium ions (**8e**). Previous work has shown that the most diagnostic peaks for these spectral comparisons are the carbonyl stretch around 1750 cm^–1^, the oxocarbenium C=O^+^ stretch around 1600 cm^–1^, and the dioxolenium ion O–C^+^–O stretch around 1550 cm^–1^.^26^ Unfortunately, both the O–C^+^–O and C=O^+^ stretches are obscured by the nitro O–N–O asymmetric stretching in the same region, but from the generated spectra, it can be concluded that the spectrum corresponding to the dual participation structure **8c** (Figure 2c) matches best, indicating that this is a favorable dioxolenium ion. This dual participation structure (**8c**) is however unable to account for the characteristic band at 1737 cm^–1^. This IR frequency points toward the presence of a carbonyl, which is present in all other isomers considered. The computed C=O stretches of the oxocarbenium with (**8b**) and without (**8e**) nitro stabilization match well with the experiment, suggesting their presence in the ion population. However, the ring-opened structure **8a** and the acetyl-stabilized oxocarbenium **8e** cannot be definitively excluded, as the former showed a blueshift for the aldehyde stretch. This general mismatch of the experimental C=O stretch with B3LYP-computed frequencies is observed for similar ring-opened structures.^26,47^

**Figure 3:**
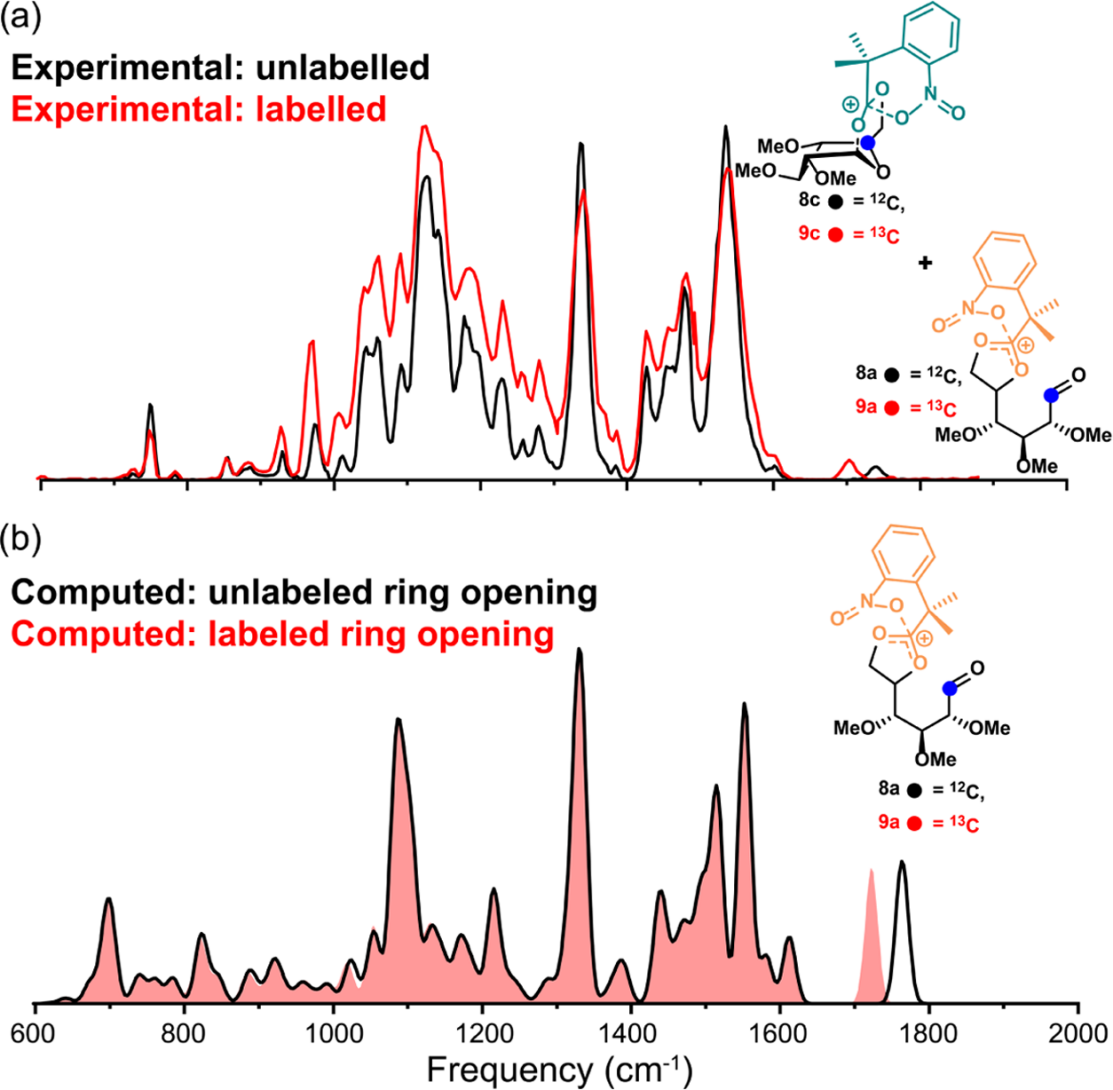
Comparison of the experimental (a) and computational (b) IR spectra of the glycosyl cations of **8** and its ^13^C-1 labeled analogue **9**.

To definitively assign the experimental C=O stretch to one of the isomers **8a, 8b, 8d**, or **8e**, we made use of isotopic labeling, where the labeled functional group can be correlated with a specific band in the spectrum by a frequency shift induced by the change in mass. In the case of compound **8**, we synthesized the labeled derivative **9** with a ^13^C atom at the C-1 position (Supporting Information Scheme S2). Figure 3a shows the experimental IR spectra of both the unlabeled (**8**, black) and the labeled (**9**, red) compounds. Although variations in intensity are observed, the positions of the IR features are generally conserved after labeling, except for the position of the carbonyl stretch. The redshift of the carbonyl stretch upon labeling indicates that this carbonyl stretch involves the ^13^C atom and thus originates from C-1. This stretch must therefore represent the aldehyde found in the ring-opened structure. This is further supported by Figure 3b, which shows an overlap of the calculated spectra of the ring-opened structure without labeling (black line) and with labeling (red filled spectrum), showing the same redshift of the carbonyl stretch. None of the other geometries showed a similar frequency shift of bands in this region (Supporting Information Figure S1). The labeling thus confirms the presence of the ring-opened conformation and excludes the other oxocarbenium structures, with and without stabilization of the nitro or acetyl group.

From the observed intensity of the aldehyde peak, it is difficult to assess how much the ring-opened and dual-participation dioxolenium ion structures contribute to the ion mixture. To quantify their relative contributions to the total ion population, an isomer population analysis (IPA) was performed.^48−51^ In this experiment, the IR wavelength is kept fixed while the number of laser pulses irradiating the ions is increased.^51^ By monitoring the normalized precursor intensity (*I*_precursor_/*I*_total_) as a function of the number of pulses, a precursor ion depletion curve is obtained. Only the ions that have a resonant absorption at the selected wavelength absorb IR light and undergo fragmentation so that convergence to a nonzero plateau is observed when the ion population consists of multiple structures with unique vibrational bands. The level of the plateau indicates the relative contribution of the absorbing ion to the total population.

**Figure 4:**
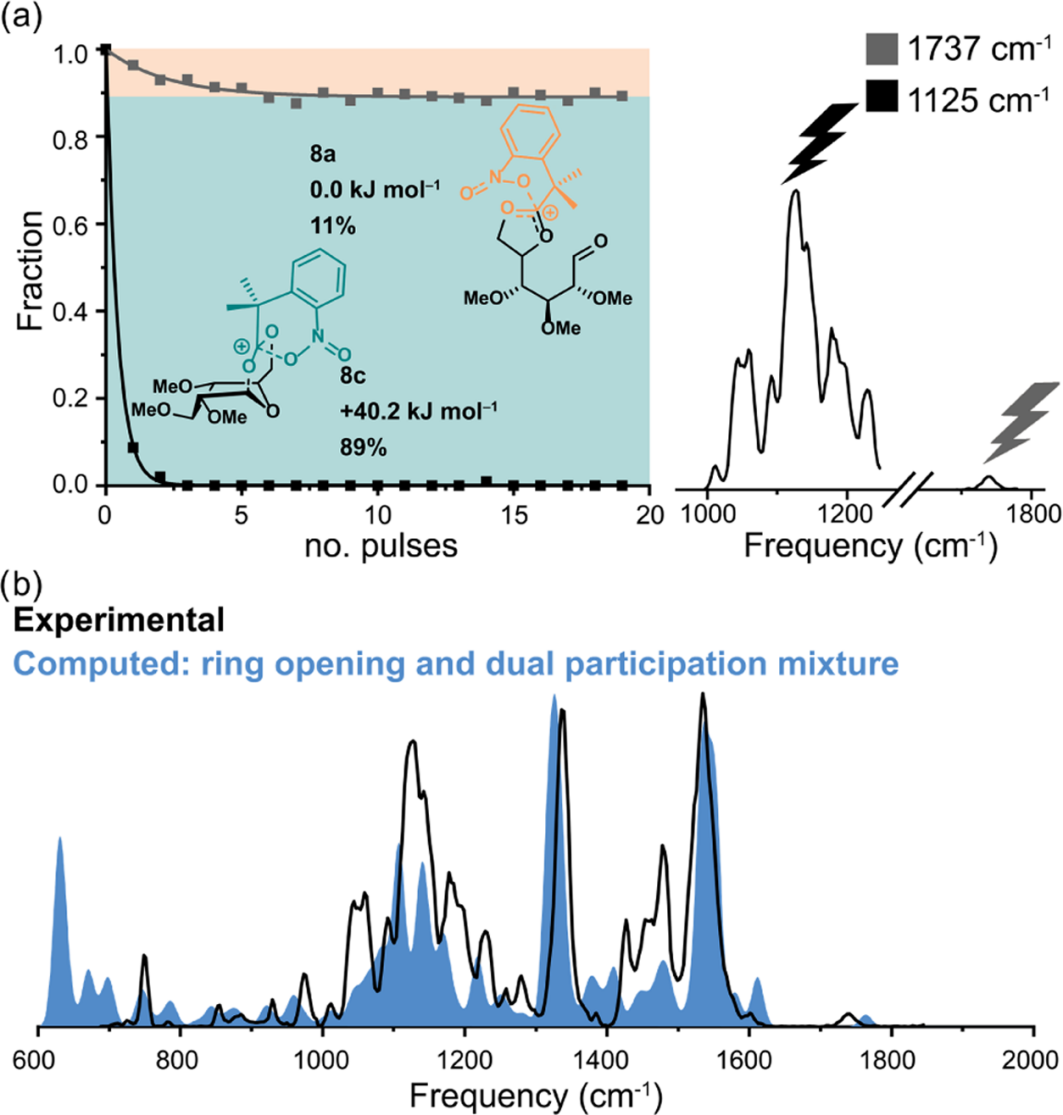
Isomer population analysis of the glycosyl cations of **8** (a) and comparison of the experimental IR spectrum of glycosyl cation of **8** to the 11:89 mix of the computed spectra of structures **8a** and **8c**, respectively (b).

Figure 4a displays the results of the IPA for the unlabeled glucosyl cation **8**. The control measurement with the laser at 1125 cm^–1^, exciting various C–H vibrations present in both the ring-opened and the dual-participation isomers, decayed to 0%, suggesting the absence of background ions (and that all trapped ions have spatial overlap with the laser focus). Tuning the laser to the aldehyde stretch at 1733 cm^–1^ of the ring-opened structure, the normalized precursor intensity converges to 89%, indicating that the 11% that is removed corresponds to the ring-opened isomer and the remaining 89% to the dual-participation isomer. A comparison of the experimental spectrum to an 11:89 mix of the computed spectra of ions **8a** and **8c** shows excellent agreement (Figure 4b), thereby further corroborating the presence of the dual-participating dioxolenium ions. The relative abundance of both structures does not parallel their stability as derived from DFT calculations, which predict the ring-opened structure to be more stable by 40.2 kJ mol^–1^. The selective depletion of the ring-opened ion **8a** from the ion mixture indicates that the isomers are not in dynamic equilibrium, so it can be argued that the ring-opened structure requires more energy to form. Thus, the formation of the dual-participating structure **8c** is kinetically controlled.^52^ To investigate the kinetic trapping of the dual-participating structure, a second IPA was performed on in-source generated glycosyl cations that were directly isolated (i.e., not generated using CID). Under the high-pressure conditions in the source region, fragmentation reactions shift toward the thermodynamic product.^53^ Indeed, a shift toward the ring-opened structure is observed (11 to 48%, Supporting Information Figure S3), thus indicating that the dual-participating structure is kinetically trapped.

The ring-opened structures have never been observed as side products in glycosylation reactions, and therefore, the relevance of these gas-phase structures for condensed-phase chemistry is only indirect. The formation of these species in the gas phase at the expense of other isomers, such as oxocarbenium or dioxolenium ions, does provide an indication of the stability of these latter ions. Stable oxocarbenium or dioxolenium ions are less likely to undergo ring opening, and therefore, the presence of the ring-opened ions can provide an indirect measure of the relative stability of the oxocarbenium/dioxolenium ions. Here, the observation of dual participation is of particular interest since it is to our knowledge the first time that a dioxolenium ion is formed by an *O*-acyl group participating from the 6-position. Earlier, such structures either underwent ring opening,^26,27^ or showed participation from the 2-^39^ or 4-^27^ position when other participating groups were present. Thus, it appears that the ability of the DMNPA group to form a dual-participating structure is a necessity for sufficient stabilization to prevent ring opening from occurring.

Overall, the IRIS spectra have indicated the dual-participation structure to be the most important glucosyl cation formed upon CID of the C-6-DMNPA glucosyl donors. For the corresponding C-6-benzoate, we have only been able to observe the ring-opened C-5,C-6-dioxolenium ion, showing that the C-6-DMNPA dual participation leads to a more stable structure. This translates well to the observed shift in stereoselectivity presented in Table 1. The dual participation of the C-6-DMNPA group stabilizes the intermediate ions during the glycosylation reaction, thereby shifting the glycosylation reaction mechanism from the side in which an anomeric α-triflate is displaced in an S_N_2 fashion to provide the β-products to the side of the ionic intermediates, leading to the formation of the α-products. The DMNPA is unable to engage in this dual participation from other positions on the glucose ring, as inferred from the very similar stereoselectivity trends of the C-3/C-4-DMNPA and Bz donors. The lack of more effective LRP by the DMNPA compared to the Bz from these latter positions may be accounted for by the steric requirements of this group while forming the bridged dioxolenium ions. The geminal dimethyl group and the quaternary carbon formed by the stabilization of the dioxolenium ion by the nitro functionality may be most easily accommodated when the DMNPA group is mounted on the primary alcohol.

## Conclusions

In conclusion, we have probed the effect of the DMNPA group on the stereoselectivity of glycosylation reactions. From the series of glycosylation reactions, it became apparent that this group can be mounted on the C-6 to direct the glycosylations to provide the challenging α-products. IRIS has provided evidence for the existence of a C-1,C-6-dioxolenium ion in the gas phase. Of note, this is the first glycosyl C-1,C-6-dioxolenium ion that we have observed. Previously, C-6-acetyl and benzoyl glycosyl oxocarbenium ions led to the formation of ring-opened C-5,C-6-dioxolenium ions, indicating that the C-1,C-6-dioxolenium ions were not stable enough. The C-6-DMNPA-derived C-1,C-6-dioxolenium ions can be stabilized by the appended nitro group. The existence of these species in the gas phase indicates that these species may form more readily than the corresponding dioxolenium ions derived from “typical” acyl groups. A crucial aspect of this study is the isomer population analysis, which quantified the C-1,C-6-dioxolenium ion as the major ion species over the ring-opened C-5,C-6-dioxolenium ion. The unique structure of the DMNPA group enables the dual participation mechanism and may shift the glycosylation reaction mechanism toward the side of the ionic intermediates, providing more of the α-products.

## Supporting Information Available

Procedures for synthesis and analytical data of all new compounds; tandem-MS IR ion spectroscopy methods; and computational details for simulation of IR spectra (PDF)
