# Supplementary material for: Stabilization of Glucosyl Dioxolenium Ions by “Dual Participation” of the 2,2-Dimethyl-2-(ortho-nitrophenyl)acetyl (DMNPA) Protection Group for 1,2-cis-Glucosylation
Source: J Org Chem. 2022 Jun 24;87(14):9139–47. doi: 10.1021/acs.joc.2c00808 (PMC9295149; doi:10.1021/acs.joc.2c00808)
Supplement: Supplementary file 1 — jo2c00808_si_001.pdf [file jo2c00808_si_001.pdf]

Supplementary Information

"Stabilization of glucosyl dioxolenium ions by 'dual participation' of the 2,2-dimethyl-2-(*ortho*-nitrophenyl)acetyl (DMNPA) protection group for 1,2-*cis*-glucosylation**."**

Wouter A. Remmerswaal[1,5], Kas J. Houthuijs[2,5], Roel van de Ven[2], Hidde Elferink[3], Thomas Hansen[1,4], Giel Berden[2], Herman S. Overkleeft[1], Gijsbert A. van der Marel[1], Floris P.J.T. Rutjes[3], Dmitri V. Filippov[1], Thomas J. Boltje[3], Jonathan Martens[2,6], Jos Oomens[2,6], Jeroen D. C. Codée[1,6]

[1]Leiden University, Leiden Institute of Chemistry, Einsteinweg 55, 2333 CC Leiden, The Netherlands. [2]Radboud University, Institute for Molecules and Materials, FELIX Laboratory, Toernooiveld 7, 6525 ED Nijmegen, The Netherlands. [3]Radboud University, Institute for Molecules and Materials, Heyendaalseweg 135, 6525 AJ Nijmegen, The Netherlands. [4]Departament de Química Inorgànica i Orgànica & IQTUB, Universitat de Barcelona, 08028 Barcelona, Spain. [5]These authors contributed equally: Wouter A. Remmerswaal, Kas J. Houthuijs. [6]Corresponding author, email addresses: jonathan.martens@ru.nl; jos.oomens@ru.nl; jcodee@chem.leidenuniv.nl.

Table of contents

Supplementary Methods S2

Tandem-MS combined with IR ion spectroscopy S2

Simulation of IR spectra S2

Glucosyl cation spectra S3

Effect of basis set and dispersion correction on computed vibrational spectra S4

Glucosyl cation labelled spectra S5

Glucosyl cation isomer population analyses (IPA) S6

Coordinates of computed structures S7

Organic Synthesis S11

General experimental procedures S11

Preparation of the donors 3, 5 and 7 .... S13

Preparation of donors 8 and 9 .... S18

Model glycosylation reactions S24

NMR spectra of new and selected compounds S31

Benzylated donor 3, 5, 7 NMR spectra S31

Benzylated donor intermediates NMR spectra S40

Methylated donor intermediates NMR spectra S61

Methylated donor NMR spectra S79

DMNPA reagent and intermediates NMR spectra S84

Model glycosylation NMR spectra S89

Supplementary References S131

Supplementary Methods

Tandem-MS combined with IR ion spectroscopy

IR spectra of the glucosyl cations were recorded in a quadrupole ion trap mass spectrometer (Bruker, AmaZon Speed ETD, Bremen, Germany) that was coupled to the Free-Electron Laser for Infrared eXperiments (FELIX) beamline. Protonated precursor ions ($[M + H]^+$) and the direct glucosyl cation ($[M\text{-}PhSOH]^+$) were generated from ~10^{-6} M solutions (50:50 acetonitrile:water) containing 2% ammonium acetate. These ions were isolated and in the case of the $[M + H]^+$ ion collisionally activated to yield only the $[M\text{-}PhSOH]^+$ glucosyl cation. After (re)isolation of the glucosyl cation, the ions were irradiated by a single macropulse of FELIX, with a pulse energy of 20-100 mJ and a bandwidth of about ~0.5% of the centre frequency. When the photon-energy is resonant with a vibrational transition of the ions, they undergo IR multi-photon dissociation (IRMPD. By monitoring the fragmentation yield ($\ln[I_{total} / I_{precursor}]$), while scanning the IR wavelength in the 600-1900 cm^{-1} range, an IR spectrum can be reconstructed. The resulting IR spectra are linearly corrected for frequency-dependent variations in laser power and the frequency is calibrated using a grating spectrometer.

Simulation of IR spectra

For the conformational search and subsequent geometry optimization and calculation of IR spectra a previously reported workflow has been used.[1] The SMILES code for the oxocarbenium, acetyl participation, dual participation, ether participation and ring opened ions were used as input for the workflow using the cheminformatics toolbox RDkit.[2] Conformations for the acetyl stabilization and nitro stabilization structures were generated with the oxocarbenium ion conformation search and therefore did not require their own input. The conformational searches were performed using a distance geometry algorithm, yielding 500 random structures, which subsequently were minimized using the MMFF94 classical forcefield.[3] A maximum of 50 conformers were selected based on the root means squared distance between geometries.[4] For these selected conformers a structure optimization and frequency calculation was performed on the semi-empirical PM6 level[5] using the Gaussian 16 package.[6] The remaining conformers were sorted based on their relative energies, which consisted out of an electronic and thermal part. A python script based on atomic distances was used to classify the oxocarbenium, acetyl stabilized and nitro stabilized structures that were obtained from the oxocarbenium conformational search. For each conformer a selection of 2 or 3 structures were reoptimized with the tight command using the B3LYP functional,[7–9] and the 6-31++G(d,p) basis set.[10] Subsequently, a frequency calculation was performed. The frequencies were scaled with a factor of 0.975 and a Gaussian broadening of 20 cm^{-1} was applied. To obtain reliable energies the thermal energy (T=298.15K) of the frequency calculation was combined with the electronic energy of the B3LYP optimized structures, calculated using second order Møller-Plesset perturbation theory and the 6-311++G(2d,2p) basis set.[11]

Glucosyl cation spectra

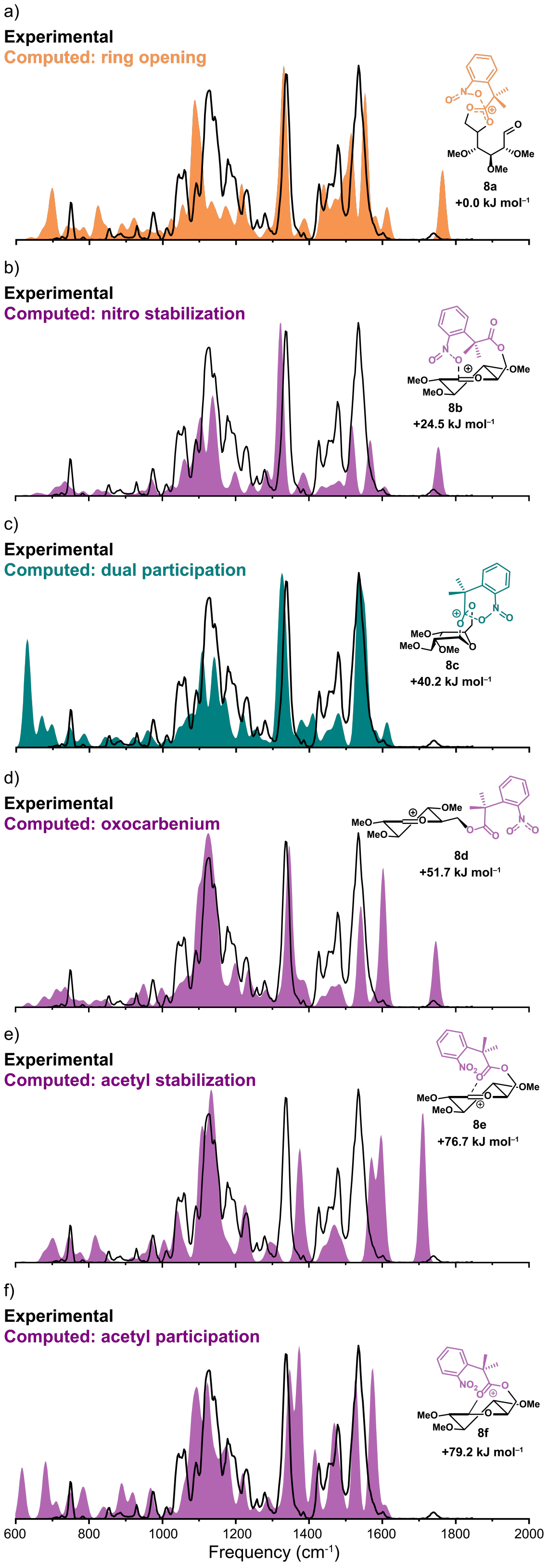

Supplementary Figure S1. Comparison of the experimental IR spectrum of the glycosyl cation of **8** with m/z 396 (black) to the calculated spectra (coloured) of the ring-opened C-5,C-6-dioxolenium ion with nitro-stabilization (a), the nitro-stabilized oxocarbenium ion (b), the C-1,C-6-dioxolenium ion with nitro-stabilization (c), the oxocarbenium ion (d), the acetyl-stabilized oxocarbenium ion (e) and the C-1,C-6-dioxolenium ion without nitro stabilization (f). Relative free energies are given. Exact coordinates of the depicted 3D structures can be found below.

Effect of basis set and dispersion correction on computed vibrational spectra

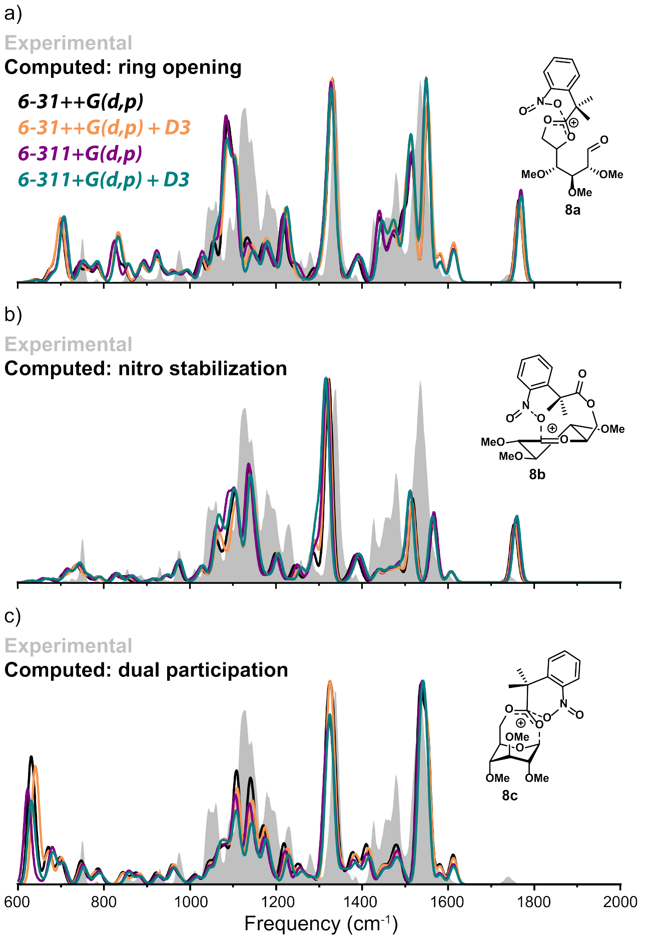

Supplementary Figure S2. The effect of basis set (6-31++G(d,p) vs 6-311+G(d,p)) and adding a dispersion correction (D3)[12]on the B3LYP computed vibrational spectra of the three lowest-energy structures. Experimental IR spectrum of the glycosyl cation of **8** with m/z 396 (gray) and the calculated spectra (coloured) of the ring-opened C-5,C-6-dioxolenium ion with nitro-stabilization (a), the nitro-stabilized oxocarbenium ion (b), the C-1,C-6-dioxolenium ion with nitro-stabilization (c). The B3LYP/6-31++G(d,p) optimized geometry served as starting point for optimization and vibrational analysis using the other methods. For the 6-31++G(d,p) and 6-311+G(d,p) basis sets scaling factors of 0.975 and 0.98 were used, respectively.

Glucosyl cation labelled spectra

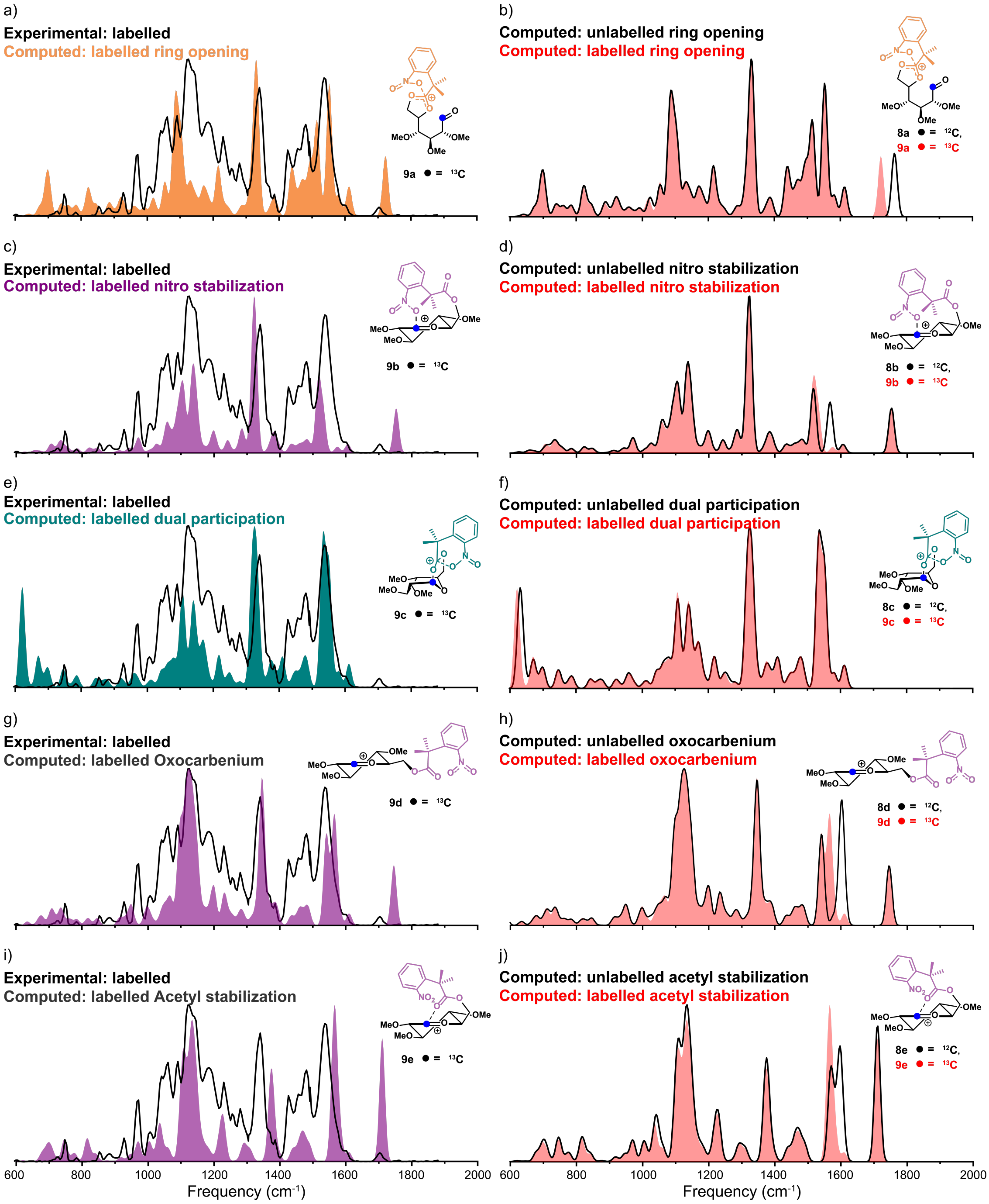

Supplementary Figure S3. Left column shows comparison of the experimental IR spectrum of ^{13}C-1 labelled glycosyl cation **9** (black) to the calculated spectra (coloured) and the right column shows comparison of the calculated spectra of both the unlabelled glycosyl cation **8** (black) and the of ^{13}C-1 labelled glycosyl cation **9** (red) of the ring-opened C-5,C-6-dioxolenium ion with nitro-stabilization (a,b), the nitro-stabilized oxocarbenium ion (c,d), the C-1,C-6-dioxolenium ion with nitro-stabilization (e,f), the oxocarbenium ion (g,h) and the acetyl-stabilized oxocarbenium ion (i,j).

Glucosyl cation isomer population analyses (IPA)

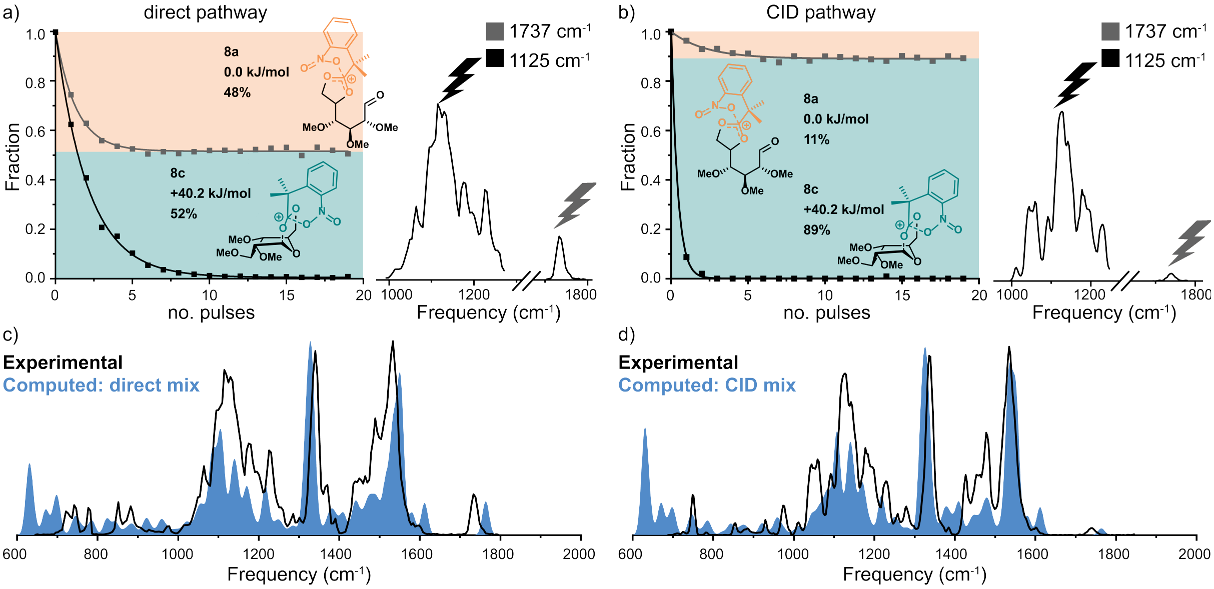

Supplementary Figure S4. Top part shows the isomer population analysis of the glycosyl cations of **8** with direct isolation of the glycosyl cation after ESI (a) and with isolation after a CID step (b). Bottom part shows the comparison of the experimental spectrum compared to a mixture of the nitro-stabilized oxocarbenium ion and the C-1,C-6-dioxolenium ion with nitro-stabilization for the direct isolation (c) and with isolation after a CID step (d), where the mixture is made using the experimentally derived populations from a) and b).

Coordinates of computed structures

All geometries were optimized with B3LYP/6-31++G(d,p) opt=tight. Energies are reported as thermal energies at MP2/6-311++G(2d,2p).

Ring opening, **8a**: +0.0 kJ mol^{-1}

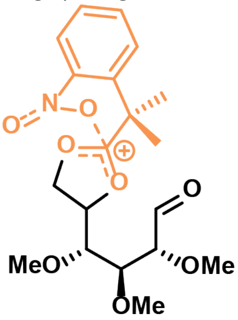

C -2.4335266523 -1.4208377637 -0.2521922569
C -2.1852020637 0.0789813141 -0.6112531015
C -3.4162923796 0.9763458086 -0.3455353985
C -4.6629041673 0.3761137719 -0.1281897419
C -5.8393676994 1.1158708062 0.0233266824
C -5.8110770518 2.5068802231 -0.0435605354
C -4.5961844917 3.1455150088 -0.2640727816
C -3.4254783613 2.3934844771 -0.4082755044
N -2.2070185027 3.1880235954 -0.6131926606
O -2.3087372524 4.3677836817 -0.9107261006
O -1.1024735220 2.6240471529 -0.4684159664
C -1.7673330219 0.1527348149 -2.1014984862
C -1.0422442547 0.4575035319 0.2901661096
O -1.2439135302 0.7334150870 1.5388677040
C 0.0575560685 0.9171597861 2.1992231589
C 1.0724374906 0.5039625264 1.1175180828
O 0.1833850600 0.2920794045 -0.0604716916
C 1.8059009263 -0.8021643423 1.4170586970
O 0.7973789187 -1.7944226190 1.5638159691
C 1.0025355679 -2.7450463544 2.6217687502
C 2.8360944249 -1.2184203790 0.3423333969
O 3.5347748610 -2.3321655421 0.8819520180
C 3.8931017677 -3.3412741785 -0.0615631646
C 3.8657286225 -0.1494036307 -0.0964257052
C 4.5877802369 0.6246992817 1.0137573458
O 4.1895244569 0.7381804565 2.1531837542
O 3.2631560981 0.8784903003 -0.8864830480
C 3.5571734803 0.8383132187 -2.2873742264
H -3.1484090130 -1.8512341941 -0.9566231460
H -2.8072324508 -1.5461725421 0.7663336863
H -1.4981880461 -1.9774898322 -0.3455659277
H -4.7361961508 -0.7015469365 -0.0771905771
H -6.7754884707 0.5938818879 0.1939795002
H -6.7174170193 3.0904410317 0.0752051664
H -4.5322637262 4.2248672183 -0.3212127330
H -1.4757907473 1.1595779646 -2.4010806784
H -2.6157106048 -0.1649108766 -2.7118467463
H -0.9302079802 -0.5210125301 -2.2987629751
H 0.1225506612 1.9671194880 2.4841321167
H 0.0579114387 0.2687040653 3.0735992422
H 1.7436221334 1.3088521228 0.8283438899
H 2.3476451005 -0.6464956053 2.3569961059
H 0.1309699012 -3.4015435065 2.6122516840
H 1.0645438504 -2.2391658085 3.5942286492
H 1.9124394392 -3.3255317965 2.4568228528
H 2.2745984826 -1.5194249305 -0.5549160006
H 4.5585180973 -2.9557337612 -0.8455992129
H 2.9997196771 -3.7760947925 -0.5290520868
H 4.4236054957 -4.1132906629 0.4975529173
H 4.6359986371 -0.6711437785 -0.6811516785
H 5.4952038858 1.1606037657 0.6717830370
H 3.0078395084 1.6643648883 -2.7409220751
H 4.6296928982 0.9749358977 -2.4689399388
H 3.2273699730 -0.1087062145 -2.7338063709

Nitro stabilization, **8b**: +24.5 kJ mol^{-1}

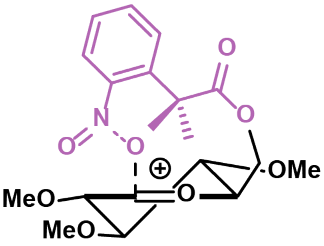

O 1.2427366951 0.1945370683 -1.8223689790
C 2.6431486509 -1.0392604344 -0.1107879176
C 2.2976949985 1.4385926889 0.0157064955
C 3.3540197281 0.3149217472 0.0503057615
C 1.3298282859 1.2419009048 -1.1209676296
C 1.8759549342 -1.1094990540 -1.4422108260
H 1.9505342203 -1.1824921293 0.7298636326
H 1.7465897303 1.4249069168 0.9662499302
H 4.0545032845 0.4555413205 -0.7858260566
H 0.7927862961 2.0966157916 -1.5306339692
H 2.5843605615 -1.2880456015 -2.2558664006
O 2.9173197517 2.6834852368 -0.2247127548
O 4.0298259452 0.3298360061 1.2823132375
O 3.5551735299 -2.1111682012 -0.2003667384
C 0.7714877838 -2.1538985554 -1.5116136333
H 1.2395723361 -3.1351822813 -1.4064586332
H 0.2419657059 -2.0881340681 -2.4639817252
C 2.3242123652 3.8073896148 0.4490778161
H 1.2916808458 3.9758023044 0.1207279514
H 2.9323716455 4.6718799165 0.1835979738

H 2.3458950179 3.6527734311 1.5342776322
C 5.3299992687 0.9406243596 1.2734368399
H 6.0014322611 0.4093347068 0.5883010509
H 5.7093670941 0.8542145672 2.2921932162
H 5.2702413266 1.9962037963 0.9903503777
C 3.8037704554 -2.8288120071 1.0202223702
H 4.4927151236 -3.6318224403 0.7564556873
H 2.8733051320 -3.2604809639 1.4103465849
H 4.2553337173 -2.1779139465 1.7726670352
O -0.1499828794 -2.0143541395 -0.4187013018
C -1.3598510490 -1.4394857767 -0.6865029729
O -1.6649512298 -1.0281773321 -1.7834521075
C -2.3114727193 -1.5065925432 0.5148325096
C -3.3013828454 -0.3207226738 0.4809365846
C -5.2873101650 1.7536716964 0.4830595967
C -3.0049024716 1.0289204648 0.1510827668
C -4.6415243852 -0.5576509331 0.8175906328
C -5.6142320649 0.4443873990 0.8346668842
C -3.9772680686 2.0409547245 0.1295800250
H -4.9514936891 -1.5627374796 1.0695006959
H -6.6318126131 0.1907401764 1.1146911683
H -3.6819561542 3.0441676333 -0.1505262874
H -6.0363189296 2.5378325866 0.4838597150
N -1.6687915642 1.4923087865 -0.1978408094
O -0.6776337891 0.8628075186 0.2390958124
O -1.5389189957 2.5020767785 -0.8864672726
C -1.5981885413 -1.5524700316 1.8881721309
H -0.9393350634 -2.4216532885 1.9463368334
H -2.3514086008 -1.6438186677 2.6757420363
H -1.0124698193 -0.6521163399 2.0748440151
C -3.0323242536 -2.8735154696 0.3025412452
H -2.2816391966 -3.6607718789 0.1923146826
H -3.6627758791 -2.8663565381 -0.5898958778
H -3.6394367238 -3.1366793669 1.1717219647

Dual participation, **8c**: +40.2 kJ mol^{-1}

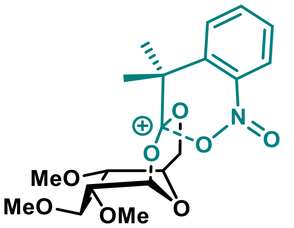

O -1.4567395516 -0.5817435198 -1.8406156710
C -2.4243808920 1.3011033060 -0.4803337289
C -2.7474577392 -1.0972469152 0.1936000324
C -3.4670315479 0.2145667566 -0.1789712629
C -1.5109716162 -1.3272618386 -0.7145733198
C -1.5215863490 0.8484272632 -1.6524602378
H -1.8314483849 1.4712287010 0.4336233533
H -2.4308823970 -1.0340173660 1.2438883995
H -4.0675315386 0.0378024126 -1.0822304026
H -1.3915591727 -2.3735670936 -0.9896695752
H -1.9642936968 1.2424307968 -2.5715306144
O -3.6561762375 -2.1596586999 0.0062881166
O -4.2789627008 0.6519753523 0.8887138957
O -2.9764484180 2.5239908377 -0.9173921984
C -0.0949703747 1.3453904390 -1.5846991128
H -0.0493650606 2.4340462390 -1.5519746195
H 0.4903919712 0.9613122669 -2.4224642596
C -3.4123964467 -3.3177881614 0.8090672708
H -2.4400889206 -3.7770933113 0.5841975883
H -4.2012479879 -4.0305276419 0.5673521805
H -3.4569447413 -3.0668167800 1.8766456683
C -5.6654307812 0.2915001741 0.8025228004
H -6.1181264930 0.7100498449 -0.1049911026
H -6.1456423222 0.7269968006 1.6799171898
H -5.7960172301 -0.7947184267 0.8101795302
C -3.2849104144 3.4851891603 0.1024931615
H -3.6220724330 4.3786173685 -0.4243847618
H -2.3874728167 3.7274654375 0.6884704330
H -4.0692748094 3.1203849190 0.7686794591
O 0.6293454182 0.9363229585 -0.3701071685
C 0.6538766363 -0.2230962148 0.2110909616
O -0.2486616740 -1.1284735191 0.1781383605
C 1.7192271130 -0.3635437641 1.2963314826
C 3.0596715014 0.2503388984 0.8317335416
C 5.6531793201 1.2722032047 0.1947035660
C 3.7766050444 -0.1561995282 -0.3213990899
C 3.7101882081 1.1908348997 1.6403568717
C 4.9758178292 1.7005007518 1.3342392289
C 5.0490323944 0.3331742112 -0.6338521309
H 3.2321853266 1.5417584935 2.5447611027
H 5.4270816873 2.4319460140 1.9970821041
H 5.5413799503 -0.0253604562 -1.5294107581
H 6.6356782809 1.6605958280 -0.0501941629
N 3.2310835603 -1.1114846165 -1.2929361675
O 1.9930426331 -1.2770075903 -1.3279751278
O 3.9953242468 -1.6986613216 -2.0421813840
C 1.9284781949 -1.8445750938 1.6988179053
H 1.0098262676 -2.2666373374 2.1115093704
H 2.7013201516 -1.8869602023 2.4698974444
H 2.2403987436 -2.4656964464 0.8584477074
C 1.1075072914 0.3805864401 2.5206814235
H 0.1320735048 -0.0519967665 2.7587597555
H 0.9812074979 1.4505722644 2.3397251658
H 1.7419499744 0.2394035711 3.3982907855

Oxocarbenium, **8d**: +51.7 kJ mol^{-1}

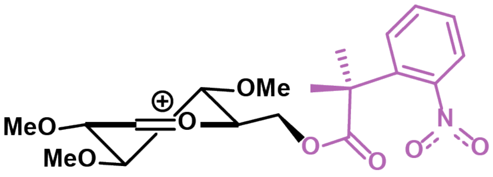

O -1.3584523679 0.0172131515 -1.6942460178
C -2.7212297214 -1.0583755932 0.1157556943
C -3.5987140738 0.8852644016 -1.2145767993
C -3.4886378068 0.2796971028 0.1990677120
C -2.3282764686 0.7537605298 -1.9915892126
C -1.3028907089 -0.8117271012 -0.4220671507

H -3.2647666700 -1.7491279454 -0.5492603724
H -4.3442605981 0.2816597451 -1.7681799831
H -2.9241036230 0.9744480285 0.8360403088
H -2.1842878744 1.3550114626 -2.8930804413
H -0.7346843369 -0.1672251174 0.2508879351
O -3.9242090605 2.2488809780 -1.2163799351
O -4.7957031952 0.0949810837 0.6791988553
O -2.5551035625 -1.6194446907 1.3945198663
C -0.4968332558 -2.0512005157 -0.7755355140
H -0.9588930435 -2.6100888809 -1.5929037118
H -0.4464276791 -2.6686415088 0.1253286774
C -5.2368741580 2.5848600229 -1.7101440939
H -5.3236137065 2.3564828522 -2.7788766514
H -5.3428211625 3.6587932660 -1.5597777443
H -6.0032706924 2.0472383495 -1.1433311123
C -5.0327559454 0.5434093033 2.0258697996
H -4.4003564148 0.0048464418 2.7380932245
H -6.0829556909 0.3337341173 2.2308313783
H -4.8520106814 1.6211875646 2.1097056494
C -3.3692062411 -2.7658907412 1.6892517042
H -3.1046786581 -3.0657583645 2.7034163011
H -4.4338719666 -2.5172002980 1.6391203763
O 0.8118504781 -1.7065172717 -1.2352837292
C 1.7070951016 -1.3470606664 -0.2659888340
O 1.4240051888 -1.3883434813 0.9121222265
C 3.0939281698 -1.0233681478 -0.8266627609
C 3.7809018174 0.0604750931 0.0365028714
C 5.2314931721 1.9830197259 1.5899320875
C 3.1657187105 1.1998736346 0.6121144783
C 5.1580566997 -0.0509517433 0.2793103142
C 5.8779645061 0.8856345561 1.0236365455
C 3.8648352274 2.1299256347 1.3905939745
H 5.6971849953 -0.9009700701 -0.1174954326
H 6.9448224437 0.7444834574 1.1656812789
H 3.3225351959 2.9646903455 1.8169724696
H 5.7780018516 2.7126395836 2.1775560603
N 1.7412593914 1.5082801320 0.4450739365
O 1.1719649843 1.1305581884 -0.5939599851
O 1.1656277197 2.1417411294 1.3255649457
C 3.0778144139 -0.5781483007 -2.3087686569
H 2.6502960401 -1.3607326736 -2.9400563623
H 4.1062408285 -0.3998297986 -2.6352469402
H 2.5044550344 0.3387202397 -2.4461451136
C 3.8321349521 -2.3942068811 -0.7368923922
H 3.2349713144 -3.1590775455 -1.2417251412
H 3.9901860462 -2.7022044088 0.2995276328
H 4.7944870116 -2.3550869779 -1.2520753409
H -3.1484460373 -3.5901054113 0.9992593874

Acetyl stabilization, **8e**: +76.7 kJ mol-1

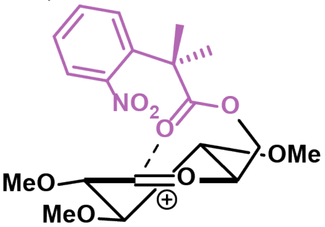

O -2.3303580813 -1.8047206126 -1.3717767755
C -2.3645097036 0.7423333270 -1.1024888787
C -3.3667343661 -0.8130996092 0.6037847916
C -3.6329415254 0.4351791858 -0.2843159693
C -2.8705162764 -1.9362181932 -0.2454529417
C -1.8746589946 -0.4717763998 -1.9150678468
H -1.5851175717 1.0712514611 -0.4041866190
H -2.5847542349 -0.5518090721 1.3343199642
H -4.4660999314 0.2154195081 -0.9675782066
H -3.0968455119 -2.9699373770 0.0255390891
H -2.3760946441 -0.4632061834 -2.8852106963
O -4.5541693922 -1.2541391053 1.2115816173
O -3.9142885667 1.5453775117 0.5204313091
O -2.5898406572 1.7245137589 -2.0884302325
C -0.3654373600 -0.5795292183 -2.1597728506
H -0.1337747058 0.0288636135 -3.0353226674
H -0.1054912604 -1.6207520740 -2.3744655193
C -4.4184575566 -1.7123508233 2.5691070970
H -3.7643654947 -2.5910320813 2.6315859434
H -5.4211629676 -1.9850008976 2.8968384648
H -4.0203405802 -0.9111338887 3.2022995105
C -5.3054337841 1.8849771725 0.6630450023
H -5.7454954103 2.1188246903 -0.3133824816
H -5.3321321956 2.7721056939 1.2962287672
H -5.8620541065 1.0725873503 1.1391548498
C -2.2355887679 3.0741980879 -1.7326069219
H -2.4277207605 3.6721555380 -2.6236404764
H -1.1718414469 3.1366921513 -1.4736237659
H -2.8440829729 3.4338884153 -0.8997480034
O 0.5070427424 -0.0567598868 -1.1463940414
C 0.5561714383 -0.6394126996 0.0756795148
O -0.2957737534 -1.4307736120 0.4454782149
C 1.7267299503 -0.1160179009 0.9224164521
C 3.0390878393 -0.5205245028 0.2101359086
C 5.3445145117 -1.4138807439 -1.2160000524
C 4.2319870442 0.2200818407 0.1650818109
C 3.0744711005 -1.7553453295 -0.4617990610
C 4.1978462169 -2.2065861774 -1.1535778641
C 5.3569619280 -0.1905421065 -0.5538498399
H 2.1957469294 -2.3949214978 -0.4367846461
H 4.1726608202 -3.1735830134 -1.6459500921
H 6.2345130540 0.4463207562 -0.5651362581
H 6.2219619305 -1.7458496615 -1.7605179314
N 4.4255545618 1.4635239074 0.9382891666
O 4.2423256434 1.4004404726 2.1553684921
O 4.7986478442 2.4621200264 0.3291066179
C 1.6736514548 -0.7708927572 2.3199971044
H 0.7422611324 -0.5133294442 2.8328017554
H 2.5128041943 -0.4030851264 2.9130106105
H 1.7395229351 -1.8593838559 2.2539312936
C 1.4650776746 1.4140910128 1.0455422030
H 0.4129979752 1.5849842068 1.2986861590
H 1.6832123475 1.9436285090 0.1156501093
H 2.0552683120 1.8433406541 1.8532028198

Acetyl participation, **8f**: +79.2 kJ mol^{-1}

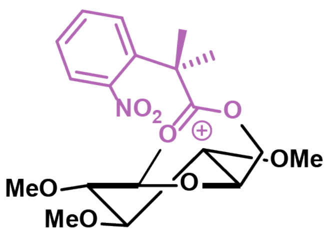

O	-0.0818839111	-0.4835011618	-0.0125474825
C	0.4488723605	-0.0741342138	1.0775009012
O	-0.2256274647	0.4836355635	2.0314983269
C	-1.6945100126	0.3514328966	2.0896083613
C	-2.3996522179	1.2365616117	1.0510103749
C	-3.7420619236	0.6301062602	0.5947635168
O	-4.4281528680	1.5221388086	-0.2491774155
C	-5.1991162755	2.5248685479	0.4144170104
C	-3.5250828876	-0.6858078095	-0.1905612999
O	-3.3301267601	-1.7259447168	0.7644262685
C	-3.8217497278	-3.0093060074	0.3595749912
C	-2.3400063439	-0.6424996378	-1.1915400108
O	-2.8258994450	-0.2025490478	-2.4423579464
C	-2.1550623569	-0.7439797946	-3.5842931577
C	-1.2173579726	0.3361804970	-0.7981369840
O	-1.5277472410	1.4358938536	-0.0870220054
C	1.9239381236	-0.3520819062	1.3245577774
C	2.3903517839	0.2145297197	2.6845874706
C	1.9503521898	-1.9179123786	1.3685105573
C	2.7112574319	0.3165066950	0.1743572871
C	2.2647626791	1.5695364404	-0.2818773629
C	2.8884405781	2.2563872154	-1.3235011075
C	3.9909787068	1.6935857749	-1.9673879859
C	4.4715409513	0.4604112820	-1.5368824120
C	3.8523206260	-0.1909554737	-0.4682643980
N	4.5388296618	-1.4173959286	-0.0153627616
O	4.8810566038	-1.4599308288	1.1679209886
O	4.7594164796	-2.2879562482	-0.8510976315
H	-1.9186424305	-0.7076393969	1.9551333402
H	-1.9408399989	0.6555146229	3.1063408741
H	-2.5411160167	2.2475152186	1.4342866168
H	-4.3410240016	0.3781514522	1.4834282886
H	-5.9396201931	2.0694160117	1.0851579387
H	-4.5699945470	3.2213014953	0.9848014196
H	-5.7137220555	3.0809176546	-0.3695294080
H	-4.4308294969	-0.8571318591	-0.7821655890
H	-3.3078640088	-3.3758541900	-0.5382235344
H	-4.9004352300	-2.9658399058	0.1685087960
H	-3.6248662580	-3.6916548000	1.1871402454
H	-1.9098788625	-1.6503785414	-1.2630930702
H	-2.6651789356	-0.3358083221	-4.4569662235
H	-1.0972029133	-0.4504380179	-3.6224277741
H	-2.2267892096	-1.8391979256	-3.5953290235
H	-0.6266259307	0.6372141557	-1.6616179894
H	2.2707306285	1.2994583113	2.7328080966
H	1.8370719370	-0.2374297050	3.5119584222
H	3.4487054333	-0.0232698772	2.8031484274
H	1.8162881664	-2.3549296339	0.3770154205
H	1.1499388421	-2.2847262722	2.0193695745
H	2.8912106151	-2.2581352158	1.7971667024
H	1.4065761719	2.0370923902	0.1963268850
H	2.5101835778	3.2267185325	-1.6288697601
H	4.4814337621	2.2116550730	-2.7844742034
H	5.3364201865	0.0004357320	-2.0019173427

Organic Synthesis

General experimental procedures

All chemicals (Acros, Fluka, Merck, and Sigma-Aldrich) were used as received unless stated otherwise. Dichloromethane was stored over activated 4 Å molecular sieves (beads, 8-12 mesh, Sigma-Aldrich). Before use traces of water present in the donor, diphenyl sulfoxide (Ph_2SO) and tri-*tert*-butylpyrimidine (TTBP) were removed by co-evaporation with dry toluene. The acceptors were stored in stock solutions (DCM, 0.5 M) over activated 3 Å molecular sieves (rods, size 1/16 in., Sigma-Aldrich). Trifluoromethanesulfonic anhydride (Tf_2O) was distilled over P_2O_5 and stored at –20 °C under a nitrogen atmosphere. Overnight temperature control was achieved by an FT902 Immersion Cooler (Julabo). Column chromatography was performed on silica gel 60 Å (0.04 – 0.063 mm, Screening Devices B.V.). Size exclusion chromatography was carried out on Sephadex$_{TM}$ (LH-20, GE Healthcare Life Sciences) by isocratic elution with DCM:MeOH (1:1, v:v). TLC-analysis was conducted on TLC Silica gel 60 (Kieselgel 60 F_{254}, Merck) with UV detection by (254 nm) and by spraying with 20% sulfuric acid in ethanol followed by charring at ± 150 °C or by spraying with a solution of $(NH_4)_6Mo_7O_{24}\cdot H_2O$ (25 g/l) and $(NH_4)_4Ce(SO_4)_4\cdot 2H_2O$ (10 g/l) in 10% sulfuric acid in water followed by charring at ± 250 °C. High-resolution mass spectra were recorded on a Thermo Finnigan LTQ Orbitrap mass spectrometer equipped with an electrospray ion source in positive mode (source voltage 3.5 kV, sheath gas flow 10, capillary temperature 275 °C) with resolution R=60.000 at m/z=400 (mass range = 150-4000). ^{1}H and $^{13}C\{^{1}H\}$ NMR spectra were recorded on a Bruker AV-400 NMR instrument (400 and 101 MHz respectively), a Bruker AV-500 NMR instrument (500 and 126 MHz respectively), or a Bruker AV-850 NMR instrument (850 and 214 MHz respectively). For samples measured in $CDCl_3$ chemical shifts (δ) are given in ppm relative to tetramethylsilane as an internal standard or the residual signal of the deuterated solvent. Coupling constants (*J*) are given in Hz. To get better resolution of signals with small coupling constants or overlapping signals a gaussian window function (LB ± -1 and GB ± 0.5) was used on the $_{1}H$ NMR spectrum. All given $_{13}C$ APT spectra are proton decoupled. NMR peak assignment was made using COSY, HSQC. If necessary additional, HMBC and HMBC-GATED experiments were used to elucidate the structure further. The anomeric product ratios were based on the integration of ^{1}H NMR. If the stereochemistry of the coupled product was not completely clear a deprotection step was used to verify the stereochemistry.

General procedure I: Installation of the DMNPA protecting group. Based on the protocol by Liu *et al*,[13] a suspension of the glycoside, DMNPAA (1.3 eq.) and 5Å molecular sieves in dry DCM was cooled to −40 °C under inert atmosphere. TMSOTf (2 eq.) was added and the reaction was stirred for 40 minutes. After completion, leftover TMSOTf and leftover DMNPA was quenched with benzylamine (7 eq). The reaction mixture was diluted with DCM and washed with $NaCHO_3$ and brine. The organic layer was dried with $MgSO_4$, filtered and concentrated in vacuo. The resulting residue was purified by silica flash column chromatography.

General procedure II: Synthesis of Phenyl per-*O*-Acetyl-1-thio-β-ᴅ-glycopyranoside. To a solution of Ac_2O (7.6 eq) and a few drops of $HClO_4$ the glycoside (1 eq) is slowly dissolved portionwise. After all of the glycoside was dissolved, the reaction mixture was stirred for 20 minutes. HBr (33 Wt% in AcOH, 4.4 eq) was added and the is reaction was stirred until completion. The reaction mixture was diluted with DCM and washed with cold H_2O, and saturated $NaHCO_3$ (aq). The combined organic layers were dried over $MgSO_4$ and concentrated under reduced pressure, yielding the crude acetobromo-α-ᴅ-glycoside. The crude product and thiophenol (1.1 eq) were dissolved in dry DMF (0.5M). The reaction mixture was cooled to 0 °C, NaH (60% dispersion in mineral oil, 1.1 eq was added) and the reaction was stirred for 17 h under inert atmosphere. The reaction was cooled to 0 °C, quenched with H_2O and diluted with DCM. The reaction mixture was washed with NaOH (1M), dried over $MgSO_4$ and concentrated in vacuo.

General procedure III: Selective protection of C-6 with a 2-naphthylmethyl ether (NAP) protecting group. Traces of solvent were removed from the glycoside by co-evaportation with toluene, and subsequently dissolved in toluene (0.4 M). Dibutyltin oxide (1.2 eq) was added, and the resulting suspension was heated with an oil bath and refluxed for 1 h. The reaction mixture was concentrated in vacuo, coevaporated with toluene and dissolved in DMF (0.5 M). 2-(bromomethyl)naphthalene (1.5 eq) and cesium fluoride (1.5 eq) were added and it was stirred for 17 h. The reaction mixture was diluted with DCM and washed with saturated $NaHCO_3$ (aq). The resulting tin paste was filtered from the solution using a Buchner funnel, and the filtrate was dried over $MgSO_4$. It was filtered off, and concentrated in vacuo.

General procedure IV: Removal of the naphthylmethyl ether (NAP) protecting group. The 2-naphthylmethyl ether (NAP) protected glycoside was dissolved in DCM:H_2O (9:1, v:v, 0.17M), after which 2,3-Dichloro-5,6-dicyano-1,4-benzoquinone (1.5 eq) was added. The reaction was protected from light and stirred for 1 h. The reaction was quenched by addition of saturated $Na_2S_3O_3$ (aq) and filtered. The bi-phasic mixture was extracted with DCM. The organic layers were combined and washed with saturated $NaHCO_3$ (aq), dried over $MgSO_4$, filtered and concentrated in vacuo.

General procedure V: Removal of the benzylidene protecting group. The benzylidene protected glycoside is dissolved in DCM:MeOH (3:7, v:v, 0.05M) of which the acidity is adjusted to pH=2 with *p*-TsOH·H_2O. It was heated to 50 °C with an oil bath and stirred for 17 h. The reaction was neutralized with Et_3N and concentrated in vacuo.

General procedure VI: pre-activation Tf_2O/Ph_2SO based *O*-glycosylation. A solution of the donor (100 µmol), Ph_2SO (26 mg, 130 µmol, 1.3 eq.) and TTBP (62 mg, 250 µmol, 2.5 eq.) in DCM (2 mL, 0.05 M) was stirred over activated 3 Å molecular sieves (rods, size 1/16 in., Sigma-Aldrich) for 30 min under an atmosphere of N_2. The solution was cooled to −80 °C and Tf_2O (22 µl, 130 µmol, 1.3 eq.) was slowly added to the reaction mixture. The reaction mixture was allowed to warm to −60 °C in approximately 45 min, followed by cooling to −80 °C and the addition of the acceptor (200 µmol, 2 eq.) in DCM (0.4 mL, 0.5 M). The reaction was allowed to warm up to −60 °C and stirred for an additional 18 h at this temperature till full reaction completion was observed. The reaction was quenched with sat. aq. $NaHCO_3$ at −60 °C and diluted with DCM (5 mL). The resulting solution was washed with H_2O and brine, dried over $MgSO_4$, filtered and concentrated under reduced pressure. Purification by column chromatography yielded the corresponding *O*-coupled glycoside.

Preparation of the donors 3, 5 and 7

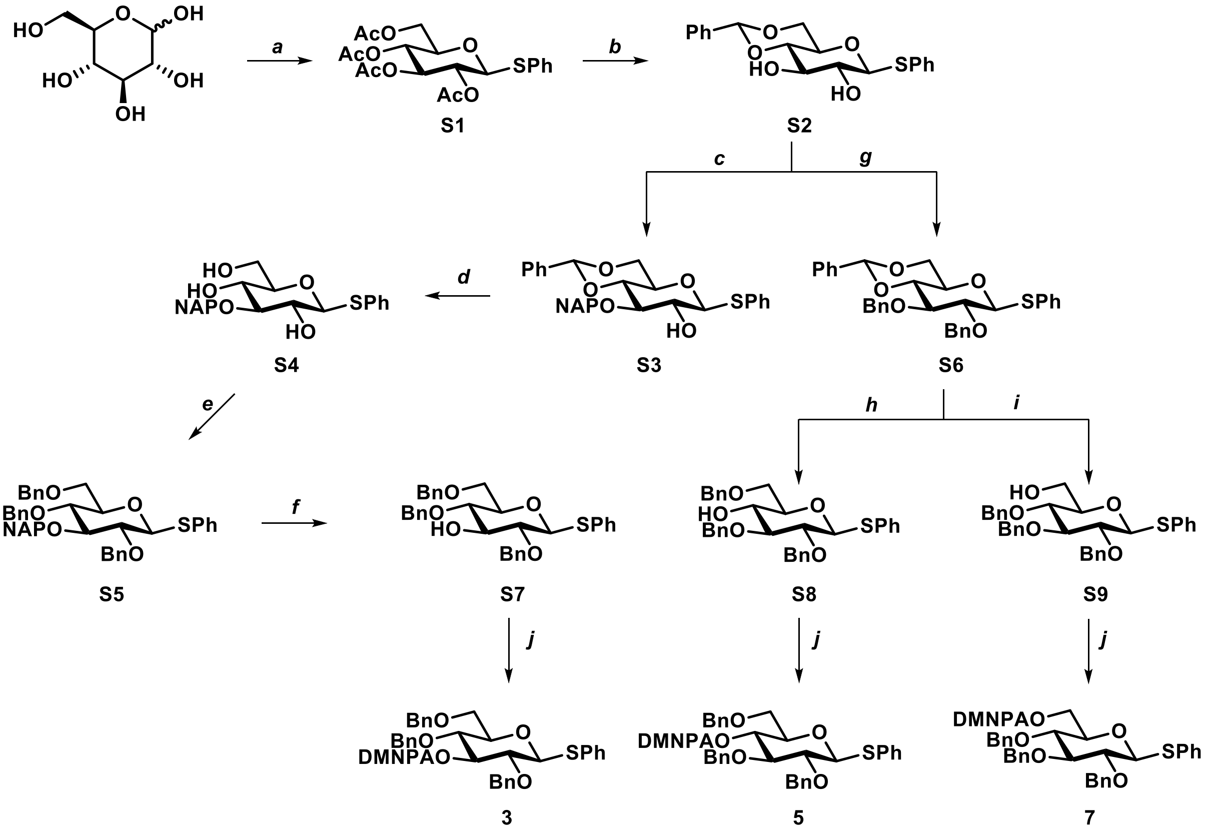

Supplementary scheme S1. Glucoside donor 3,5 and 7 synthesis. *Reagents and conditions:* a) 1. Ac_2O, HCl_4O, HBr; 2. HSPh, NaH, DMF, **S1**: 59%; b) 1. NaOMe, MeOH; 2. benzaldehyde dimethyl acetal, *p*-TsOH·H_2O, acetonitrile, 60 °C, **S2**: 93%; c) 1. Dibutyltin(IV) oxide, toluene, reflux; 2. 2-(bromomethyl)naphthalene, CsF, DMF, **S3**: 60%; d) *p*-TsOH·H_2O, DCM:MeOH, **S4**: quant; e) BnBr, NaH, DMF, 0 °C to rt, **S5**: 88%; f) DDQ, DCM:H_2O, **S7**: quant; g) BnBr, NaH, DMF, 0 °C to rt, **S6**: 93%; h) $NaCNBH_4$, HCl in dioxane, THF, **S8**: 87%; i) $CoCl_2$ in BH_3·THF, **S9**: 89%; j) DMNPAA, TMSOTf, DCM, -40 °C, **3**: 98%, **5**: 94%, **7**: quant.

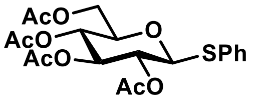

C{^{1}H} NMR **Phenyl 2,3,4,6-tetra-*O*-acetyl-1-thio-β-ᴅ-glucopyranoside (S1).** The title compound was prepared from ᴅ-Glucose (50 g, 278 mmol) via general procedure II. Crystallization from boiling EtOH yielded the title compound (63.7 g, 144.6 mmol, 59%) as a white solid. TLC: R_f 0.36, (Pentane:EtOAc, 70:30, v:v); ^{1}H NMR (400 MHz, $CDCl_3$, HH-COSY, HSQC, HMBC-Gated) δ 7.54 – 7.45 (m, 2H, CH_{arom}), 7.38 – 7.27 (m, 3H, CH_{arom}), 5.23 (t, *J* = 9.3 Hz, 1H, H-4), 5.05 (t, *J* = 9.8 Hz, 1H, H-3), 4.98 (t, *J* = 9.8 Hz, 1H, H-2), 4.71 (d, *J* = 10.1 Hz, 1H, H-1), 4.27 – 4.14 (m, 2H, H-6, H-6), 3.73 (ddd, *J* = 10.1, 5.0, 2.7 Hz, 1H, H-5), 2.12 – 2.06 (m, 6H, 2x CH_3 Ac), 2.02 (s, 3H, CH_3 Ac), 2.00 (s, 3H); ^{13}C{^{1}H} NMR (101 MHz, $CDCl_3$, HSQC, HMBC-Gated) δ 170.7, 170.3, 169.5, 169.4 (C=O Ac), 133.3 (CH_{arom}), 131.8 ($C_{q\text{-}arom}$), 129.1, 128.6 (CH_{Arom}), 85.9 (C-1), 75.9 (C-5), 74.1 (C-4), 70.0 (C-2), 68.3 (C-3), 62.3 (C-6), 20.9, 20.9, 20.7, 20.7 (CH_3 Ac); ^{13}C-GATED NMR (101 MHz, $CDCl_3$) δ 85.9 ($J_{H1\text{-}C1}$ = 156 Hz, β); HRMS (ESI) M/Z: [M + Na]$^+$ Calcd for $C_{20}H_{24}O_9NaS^+$ 463.1033; Found 463.1032.

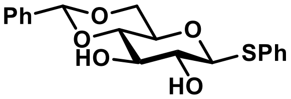

Phenyl 4,6-*O*-benzylidene-1-thio-β-ᴅ-glucopyranoside (S2). **S1** (11.9 g, 26.9 mmol) was dissolved in MeOH (50 ml, 0.5M). NaOMe in MeOH (25 % Wt, 1.0 ml, 4.37 mmol, 0.16 eq) was added and the reaction was stirred for 32 hrs. The reaction mixture was acidified to pH=4 using Amberlite H^+, filtered and concentrated in vacuo. Traces of MeOH were removed through co-evaporating with toluene, yielding a white solid. The crude product, benzaldehyde dimethyl acetal (4.92 g, 4.85 ml, 32.3 mmol, 1.2 eq) and *p*-TsOH·H_2O (0.512 g, 2.69 mmol, 0.1 eq) were dissolved in acetonitrile (50 ml, 0.5M), heated to 60 °C with a water bath and gently concentrated under reduced pressure. After all acetonitrile and formed MeOH were evaporated, the procedure was repeated until TLC indicated full conversion, subsequently the reaction mixture was quenched using Et_3N (0.41 g, 0.56 ml, 4.04 mmol, 0.15 eq) and concentrated in vacuo. Purification by crystallization from boiling acetonitrile yielded the title compound (9.0 g, 25.0 mmol, 93%) as a white solid. TLC: R_f 0.23, (DCM:MeOH, 90:10, v:v); ^{1}H NMR (400 MHz, CD_3OD, HH-COSY, HSQC, HMBC) δ 1H NMR (400 MHz, MeOD) δ 7.56 – 7.23 (m, 10H, CH_{arom}), 5.55 (s, 1H, C*H*Ph), 4.69 (d, J = 9.8 Hz, 1H, H-1), 4.26 (dd, J = 10.3, 4.7 Hz, 1H, H-6), 3.73 (t, J = 10.0 Hz, 1H, H-6), 3.64 (t, J = 8.7 Hz, 1H, H-3), 3.52jjj – 3.39 (m, 2H, H-4, H-5), 3.32 – 3.24 (m, 1H, H-2).; ^{13}C{^{1}H} NMR (101 MHz, $CDCl_3$, HSQC, HMBC) δ 137.7, 132.9 ($C_{q\text{-}arom}$), 132.1, 128.6, 127.6, 127.4, 126.1 (CH_{arom}), 101.5 (CHPh), 88.4 (C-1), 80.5 (C-4), 74.7 (C-3), 73.0 (C-2), 70.3 (C-5), 68.2 (C-6); HRMS (ESI) M/Z: $[M + H]^+$ Calcd for $C_{19}H_{21}O_5S^+$ 361.1104; Found 361.1102.

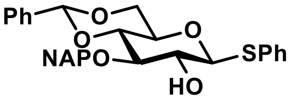

Phenyl 3-*O*-(2-naphthyl)methyl-4,6-*O*-benzylidene-1-thio-β-ᴅ-glucopyranoside (S3). The title compound was prepared from **S2** (9.0 g, 25.0 mmol) via general procedure III. Crystallization from boiling EtOAc yielded the title compound (7.5 g, 15.0 mmol, 60%) as a white solid. TLC: R_f 0.55, (pentane:EtOAc, 80:20, v:v); ^{1}H NMR (500 MHz, DMSO-d_6, HH-COSY, HSQC) δ 7.91 – 7.24 (m, 17H, CH_{arom}), 5.97 (d, J = 6.7 Hz, 1H, OH), 5.70 (s, 1H, C*H*Ph), 5.01 – 4.97 (m, 2H, 2x C*H*H Nap), 4.94 (d, J = 9.8 Hz, 1H, H-1), 4.24 (dd, J = 10.0, 4.8 Hz, 1H, H-6), 3.77 – 3.59 (m, 4H, H-3, H-4, H-5, H-6), 3.49 – 3.41 (m, 1H, H-2); ^{13}C{^{1}H} NMR (126 MHz, DMSO-d_6, HSQC) δ 137.7, 136.7, 133.6, 132.8, 132.4 ($C_{q\text{-}arom}$), 130.4, 129.0, 128.8, 128.1, 127.7, 127.5, 126.9, 126.0, 126.0, 126.0, 125.7 (CH_{arom}), 100.2 (CHPh), 87.4 (C-1), 82.3 (C-3), 80.1 (C-4), 73.7 (CH_2 Nap), 72.6 (C-2), 69.4 (C-5), 67.8 (C-6); HRMS (ESI) M/Z: $[M + NH_4]^+$ Calcd for $C_{30}H_{32}NO_5S^+$ 518.1996; Found 518.1995.

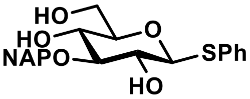

Phenyl 3-*O*-(2-naphthyl)methyl-1-thio-β-ᴅ-glucopyranoside (S4). The title compound was synthesized from **S3** (7.5 g, 15.0 mmol) via general procedure V. Flash column chromatography (100:0 → 90:10, DCM:MeOH v:v) yielded the title compound (6.20 g, 15.1 mmol, quant) as a white solid. TLC: R_f 0.30, (DCM:MeOH, 95:5, v:v); ^{1}H NMR (300 MHz, $CDCl_3$, HH-COSY, HSQC) δ 7.92 – 7.09 (m, 12H, CH_{arom}), 5.11 (d, J = 11.6 Hz, 1H, C*H*H Nap), 4.97 (d, J = 11.8 Hz, 1H, C*H*H Nap), 4.58 (d, J = 9.5 Hz, 1H, H-1), 3.96 – 3.71 (m, 2H, H-6, H-6), 3.69 – 3.55 (m, 1H, H-4), 3.56 – 3.32 (m, 3H, H-2, H-3, H-5), 3.21 (d, J = 3.5 Hz, 1H, OH-4), 2.79 – 2.56 (m, 2H, OH-2, OH-6); ^{13}C{^{1}H} NMR (80 MHz, $CDCl_3$, HSQC) δ 133.4, 133.1, 132.1 ($C_{q\text{-}arom}$), 129.2, 129.0, 128.5, 128.2, 128.1, 127.8, 127.0, 126.3, 126.1, 126.0, 126.0 (CH_{arom}), 88.5 (C-1), 85.2 (C-3), 79.7 (C-5), 75.0 (CH_2 Nap), 72.7 (C-2), 70.3 (C-4), 62.6 (C-6); HRMS (ESI) M/Z: $[M + NH_4]^+$ Calcd for $C_{23}H_{28}NO_5S^+$ 430.1683; Found 430.1686.

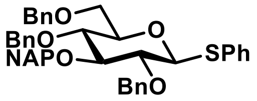

Phenyl 3-*O*-(2-naphthyl)methyl-2,4,6-tri-*O*-benzyl-1-thio-β-ᴅ-glucopyranoside (S5). S4 (6.19 g, 15.0 mmol) was dissolved in dry DMF (75 ml, 0.2M) and cooled to 0 °C. Under inert atmosphere, NaH (60 Wt % dispersion in mineral oil, 2.70 g, 67.5 mmol, 4.5 eq) was added portion wise and the reaction mixture was stirred for 10 minutes. Benzyl bromide (6.70 ml, 9.62 g, 56.3 mmol, 3.75 eq) was added and the reaction was stirred for 5 h while allowing to warm up to RT. Subsequently, the reaction was cooled to 0 °C and quenched with H_2O. The reaction mixture was diluted with H_2O and extracted with DCM. The organic layers were combined, washed with brine, dried over $MgSO_4$ and concentrated in vacuo. Flash column chromatography (95:5 → 80:20, pentane:EtOAc v:v) yielded the title compound (9.05 g, 13.3 mmol, 88%) as a white solid. TLC: R_f 0.66, (pentane:EtOAc, 80:20, v:v); ^{1}H NMR (400 MHz, $CDCl_3$, HH-COSY, HSQC) δ 7.88 – 7.12 (m, 27H, CH_{arom}), 5.05 (d, J = 11.1 Hz, 1H, C*H*H Bn), 4.99 (d, J = 11.1 Hz, 1H, C*H*H Bn), 4.91 (d, J = 10.3 Hz, 1H, C*H*H Nap), 4.85 (d, J = 10.9 Hz, 1H, C*H*H Bn), 4.75 (d, J = 10.2 Hz, 1H, C*H*H Nap), 4.69 (d, J = 9.7 Hz, 1H, H-1), 4.65 – 4.52 (m, 3H, 3x C*H*H Bn), 3.85 – 3.63 (m, 4H, H-4, H-5, H-6, H-6), 3.60 – 3.47 (m, 2H, H-2, H-3); ^{13}C{^{1}H} NMR (101 MHz, $CDCl_3$, HSQC) δ 138.4, 138.2, 136.0, 134.0,133.4, 133.1 ($C_{q\text{-}arom}$), 132.1, 129.0, 128.6, 128.5, 128.3, 128.3, 128.1, 128.0, 128.0, 127.9, 127.8, 127.7, 127.6, 126.6, 126.2, 126.0, 126.0 (CH_{arom}), 87.6 (C-1), 86.9 (C-4), 81.0 (C-2), 79.2 (C-3), 77.9 (C-5), 76.0 (CH_2 Bn), 75.6 (CH_2 Nap), 75.2, 73.6 (CH_2 Bn), 69.1 (C-6); HRMS (ESI) M/Z: [M + NH_4]$^+$ Calcd for $C_{44}H_{46}NO_5S^+$ 700.3091; Found 700.3088.

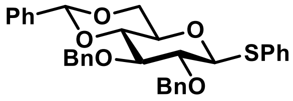

Phenyl 2,3-di-*O*-benzyl-4,6-*O*-benzylidene-1-thio-β-ᴅ-glucopyranoside (S6). S2 (5.08 g, 14.0 mmol) was dissolved in dry DMF (100 ml, 0.14M) and cooled to 0 °C. Under inert atmosphere, NaH (60 Wt % dispersion in mineral oil, 1.68 g, 42 mmol, 3 eq) was added portion wise and the reaction mixture was stirred for 10 minutes. Benzyl bromide (4.2 ml, 35 mmol, 2.5 eq) was added and the reaction was stirred for 17 h while allowing to warm up to RT. Subsequently, the reaction was cooled to 0 °C and quenched with H_2O. The reaction mixture was diluted with H_2O and extracted with DCM. The organic layers were combined, washed with brine, dried over $MgSO_4$ and concentrated in vacuo. Flash column chromatography (100:0 → 80:20, pentane:EtOAc v:v) yielded the title compound (7.03 g, 13.0 mmol, 93%) as a white solid. TLC: R_f 0.61, (Pentane:EtOAc, 90:10, v:v); ^{1}H NMR (400 MHz, $CDCl_3$, HH-COSY, HSQC) δ 7.61 – 7.17 (m, 20H, CH_{arom}), 5.59 (s, 1H, C*H*Ph), 4.94 (d, *J* = 11.1 Hz, 1H, C*H*H Bn), 4.89 – 4.73 (m, 4H, H-1, 3x C*H*H Bn), 4.39 (dd, *J* = 10.5, 5.0 Hz, 1H, H-6), 3.88 – 3.76 (m, 2H, H-3, H-6), 3.71 (t, *J* = 9.4 Hz, 1H, H-4), 3.56 – 3.43 (m, 2H, H-2, H-5); ^{13}C{^{1}H} NMR (101 MHz, $CDCl_3$, HSQC) δ 138.4, 138.1, 137.4, 133.2 ($C_{q\text{-}arom}$), 132.5, 129.2, 129.1, 128.6, 128.5, 128.4, 128.4, 128.3, 128.0, 128.0, 127.9, 126.1 (CH_{arom}), 101.3 (CHPh), 88.4 (C-1), 83.1 (C-3), 81.6 (C-4), 80.6 (C-2), 76.1, 75.5 (CH_2 Bn), 70.4 (C-5), 68.8 (C-6); HRMS (ESI) M/Z: [M + NH_4]$^+$ Calcd for $C_{33}H_{36}NO_5S^+$ 558.2309; Found 558.2308.

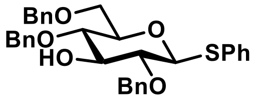

Phenyl 2,4,6-tri-*O*-benzyl-1-thio-β-ᴅ-glucopyranoside (S7). The title compound was synthesized from **S5** (9.05 g, 12.3 mmol) via general procedure IV. Flash column chromatography (95:5 → 70:30, pentane:EtOAc v:v) yielded the title compound (7,49 g, 13.3 mmol, quant) as a white wax. TLC: R_f 0.52, (pentane:EtOAc, 80:20, v:v); ^{1}H NMR (400 MHz, $CDCl_3$, HH-COSY, HSQC, HMBC) δ δ 7.67 – 7.16 (m, 20H, CH_{arom}), 4.94 (d, *J* = 11.0 Hz, 1H, C*H*H Bn), 4.78 (d, *J* = 11.2 Hz, 1H, C*H*H Bn), 4.70 – 4.49 (m, 5H, H-1, 4x C*H*H Bn), 3.83 – 3.67 (m, 3H, H-3, H-6, H-6), 3.58 – 3.43 (m, 2H, H-4, H-5), 3.37 (dd, *J* = 9.7, 8.7 Hz, 1H, H-2), 2.45 (d, *J* = 2.6 Hz, 1H, OH-3); ^{13}C{^{1}H} NMR (101 MHz, $CDCl_3$, HSQC, HMBC) δ 138.3, 138.3, 138.2, 133.9 ($C_{q\text{-}arom}$), 131.9, 129.0, 128.7, 128.6, 128.4, 128.3, 128.2, 128.0, 128.0, 127.8, 127.7, 127.5 (CH_{arom}), 87.1 (C-1), 80.7 (C-2), 78.9 (C-5), 78.7 (C-3), 77.4 (C-4), 75.2, 74.7, 73.5 (CH_2 Bn), 69.1 (C-6); HRMS (ESI) M/Z: [M + NH_4]$^+$ Calcd for $C_{33}H_{38}NO_5S^+$ 560.2465; Found 560.2466.

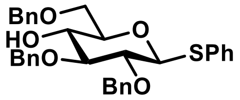

Phenyl 2,3,6-tri-*O*-benzyl-1-thio-β-D-glucopyranoside (S8). **S6** (0.54 g, 1 mmol) was co-evaporated with toluene and dissolved in dry THF (14 ml, 0.1M). $NaCNBH_4$ (0.31 g, 5.0 mmol, 5 eq) is added under protective atmosphere after which HCl in dioxane (1.3 ml, 5.2 mmol, 4.0M, 5 eq) is added dropwise. The reaction was stirred for 20 min and subsequently diluted with ice water. The reaction mixute was extracted with DCM and washed with sat. $NaHCO_3$ (aq) and brine. The combined organic layers were dried over $MgSO_4$, filtered and concentrated in vacuo. Flash column chromatography (100:0 → 80:20, pentane:EtOAc v:v) yielded the title compound (0.47 g, 0.87 mmol, 87%) as a white solid. TLC: R_f 0.56, (pentane:EtOAc, 70:30, v:v); ^1H NMR (400 MHz, $CDCl_3$, HH-COSY, HSQC, HMBC) δ 7.58 – 7.21 (m, 20H, CH_{arom}), 4.93 – 4.86 (m, 2H, 2x C*H*H Bn), 4.80 – 4.70 (m, 2Hz, 2x C*H*H Bn), 4.67 (d, J = 9.4 Hz, 1H, H-1), 4.59 – 4.50 (m, 2H, 2x C*H*H Bn), 3.81 – 3.68 (m, 2H, H-6, H-6), 3.64 (t, J = 9.0 Hz, 1H, H-4), 3.58 – 3.40 (m, 3H, H-2, H-3, H-5), 2.73 (s, 1H, OH); $^{13}C\{^1H\}$ NMR (101 MHz, $CDCl_3$, HSQC, HMBC) δ 138.5, 138.0, 138.0, 134.2 ($C_{q\text{-}arom}$), 131.9, 129.0, 128.7, 128.6, 128.5, 128.5, 128.3, 128.0, 128.0, 127.8, 127.8, 127.7, 127.6, 127.0 (CH_{arom}), 87.7 (C-1), 86.2 (C-3), 80.5 (C-2), 78.2 (C-5), 75.6, 75.5, 73.7 (CH_2 Bn), 71.6 (C-4), 70.3 (C-6); HRMS (ESI) M/Z: $[M + NH_4]^+$ Calcd for $C_{33}H_{38}NO_5S^+$ 560.2465; Found 560.2466.

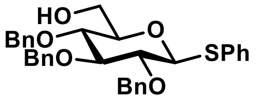

Phenyl 2,3,4-tri-*O*-benzyl-1-thio-β-D-glucopyranoside (S9). The title compound was prepared from **S6** (0.541 g, 1 mmol) according to literature procedure,[14] yielding the title compound (485 mg, 0.89 mmol, 89%) as a colorless oil. TLC: R_f 0.74, (pentane:EtOAc, 85:15, v:v); ^1H NMR (500 MHz, $CDCl_3$, HH-COSY, HSQC) δ 7.53 – 7.20 (m, 21H, CH_{arom}), 4.93 – 4.81 (m, 4H, 4x C*H*H Bn), 4.75 (d, J = 10.3 Hz, 1H, C*H*H Bn), 4.72 (d, J = 9.8 Hz, 1H, H-1), 4.65 (d, J = 11.0 Hz, 1H, C*H*H Bn), 3.86 (dq, J = 12.0, 2.6 Hz, 1H, H-6), 3.76 – 3.64 (m, 2H, , H-3, H-6), 3.58 (t, J = 9.4 Hz, 1H, H-4), 3.48 (dd, J = 9.8, 8.7 Hz, 1H, H-2), 3.38 (ddd, J = 9.7, 4.8, 2.6 Hz, 1H, H-5), 2.17 (t, J = 6.5 Hz, 1H, OH); $^{13}C\{^1H\}$ NMR (101 MHz, $CDCl_3$, HSQC) δ 138.4, 138.0, 137.9, 133.6 ($C_{q\text{-}arom}$), 131.8, 129.1, 128.6, 128.5, 128.5, 128.3, 128.1, 128.0, 128.0, 127.8, 127.8, 127.7 (CH_{arom}), 87.6 (C-1), 86.6 (C-3), 81.1 (C-2), 79.4 (C-5), 77.6 (C-4), 75.8, 75.6, 75.1 (CH_2 Bn), 62.1 (C-6); HRMS (ESI) M/Z: $[M + NH_4]^+$ Calcd for $C_{33}H_{38}NO_5S^+$ 560.2465; Found 560.2465.

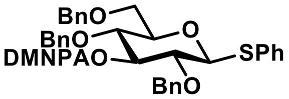

Phenyl 3-*O*-(2,2-dimethyl-2-(ortho-nitrophenyl)acetyl)-2,4,6-tri-*O*-benzyl-1-thio-β-D-glucopyranoside (3). The title compound was prepared from **S7** (0.81 g, 1.50 mmol) through general procedure I. Flash column chromatography (90:10 → 70:30, pentane:EtOAc v:v) yielded the title compound (1.08 g, 1.47 mmol, 98%) as a yellow oil. TLC: R_f 0.48, (pentane:EtOAc, 80:20, v:v); ^1H NMR (400 MHz, $CDCl_3$, HH-COSY, HSQC, HMBC, HMBC-Gated) δ 7.73 (dd, J = 7.9, 1.4 Hz, 1H, CH_{arom}), 7.56 – 7.42 (m, 4H, CH_{arom}), 7.40 – 7.17 (m, 19H, CH_{arom}), 5.42 (t, J = 7.7 Hz, 1H, H-3), 4.86 (d, J = 8.9 Hz, 1H, H-1), 4.82 (d, J = 10.6 Hz, 1H, C*H*H Bn), 4.65 – 4.55 (m, 2H, 2x C*H*H Bn), 4.50 (d, J = 3.2 Hz, 2H, 2x C*H*H Bn), 4.44 (d, J = 11.3 Hz, 1H, C*H*H Bn), 3.73 – 3.53 (m, 5H, H-2, H-4, H-5, H-6, H-6), 1.60 (s, 3H, CH_3 DMNPA), 1.58 (s, 3H, CH_3 DMNPA); $^{13}C\{^1H\}$ NMR (101 MHz, $CDCl_3$, HSQC, HMBC, HMBC-Gated) δ 174.3 (C=O DMNPA), 149.1, 138.3, 138.2, 138.0, 137.9, 134.0 ($C_{q\text{-}arom}$), 132.9, 131.9, 129.0, 128.8, 128.5, 128.4, 128.3, 128.0, 127.9, 127.8, 127.7, 127.7, 127.7, 127.6, 127.6, 125.5 (CH_{arom}), 86.7 (C-1), 78.9 (C-2), 78.5 (C-4), 76.0 (C-3), 75.8 (C-5), 73.5, 73.1, 72.4 (CH_2 Bn), 69.2 (C-6), 46.9 (C_q DMNPA), 27.0, 26.9 (CH_3 DMNPA); ^{13}C-GATED NMR (101 MHz, $CDCl_3$) δ 86.7 ($J_{H1\text{-}C1}$ = 158 Hz, β); HRMS (ESI) M/Z: $[M + NH_4]^+$ Calcd for $C_{43}H_{47}N_2O_8S^+$ 751.3048; Found 751.3043.

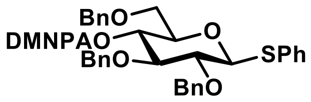

Phenyl 4-*O*-(2,2-dimethyl-2-(ortho-nitrophenyl)acetyl)-2,3,6-tri-*O*-benzyl-1-thio-β-D-glucopyranoside (5). The title compound was prepared from **S8** (0.47 g, 0.87 mmol) through general procedure I. Flash column chromatography (100:0 → 70:30, Pentane:EtOAc v:v) yielded the title compound (600 mg, 0,818 mmol, 94%) as a colorless oil TLC: R_f 0.51, (pentane:EtOAC, 80:20, v:v); ^{1}H NMR (500 MHz, $CDCl_3$, HH-COSY, HSQC, HMBC) δ 7.79 (dd, J = 8.1, 1.4 Hz, 1H, CH_{arom}), 7.59 – 7.56 (m, 2H, CH_{arom}), 7.53 (ddd, J = 7.9, 7.3, 1.5 Hz, 1H, CH_{arom}), 7.47 (dd, J = 8.1, 1.4 Hz, 1H, CH_{arom}), 7.40 – 7.16 (m, 19H, CH_{arom}), 4.97 (t, J = 9.1 Hz, 1H, H-4), 4.88 – 4.80 (m, 2H, 2x C*H*H Bn), 4.69 (d, J = 9.5 Hz, 1H, H-1), 4.63 (d, J = 11.8 Hz, 1H, C*H*H Bn), 4.59 – 4.50 (m, 4H, 3x C*H*H Bn), 3.81 – 3.76 (m, 1H, H-6), 3.71 – 3.56 (m, 5H, H-2, H-3, H-5, H-6), 1.55 – 1.53 (m, 6H, 2x CH_3 DMNPA); $^{13}C\{^1H\}$ NMR (101 MHz, $CDCl_3$, HSQC, HMBC) δ 174.6 (C=O DMNPA), 148.9 (C_{q-arom} DMNPA), 138.7, 138.4, 138.3, 137.8, 134.1 (C_{q-arom}), 133.2, 131.6, 129.1, 128.5, 128.4, 128.4, 128.4, 128.3, 128.1, 128.0, 127.9, 127.6, 127.5, 127.5, 126.9, 125.7 (CH_{arom}), 87.5 (C-1), 83.6 (C-5), 80.8 (C-2), 78.4 (C-3), 75.3, 74.5, 73.5 (CH_2 Bn), 71.5 (C-4), 70.0 (C-6), 46.9 (C_q DMNPA), 27.2, 27.1 (CH_3 DMNPA); HRMS (ESI) M/Z: $[M + Na]^+$ Calcd for $C_{43}H_{43}NNaO_8S^+$ 756.2602; Found 756.2593.

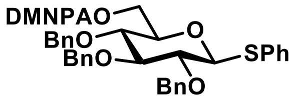

Phenyl 6-*O*-(2,2-dimethyl-2-(ortho-nitrophenyl)acetyl)-2,3,4-tri-*O*-benzyl-1-thio-β-D-glucopyranoside (7). The title compound was prepared from **S9** (0.38 g, 0.70 mmol) through general procedure I. Flash column chromatography (100:0 → 70:30, pentane:EtOAc v:v) yielded the title compound (0,51 g, 0,70 mmol, quant) as a colorless oil. TLC: R_f 0.39, (pentane:EtOAc, 80:20, v:v); ^{1}H NMR (500 MHz, $CDCl_3$, HH-COSY, HSQC, HMBC) δ 7.90 – 7.86 (m, 1H, CH_{arom}), 7.63 – 7.57 (m, 2H, CH_{arom}), 7.56 – 7.53 (m, 2H, CH_{arom}), 7.43 – 7.22 (m, 19H, CH_{arom}), 4.90 – 4.81 (m, 2H, 3x C*H*H Bn), 4.76 (d, J = 10.5 Hz, 1H, C*H*H Bn), 4.71 (d, J = 10.3 Hz, 1H, C*H*H Bn), 4.62 (d, J = 9.8 Hz, 1H, H-1), 4.58 (dd, J = 11.9, 2.0 Hz, 1H, H-6), 4.53 (d, J = 10.6 Hz, 1H, C*H*H Bn), 4.18 (dd, J = 11.9, 5.3 Hz, 1H, H-6), 3.70 (t, J = 8.9 Hz, 1H, H-3), 3.53 (ddd, J = 9.9, 5.3, 2.1 Hz, 1H, H-5), 3.46 – 3.37 (m, 2H, H-2, H-4), 1.70 (s, 3H, CH_3 DMNPA), 1.69 (s, 3H, CH_3 DMNPA); $^{13}C\{^1H\}$ NMR (126 MHz, $CDCl_3$, HSQC, HMBC) δ 175.1 (C=O DMNPA), 148.8, 139.2 (C_{q-arom} DMNPA), 138.3, 138.1, 137.7, 133.5 (C_{q-arom}), 133.3, 132.5, 129.0, 128.7, 128.6, 128.6, 128.3, 128.2, 128.1, 128.0, 128.0, 127.9, 127.8, 125.8 (CH_{arom}), 87.4 (C-1), 86.7 (C-3), 80.8 (C-2), 77.9 (C-4), 77.1 (C-5), 76.1, 75.5, 75.2 (CH_2 Bn), 63.6 (C-6), 46.7 (C_q DMNPA), 27.5, 27.4 (CH_3 DMNPA); HRMS (ESI) M/Z: $[M + NH_4]^+$ Calcd for $C_{43}H_{47}N_2O_8S^+$ 751.3048; Found 751.3045.

Preparation of donors 8 and 9

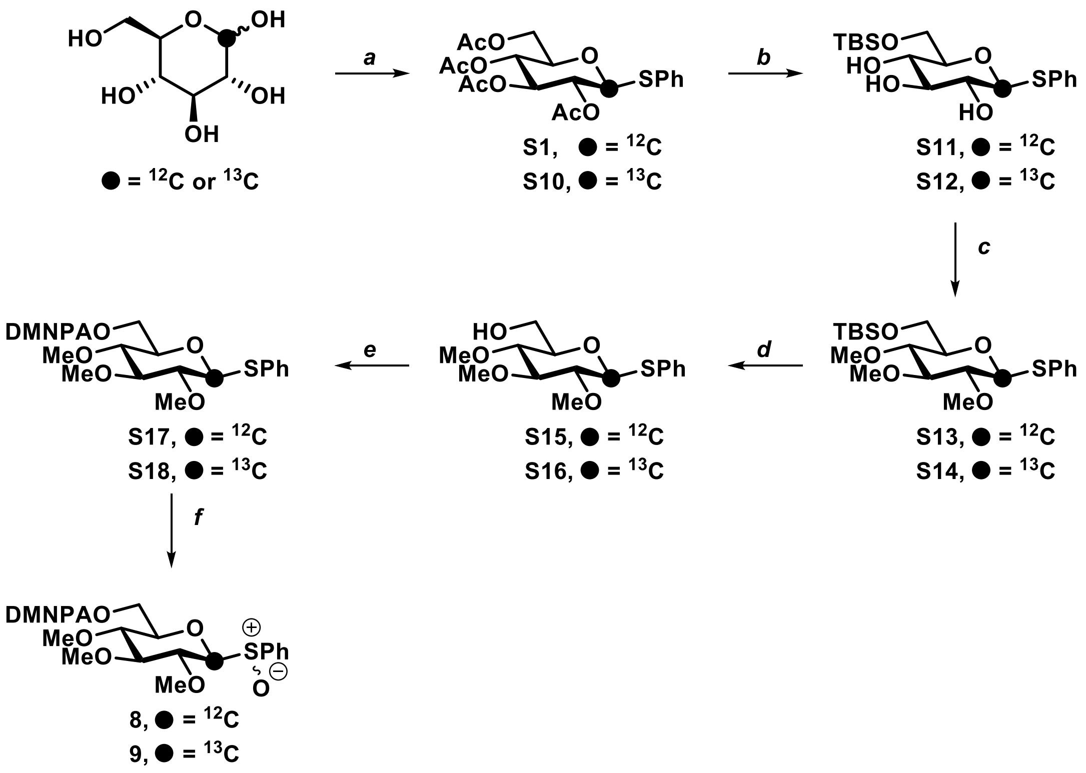

Supplementary scheme S2. Glucoside donor **8** and **9** synthesis. a) 1. Ac_2O, HCl_4O, HBr; 2. HSPh, NaH, DMF, **S1**: 59%, **S10:** 31%; b) 1. NaOMe, MeOH; 2. TBDMSCl, Et_3N, MeCN:DMF, **S11**: quant, **S12**: quant; c) MeI, NaH, DMF, 0 °C to rt, **S13**: 76%, **S14**: 80%; d) TBAF, THF, **S15**: 96%, **S16**: 88%; e) DMNPAA, TMSOTf, DCM, -40 °C, **S17**: 62%, **S18**: quant%; f) *m*-CPBA, DCM, -78 °C.

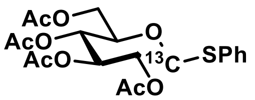

Phenyl 2,3,4,6-tetra-*O*-acetyl-1-^{13}C-thio-β-ᴅ-glucopyranoside (S10). The title compound was prepared from ᴅ-Glucose-1-^{13}C (1 g, 5.52 mmol) via general procedure II. Flash column chromatography (85:15 → 60:40, Pentane:EtOAc v:v) yielded the title compound (0.75 g, 1.70 mmol, 31%) as a white solid. TLC: R_f 0.64, (pentane:EtOAc, 60:30, v:v); ^{1}H NMR (500 MHz, $CDCl_3$, HH-COSY, HSQC, HMBC) δ 7.54 – 7.29 (m, 5H, CH_{arom}), 5.23 (td, J = 9.4, 0.9 Hz, 1H (C-3)), 5.05 (dd, J = 10.1, 9.4 Hz, 1H (C-4)), 4.98 (ddd, J = 10.1, 9.2, 5.0 Hz, 1H (C-2)), 4.71 (dd, J = 156.3, 10.1 Hz, 1H (C-1)), 4.27 – 4.15 (m, 2H, H-6, H-6), 3.73 (ddt, J = 10.2, 5.1, 2.6 Hz, 1H, H-5), 2.09 (s, 3H, OAc), 2.08 (s, 3H, OAc), 2.02 (s, 3H, OAc), 1.99 (s, 3H, OAc); $^{13}C\{^1H\}$ NMR (126 MHz, $CDCl_3$, HSQC, HMBC) δ 170.7, 170.3, 169.5, 169.3 (C=O Ac), 133.2 (CH_{arom}), 131.7 ($C_{q\text{-}arom}$), 129.1, 128.5 (CH_{arom}), 85.8 (C-1), 75.9 (C-5), 74.1 (d, *J* = 3.0 Hz, C-3), 70.1 (d, *J* = 47.4 Hz, C-2), 68.3 (C-4), 62.25 (d, *J* = 4.6 Hz, C-6), 20.8, 20.8, 20.7, 20.7 (OAc); HRMS (ESI) M/Z: $[M + NH_4]^+$ Calcd for $^{13}CC_{19}H_{28}NO_9S^+$ 459.1513; Found 459.1511.

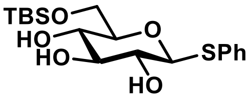

Phenyl 6-*O*-(*tert*-butyldimethylsilyl)-1-thio-β-D-glucopyranoside (S11). S1 (2.16 g, 4.90 mmol) was dissolved in MeOH (10 ml, 0.5M). NaOMe in MeOH (25 % Wt, 0.11 ml, 0.49 mmol, 0.1 eq) was added and the reaction was stirred for 3 hrs. The reaction mixture was neutralized to pH=7 using Amberlite H^+, filtered and concentrated in vacuo. The TBS installation protocol was adopted from Lv *et al* (2019).[15] The crude reaction mixture was suspended in a MeCN:DMF mixture (13.4 ml, 0.4M), and Et_3N (1.23 ml, 0.89 g, 8.83 mmol, 1.8 eq) was added dissolving all solids. Tert-Butyldimethylsilyl chloride (1.11 g, 7.36 mmol, 1.5 eq) was added and the reaction was stirred for 45 min. The reaction mixture was directly subjected to flash column chromatography (75:25 → 25:75, pentane:EtOAc v:v), yielding the title compound (1.95 g, 5.04 mmol, quant) as a colorless oil. TLC: R_f 0.37, (DCM:MeOH, 90:10, v:v); ^{1}H NMR (400 MHz, $CDCl_3$, HH-COSY, HSQC, HMBC) δ 7.61 – 7.11 (m, 5H, CH_{arom}), 4.54 (d, *J* = 9.7 Hz, 1H, H-1), 4.03 – 3.80 (m, 2H, H-6, H-6), 3.58 (t, *J* = 8.8 Hz, 1H, H-3), 3.50 (t, *J* = 9.1 Hz, 1H, H-4), 3.43 – 3.30 (m, 2H, H-2, H-5), 0.90 (s, 9H, C(CH_3)$_3$ TBS), 0.14 – 0.02 (m, 6H, 2x Si(CH_3)$_2$); ^{13}C{^{1}H} NMR (101 MHz, $CDCl_3$, HSQC, HMBC) δ 132.7 ($C_{q\text{-}arom}$), 132.3, 129.1, 129.0, 128.3, 127.9, 125.4 (CH_{arom}), 88.0 (C-1), 79.0 (C-2), 78.0 (C-3), 71.9 (C-5), 71.7 (C-4), 64.2 (C-6), 26.0 (3x C(CH_3)$_3$ TBS), 18.4 (C_q TBS), 5.2 (2x Si(CH_3)$_2$ TBS); HRMS (ESI) M/Z: $[M + NH_4]^+$ Calcd for $C_{18}H_{34}NO_5SSi^+$ 404.1922; Found 404.1923.

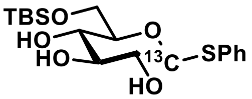

Phenyl 6-*O*-(*tert*-butyldimethylsilyl)-1-^{13}C-thio-β-D-glucopyranoside (S12). S10 (0.72 g, 1.63 mmol) was dissolved in MeOH (3.3 ml, 0.5M). NaOMe in MeOH (25 % Wt, 0.10 ml, 0.49 mmol, 0.3 eq) was added and the reaction was stirred for 3 hrs. The reaction mixture was neutralized to pH=7 using Amberlite H^+, filtered and concentrated in vacuo. The TBS installation protocol was adopted from Lv *et al* (2019).[15] The crude reaction mixture was suspended in a MeCN:DMF mixture (4.4 ml, 0.4M), and Et_3N (0.41 ml, 2.94 mmol, 1.8 eq) was added dissolving all solids. Tert-Butyldimethylsilyl chloride in toluene (50%, 0.85 ml, 2.45 mmol, 1.5 eq) was added and the reaction was stirred for 45 min. The reaction mixture was directly subjected to flash column chromatography (75:25 → 25:75, pentane:EtOAc v:v), yielding the title compound (0.95 g, 1.63 mmol, quant) as a white solid. TLC: R_f 0.31, (DCM:MeOH, 90:10, v:v); ^{1}H NMR (500 MHz, $CDCl_3$, HH-COSY, HSQC, HMBC) δ 7.59 – 7.50 (m, 2H, CH_{arom}), 7.31 – 7.19 (m, 3H, CH_{arom}), 4.63 (dd, *J* = 155.2, 9.7 Hz, 1H, H-2), 3.96 (dd, *J* = 11.1, 2.7 Hz, 1H, H-6), 3.79 (dd, *J* = 11.1, 5.4 Hz, 1H, H-6), 3.64 (t, *J* = 8.4 Hz, 1H, H-4), 3.49 – 3.33 (m, 3H, H-2, H-3, H-5), 0.90 (s, 9H, C(CH_3)$_3$ TBS), 0.08 (s, 3H, $SiCH_3$ TBS), 0.06 (s, 3H, $SiCH_3$ TBS); ^{13}C{^{1}H} NMR (126 MHz, $CDCl_3$, HSQC, HMBC) δ 133.5 ($C_{q\text{-}arom}$), 131.4, 131.4, 128.7, 127.1 (CH_{arom}), 87.5 (C-1), 80.0 (d, *J* = 2.0 Hz, C-3), 78.3 (d, *J* = 2.4 Hz, C-5), 72.1 (d, *J* = 44.6 Hz, C-2), 70.8 (C-4), 63.6 (d, *J* = 4.8 Hz, C-6), 18.2 (*C*(CH_3)$_3$ TBS), -5.4, -5.4 ($SiCH_3$ TBS); HRMS (ESI) M/Z: $[M + Na]^+$ Calcd for $^{13}CC_{17}H_{30}NaO_5SSi^+$ 410.1509; Found 410.1510.

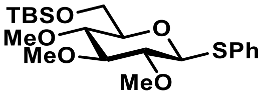

Phenyl 6-*O*-(*tert*-butyldimethylsilyl)-2,3,4-tri-*O*-methyl-1-thio-β-D-glucopyranoside (S13). S11 (1.89 g, 4.90 mmol) was dissolved in dry DMF (24.5 ml, 0.2M) and cooled to 0 °C. Under inert atmosphere, NaH (60 Wt % dispersion in mineral oil, 0.882 g, 22.1 mmol, 4.5 eq) was added portion wise and the reaction mixture was stirred for 10 minutes. methyl iodide (1.15 ml, 2.61 g, 18.4 mmol, 3.75 eq) was added and the reaction was stirred for 17 h while allowing to warm up to RT. Subsequently, the reaction was cooled to 0 °C and quenched with H_2O. The reaction mixture was diluted with H_2O and extracted with DCM. The organic layers were combined, washed with brine, dried over $MgSO_4$ and concentrated in vacuo. Flash column chromatography (100:0 → 80:20, pentane:EtOAc v:v) yielded the title compound (1.61 g, 3.74 mmol, 76%) as a colorless oil. TLC: R_f 0.64, (pentane:EtOAc, 90:10, v:v); ^{1}H NMR (500 MHz, $CDCl_3$, HH-COSY, HSQC, HMBC) δ 7.60 – 7.50 (m, 2H, CH_{arom}), 7.31 – 7.20 (m, 3H, CH_{arom}), 4.46 (d, J = 9.8 Hz, 1H, H-1), 3.85 (dd, J = 11.3, 1.6 Hz, 1H, H-6), 3.79 (dd, J = 11.3, 3.7 Hz, 1H, H-6), 3.65 (s, 3H, OCH_3), 3.57 (s, 3H, OCH_3), 3.55 (s, 3H, OCH_3), 3.26 – 3.15 (m, 3H, H-3, H-4, H-5), 3.08 – 2.95 (m, 1H, H-2), 0.92 (s, 9H, $C(CH_3)_3$ TBS), 0.10 (s, 3H, $SiCH_3$), 0.07 (s, 3H, $SiCH_3$); $^{13}C\{^1H\}$ NMR (126 MHz, $CDCl_3$, HSQC, HMBC) δ 133.8 ($C_{q\text{-}arom}$), 132.1, 128.9, 127.4 (CH_{arom}), 88.9 (C-3), 87.0 (C-1), 82.3 (C-2), 80.1 (C-5), 79.0 (C-4), 62.2 (C-6), 61.1, 60.8, 60.6 (OCH_3), 26.0 (3x $C(\mathit{C}H_3)_3$ TBS), 18.4 (C_q TBS), -5.0, -5.3 ($Si(\mathit{C}H_3)_2$ TBS); HRMS (ESI) M/Z: $[M + NH_4]^+$ Calcd for $C_{21}H_{40}NO_5SSi^+$ 446.2391; Found 446.2392.

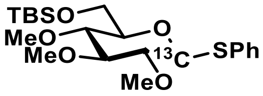

Phenyl 6-*O*-(*tert*-butyldimethylsilyl)-2,3,4-tri-*O*-methyl-1-^{13}C-thio-β-D-glucopyranoside (S14). S12 (0.63 g, 1.63 mmol) was dissolved in dry DMF (15.0 ml, 0.1M) and cooled to 0 °C. Under inert atmosphere, NaH (60 Wt % dispersion in mineral oil, 0.40 g, 10.1 mmol, 6.15 eq) was added portion wise and the reaction mixture was stirred for 10 minutes. methyl iodide (0.525 ml, 8.39 mmol, 5.15 eq) was added and the reaction was stirred for 17 h while allowing to warm up to RT. Subsequently, the reaction was cooled to 0 °C and quenched with H_2O. The reaction mixture was diluted with H_2O and extracted with DCM. The organic layers were combined, washed with brine, dried over $MgSO_4$ and concentrated in vacuo. Flash column chromatography (100:0 → 80:20, pentane:EtOAc v:v) yielded the title compound (0.56 g, 1.31 mmol, 80%) as a white oil. TLC: R_f 0.76, (pentane:EtOAc, 90:10, v:v); ^{1}H NMR (500 MHz, $CDCl_3$, HH-COSY, HSQC, HMBC) δ 7.61 – 7.19 (m, 5H, CH_{arom}), 4.46 (dd, *J* = 154.5, 9.8 Hz, 1H, H-1), 3.85 (dd, *J* = 11.3, 1.6 Hz, 1H, H-6), 3.79 (dd, *J* = 11.3, 3.4 Hz, 1H, H-6), 3.65 (s, 3H, OCH_3), 3.56 (s, 3H, OCH_3), 3.54 (s, 3H, OCH_3), 3.27 – 3.14 (m, 3H, H-3, H-4, H-5), 3.05 – 2.97 (m, 1H, H-2), 0.92 (s, 9H, $C(CH_3)_3$ TBS), 0.10 (s, 3H, $SiCH_3$), 0.07 (s, 3H, $SiCH_3$); $^{13}C\{^1H\}$ NMR (126 MHz, $CDCl_3$, HSQC, HMBC) δ 133.8 ($C_{q\text{-}arom}$), 132.1, 132.1, 128.8, 127.4 (CH_{arom}), 88.8 (d, *J* = 3.6 Hz, C-3), 87.0 (C-1), 82.3 (d, *J* = 44.0 Hz, C-2), 80.0 (d, *J* = 2.3 Hz, C-5), 78.9 (C-4), 62.2 (d, *J* = 4.8 Hz, C-6), 61.0, 60.8, 60.8, 60.5 (OCH_3), 18.4 ($\mathit{C}(CH_3)_3$ TBS), -5.1, -5.3 ($SiCH_3$ TBS); HRMS (ESI) M/Z: $[M + Na]^+$ Calcd for $^{13}CC_{20}H_{36}NaO_5S^+$ 452.1979; Found 452.1977.

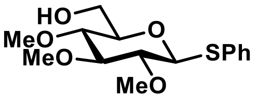

Phenyl 2,3,4-*O*-methyl-1-thio-β-ᴅ-glucopyranoside (S15). **S13** (1.52 g, 3.55 mmol) was co-evaporated with toluene, and dissolved in THF (10 ml, 0.4M). Tetra-n-butylammonium fluoride in THF (0.1M, 7.11 ml, 7.11 mmol, 2 eq) was added and the reaction was stirred for 1 h under intert atmosphere. Flash column chromatography (90:10 → 70:30, pentane:EtOAc v:v) yielded the title compound (1.07 g, 3.55 mmol, 96%) as a white solid. TLC: R_f 0.17, (pentane:EtOAc, 80:20, v:v); ^{1}H NMR (400 MHz, $CDCl_3$, HH-COSY, HSQC, HMBC) δ 7.52 – 7.46 (m, 2H, CH_{arom}), 7.34 – 7.23 (m, 3H, CH_{arom}), 4.55 (d, *J* = 9.9 Hz, 1H, H-2), 3.86 (ddd, *J* = 11.9, 6.1, 2.8 Hz, 1H, H-6), 3.70 (ddd, *J* = 12.0, 7.3, 5.0 Hz, 1H, H-6), 3.66 (s, 3H, OCH_3), 3.62 (s, 3H, OCH_3), 3.55 (s, 3H, OCH_3), 3.28 – 3.21 (m, 2H, H-3, H-5), 3.13 (dd, *J* = 9.8, 8.9 Hz, 1H, H-4), 3.03 (dd, *J* = 9.8, 8.7 Hz, 1H, H-2), 1.94 (t, *J* = 6.7 Hz, 1H, OH-6); $^{13}C\{^1H\}$ NMR (101 MHz, $CDCl_3$, HSQC, HMBC) δ 133.5 ($C_{q\text{-}arom}$), 132.0, 129.1, 127.8 (CH_{arom}), 88.6 (C-3), 87.2 (C-1), 82.8 (C-2), 79.6 (C-4), 79.2 (C-5), 62.3 (C-6), 61.1, 61.0, 60.7 (OCH_3); HRMS (ESI) M/Z: $[M + NH_4]^+$ Calcd for $C_{15}H_{26}NO_5S^+$ 332.1526; Found 332.1527.

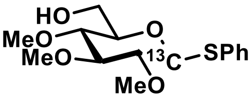

Phenyl 2,3,4-tri-*O*-methyl-1-^{13}C-thio-β-ᴅ-glucopyranoside (S16). **S14** (0.53 g, 1.23 mmol) was co-evaporated with toluene, and dissolved in THF (3,5 ml, 0.35M). Tetra-n-butylammonium fluoride in THF (0.1M, 2.47 ml, 2.47 mmol, 2 eq) was added and the reaction was stirred for 1 h under intert atmosphere. Flash column chromatography (80:20 → 60:40, pentane:EtOAc v:v) yielded the title compound (0.34 g, 1.08 mmol, 88%) as a white solid. TLC: R_f 0.27, (pentane:EtOAc, 70:30, v:v); ^{1}H NMR (500 MHz, $CDCl_3$, HH-COSY, HSQC, HMBC) δ 7.6 – 7.2 (m, 5H, CH_{arom}), 4.5 (dd, *J* = 156.3, 9.8 Hz, 1H, H-1), 3.9 – 3.6 (m, 8H, H-6, H-6, 2x OMe), 3.5 (s, 3H, OMe), 3.3 – 3.2 (m, 2H, H-3, H-5), 3.1 (dd, *J* = 9.8, 9.0 Hz, 1H, H-4), 3.0 (ddd, *J* = 9.8, 8.7, 5.3 Hz, 1H, H-2), 2.1 (s, 1H, OH-6); $^{13}C\{^1H\}$ NMR (126 MHz, $CDCl_3$, HSQC, HMBC) δ 133.5 ($C_{q\text{-}arom}$), 131.9, 129.1, 127.7 (CH_{arom}), 88.6 (d, *J* = 3.7 Hz, C-3), 87.1 (C-1), 82.7 (d, *J* = 44.1 Hz, C-2), 79.5 (C-4), 79.2 (d, *J* = 2.2 Hz, C-5), 62.2 (d, *J* = 4.4 Hz, C-6), 61.1 (CH_3), 60.1 (d, *J* = 1.7 Hz, CH_3), 60.7 (CH_3); HRMS (ESI) M/Z: $[M + Na]^+$ Calcd for $^{13}CC_{14}H_{22}NaO_5S^+$ 338.1114; Found 338.1111.

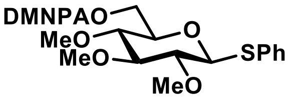

Phenyl 6-*O*-(2,2-dimethyl-2-(ortho-nitrophenyl)acetyl)-2,3,4-tri-*O*-methyl-1-thio-β-ᴅ-glucopyranoside (S17). The title compound was prepared from **S15** (0.15 g, 0.48 mmol) through general procedure I. Flash column chromatography (90:10 → 70:30, pentane:EtOAc v:v) yielded the title compound (0,15 g, 0,30 mmol, 62%) as a colorless oil. TLC: R_f 0.55, (pentane:EtOAc, 70:30, v:v); ^{1}H NMR (400 MHz, $CDCl_3$, HH-COSY, HSQC, HMBC) δ 8.06 (dd, *J* = 8.0, 1.3 Hz, 1H, CH_{arom}), 7.80 – 7.69 (m, 2H, CH_{arom}), 7.69 – 7.60 (m, 2H, CH_{arom}), 7.56 (ddd, *J* = 8.1, 6.7, 2.0 Hz, 1H, CH_{arom}), 7.43 – 7.34 (m, 3H, CH_{arom}), 4.67 – 4.55 (m, 2H, H-1, H-6), 4.27 (dd, *J* = 11.8, 5.8 Hz, 1H), 3.78 (s, 3H, OCH_3), 3.73 (s, 3H, OCH_3), 3.62 (s, 3H, OCH_3), 3.53 (ddd, *J* = 9.9, 5.9, 2.1 Hz, 1H, H-5), 3.36 (t, *J* = 8.8 Hz, 1H, H-3), 3.15 – 3.05 (m, 2H, H-2, H-4), 1.85 – 1.79 (m, 6H, 2x CH_3 DMNPA); $^{13}C\{^1H\}$ NMR (101 MHz, $CDCl_3$, HSQC, HMBC) δ 175.2 (C=O DMNPA), 148.8, 139.3, 133.4 ($C_{q\text{-}arom}$), 133.3, 132.4, 128.9, 128.2, 127.9, 127.7, 125.8 (CH_{arom}), 88.6 (C-3), 87.0 (C-1), 82.4 (C-2), 79.8 (C-4), 76.9 (C-5), 63.9 (C-6), 61.1, 60.9, 60.6 (OCH_3), 46.7 (C_q DMNPA), 27.5, 27.4 (CH_3 DMNPA); HRMS (ESI) M/Z: $[M + NH_4]^+$ Calcd for $C_{25}H_{35}N_2O_8S^+$ 523.2109; Found 523.2110.

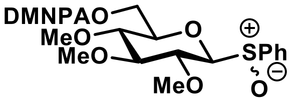

Phenyl 6-*O*-(2,2-dimethyl-2-(ortho-nitrophenyl)acetyl)-2,3,4-tri-*O*-methyl-1-sulphoxide-β-D-glucopyranoside (8). A solution of **S17** (15 mg, 0.030 mmol) in DCM (0.6 mL) was cooled to -78°C under inert atmosphere and then *m*-CPBA (8.0 mg, 0.033 mmol) was added. The reaction was stirred for three hours, diluted with DCM (15 mL) and washed with 10% aq. $Na_2S_2O_3$ solution, sat. aq. $NaHCO_3$ and brine. The organic layer was dried ($MgSO_4$), filtered, concentrated *in vacuo*. The crude product (15 mg) was used directly for IRMPD experiments. HRMS (ESI) M/Z: $[M + Na]^+$ Calcd for $C_{25}H_{31}NNaO_9S^+$, 544.1617; Found 544.1606.

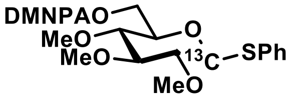

Phenyl 6-*O*-(2,2-dimethyl-2-(ortho-nitrophenyl)acetyl)-2,3,4-tri-*O*-methyl-1-^{13}C-thio-β-D-glucopyranoside (S18). The title compound was prepared from **S16** (0.075 g, 0.239 mmol) through general procedure I. Flash column chromatography (90:10 → 70:30, pentane:EtOAc v:v) yielded the title compound (0.134 g, 0.27 mmol, quant) as a colorless oil. TLC: R_f 0.48, (pentane:EtOAc, 70:30, v:v); ^{1}H NMR (500 MHz, $CDCl_3$, HH-COSY, HSQC, HMBC) δ 7.92 (dd, *J* = 8.1, 1.4 Hz, 1H, CH_{arom}), 7.65 – 7.56 (m, 2H, CH_{arom}), 7.55 – 7.47 (m, 2H, CH_{arom}), 7.41 (ddd, *J* = 8.1, 6.9, 1.8 Hz, 1H, CH_{arom}), 7.29 – 7.21 (m, 3H, CH_{arom}), 4.48 (dd, *J* = 11.8, 2.1 Hz, 1H, H-6), 4.45 (dd, *J* = 155.4, 9.8 Hz, 1H, H-1), 4.13 (dd, *J* = 11.8, 5.9 Hz, 1H, H-6), 3.63 (s, 3H, OCH_3), 3.58 (s, 3H, OCH_3), 3.47 (s, 3H, OCH_3), 3.39 (ddt, *J* = 10.3, 5.8, 2.3 Hz, 1H, H-5), 3.21 (td, *J* = 8.8, 1.0 Hz, 1H, H-3), 2.96 (ddd, *J* = 10.0, 8.8, 5.0 Hz, 2H, H-2), 1.69 – 1.65 (m, 6H, 2x CH_3 DMNPA); $^{13}C\{^1H\}$ NMR (126 MHz, $CDCl_3$, HSQC, HMBC) δ 175.2 (C=O DMNPA), 139.3, 133.4 ($C_{q\text{-}arom}$), 133.3, 132.4, 132.4, 128.9, 128.2, 127.9, 127.7, 125.8 (CH_{arom}), 88.6 (d, *J* = 3.7 Hz, C-3), 87.0 (C-1), 82.4 (d, *J* = 44.7 Hz, C-2), 79.8 (C-4), 76.9 (C-5), 63.9 (d, *J* = 4.6 Hz, C-6), 61.1 (OCH_3), 60.9 (d, *J* = 1.7 Hz, OCH_3), 60.6 (OCH_3), 46.7 (C_q DMNPA), 27.5, 27.4 (CH_3 DMNPA); HRMS (ESI) M/Z: $[M + NH_4]^+$ Calcd for $^{13}CC_{24}H_{35}N_2O_8S^+$ 524.2142; Found 524.2141.

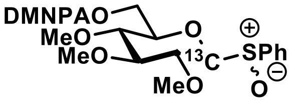

Phenyl 6-*O*-(2,2-dimethyl-2-(ortho-nitrophenyl)acetyl)-2,3,4-tri-*O*-methyl-1-^{13}C-sulphoxide-β-D-glucopyranoside (9). A solution of **S18** (15 mg, 0.030 mmol) in DCM (0.6 mL) was cooled to -78°C under inert atmosphere and then *m*-CPBA (8.0 mg, 0.033 mmol) was added. The reaction was stirred for three hours, diluted with DCM (15 mL) and washed with 10% aq. $Na_2S_2O_3$ solution, sat. aq. $NaHCO_3$ and brine. The organic layer was dried ($MgSO_4$), filtered, concentrated *in vacuo*. The crude product (15 mg) was used directly for IRMPD experiments. HRMS (ESI) M/Z: $[M + Na]^+$ Calcd for $C_{24}{}^{13}CH_{31}NNaO_9S^+$, 545.1651; Found 545.1645.

Preparation of DMNPAA reagent

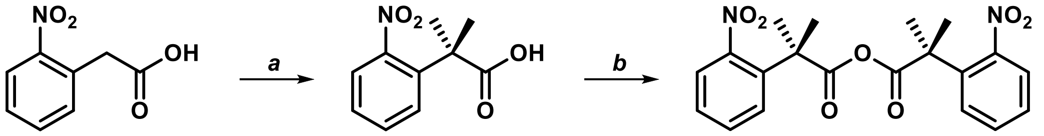

S19 S20

Supplementary scheme S3. DMNPAA reagent **S20** synthesis. a) 1. $SOCl_2$, MeOH, 0 °C to rt; 2. MeI, NaH, DMF, 0 °C to rt; 3. MeOH, NaOH,reflux, **S19**: 47%; b) DCC, CH_2Cl_2, **S20**: 74%.

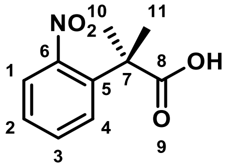

2-methyl-2-(2-nitrophenyl)propanoic acid (S19). The title compound was prepared according to literature procedure,[13] (12.7 g, 60.7 mmol, 47%) as a yellow solid. TLC: R_f 0.61, (DCM:MeOH, 95:5, v:v); ^{1}H NMR (400 MHz, $CDCl_3$, HH-COSY, HSQC) δ 7.97 (dt, J = 8.2, 0.9 Hz, 1H, CH_{arom}-1), 7.62 (dd, J = 4.7, 0.9 Hz, 2H, CH_{arom}-3,4), 7.46 – 7.38 (m, 1H, CH_{arom}-2), 1.70 (s, 6H, 2x CH_3); $^{13}C\{^1H\}$ NMR (101 MHz, $CDCl_3$, HSQC) δ 181.9 (C=O), 148.4 (C_q-6), 138.9 (C_q-5), 133.5 (C-3), 128.3 (C-4), 128.1 (C-2), 125.9 (C-1), 46.5 (C_q-7), 27.2 (C-10, C-11); HRMS (ESI) M/Z: [M + $NH_4]^+$ Calcd for $C_{10}H_{15}N_2O_4^+$ 227.1026; Found 227.1025.

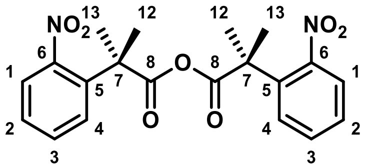

2-methyl-2-(2-nitrophenyl)propanoic anhydride (S20). The DMNPA reagent was prepared from **S19** (12.7 g, 60.7 mmol) according to literature procedure,[13] yielding the title compound (9.0 g, 30.4 mmol, 74%) as yellow crystals. TLC: R_f 0.39, (pentane:EtOAc, 70:30, v:v); ^{1}H NMR (400 MHz, $CDCl_3$, HH-COSY, HSQC, HMBC) δ 7.75 (dd, J = 8.1, 1.5 Hz, 1H, H-1), 7.55 (ddd, J = 8.0, 7.3, 1.5 Hz, 1H, H-3), 7.45 (dd, J = 8.1, 1.4 Hz, 1H, H-4), 7.34 (ddd, J = 8.1, 7.3, 1.4 Hz, 1H, H-2), 1.59 (s, 6H, 2x CH_3); $^{13}C\{^1H\}$ NMR (101 MHz, $CDCl_3$, HSQC, HMBC) δ 169.3 (C=O), 147.6 (C-6), 137.7 (C-5), 133.7 (C-3), 128.2 (C-4), 128.0 (C-2), 125.8 (C-1), 47.4 (C-7), 26.2 (C-13, C-12); HRMS (ESI) M/Z: [M + $NH_4]^+$ Calcd for $C_{20}H_{24}N_3O_7^+$ 418.1609; Found 418.1606.

Model glycosylation reactions

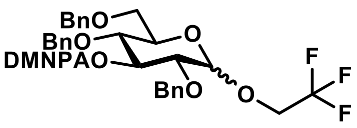

2,2,2-Trifluoroethyl 3-*O*-(2,2-dimethyl-2-(ortho-nitrophenyl)acetyl)-2,4,6-tri-*O*-benzyl-D-glucopyranoside (S21). The title compound was prepared according to general procedure VI, using **3** as the donor and 2,2,2-trifluoroethanol as the acceptor. Flash column chromatography (100:0 → 85:15, pentane:EtOAc v:v) yielded the title compound (52 mg, 0.066 mmol, 66%, α:β; 63:37) as a colorless oil. TLC: R_f 0.38, (85:15, pentane:EtOAc, v:v); ^{1}H NMR (850 MHz, $CDCl_3$, HH-COSY, HSQC, HMBC, HMBC-Gated) δ 7.83 – 7.12 (m, 50H), 5.53 (t, *J* = 9.5 Hz, 1H, H-3, α), 5.25 (dd, *J* = 8.1, 6.1 Hz, 1H, H-3 β), 4.84 (d, *J* = 11.4 Hz, 1H, C*H*H Bn βz), 4.70 (dd, *J* = 11.9, 4.8 Hz, 2H), 4.68 (d, *J* = 5.7 Hz, 1H, H-1 β), 4.62 (d, *J* = 3.6 Hz, 1H, H-1 α), 4.61 (d, *J* = 11.0 Hz, 2H), 4.54 – 4.49 (m, 2H), 4.48 (d, *J* = 12.1 Hz, 1H), 4.42 (d, *J* = 12.1 Hz, 1H), 4.38 – 4.33 (m, 3H), 4.19 (dq, *J* = 12.0, 8.7 Hz, 1H, C*H*HCF_3 β), 3.85 (dd, *J* = 9.9, 8.1 Hz, 1H, H-4 β), 3.83 – 3.78 (m, 2H), 3.75 (dq, *J* = 12.4, 8.7 Hz, 1H, CH*H*CF_3 α), 3.71 (ddd, *J* = 9.9, 3.8, 2.4 Hz, 1H, CH*H*CF_3 β), 3.70 – 3.65 (m, 2H), 3.65 – 3.60 (m, 2H), 3.57 – 3.53 (m, 2H), 3.50 (dd, *J* = 9.8, 3.6 Hz, 1H, H-2 α), 1.65 (s, 3H, CH_3 DMNPA α), 1.63 (s, 2H, CH_3 DMNPA β), 1.62 (s, 3H, CH_3 DMNPA α), 1.58 (s, 2H, CH_3 DMNPA β); ^{13}C{^{1}H} NMR (214 MHz, $CDCl_3$, HSQC, HMBC, HMBC-Gated) δ 174.5 (C=O DMNPA β), 174.3 (C=O DMNPA α), 149.1, 149.0, 145.7, 138.3, 138.3, 138.2, 138.0, 138.0, 138.0, 137.9, 137.7, 132.8, 131.2, 129.4, 128.9, 128.5, 128.5, 128.5, 128.4, 128.3, 128.3, 128.1, 128.0, 128.0, 128.0, 128.0, 127.9, 127.8, 127.8, 127.8, 127.7, 127.6, 127.6, 127.5, 125.6, 125.4, 124.9, 123.8 (q, *J* = 277.9 Hz, CF_3), 123.6 (q, *J* = 278.5 Hz, CF_3), 101.4 (C-1 β), 97.5 (C-1 α), 78.7 (C-2 β), 78.2 (C-2 α), 76.1 (C-3 β), 75.6, 75.2 (C-4 β), 73.8 (C-3 α), 73.6, 73.5, 73.5, 73.2, 73.0, 70.5, 68.6 (C-6 β), 68.1 (C-6 α), 65.4, 65.2, 65.2, 65.0, 65.0, 64.9, 64.8, 64.7, 47.1 (C_q DMNPA α), 46.8 (C_q DMNPA β), 27.1 (CH_3 DMNPA α), 26.9 (CH_3 DMNPA β), 26.8 (CH_3 DMNPA α), 26.7 (CH_3 DMNPA β); ^{13}C-GATED NMR (214 MHz, $CDCl_3$) δ 101.4 ($J_{H1\text{-}C1}$ = 166 Hz, β) 97.5 ($J_{H1\text{-}C1}$ = 169 Hz, α); HRMS (ESI) M/Z: $[M + NH_4]^+$ Calcd for $C_{39}H_{44}F_3N_2O_9^+$ 741.2993; Found 741.2982.

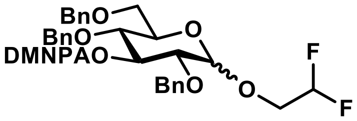

2,2-Difluoroethyl 3-*O*-(2,2-dimethyl-2-(ortho-nitrophenyl)acetyl)-2,4,6-tri-*O*-benzyl-D-glucopyranoside (S22). The title compound was prepared according to general procedure VI, using **3** as the donor and 2,2-difluoroethanol as the acceptor. Flash column chromatography (100:0 → 80:20, pentane:EtOAc v:v) yielded the title compound (56 mg, 0.079 mmol, 79%, α:β; 51:49) as a colorless oil. TLC: R_f 0.35, (85:15, pentane:EtOAc, v:v); ^{1}H NMR (400 MHz, $CDCl_3$, HH-COSY, HSQC, HMBC, HMBC-Gated) δ 7.81 – 7.09 (m, 55H), 5.88 (tt, *J* = 55.6, 4.3 Hz, 1H, CHF_2 β), 5.82 (tdd, *J* = 55.5, 5.1, 3.2 Hz, 1H, CHF_2 α), 5.52 (t, *J* = 9.6 Hz, 1H, H-3 α), 5.27 (dd, *J* = 8.3, 7.2 Hz, 1H, H-3 β), 4.83 (d, *J* = 11.6 Hz, 1H), 4.71 (d, *J* = 12.4 Hz, 1H), 4.66 (d, *J* = 11.7 Hz, 1H), 4.63 – 4.55 (m, 4H, H-1 α, H-1 β, C*H*H Bn, C*H*H Bn), 4.55 – 4.45 (m, 3H), 4.42 (d, *J* = 12.1 Hz, 1H), 4.40 – 4.30 (m, 3H), 4.02 – 3.88 (m, 1H, C*H*HCHF_2), 3.83 (ddd, *J* = 10.1, 3.5, 2.1 Hz, 1H, H-5 β), 3.76 (dd, *J* = 9.6, 8.3 Hz, 1H, H-4 β), 3.72 – 3.44 (m, 11H), 1.65 (s, 3H), 1.63 – 1.61 (m, 6H), 1.58 (s, 3H); ^{13}C{^{1}H} NMR (101 MHz, $CDCl_3$, HSQC, HMBC, HMBC-Gated) δ 174.4 (C=O), 174.3 (C=O), 149.2, 149.0, 145.7, 138.3, 138.3, 138.3, 138.0, 137.9, 137.7, 132.9, 132.7, 131.2, 129.4, 128.9, 128.7, 128.5, 128.5, 128.4, 128.3, 128.3, 128.1, 128.0, 128.0, 127.9, 127.9, 127.8, 127.8, 127.7, 127.6, 127.6, 127.5, 127.4, 125.5, 125.4, 124.9, 114.2 (t, *J* = 240.9 Hz, CHF_2 α), 114.1 (t, *J* = 241.2 Hz CHF_2 β), 102.5 (C-1 β), 97.5 (C-1 α), 79.3, 78.3, 76.1 (C-3 β), 75.7 (C-4 β), 75.5, 74.1 (C-3 α), 73.9, 70.2 (C-5 β), 68.7, 68.4, 68.3, 68.1, 67.8, 67.6, 67.3, 67.0, 47.1 (C_q DMNPA), 46.9 (C_q DMNPA), 27.1 (CH_3 DMNPA), 27.0 (CH_3 DMNPA), 26.8 (CH_3 DMNPA), 26.8 (CH_3 DMNPA); ^{13}C-GATED NMR (101 MHz, $CDCl_3$) δ 102.5 ($J_{H1\text{-}C1}$ = 164 Hz, β), 97.5 ($J_{H1\text{-}C1}$ = 170 Hz, α); HRMS (ESI) M/Z: $[M + NH_4]^+$ Calcd for $C_{39}H_{45}F_2N_2O_9^+$ 723.3088; Found 723.3084.

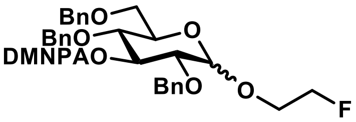

f2-Fluoroethyl 3-*O*-(2,2-dimethyl-2-(ortho-nitrophenyl)acetyl)-2,4,6-tri-*O*-benzyl-D-glucoyranoside (S23). The title compound was prepared according to general procedure VI, using **3** as the donor and 2-Fluoroethanol as the acceptor. Flash column chromatography (100:0 → 80:20, pentane:EtOAc v:v) yielded the title compound (66 mg, 0.096 mmol, 96%, α:β; 41:59) as a colorless oil. TLC: R_f 0.30, (85:15, pentane:EtOAc, v:v); ^{1}H NMR (400 MHz, $CDCl_3$, HH-COSY, HSQC, HMBC, HMBC-Gated) δ 7.78 – 7.12 (m, 51H), 5.57 (t, *J* = 9.5 Hz, 1H, H-3 α), 5.30 (t, *J* = 8.3 Hz, 1H, H-3 β), 4.92 (d, *J* = 11.5 Hz, 1H), 4.69 (d, *J* = 12.4 Hz, 1H), 4.65 (d, *J* = 3.5 Hz, 1H, H-1 α), 4.64 – 4.58 (m, 3H), 4.56 (d, *J* = 7.2 Hz, 1H, H-1 β), 4.55 – 4.31 (m, 9H), 4.05 (dddd, *J* = 32.3, 12.0, 4.8, 2.5 Hz, 1H, C*H*HCHF$_2$ β), 3.89 (ddd, *J* = 10.1, 3.4, 2.1 Hz, 1H, H-5 β), 3.81 – 3.41 (m, 11H), 1.65 (s, 2H), 1.62 (d, *J* = 1.8 Hz, 6H), 1.57 (s, 3H); ^{13}C{^{1}H} NMR (101 MHz, $CDCl_3$, HSQC, HMBC, HMBC-Gated) δ 174.3 (C=O β), 174.3 (C=O α), 149.2, 149.1, 145.6, 138.5, 138.3, 138.3, 138.3, 138.1, 138.0, 137.8, 132.8, 132.6, 131.1, 129.4, 128.9, 128.7, 128.5, 128.5, 128.4, 128.4, 128.4, 128.3, 128.3, 128.2, 128.1, 128.0, 127.9, 127.9, 127.8, 127.8, 127.8, 127.7, 127.7, 127.6, 127.6, 127.6, 127.5, 127.4, 127.3, 125.4, 125.3, 124.8, 102.7 (C-1 β), 97.0 (C-1 α), 82.61 (d, *J* = 169.8 Hz, CHF$_2$ β), 82.52 (d, *J* = 169.9 Hz, CHF$_2$ α), 79.6, 78.3, 76.1 (C-3 β), 75.9, 75.7, 74.2, 74.1 (C-3 α), 73.6, 73.5, 73.5, 73.2, 72.9, 69.7 (C-5 β), 68.8, 68.5, 68.4, 68.3, 67.2, 67.0, 47.1 (C_q DMNPA α), 46.9 (C_q DMNPA β), 27.0 (CH_3 DMNPA α), 26.9 (CH_3 DMNPA β), 26.8 (CH_3 DMNPA α), 26.7 (CH_3 DMNPA β); ^{13}C-GATED NMR (101 MHz, $CDCl_3$) δ 102.7 ($J_{H1\text{-}C1}$ = 161 Hz, β), 97.0 ($J_{H1\text{-}C1}$ = 169 Hz, α); HRMS (ESI) M/Z: $[M + NH_4]^+$ Calcd for $C_{39}H_{46}FN_2O_9^+$ 705.3182; Found 705.3182.

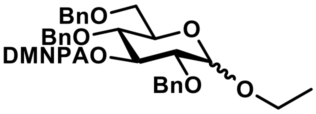

Ethyl 3-*O*-(2,2-dimethyl-2-(ortho-nitrophenyl)acetyl)-2,4,6-tri-*O*-benzyl-D-glucopyranoside (S24). The title compound was prepared according to general procedure VI, using **3** as the donor and ethanol as the acceptor. Flash column chromatography (100:0 → 80:20, pentane:EtOAc v:v) yielded the title compound (69 mg, quant, α:β; 43:57) as a colorless oil. TLC: R_f 0.35, (85:15, pentane:EtOAc, v:v); ^{1}H NMR (400 MHz, $CDCl_3$, HH-COSY, HSQC, HMBC, HMBC-Gated) δ 7.77 – 7.12 (m, 42H), 5.57 (t, *J* = 9.5 Hz, 1H, H-3 α), 5.31 (t, *J* = 8.6 Hz, 1H, H-3 β), 4.95 (d, *J* = 11.6 Hz, 1H), 4.68 (d, *J* = 12.5 Hz, 1H), 4.63 – 4.58 (m, 1H), 4.59 (d, *J* = 3.3 Hz, 1H, H-1 α), 4.57 – 4.51 (m, 3H), 4.50 (d, *J* = 7.5 Hz, 1H, H-1 β), 4.44 (d, *J* = 12.1 Hz, 1H), 4.40 – 4.31 (m, 3H), 3.95 (dq, *J* = 9.5, 7.1 Hz, 1H, C*H*HCH$_3$ β), 3.84 (ddd, *J* = 10.1, 3.5, 2.1 Hz, 1H, H-5 β), 3.71 – 3.48 (m, 8H), 3.45 (dd, *J* = 9.9, 3.5 Hz, 1H, H-2 α), 3.39 (dd, *J* = 8.6, 7.3 Hz, 1H, H-2 β), 3.30 (dq, *J* = 9.8, 7.1 Hz, 1H, C*H*HCH$_3$ α), 1.65 (s, 3H, CH_3 DMNPA α), 1.62 (s, 3H, CH_3 DMNPA α), 1.61 (s, 3H, CH_3 DMNPA β), 1.56 (s, 3H, CH_3 DMNPA β) 1.22 – 1.14 (m, 6H, CH_2CH_3 α, CH_2CH_3 β); ^{13}C{^{1}H} NMR (101 MHz, $CDCl_3$, HSQC, HMBC, HMBC-Gated) δ 174.3 (C=O α), 174.2 (C=O β), 149.2, 149.1, 145.6, 138.7, 138.4, 138.3, 138.2, 138.2, 138.1, 138.0, 137.8, 102.9 (C-1 β), 96.4 (C-1 α), 79.9 (C-2 β), 78.4 (C-2 α), 76.2 (C-3 β), 76.1, 76.0, 74.4 (C-3 α), 74.4, 73.6, 73.5, 73.4, 73.3, 73.3, 72.8, 69.5 (C-5 β), 68.9, 68.5, 65.4 (CH_2 Et β), 63.5 (CH_2 Et α), 47.2 (C_q DMNPA β), 47.0 (C_q DMNPA α), 27.0, 26.9, 26.7, 15.3 (CH_3 Et β), 15.0 (CH_3 Et β); ^{13}C-GATED NMR (101 MHz, $CDCl_3$) δ 102.9 ($J_{H1\text{-}C1}$ = 160 Hz, β), 96.4 ($J_{H1\text{-}C1}$ = 169 Hz, α); HRMS (ESI) M/Z: $[M + NH_4]^+$ Calcd for $C_{39}H_{47}N_2O_9^+$ 687.3276; Found 687.3277.

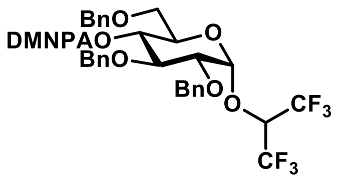

1,1,1,3,3,3-Hexafluoro-2-propyl 4-*O*-(2,2-dimethyl-2-(ortho-nitrophenyl)acetyl)-2,3,6-tri-*O*-benzyl-α-D-glucopyranoside (S25). The title compound was prepared according to general procedure VI, using **5** as the donor and 1,1,1,3,3,3-hexafluoro-2-propanol as the acceptor. Flash column chromatography (100:0 → 85:15, pentane:EtOAc v:v) yielded the title compound (31 mg, 39 mmol, 39%, α:β; >98:2) as a colorless oil. TLC: R_f 0.32, (pentane:EtOAc, 85:15, v:v); ^{1}H NMR (850 MHz, $CDCl_3$, HH-COSY, HSQC, HMBC, HMBC-Gated) δ 7.86 – 7.16 (m, 39H), 5.19 (d, *J* = 3.7 Hz, 1H, H-1), 5.15 (dd, *J* = 10.2, 8.9 Hz, 1H, H-4), 4.95 (d, *J* = 11.4 Hz, 1H, C*H*H Bn), 4.65 (d, *J* = 11.8 Hz, 1H, C*H*H Bn), 4.62 (d, *J* = 11.4 Hz, 1H, C*H*H Bn), 4.59 – 4.54 (m, 2H, CH*H* Bn, CH*H* Bn), 4.52 (d, *J* = 11.8 Hz, 1H, CH*H* Bn), 3.94 (ddd, *J* = 10.3, 4.9, 2.2 Hz, 1H, H-5), 3.91 (t, *J* = 9.3 Hz, 1H, H-3), 3.71 (dd, *J* = 9.6, 3.6 Hz, 1H, H-2), 3.69 – 3.61 (m, 2H, H-6, H-6), 1.57 (s, 3H, CH_3 DMNPA), 1.53 (s, 3H, CH_3 DMNPA); $^{13}C\{^{1}H\}$ NMR (214 MHz, $CDCl_3$, HSQC, HMBC, HMBC-Gated) δ 174.3 (C=O), 148.8, 145.7, 138.7, 138.4, 138.3, 137.3, 133.3, 131.2, 129.4, 128.6, 128.4, 128.3, 128.3, 128.2, 128.1, 128.0, 128.0, 127.6, 127.3, 126.9, 125.7, 124.9, 98.5 (C-1), 78.7 (C-2), 77.8 (C-3), 74.4 (CH_2 Bn), 73.6 (CH_2 Bn), 73.3 (CH_2 Bn), 72.4 (p, *J* = 32.8 Hz, $CH(CF_3)_2$), 70.9 (C-5), 70.2 (C-4), 68.3 (C-6), 47.0 (C_q DMNPA), 27.4 (CH_3 DMNPA), 27.2 (CH_3 DMNPA); ^{13}C-GATED NMR (214 MHz, $CDCl_3$) δ 98.5 ($J_{H1\text{-}C1}$ = 172 Hz, β); HRMS (ESI) M/Z: $[M + NH_4]^+$ Calcd for $C_{40}H_{43}F_6N_2O_9^+$ 809.2867; Found 809.2863.

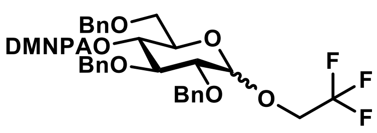

2,2,2-Trifluoroethyl 4-*O*-(2,2-dimethyl-2-(ortho-nitrophenyl)acetyl)-2,3,6-tri-*O*-benzyl-D-glucopyranoside (S26). The title compound was prepared according to general procedure VI, using **5** as the donor and 2,2,2-trifluoroethanol as the acceptor. Flash column chromatography (100:0 → 85:15, pentane:EtOAc v:v) yielded the title compound (72 mg, 0.066 mmol, 66%, α:β; 80:20) as a colorless oil. TLC: R_f 0.45, (pentane:EtOAc, 80:20, v:v); ^{1}H NMR (500 MHz, $CDCl_3$, HH-COSY, HSQC, HMBC, HMBC-Gated) δ 7.86 – 7.18 (m, 36H), 5.05 (dd, *J* = 10.2, 9.2 Hz, 1H, H-4 α), 5.00 – 4.91 (m, 1H), 4.88 (d, *J* = 2.9 Hz, 0.25H), 4.86 (d, *J* = 3.5 Hz, 1H, H-1 α), 4.69 – 4.61 (m, 2H), 4.59 – 4.45 (m, 4H, H-1 β), 4.20 (dq, *J* = 12.5, 8.8 Hz, 0.25H, C*H*HCF₃), 4.01 – 3.75 (m, 4H), 3.73 – 3.53 (m, 4H), 1.57 (s, 3H, CH_3 DMNPA α), 1.54 (s, 0.75H, CH_3 DMNPA β), 1.52 (s, 3H, CH_3 DMNPA α), 1.52 (s, 0.75H, CH_3 DMNPA β); $^{13}C\{^{1}H\}$ NMR (126 MHz, $CDCl_3$, HSQC, HMBC, HMBC-Gated) δ 174.5 (C=O β), 174.4 (C=O α), 148.8, 145.9, 138.7, 138.6, 138.5, 138.4, 138.4, 138.3, 137.8, 133.3, 131.2, 129.4, 128.6, 128.5, 128.5, 128.4, 128.4, 128.3, 128.3, 128.2, 128.1, 128.0, 128.0, 127.9, 127.6, 127.3, 127.0, 124.9, 123.9 (q, *J* = 278.8 Hz, CF_3 β), 122.8, 120.6, 103.3 (C-1 β), 97.1 (C-1 α), 81.2 (C-3 β), 81.1 (C-2 β), 79.6 (C-2 α), 78.4 (C-3 αf), 74.7, 74.4, 74.0, 73.6, 73.1, 71.1 (C-4 β), 70.8 (C-4 α), 70.0, 69.6 (C-6 β), 68.8 (C-6 α), 66.1, 65.8, 64.43 (q, *J* = 34.8 Hz, CH_2CF_3), 47.0 (C_q DMNPA α), 46.9 (C_q DMNPA β), 27.3 (CH_3 DMNPA α), 27.2 (CH_3 DMNPA β), 27.1 (CH_3 DMNPA α), 27.1 (CH_3 DMNPA β); ^{13}C-GATED NMR (126 MHz, $CDCl_3$) δ 103.3 ($J_{H1\text{-}C1}$ = 161 Hz, β), 97.1 ($J_{H1\text{-}C1}$ = 172 Hz, α); HRMS (ESI) M/Z: $[M + NH_4]^+$ Calcd for $C_{39}H_{44}F_3N_2O_9^+$ 741.2993; Found 741.2994.

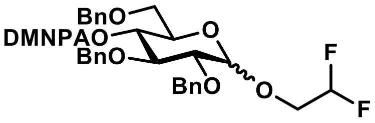

2,2-Difluoroethyl 4-*O*-(2,2-dimethyl-2-(ortho-nitrophenyl)acetyl)-2,3,6-tri-*O*-benzyl-D-glucopyranoside (S27). The title compound was prepared according to general procedure VI, using **5** as the donor and 2,2-difluoroethanol as the acceptor. Flash column chromatography (95:5 → 80:20, pentane:EtOAc v:v) yielded the title compound (54 mg, 0.077 mmol, 77%, α:β; 50:50) as a colorless oil. TLC: R_f 0.36, (pentane:EtOAc, 80:20, v:v); ^{1}H NMR (500 MHz, $CDCl_3$, HH-COSY, HSQC, HMBC, HMBC-Gated) δ 7.93 – 7.17 (m, 55H), 5.99 (tdd, *J* = 55.4, 4.9, 3.6 Hz, 1H, CHF_2 α), 5.95 (dddd, *J* = 56.1, 54.8, 5.4, 2.9 Hz, 1H, CHF_2 β), 5.03 (dd, *J* = 10.2, 9.2 Hz, 1H, H-4 α), 4.98 – 4.91 (m, 2H), 4.89 – 4.82 (m, 2H), 4.77 (d, *J* = 3.6 Hz, 1H, H-1 α), 4.69 – 4.47 (m, 9H), 4.45 (d, *J* = 7.5 Hz, 1H, H-1 β), 4.01 (dddd, *J* = 20.7, 11.8, 10.4, 2.9 Hz, 1H, C*H*HCHF$_2$ β), 3.90 (t, *J* = 9.4 Hz, 1H, H-3 α), 3.87 – 3.66 (m, 6H), 3.66 – 3.51 (m, 6H), 1.56 (s, 3H, CH_3 DMNPA), 1.54 (s, 3H, CH_3 DMNPA), 1.52 (s, 6H, CH_3 DMNPA, CH_3 DMNPA); $^{13}C\{^1H\}$ NMR (126 MHz, $CDCl_3$, HSQC, HMBC, HMBC-Gated) δ 174.5 (C=O α), 174.5 (C=O β), 148.8, 145.8, 138.6, 138.5, 138.4, 138.4, 138.3, 138.0, 137.8, 133.3, 131.2, 129.4, 128.6, 128.5, 128.5, 128.4, 128.3, 128.3, 128.3, 128.2, 128.2, 128.0, 128.0, 127.9, 127.9, 127.7, 127.6, 127.4, 127.3, 127.0, 127.0, 125.7, 124.9, 114.2 (dd, *J* = 242.2, 239.8 Hz, CHF_2 β), 114.1 (t, *J* = 241.2 Hz, CHF_2 α), 103.8 (C-1 β), 97.6 (C-1 α), 81.4 (C-2 α), 81.4 (C-2 β), 79.8, 78.6, 74.7, 74.4, 74.2, 73.9, 73.5, 73.3, 71.2 (C-4 β), 70.9 (C-4 α), 69.7, 69.6, 68.9, 68.9, 68.7, 68.7, 68.5, 67.7, 67.4, 67.2, 47.0 (C_q DMNPA), 46.9 (C_q DMNPA), 27.3 (CH_3 DMNPA), 27.2 (CH_3 DMNPA), 27.1 (CH_3 DMNPA, CH_3 DMNPA); ^{13}C-GATED NMR (126 MHz, $CDCl_3$) δ 103.8 (J_{H1-C1} = 160 Hz, β), 97.6 (J_{H1-C1} = 170 Hz, α); HRMS (ESI) M/Z: $[M + NH_4]^+$ Calcd for $C_{39}H_{45}F_2N_2O_9^+$ 723.3088; Found 723.3087.

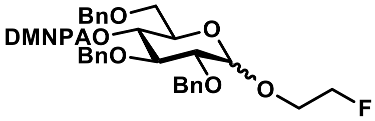

2-Fluoroethyl 4-*O*-(2,2-dimethyl-2-(ortho-nitrophenyl)acetyl)-2,4,6-tri-*O*-benzyl-D-glucopyranoside (S28). The title compound was prepared according to general procedure VI, using **5** as the donor and 2-Fluoroethanol as the acceptor. Flash column chromatography (90:10 → 75:25, pentane:EtOAc v:v) yielded the title compound (51 mg, 0.074 mmol, 75%, α:β; 36:64) as a colorless oil. TLC: R_f 0.25, (pentane:EtOAc, 80:20, v:v); ^{1}H NMR (500 MHz, $CDCl_3$, HH-COSY, HSQC, HMBC, HMBC-Gated) δ 7.85 – 7.20 (m, 38H), 5.06 (dd, *J* = 10.2, 9.2 Hz, 0.56H, H-4 α), 4.99 (d, *J* = 11.5 Hz, 1H), 4.98 – 4.90 (m, 2H), 4.88 (d, *J* = 11.4 Hz, 1H), 4.83 (d, *J* = 3.6 Hz, 0.56H, H-1 α), 4.73 – 4.48 (m, 8H), 4.48 (d, *J* = 7.5 Hz, 1H, H-1 β), 4.11 (dddd, *J* = 33.8, 12.2, 4.5, 2.4 Hz, 1H, CH_2F β), 3.95 (t, *J* = 9.4 Hz, 0.56H, H-3 α), 3.94 – 3.79 (m, 2H), 3.81 – 3.53 (m, 7H), 1.57 (s, 1.68H, CH_3 DMNPA α), 1.55 (s, 3H, CH_3 DMNPA β), 1.53 (s, 4.68H, CH_3 DMNPA α, CH_3 DMNPA β); $^{13}C\{^1H\}$ NMR (126 MHz, $CDCl_3$, HSQC, HMBC, HMBC-Gated) δ 174.5 (C=O β), 174.5 (C=O α), 148.8, 145.7, 138.8, 138.7, 138.5, 138.5, 138.4, 138.2, 138.0, 133.2, 131.2, 129.4, 128.6, 128.6, 128.5, 128.5, 128.5, 128.4, 128.4, 128.3, 128.3, 128.3, 128.2, 128.1, 128.1, 128.0, 128.0, 128.0, 127.9, 127.9, 127.8, 127.6, 127.5, 127.4, 127.2, 127.1, 126.9, 125.7, 124.9, 103.5 (C-1 β), 96.9 (C-1 α), 82.7 (d, *J* = 169.8 Hz, CH_2F β), 82.6 (d, *J* = 169.7 Hz, CH_2F α), 81.5 (C-3 β), 81.4 (C-2 β), 79.9 (C-2 α), 78.7 (C-3 α), 77.4, 77.2, 76.9, 74.6, 74.3, 74.2, 73.8, 73.5, 73.5, 73.1 (C-4 β), 71.3 (C-4 α), 71.1, 69.8, 69.3, 69.0, 68.9 (d, *J* = 19.9 Hz, CH_2CH_2F β), 67.2 (d, *J* = 20.1 Hz, CH_2CH_2F α), 47.0 (C_q DMNPA α), 46.9 (C_q DMNPA β), 27.3 (CH_3 DMNPA α), 27.2 (CH_3 DMNPA β), 27.1 (CH_3 DMNPA α), 27.1 (CH_3 DMNPA β); ^{13}C-GATED NMR (126 MHz, $CDCl_3$) δ 103.5 (J_{H1-C1} = 160 Hz, β), 96.9 (J_{H1-C1} = 171zz Hz, α); HRMS (ESI) M/Z: $[M + NH_4]^+$ Calcd for $C_{39}H_{46}FN_2O_9^+$ 705.3182; Found 705.3181.

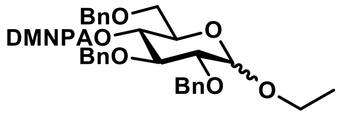

Ethyl 4-*O*-(2,2-dimethyl-2-(ortho-nitrophenyl)acetyl)-2,3,6-tri-*O*-benzyl-D-glucopyranoside (S29). The title compound was prepared according to general procedure VI, using **5** as the donor and ethanol as the acceptor. Flash column chromatography (95:5 → 80:20, pentane:EtOAc v:v) yielded the title compound (48 mg, 0.072 mmol, 72%, α:β; 26:74) as a colorless oil. TLC: R_f 0.45, (pentane:EtOAc, 80:20, v:v); ^{1}H NMR (500 MHz, $CDCl_3$, HH-COSY, HSQC, HMBC, HMBC-Gated) δ 7.84 – 7.17 (m, 35H), 5.04 (dd, *J* = 10.2, 9.1 Hz, 0.31H, H-4 α), 4.99 (d, *J* = 11.5 Hz, 0.31H, C*H*H Bn α), 4.95 – 4.88 (m, 2H), 4.85 (d, *J* = 11.4 Hz, 1H), 4.78 (d, *J* = 3.6 Hz, 0.31H, H-1 α), 4.69 – 4.50 (m, 5H), 4.47 (d, *J* = 11.4 Hz, 1H), 4.42 (d, *J* = 7.6 Hz, 1H, H-1 β), 4.01 (dq, *J* = 9.6, 7.1 Hz, 1H, C*H*HCH_3 β), 3.93 (t, *J* = 9.4 Hz, 0.31H, H-3 α), 3.85 (ddd, *J* = 10.2, 5.8, 2.1 Hz, 0.31H, H-5 α), 3.81 – 3.47 (m, 8H), 1.56 (s, 0.93H, CH_3 DMNPA α), 1.53 (s, 3H, CH_3 DMNPA β), 1.52 (s, 0.93H, CH_3 DMNPA α), 1.51 (s, 3H, CH_3 DMNPA β), 1.31 – 1.24 (m, 5H, CH_2CH_3 α, CH_2CH_3 β); $^{13}C\{^1H\}$ NMR (126 MHz, $CDCl_3$, HSQC, HMBC, HMBC-Gated) δ 174.5 (C=O β), 174.5 (C=O β), 148.9, 145.7, 138.9, 138.7, 138.6, 138.6, 138.5, 138.5, 138.4, 138.1, 133.1, 131.1, 129.4, 128.5, 128.5, 128.5, 128.4, 128.4, 128.3, 128.2, 128.2, 128.2, 128.0, 128.0, 127.9, 127.7, 127.5, 127.4, 127.3, 127.1, 126.9, 125.6, 124.9, 103.2 (C-1 β), 96.1 (C-1 α), 81.6, 81.6, 80.0 (C-2 α), 78.9 (C-3 α), 74.5, 74.3, 74.2, 73.7, 73.6, 73.5, 73.0, 71.5 (C-4 β), 71.4 (C-4 α), 69.9 (C-6 β), 69.1 (C-5 α), 69.1 (C-6 α), 65.7 (CH_2 Et β), 63.5 (CH_2 Et α), 47.0 (C_q DMNPA α), 46.9 (C_q DMNPA β), 27.3 (CH_3 DMNPA α), 27.2 (CH_3 DMNPA β), 27.1 (CH_3 DMNPA α), 27.1 (CH_3 DMNPA β), 15.4 (CH_3 Et α), 15.0 (CH_3 Et β); ^{13}C-GATED NMR (126 MHz, $CDCl_3$) δ 103.2 (J_{H1-C1} = 159 Hz, β), 96.1 (J_{H1-C1} = 167 Hz, α); HRMS (ESI) M/Z: $[M + NH_4]^+$ Calcd for $C_{39}H_{47}N_2O_9^+$ 687.3276; Found 687.3277.

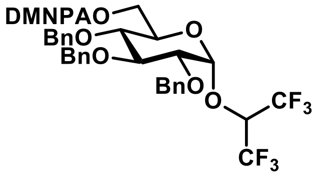

1,1,1,3,3,3-Hexafluoro-2-propyl 6-*O*-(2,2-dimethyl-2-(ortho-nitrophenyl)acetyl)-2,3,4-tri-*O*-benzyl-α-D-glucopyranoside (S30). The title compound was prepared according to general procedure VI, using **7** as the donor and 1,1,1,3,3,3-hexafluoro-2-propanol as the acceptor. Flash column chromatography (100:0 → 90:10, pentane:EtOAc v:v) yielded the title compound (27 mg, 0.034 mmol, 0.034, 34%, α:β; >98:2) as a colorless oil. TLC: R_f 0.48, (80:20, pentane:EtOAc, v:v); ^{1}H NMR (500 MHz, $CDCl_3$, HH-COSY, HSQC, HMBC, HMBC-Gated) δ 7.94 – 7.20 (m, 45H), 5.10 (d, *J* = 3.8 Hz, 1H, H-1 α), 4.94 (d, *J* = 10.7 Hz, 1H), 4.83 – 4.76 (m, 2H), 4.73 – 4.63 (m, 2H), 4.40 (dd, *J* = 11.9, 1.9 Hz, 1H, H-6), 4.32 (hept, *J* = 5.9 Hz, 1H, $CH(CF_3)_2$), 4.14 (dd, *J* = 12.0, 5.7 Hz, 1H, H-6), 3.98 – 3.85 (m, 2H, H-3, H-5), 3.53 (dd, *J* = 9.8, 3.8 Hz, 1H, H-2), 3.30 (dd, *J* = 10.2, 9.1 Hz, 1H, H-4), 1.68 (s, 3H, CH_3 DMNPA), 1.66 (s, 3H, CH_3 DMNPA); $^{13}C\{^1H\}$ NMR (126 MHz, $CDCl_3$, HSQC, HMBC, HMBC-Gated) δ 175.0 (C=O DMNPA), 148.4, 145.7, 139.4, 138.4, 137.6, 137.5, 133.6, 131.2, 129.4, 128.7, 128.6, 128.6, 128.2, 128.2, 128.2, 128.1, 128.1, 128.1, 128.0, 127.9, 125.7, 124.9, 98.6 (C-1), 81.0 (C-3), 79.0 (C-2), 77.5 (C-4), 76.0, 75.5, 73.4, 72.7 – 71.5 (m, $CH(CF_3)_2$), 70.4 (C-5), 63.5 (C-6), 46.7 (C_q DMNPA), 27.5 (CH_3 DMNPA), 27.5 (CH_3 DMNPA); ^{13}C-GATED NMR (126 MHz, $CDCl_3$) δ 98.6 (J_{H1-C1} = 171 Hz, α); HRMS (ESI) M/Z: $[M + NH_4]^+$ Calcd for $C_{40}H_{43}F_6N_2O_9^+$ 809.2867; Found 809.2866.

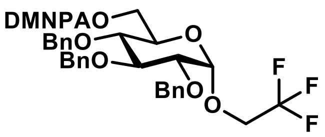

2,2,2-Trifluoroethyl 6-*O*-(2,2-dimethyl-2-(ortho-nitrophenyl)acetyl)-2,3,4-tri-*O*-benzyl-α-D-glucopyranoside (S31). The title compound was prepared according to general procedure VI, using **7** as the donor and 2,2,2-trifluoroethanol as the acceptor. Flash column chromatography (100:0 → 90:10, pentane:EtOAc v:v) yielded the title compound (46 mg, 0.064 mmol, 64%, α:β; >98:2) as a colorless oil. TLC: R_f 0.40, (80:20, pentane:EtOAc, v:v); ^{1}H NMR (500 MHz, $CDCl_3$, HH-COSY, HSQC, HMBC, HMBC-Gated) δ 7.93 – 7.22 (m, 27H), 4.95 (d, *J* = 10.7 Hz, 1H), 4.82 – 4.73 (m, 3H), 4.75 (d, *J* = 3.5 Hz, 1H, H-1), 4.63 (d, *J* = 11.9 Hz, 1H), 4.47 (d, *J* = 10.6 Hz, 1H), 4.43 (dd, *J* = 11.8, 2.0 Hz, 1H, H-6), 4.09 (dd, *J* = 11.8, 6.0 Hz, 1H, H-6), 3.95 (t, *J* = 9.3 Hz, 1H, H-3), 3.75 (ddd, *J* = 10.2, 5.9, 1.9 Hz, 1H, H-5), 3.68 (q, *J* = 8.7 Hz, 2H, CH_2CF_3), 3.48 (dd, *J* = 9.6, 3.7 Hz, 1H, H-2), 3.28 (dd, *J* = 10.2, 8.9 Hz, 1H, H-4), 1.67 (s, 3H, CH_3 DMNPA), 1.66 (s, 3H, CH_3 DMNPA); $^{13}C\{^1H\}$ NMR (126 MHz, $CDCl_3$, HSQC, HMBC, HMBC-Gated) δ 175.0 (C=O DMNPA), 148.5, 145.8, 139.3, 138.6, 138.0, 137.7, 133.5, 131.2, 129.4, 128.6, 128.6, 128.6, 128.2, 128.1, 128.1, 128.0, 127.8, 125.7, 124.9, 124.3 (q, *J* = 277.6 Hz, CF_3), 97.2 (C-1), 81.4 (C-3), 79.7 (C-2), 77.7 (C-4), 76.0, 75.3, 73.3, 69.5, 63.7 (C-6), 64.21 (q, *J* = 34.9 Hz), 46.6 (C_q DMNPA), 27.4 (CH_3 DMNPA, CH_3 DMNPA); ^{13}C-GATED NMR (126 MHz, $CDCl_3$) δ 97.2 (J_{H1-C1} = 171 Hz, α); HRMS (ESI) M/Z: $[M + NH_4]^+$ Calcd for $C_{39}H_{44}F_3N_2O_9^+$ 741.2993; Found 741.2989.

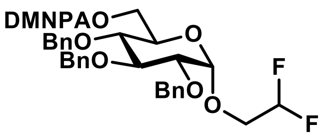

2,2-Difluoroethyl 6-*O*-(2,2-dimethyl-2-(ortho-nitrophenyl)acetyl)-2,3,4-tri-*O*-benzyl-α-D-glucopyranoside (S32). The title compound was prepared according to general procedure VI, using **7** as the donor and 2,2-difluoroethanol as the acceptor. Flash column chromatography (95:5 → 85:15, pentane:EtOAc v:v) yielded the title compound (47 mg, 0.067 mmol, 67%, α:β; 90:10) as a colorless oil. TLC: R_f 0.31, (80:20, pentane:EtOAc, v:v); ^{1}H NMR (400 MHz, $CDCl_3$, HH-COSY, HSQC, HMBC, HMBC-Gated) δ 7.94 – 7.21 (m, 31H), 6.04 – 5.79 (m, 0.11H, $CHCF_2$ β), 5.85 (tt, *J* = 55.6, 4.4 Hz, 1H, $CHCF_2$ α), 4.95 (d, *J* = 10.7 Hz, 1H), 4.93 – 4.86 (m, 0.11H), 4.82 – 4.74 (m, 3H), 4.68 (d, *J* = 3.7 Hz, 1H, H-1 α), 4.62 (d, *J* = 11.9 Hz, 1H), 4.47 (d, *J* = 10.5 Hz, 1H), 4.43 (dd, *J* = 11.8, 2.0 Hz, 1H, H-6 α), 4.08 (dd, *J* = 11.8, 6.1 Hz, 1H, H-6 α), 4.00 (dd, *J* = 11.8, 6.1 Hz, 0.11H, H-6 β), 3.97 – 3.93 (t, *J* = 9.5, 1H, H-3 α), 3.78 (ddd, *J* = 10.1, 6.1, 1.9 Hz, 1H, H-5 α), 3.66 – 3.59 (m, 0.12H), 3.57 – 3.49 (m, 2H, CH_2CHF_2 α), 3.46 (dd, *J* = 9.7, 3.6 Hz, 1H, H-2 α), 3.37 (dd, *J* = 9.2, 7.8 Hz, 0.11H, H-2 β), 3.28 (dd, *J* = 10.1, 8.9 Hz, 1H, H-4 α), 1.67 (s, 3H, CH_3 DMNPA α), 1.66 (s, 3H, CH_3 DMNPA); $^{13}C\{^1H\}$ NMR (126 MHz, $CDCl_3$, HSQC, HMBC, HMBC-Gated) δ 175.0 (C=O α), 174.9 (C=O β), 148.5, 145.7, 139.2, 138.6, 138.4, 138.3, 138.0, 137.7, 137.7, 133.5, 131.2, 129.4, 128.6, 128.6, 128.5, 128.5, 128.3, 128.2, 128.2, 128.1, 128.1, 128.1, 128.0, 127.9, 127.8, 125.8, 124.9, 114.05 (t, *J* = 241.3 Hz, CHF_2), 103.8 (C-1 β), 97.4 (C-1 α), 84.3 (C-3 β), 82.1 (C-2 β), 81.6 (C-3 α), 79.8 (C-2 α), 77.9 (C-4 α), 77.8 (C-4 β), 76.0, 75.9, 75.3, 75.1, 75.0, 73.4, 73.1 (C-5 β), 69.3 (C-5 α), 66.9 (t, *J* = 28.9 Hz, CH_2CHF_2), 63.8 (C-6 α), 63.4 (C-6 β), 46.7 (C_q DMNPA β), 46.6 (C_q DMNPA α), 27.5 (CH_3 DMNPA β), 27.4 (CH_3 DMNPA α, CH_3 DMNPA α), 27.1 (CH_3 DMNPA β); ^{13}C-GATED NMR (126 MHz, $CDCl_3$) δ 103.8 (J_{H1-C1} = 160 Hz, β), 97.4 (J_{H1-C1} = 168 Hz, α); HRMS (ESI) M/Z: $[M + NH_4]^+$ Calcd for $C_{39}H_{45}F_2N_2O_9^+$ 723.3088; Found 723.3085.

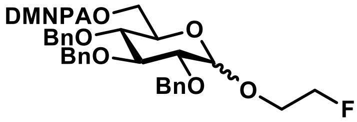

2-Fluoroethyl 6-*O*-(2,2-dimethyl-2-(ortho-nitrophenyl)acetyl)-2,3,4-tri-*O*-benzyl-D-glucopyranoside (S33). The title compound was prepared according to general procedure VI, using **7** as the donor and 2-Fluoroethanol as the acceptor. Flash column chromatography (90:10 → 75:25, pentane:EtOAc v:v) yielded the title compound (43 mg, 0.063, 63% mmol, α:β; 70:30) as a colorless oil. TLC: R_f 0.24, (80:20, pentane:EtOAc, v:v); ^{1}H NMR (500 MHz, $CDCl_3$, HH-COSY, HSQC, HMBC, HMBC-Gated) δ 7.95 – 7.18 (m, 44H), 4.99 – 4.89 (m, 2H), 4.82 – 4.74 (m, 4H), 4.73 (d, *J* = 3.6 Hz, 1H, H-1 α), 4.69 (d, *J* = 10.9 Hz, 0.55H, C*H*H Bn β), 4.67 – 4.43 (m, 8H), 4.42 (d, *J* = 7.8 Hz, 0.45H, H-1 β), 4.11 (dd, *J* = 11.8, 5.5 Hz, 1H, H-6 α), 4.04 (dd, *J* = 11.7, 5.7 Hz, 1H, H-6 βffzgff), 4.01 – 3.91 (m, 2H), 3.87 – 3.73 (m, 2H), 3.70 – 3.53 (m, 3H), 3.50 – 3.43 (m, 2H), 3.38 (dd, *J* = 9.2, 7.8 Hz, 0.45H, H-2 β), 3.34 – 3.26 (m, 2H), 1.68 (s, 1.35H, CH_3 DMNPA β), 1.67 (s, 1.35H, CH_3 DMNPA β), 1.67 (s, 3H, CH_3 DMNPA α), 1.66 (s, 3H, CH_3 DMNPA α); ^{13}C{^{1}H} NMR (126 MHz, $CDCl_3$, HSQC, HMBC, HMBC-Gated) δ 175.0 (C=O α), 175.0 (C=O β), 148.7, 148.6, 145.7, 139.3, 139.3, 138.7, 138.5, 138.5, 138.4, 138.2, 137.9, 137.8, 133.4, 133.3, 131.2, 129.4, 128.6, 128.6, 128.5, 128.5, 128.5, 128.4, 128.4, 128.2, 128.2, 128.2, 128.1, 128.1, 128.1, 128.0, 128.0, 128.0, 127.8, 127.8, 127.8, 125.7, 125.7, 124.9, 103.5 (C-1 β), 96.9 (C-1 α), 84.4 (C-3 β), 82.7 (d, *J* = 169.6 Hz, CH_2F β), 82.2 (C-2 β), 82.4 (d, *J* = 169.9 Hz, CH_2F α), 81.8 (C-3 α), 80.0 (C-2 α), 78.0 (C-4 α), 77.9 (C-4 β), 75.9, 75.9, 75.3, 75.1, 74.9, 73.2, 72.9 (C-5 β), 68.9 (C-5 α), 68.7 (d, *J* = 20.0 Hz, CH_2CH_2F β), 66.7 (d, *J* = 20.4 Hz, CH_2CH_2F α), 63.8 (C-6 α), 63.5 (C-6 β), 46.7 (C_q DMNPA α, C_q DMNPA β), 27.4 (CH_3 DMNPA β), 27.4 (CH_3 DMNPA β), 27.4 (CH_3 DMNPA α, CH_3 DMNPA β); ^{13}C-GATED NMR (126 MHz, $CDCl_3$) δ 103.5 ($J_{H1\text{-}C1}$ = 158 Hz, β), 96.9 ($J_{H1\text{-}C1}$ = 168 Hz, α); HRMS (ESI) M/Z: $[M + NH_4]^+$ Calcd for $C_{39}H_{46}FN_2O_9^+$ 705.3182; Found 705.3179.

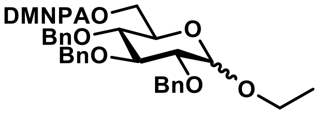

Ethyl 6-*O*-(2,2-dimethyl-2-(ortho-nitrophenyl)acetyl)-2,3,4-tri-*O*-benzyl-D-glucopyranoside (S34). The title compound was prepared according to general procedure VI, using **7** as the donor and ethanol as the acceptor. Flash column chromatography (100:0 → 80:20, pentane:EtOAc v:v) yielded the title compound (52 mg, 0.078 mmol, 78%, α:β; 60:40) as a colorless oil. TLC: R_f 0.44, (80:20, pentane:EtOAc, v:v); ^{1}H NMR (500 MHz, $CDCl_3$, HH-COSY, HSQC, HMBC, HMBC-Gated) δ7.7 – 7.2 (m, 95H), 5.0 (d, *J* = 10.7 Hz, 1H), 5.0 – 4.7 (m, 12H), 4.7 (d, *J* = 3.6 Hz, 1H, H-1 α), 4.7 – 4.4 (m, 7H), 4.4 (d, *J* = 7.8 Hz, 1H, H-1 β), 4.1 (dd, *J* = 11.8, 5.7 Hz, 1H, H-6 α), 4.0 (dd, *J* = 11.7, 6.1 Hz, 1H, H-6 β), 4.0 (d, *J* = 9.2 Hz, 1H, H-3 α), 3.9 – 3.8 (m, 2H), 3.6 (t, *J* = 9.1 Hz, 1H, H-3 β), 3.6 – 3.2 (m, 9H), 1.7 (s, 3H, CH_3 DMNPA α), 1.7 (s, 3H, CH_3 DMNPA β), 1.7 (s, 3H, CH_3 DMNPA α), 1.7 (s, 3H, CH_3 DMNPA β), 1.2 (t, *J* = 7.2 Hz, 3H, CH_3 Et β), 1.1 (t, *J* = 7.1 Hz, 3H, CH_3 Et α); ^{13}C{^{1}H} NMR (126 MHz, $CDCl_3$, HSQC, HMBC, HMBC-Gated) δ 175.1 (C=O β), 175.0 (C=O α), 145.7, 139.4, 139.3, 139.2, 138.8, 138.6, 138.6, 138.3, 138.3, 138.1, 137.9, 137.9, 137.7, 133.5, 131.2, 129.4, 129.0, 128.6, 128.6, 128.6, 128.6, 128.5, 128.5, 128.5, 128.2, 128.2, 128.2, 128.2, 128.1, 128.1, 128.0, 127.9, 127.8, 127.8, 127.8, 125.8, 125.7, 124.9, 103.3 (C-1 β), 96.2 (C-1 α), 84.6 (C-3 β), 82.4 (C-2 β), 82.0 (C-3 α), 80.1 (C-2 α), 78.3, 78.2, 75.9, 75.9, 75.3, 75.1, 74.9, 73.2, 72.9, 68.8, 65.5 (C-6 β), 63.9 (CH_2 α), 63.8 (CH_2 β), 63.1 (C-6 α), 46.7 (C_q DMNPA α), 46.7 (C_q DMNPA β), 27.4 (CH_3 DMNPA α), 27.4 (CH_3 DMNPA α, CH_3 DMNPA β), 27.4 (CH_3 DMNPA β), 15.5, 15.0; ^{13}C-GATED NMR (126 MHz, $CDCl_3$) δ 103.3 ($J_{H1\text{-}C1}$ = 157 Hz, β), 96.2 ($J_{H1\text{-}C1}$ = 167 Hz, α); HRMS (ESI) M/Z: $[M + NH_4]^+$ Calcd for $C_{39}H_{47}N_2O_9^+$ 687.3276; Found 687.3278.

NMR spectra of new and selected compounds

Benzylated donor 3, 5, 7 NMR spectra

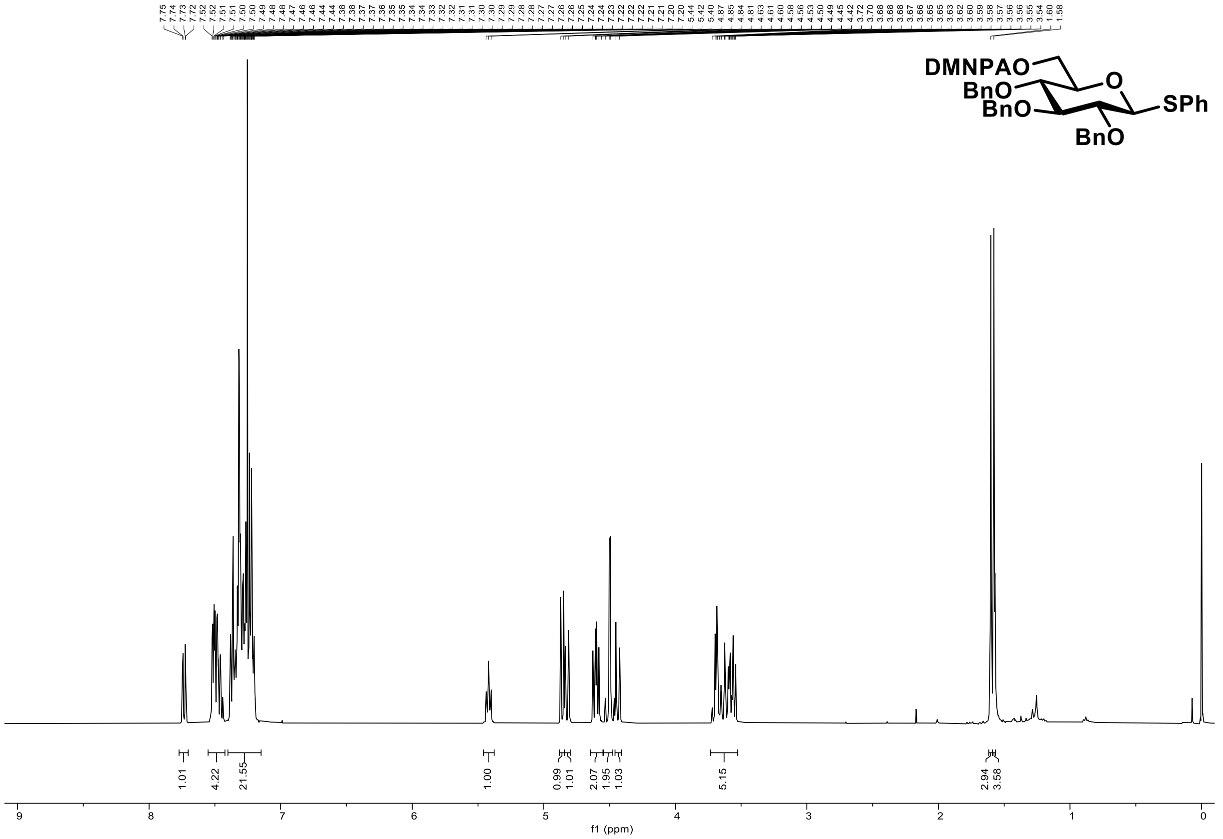

Supplementary Figure S5. ^{1}H NMR, 400 MHz, $CDCl_3$ of compound **3**

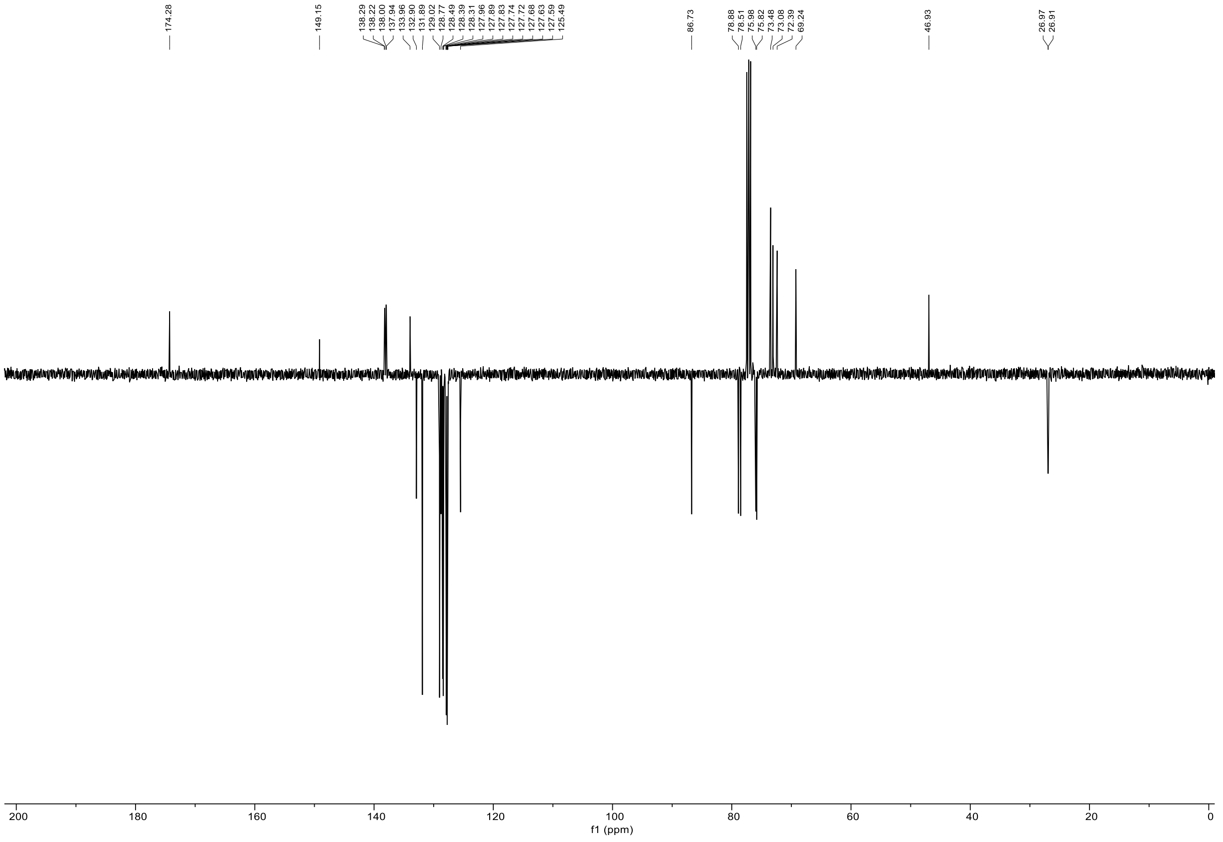

Supplementary Figure S6. $^{13}C\{^1H\}$ NMR, 101 MHz, $CDCl_3$ of compound **3**

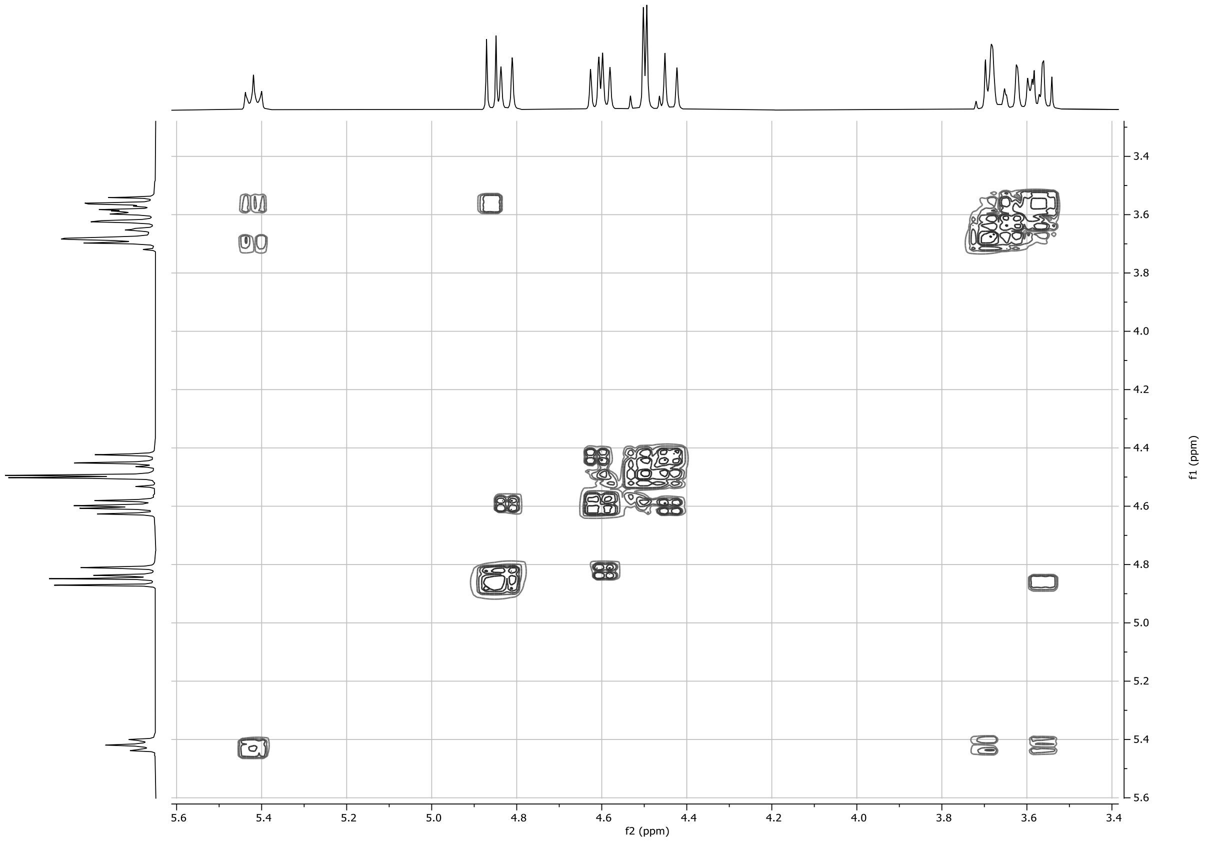

Supplementary Figure S7. HH-COSY NMR, $CDCl_3$ of compound **3**

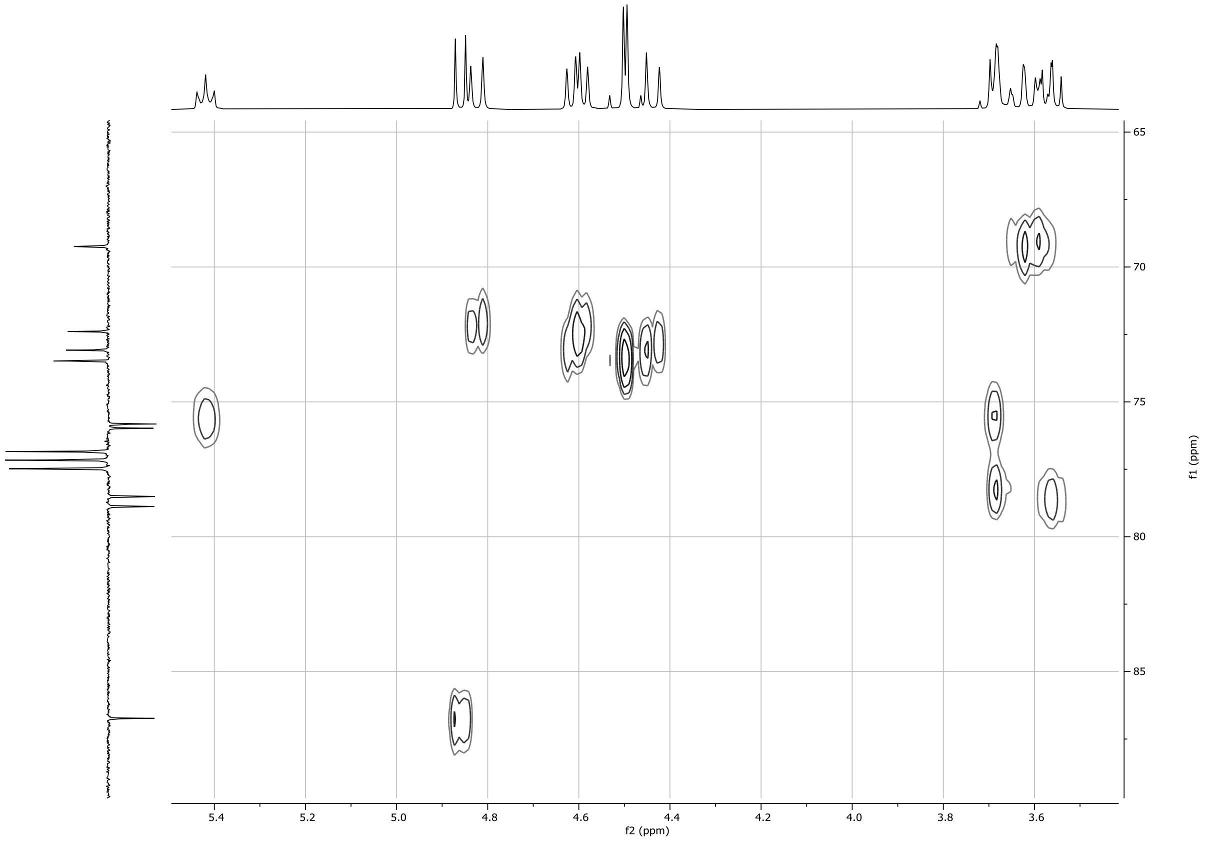

Supplementary Figure S8. HSQC{^{1}H} NMR, $CDCl_3$ of compound **3**

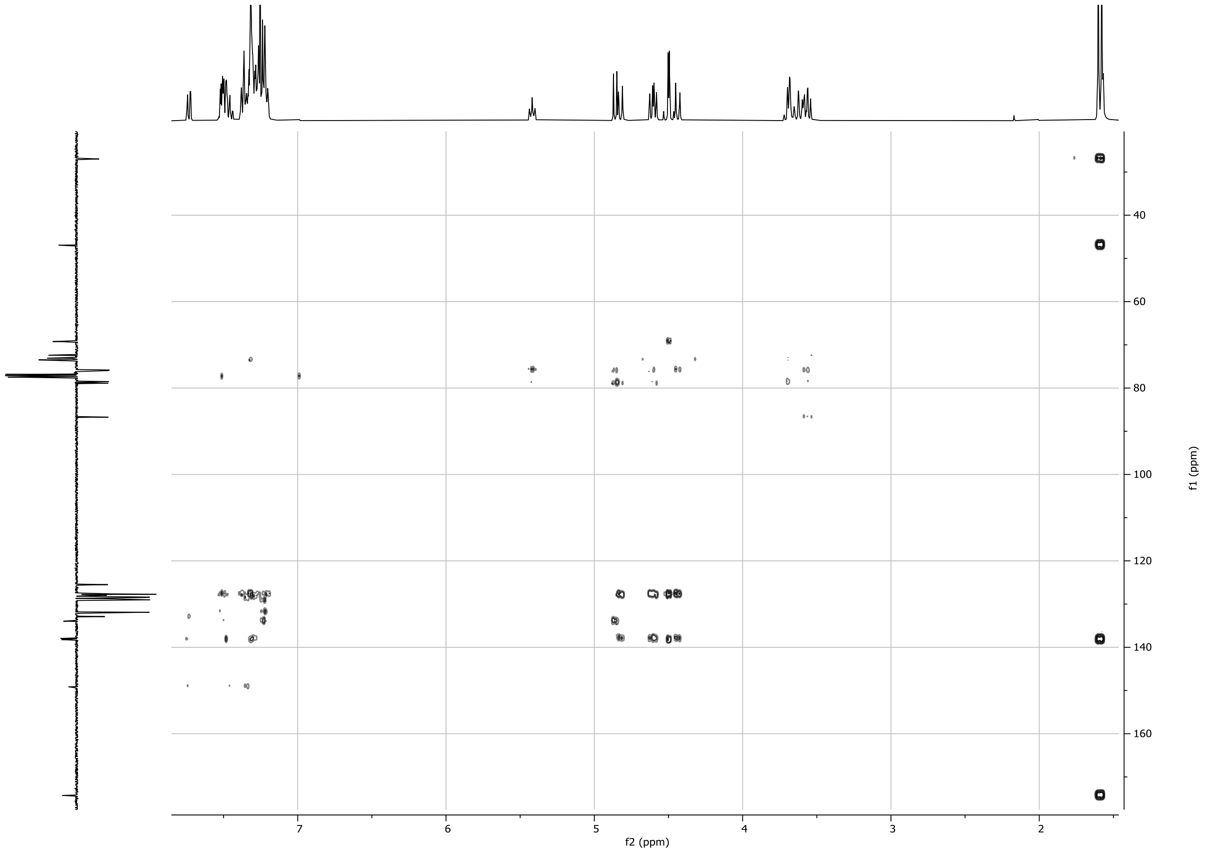

Supplementary Figure S9. HMBC{^{1}H} NMR, $CDCl_3$ of compound **3**

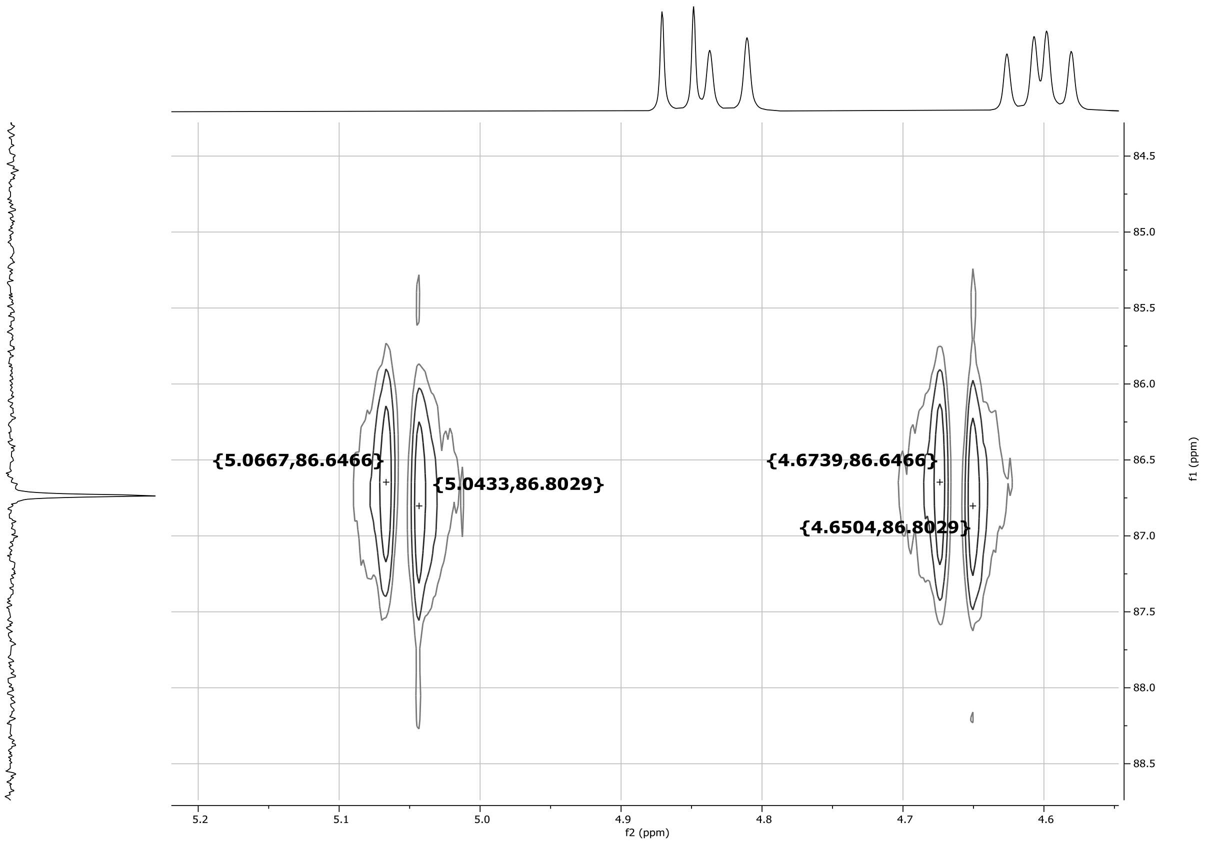

Supplementary Figure S10. HMBC-Gated NMR, $CDCl_3$ of compound **3**

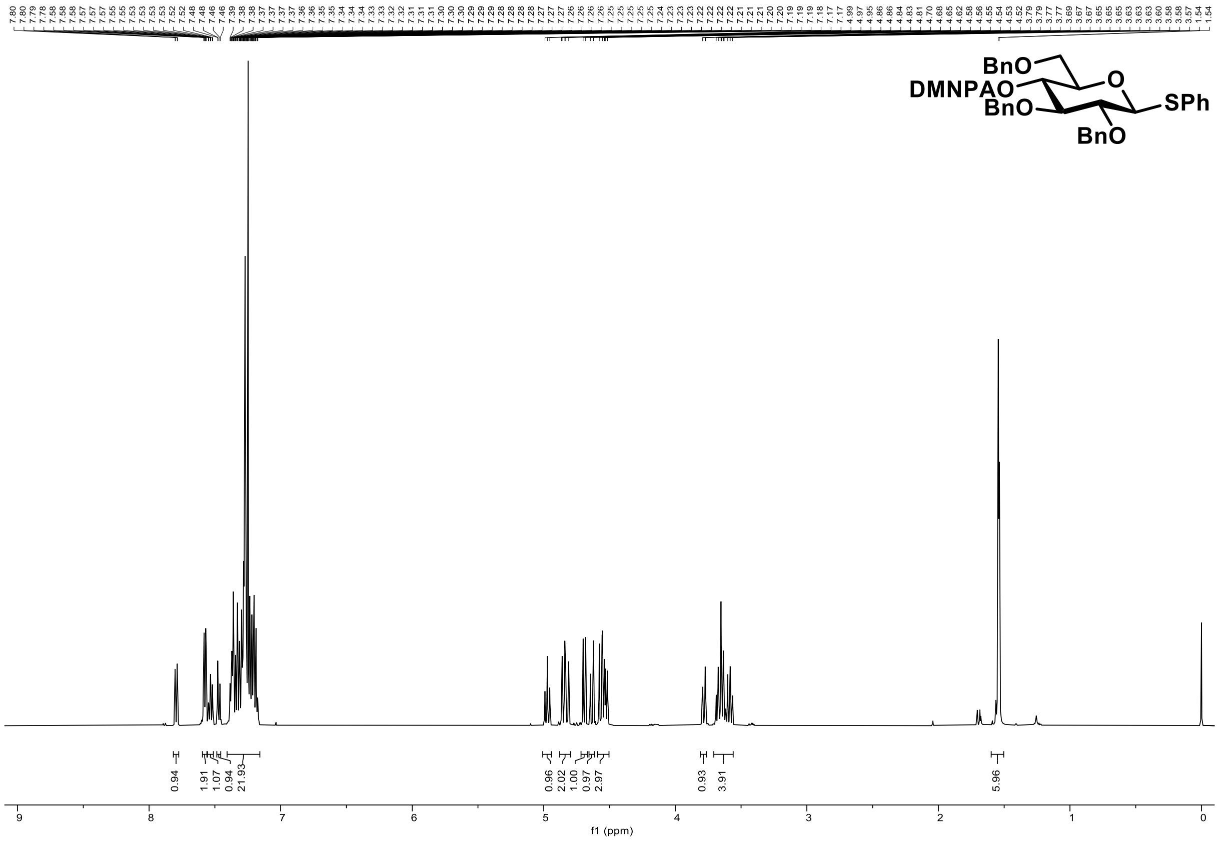

Supplementary Figure S11. ^{1}H NMR, 500 MHz, $CDCl_3$ of compound **5**

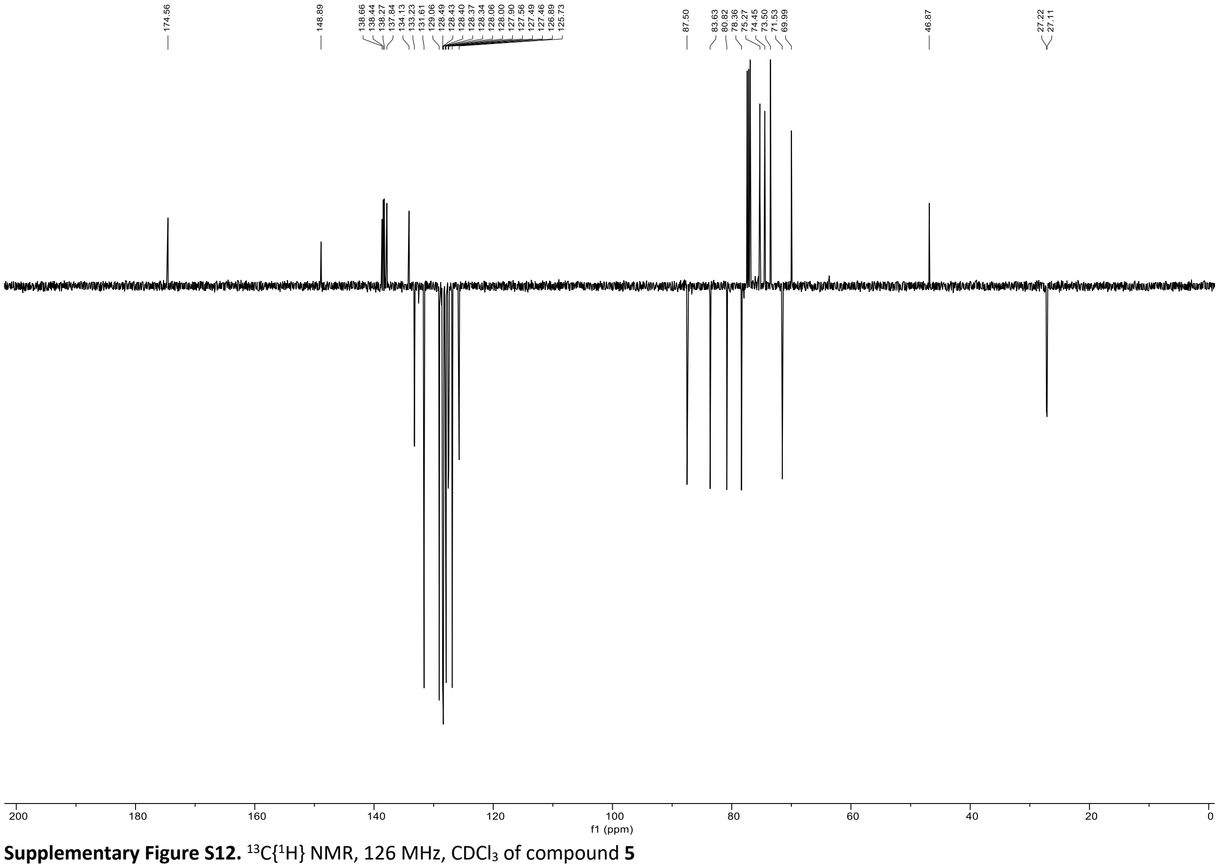

Supplementary Figure S12. $^{13}C\{^{1}H\}$ NMR, 126 MHz, $CDCl_3$ of compound **5**

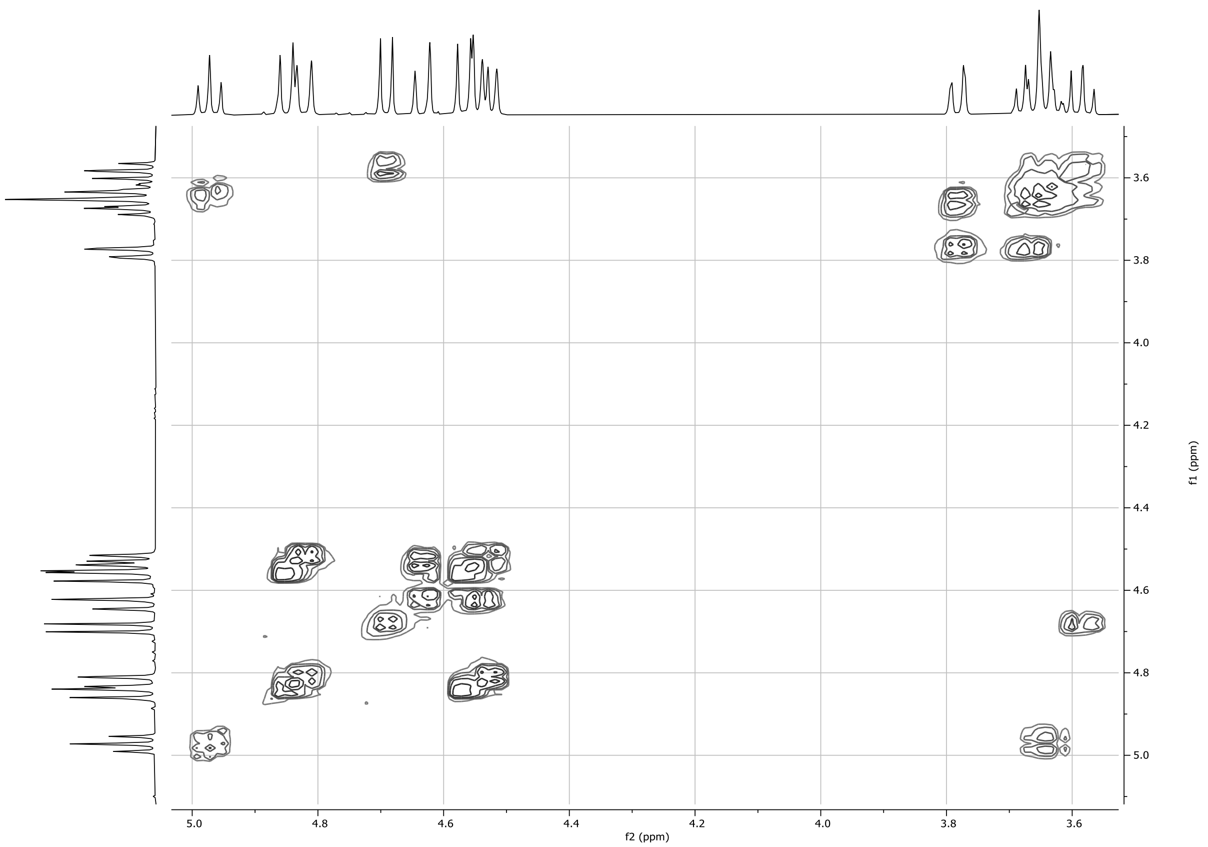

Supplementary Figure S13. HH-COSY NMR, $CDCl_3$ of compound **5**

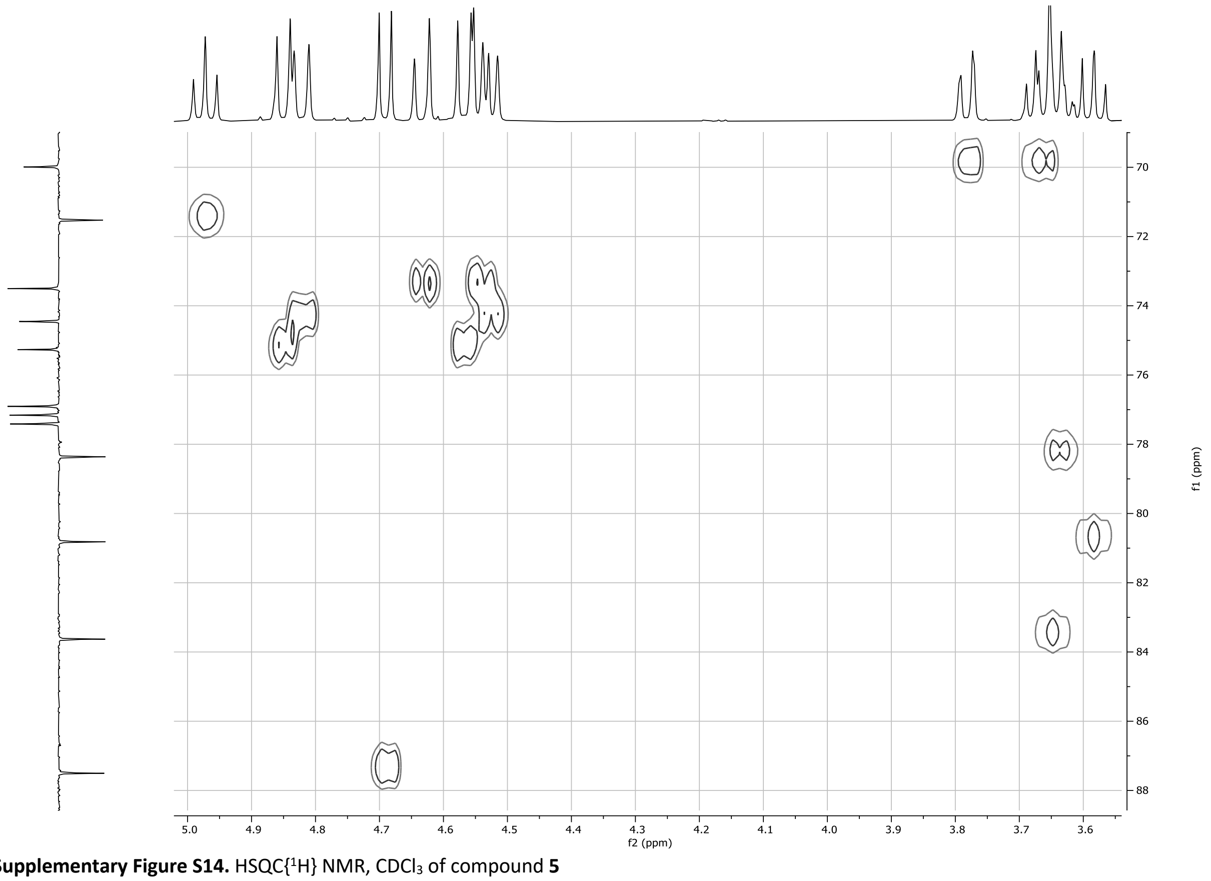

Supplementary Figure S14. HSQC{^{1}H} NMR, $CDCl_3$ of compound **5**

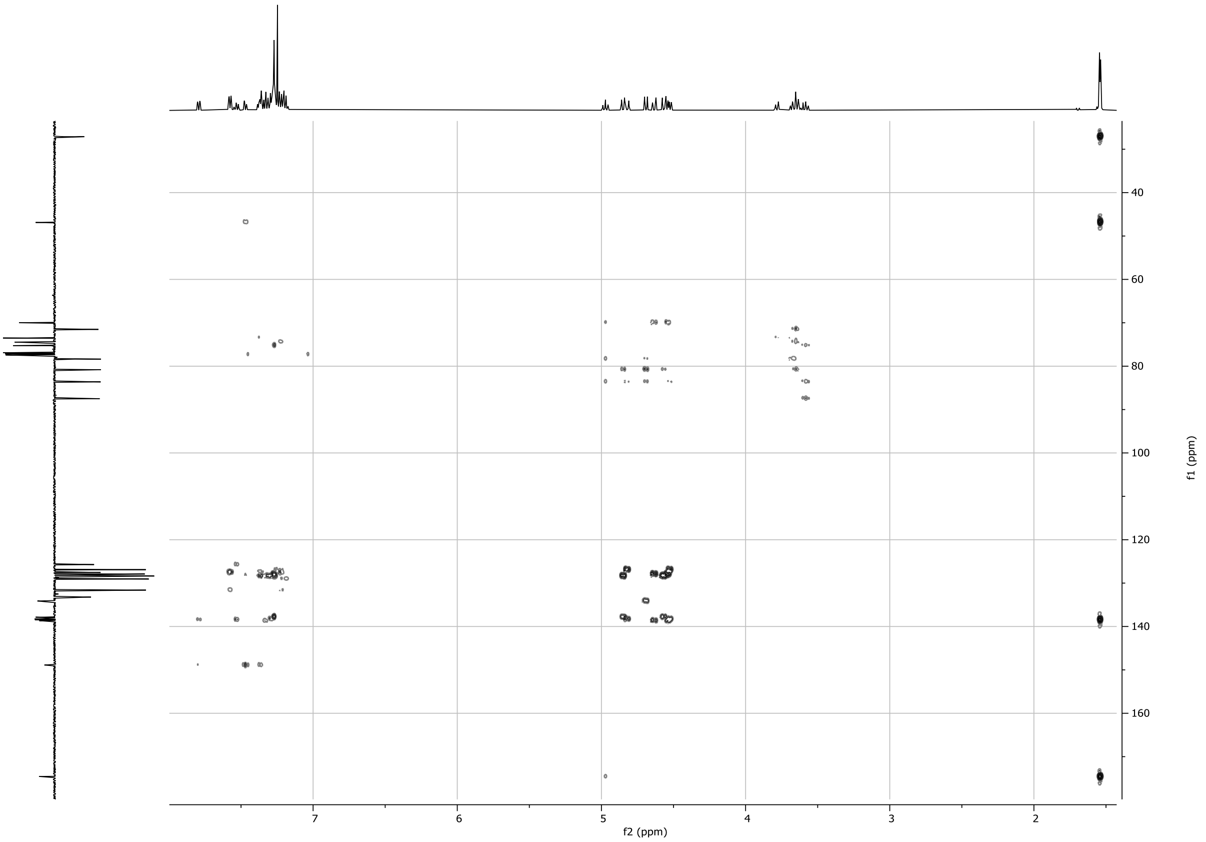

Supplementary Figure S15. HMBC{^{1}H} NMR, $CDCl_3$ of compound **5**

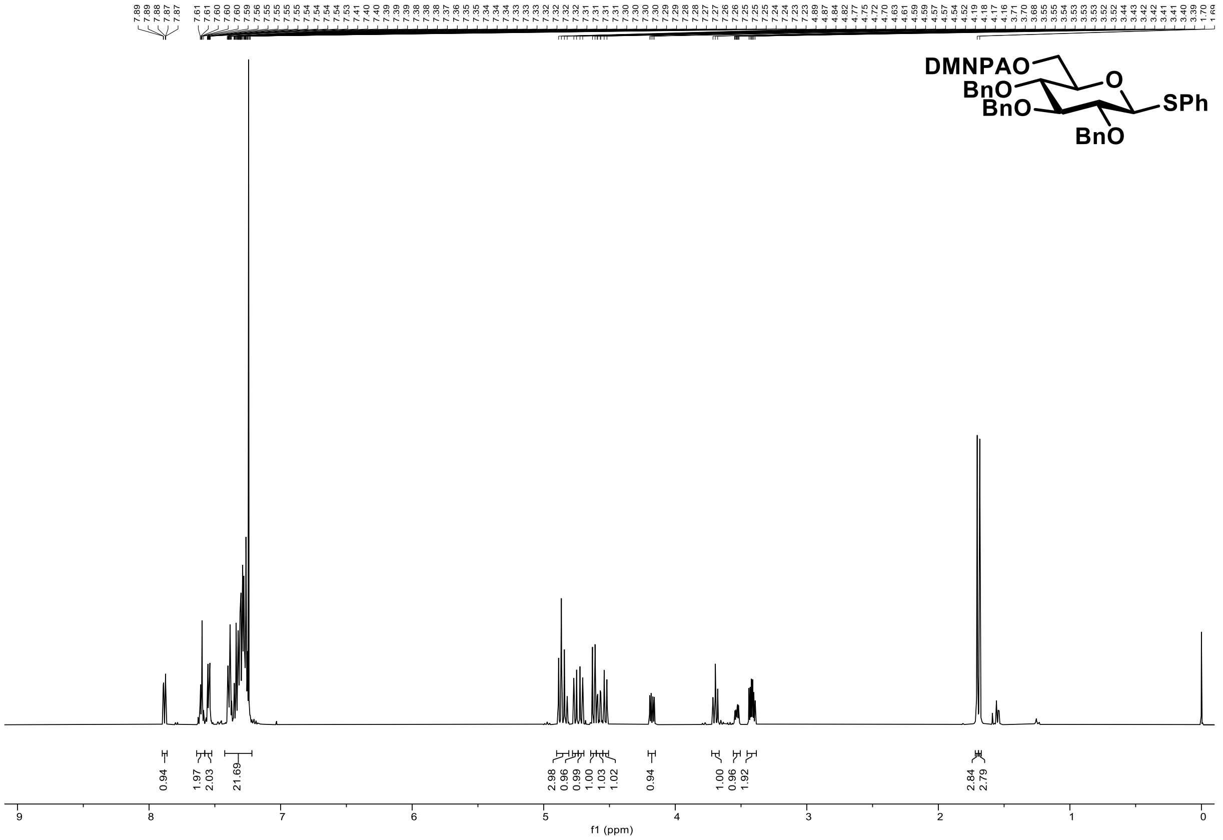

Supplementary Figure S16. ^{1}H NMR, 500 MHz, $CDCl_3$ of compound **7**

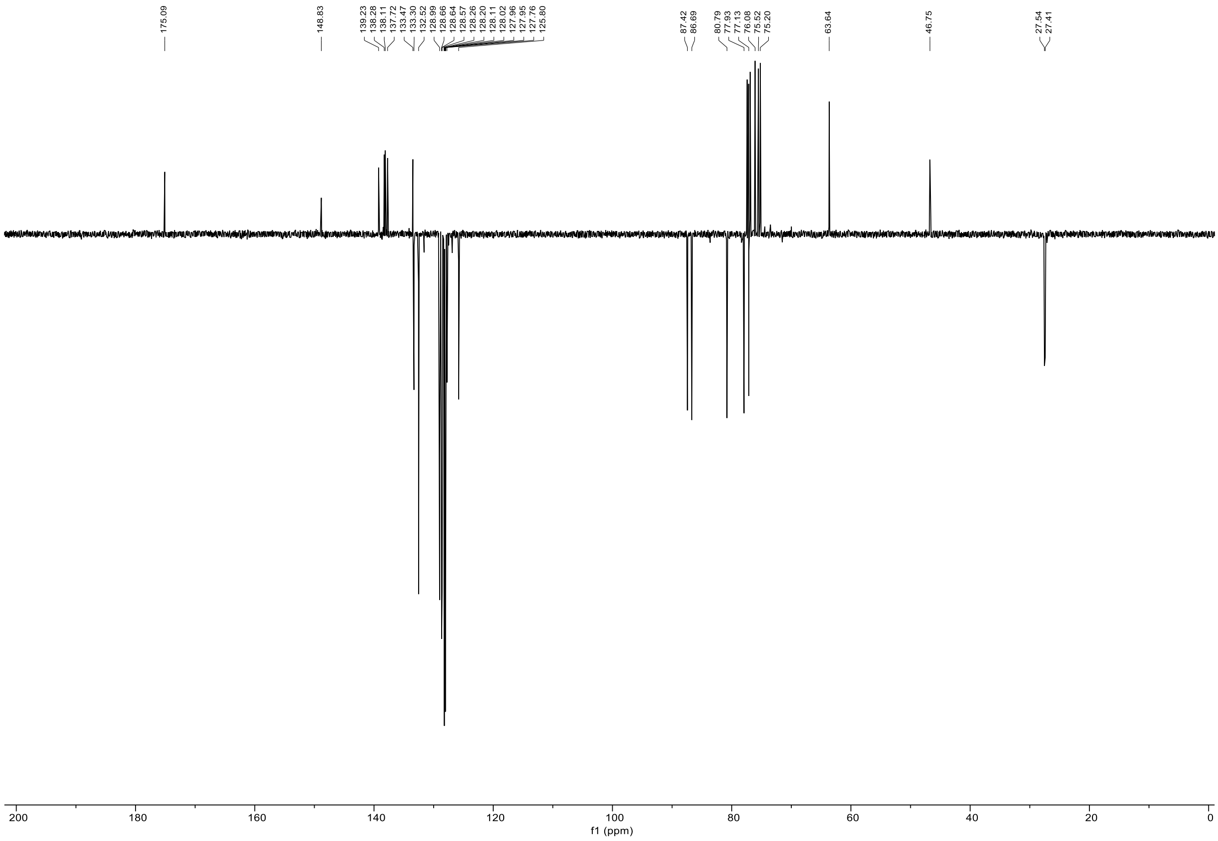

Supplementary Figure S17. $^{13}C\{^1H\}$ NMR, 126 MHz, $CDCl_3$ of compound **7**

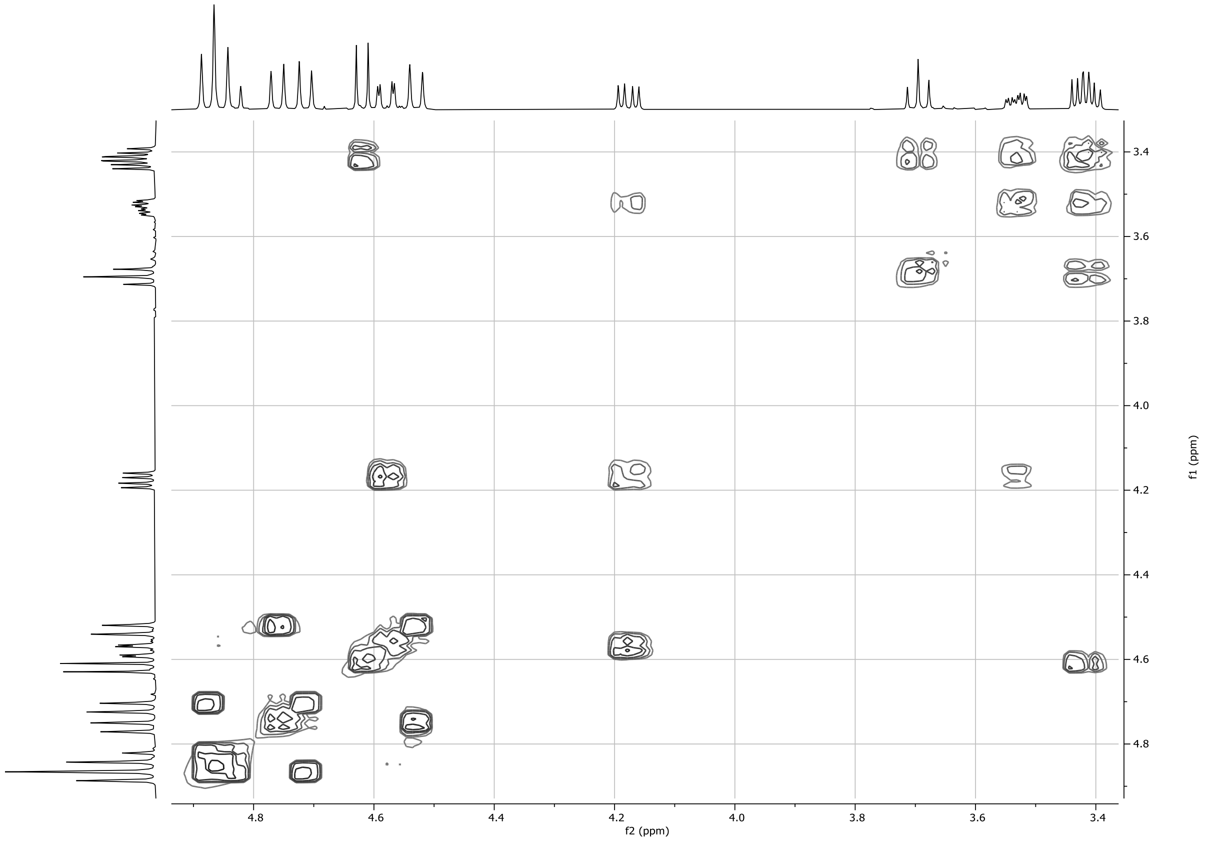

Supplementary Figure S18. HH-COSY NMR, $CDCl_3$ of compound **7**

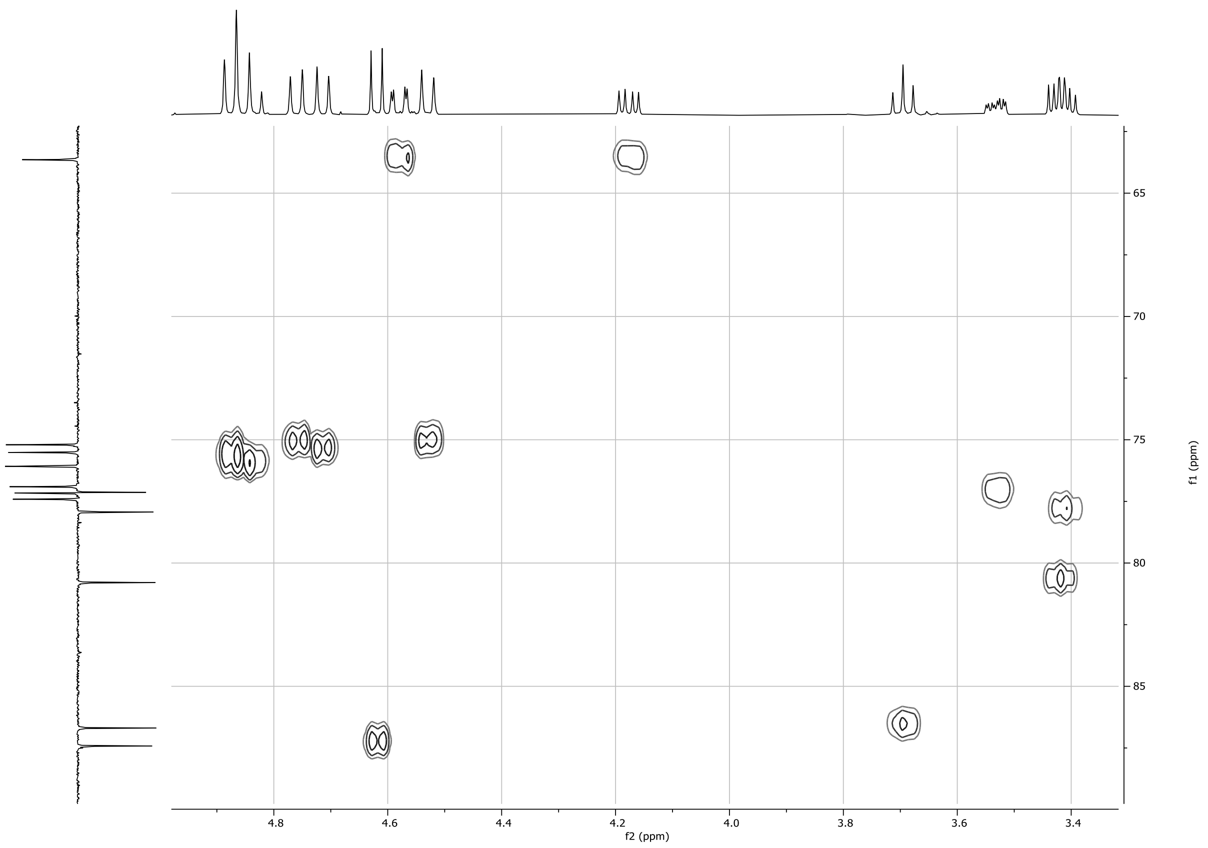

Supplementary Figure S19. HSQC{1H} NMR, $CDCl_3$ of compound **7**

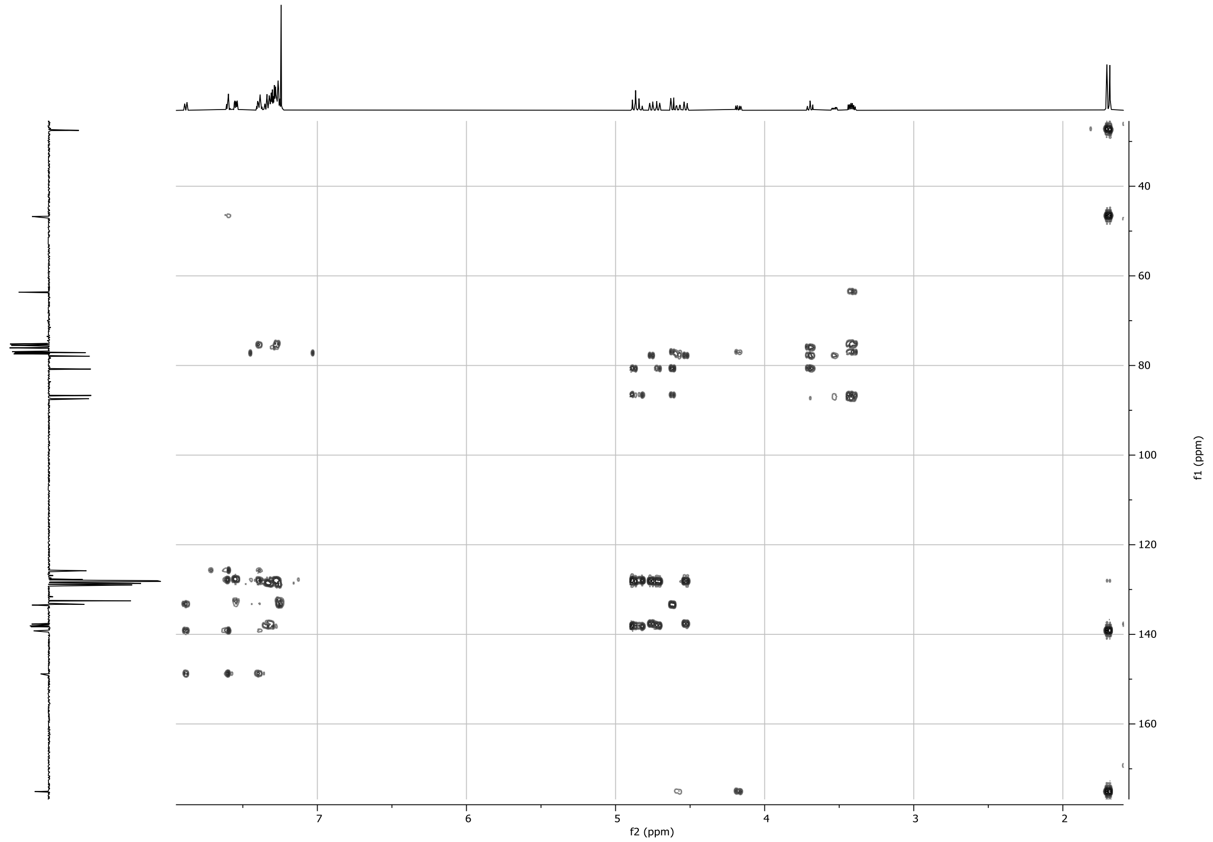

Supplementary Figure S20. HMBC{^{1}H} NMR, $CDCl_3$ of compound **7**

Benzylated donor intermediates NMR spectra

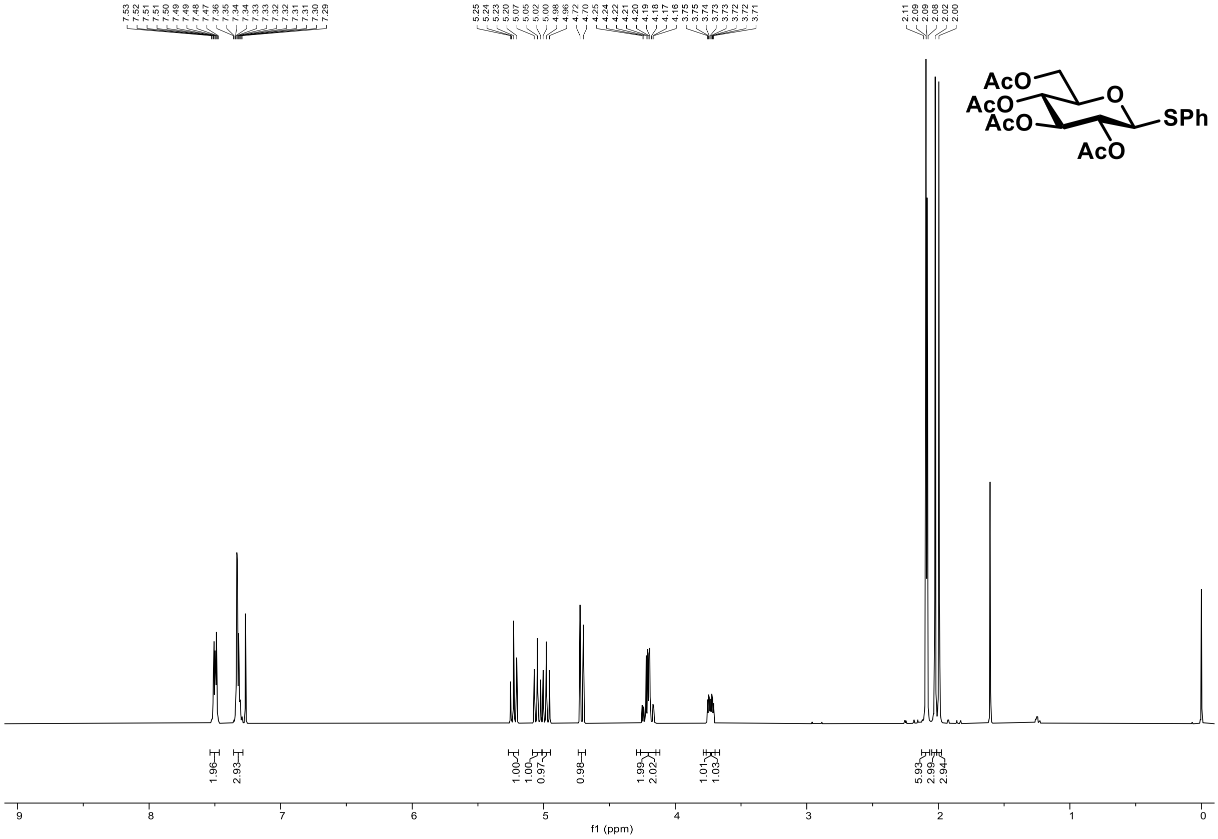

Supplementary Figure S21. ^{1}H NMR, 400 MHz, $CDCl_3$ of compound **S1**

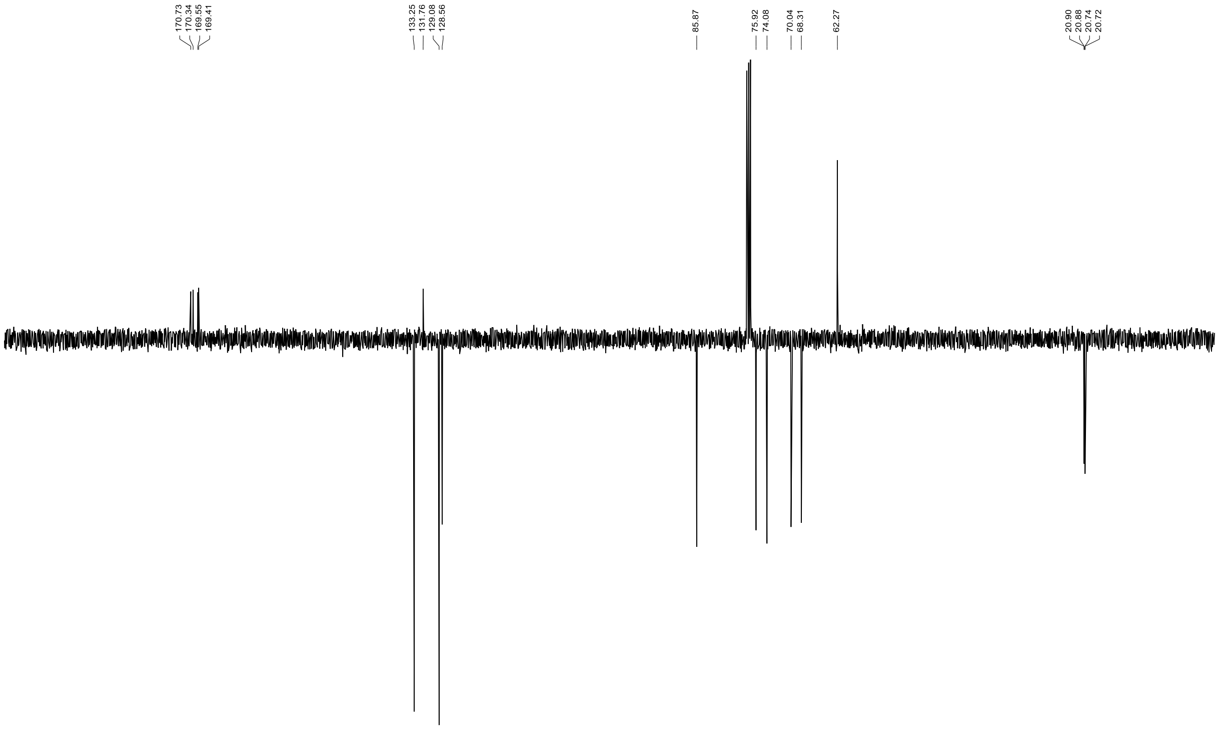

Supplementary Figure S22. $^{13}C\{^{1}H\}$ NMR, 101 MHz, $CDCl_3$ of compound **S1**

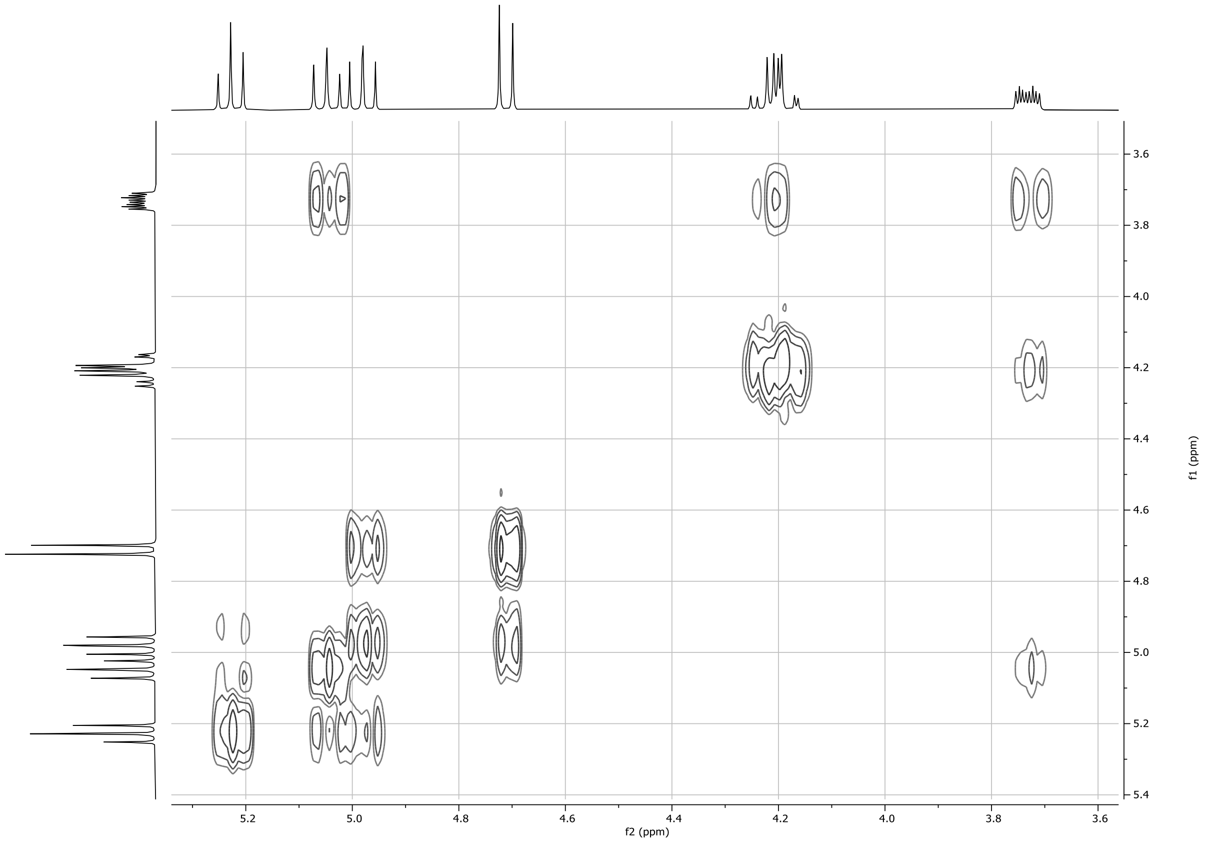

Supplementary Figure S23. HH-COSY NMR, $CDCl_3$ of compound **S1**

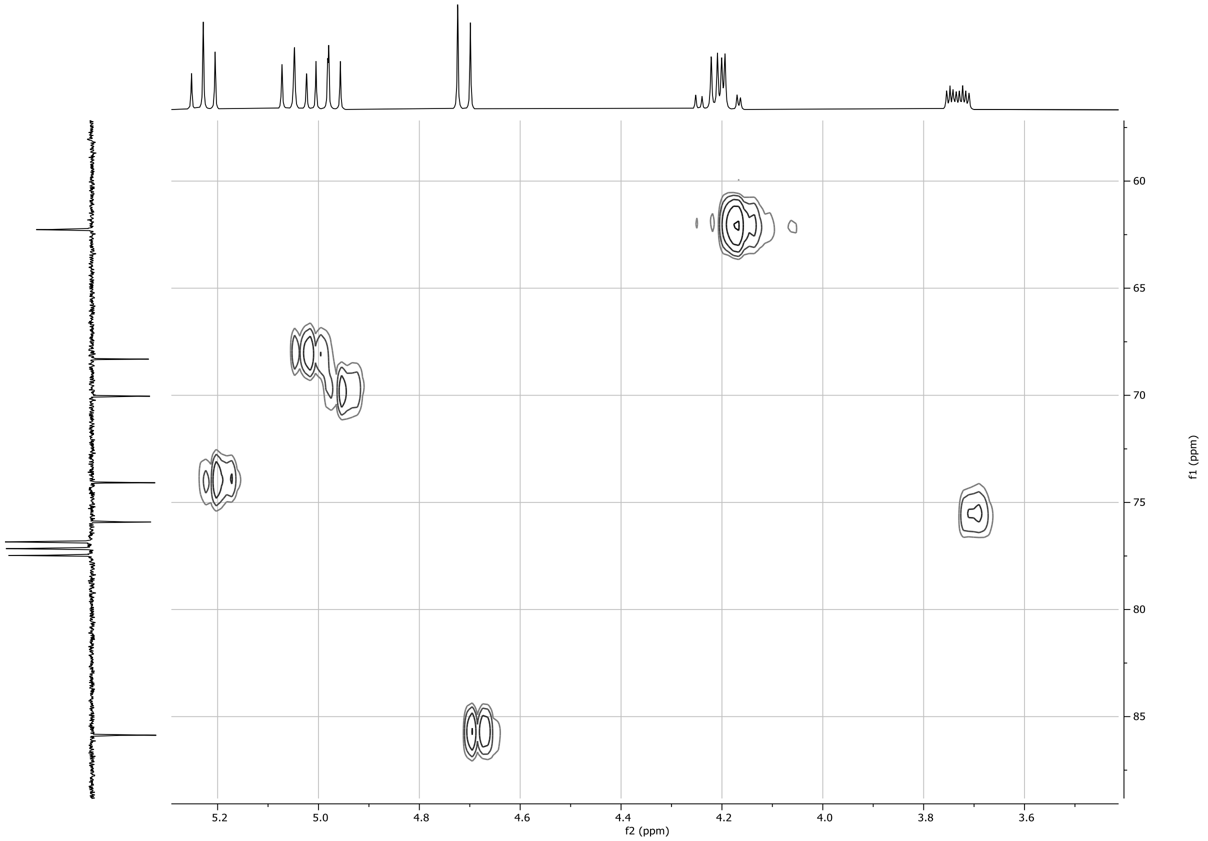

Supplementary Figure S24. HSQC{^{1}H} NMR, $CDCl_3$ of compound **S1**

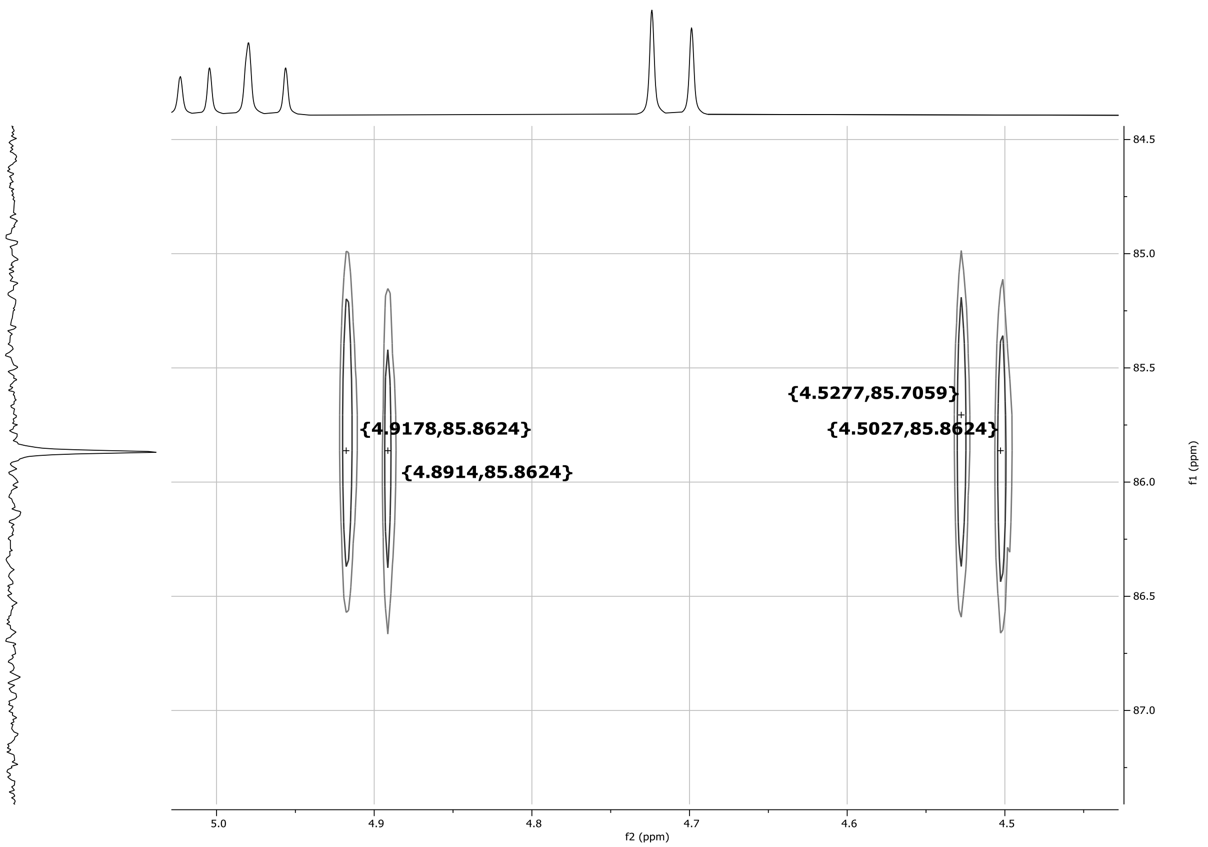

Supplementary Figure S25. HMBC{^{1}H} NMR, $CDCl_3$ of compound **S1**

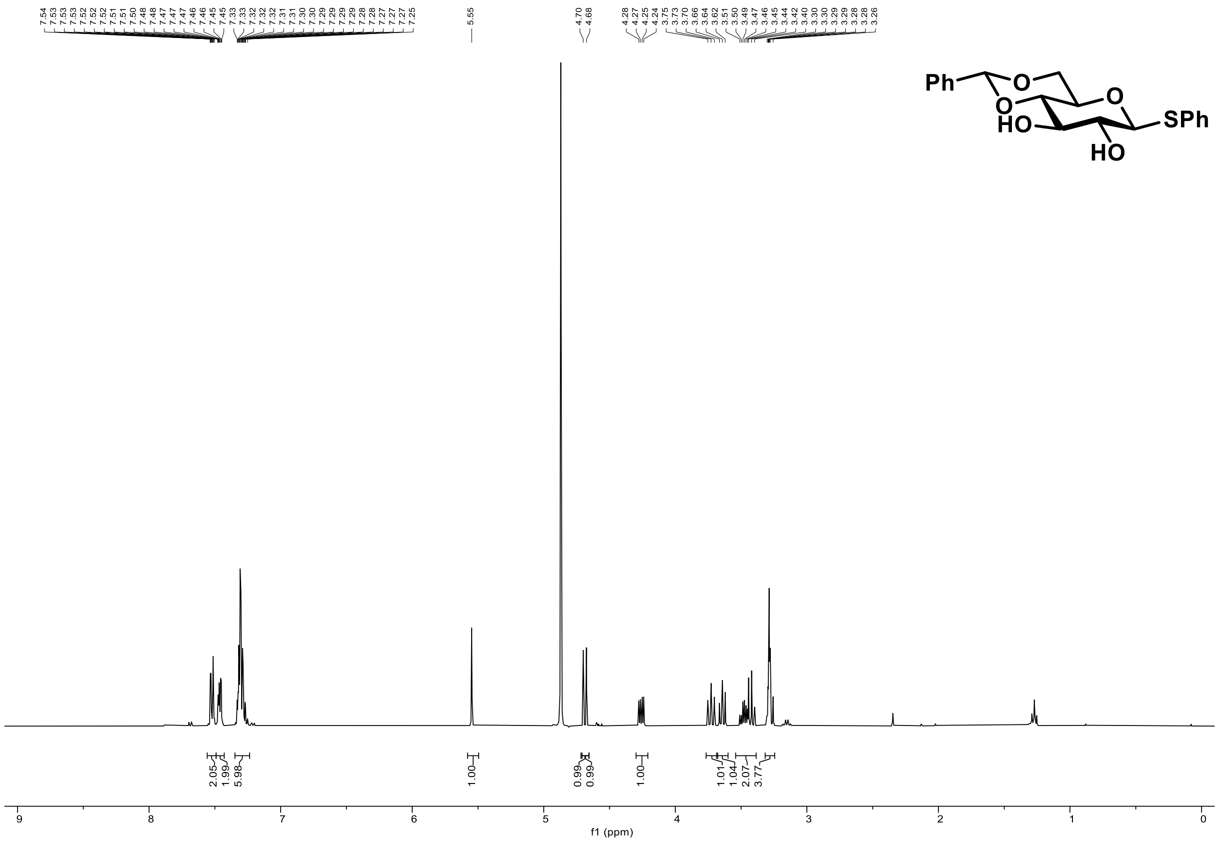

Supplementary Figure S26. ^{1}H NMR, 400 MHz, $CDCl_3$ of compound **S2**

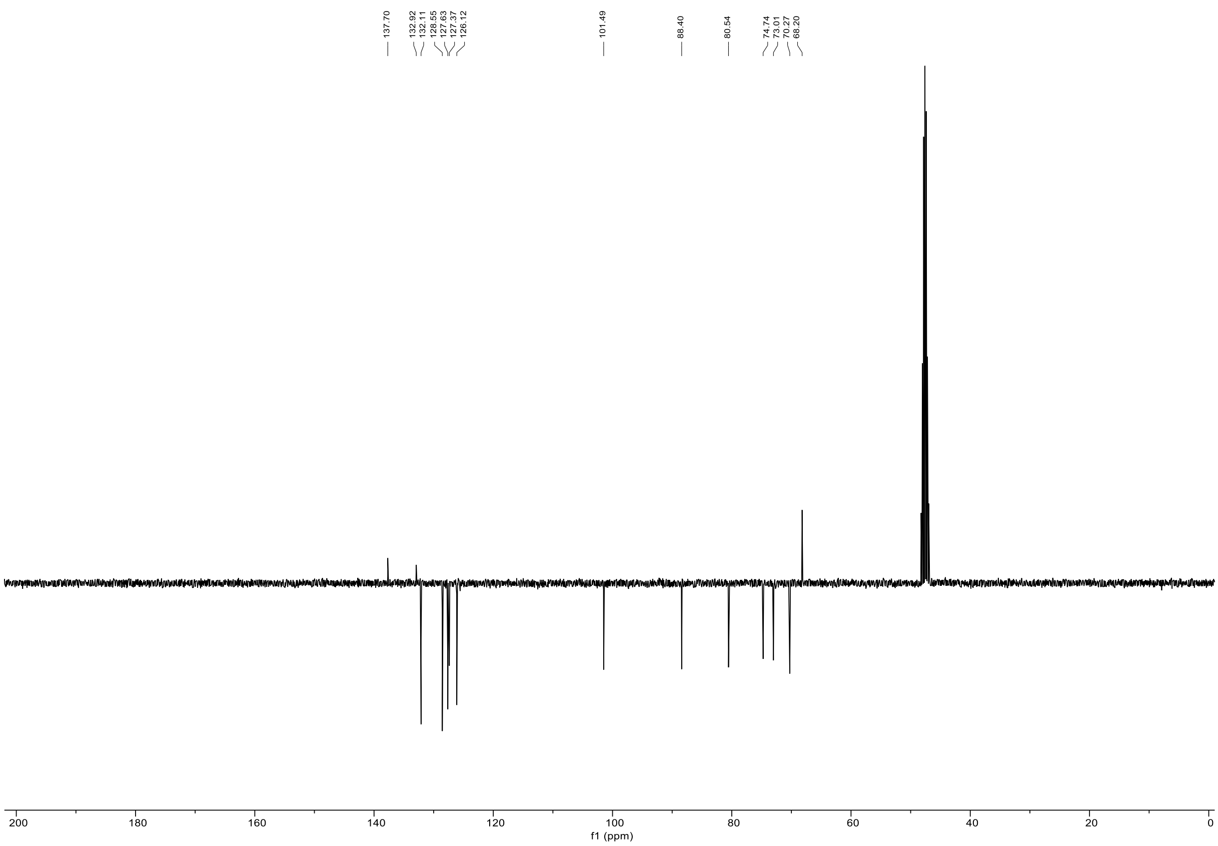

Supplementary Figure S27. $^{13}C\{^1H\}$ NMR, 101 MHz, $CDCl_3$ of compound **S2**

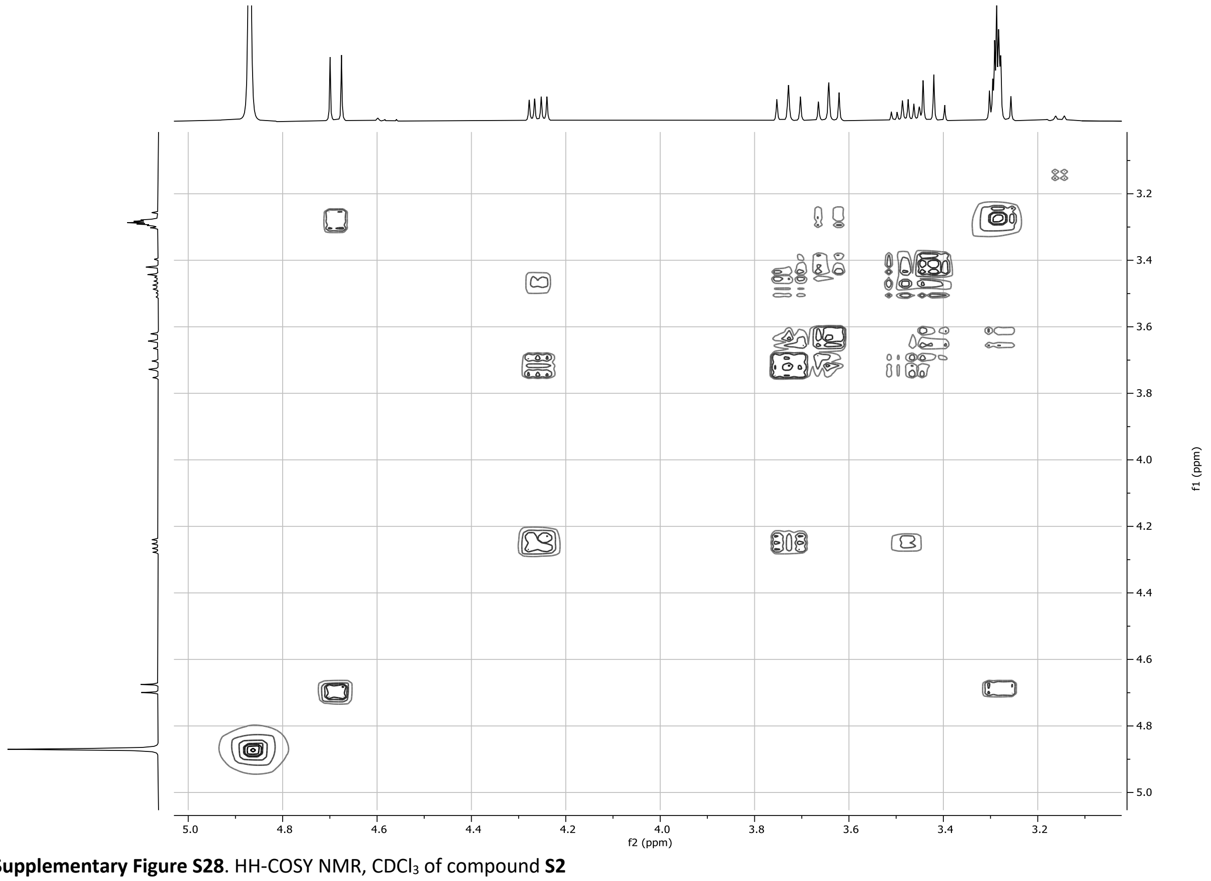

Supplementary Figure S28. HH-COSY NMR, $CDCl_3$ of compound **S2**

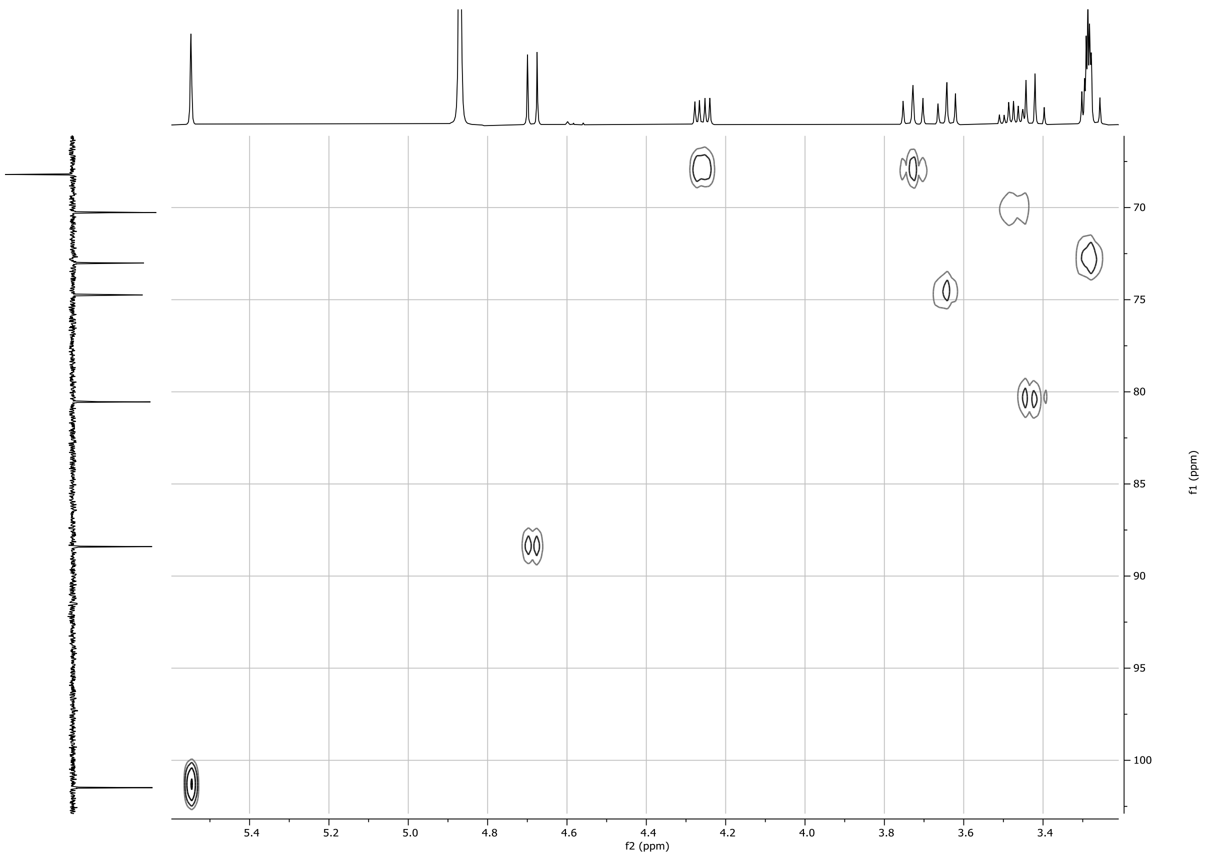

Supplementary Figure S29. HSQC{^{1}H} NMR, $CDCl_3$ of compound **S2**

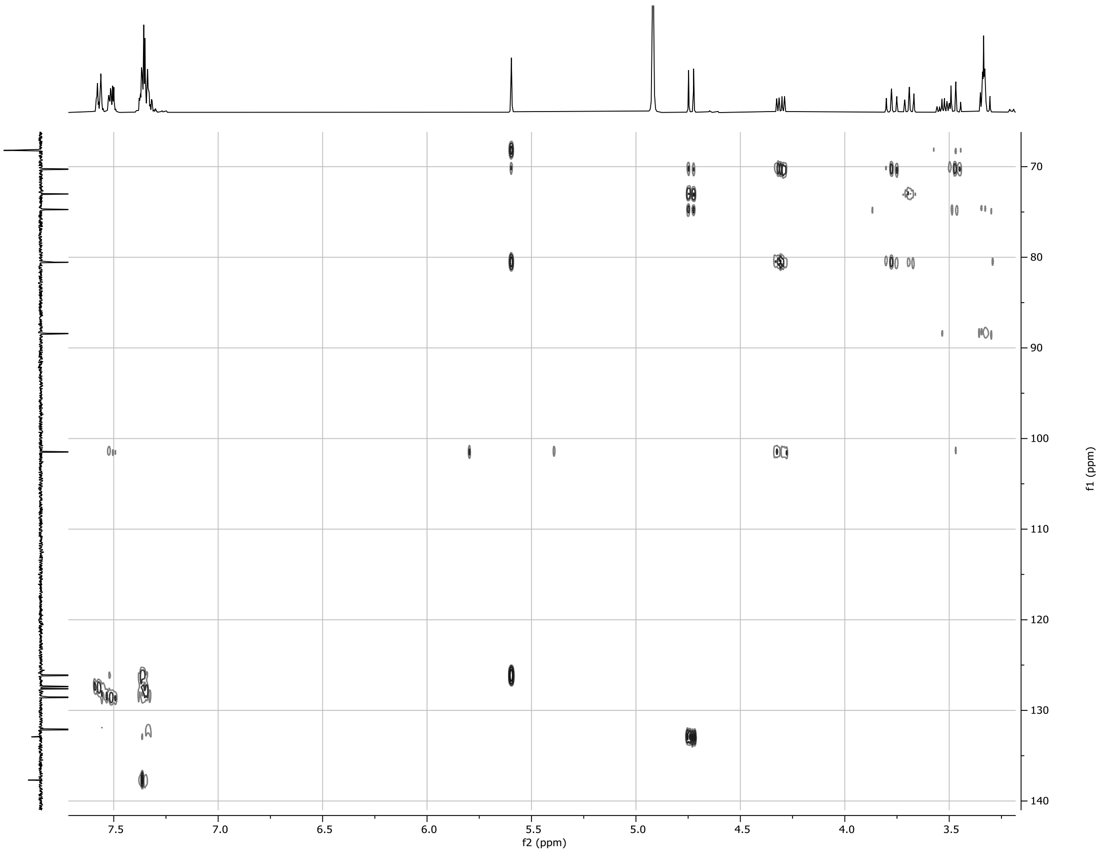

Supplementary Figure S30. HMBC{^{1}H} NMR, $CDCl_3$ of compound **S2**

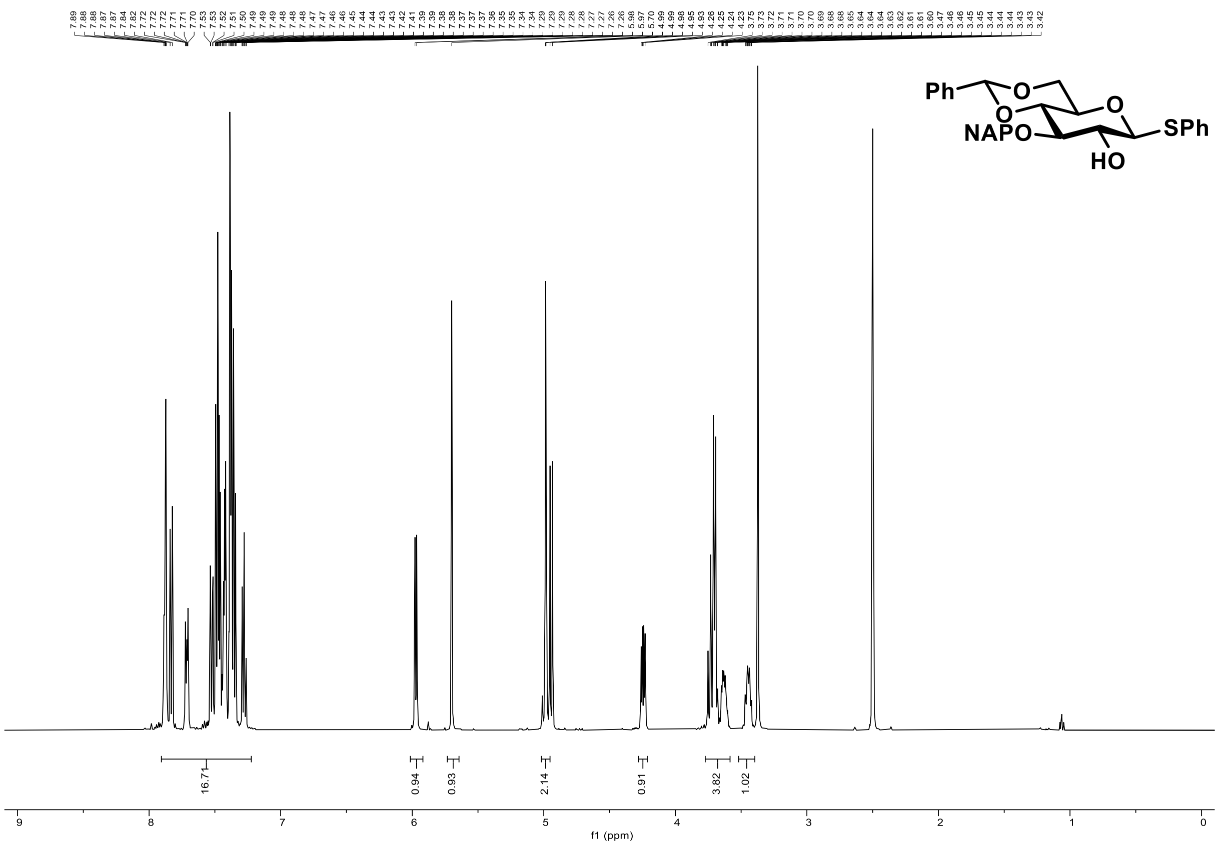

Supplementary Figure S31. ^{1}H NMR, 500 MHz, $CDCl_3$ of compound **S3**

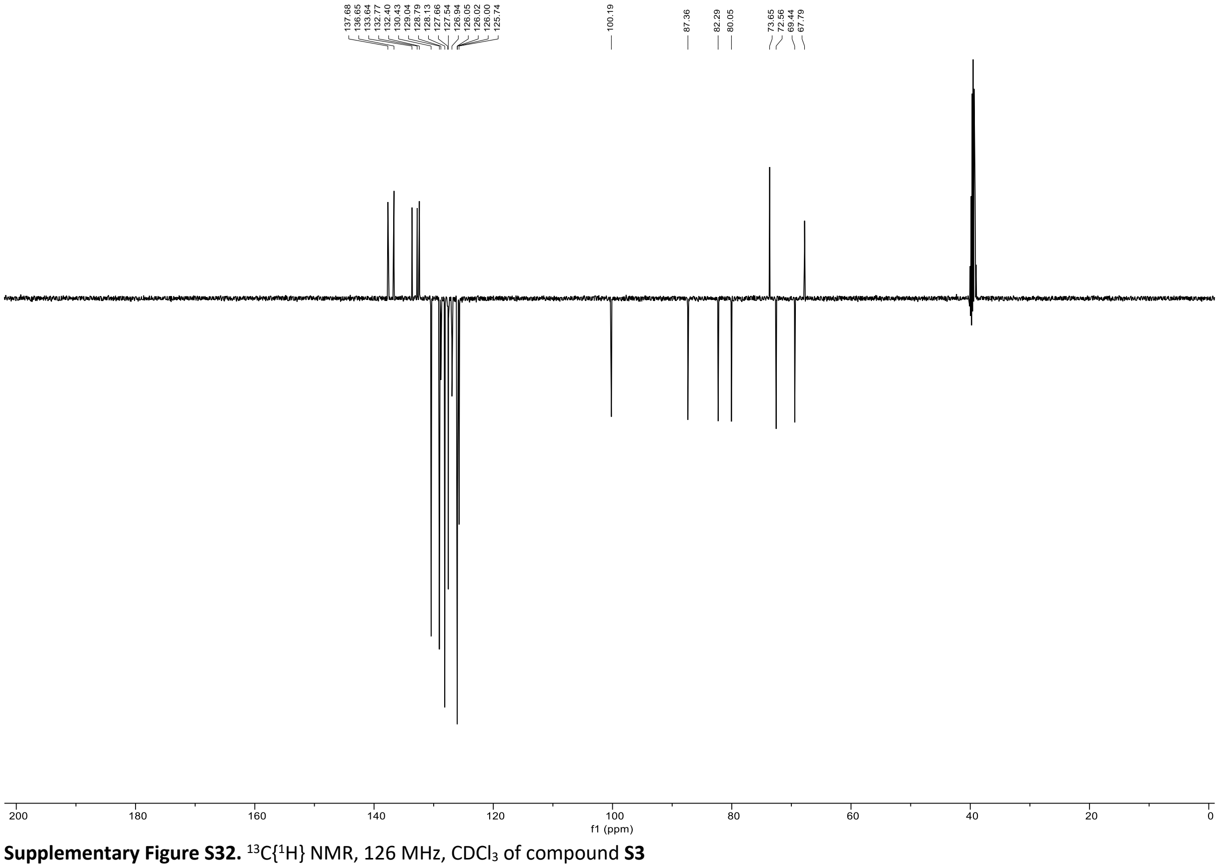

Supplementary Figure S32. $^{13}C\{^{1}H\}$ NMR, 126 MHz, $CDCl_3$ of compound **S3**

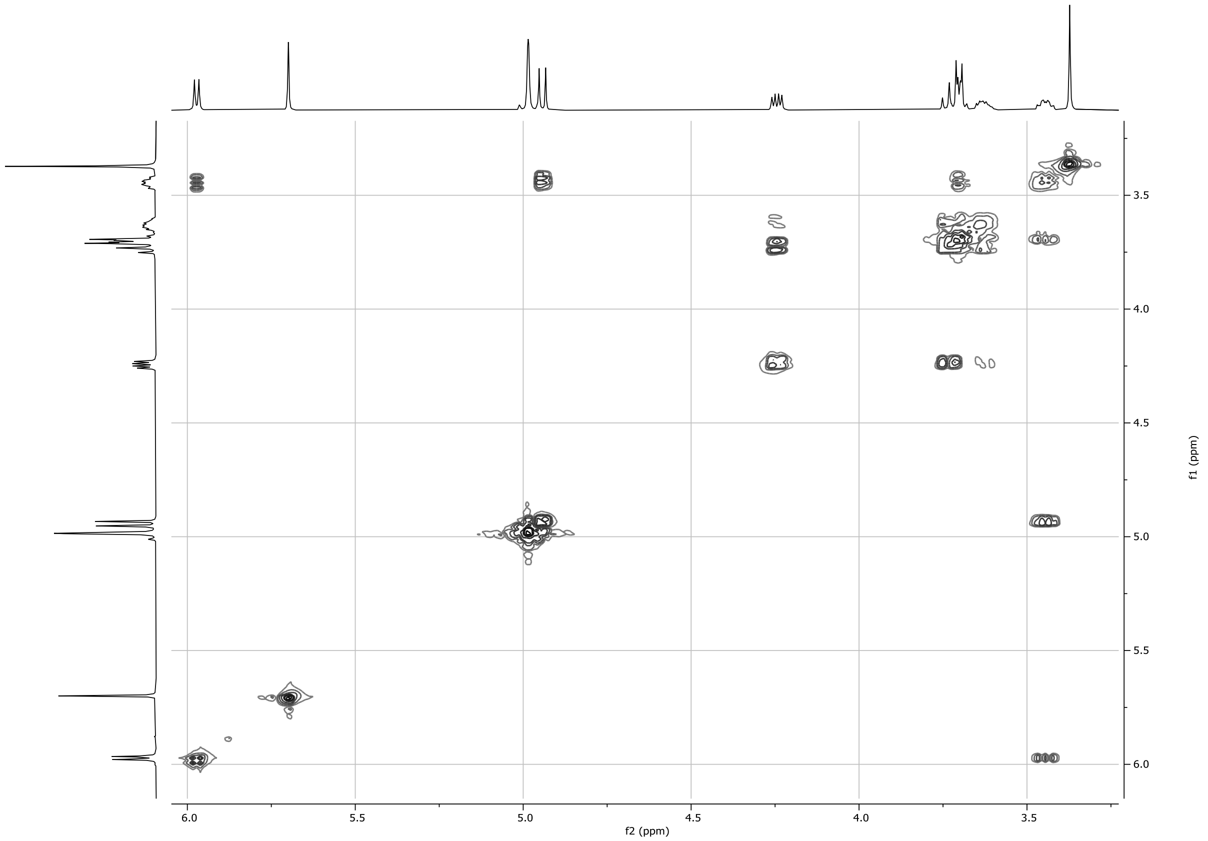

Supplementary Figure S33. HH-COSY NMR, $CDCl_3$ of compound **S3**

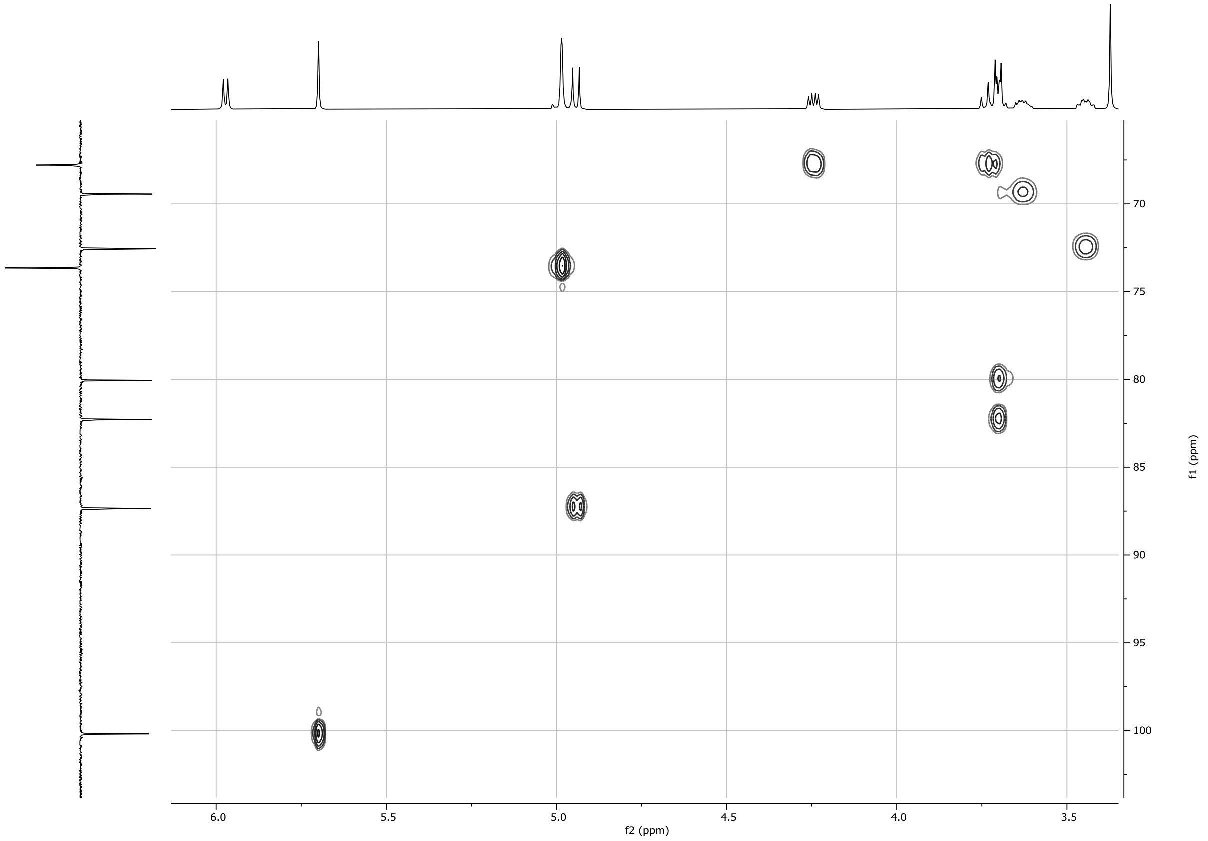

Supplementary Figure S34. HSQC{^{1}H} NMR, $CDCl_3$ of compound **S3**

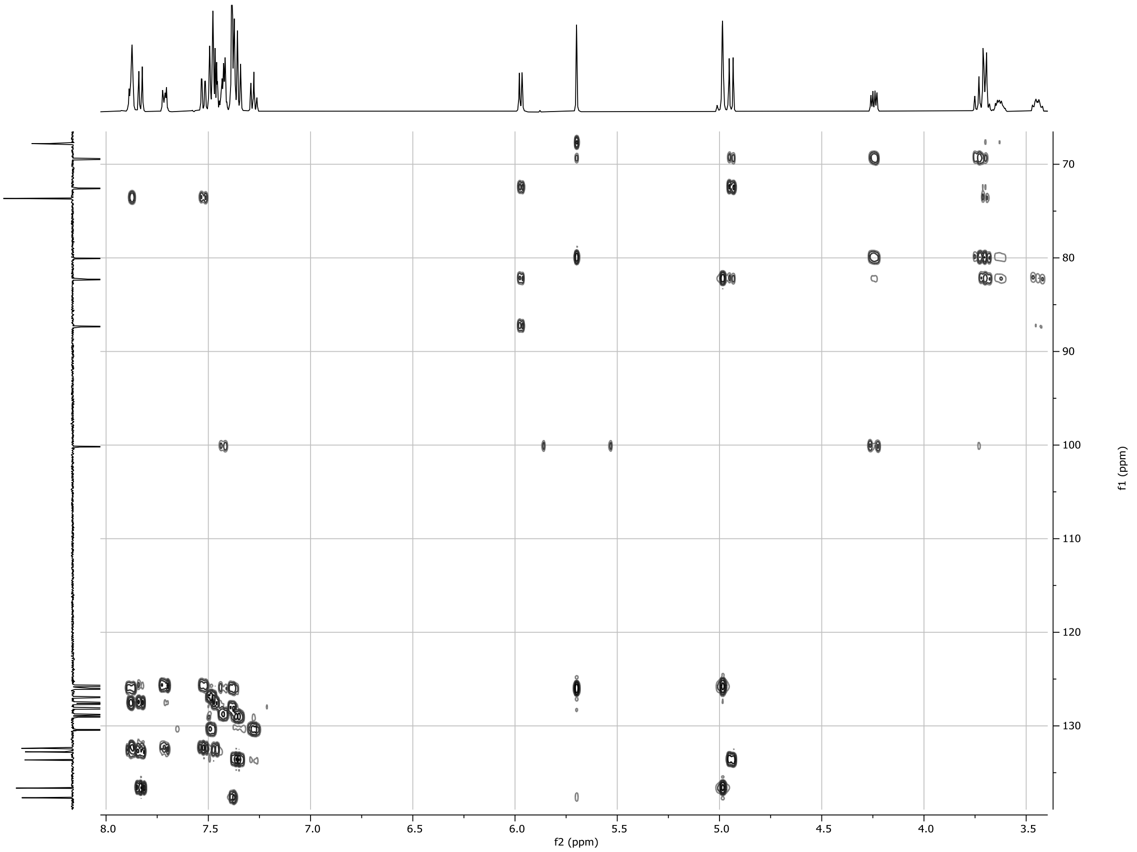

Supplementary Figure S35. HMBC{^{1}H} NMR, $CDCl_3$ of compound **S3**

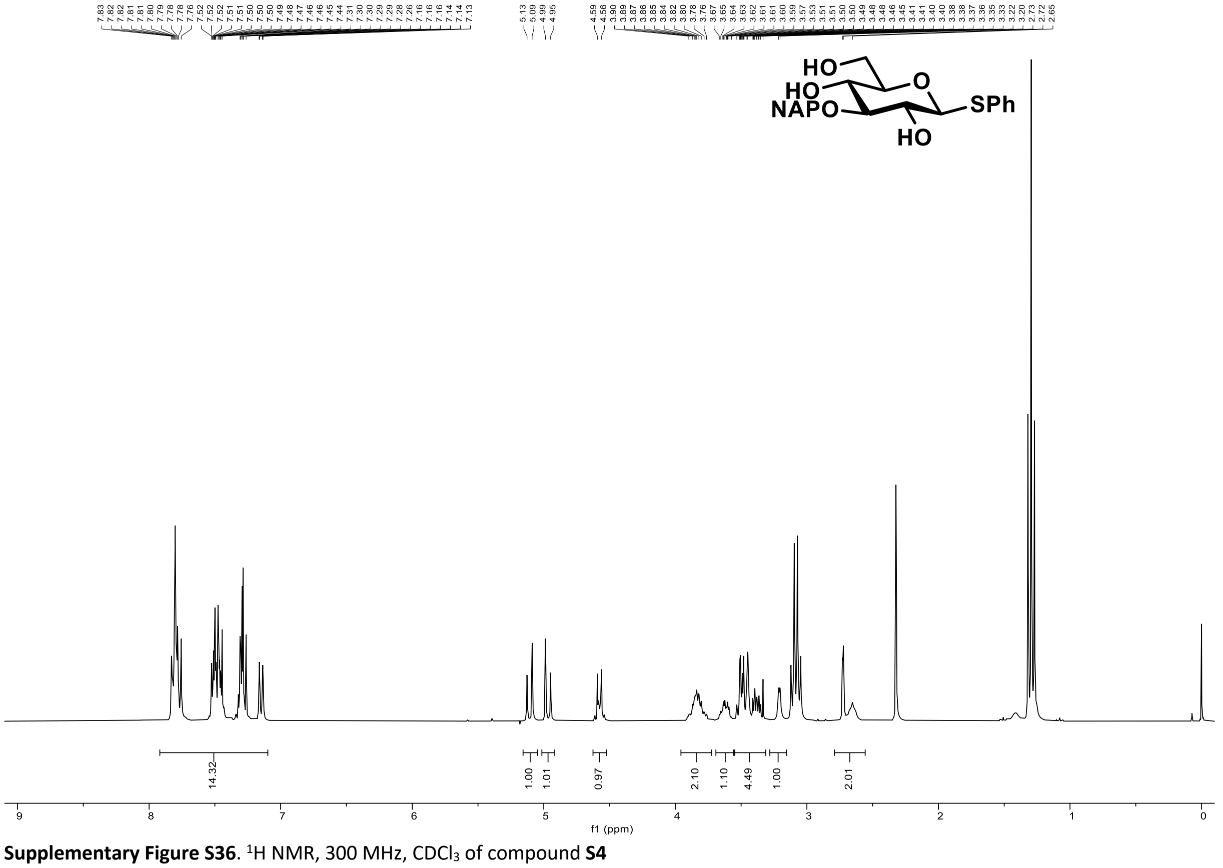

Supplementary Figure S36. ^{1}H NMR, 300 MHz, $CDCl_3$ of compound **S4**

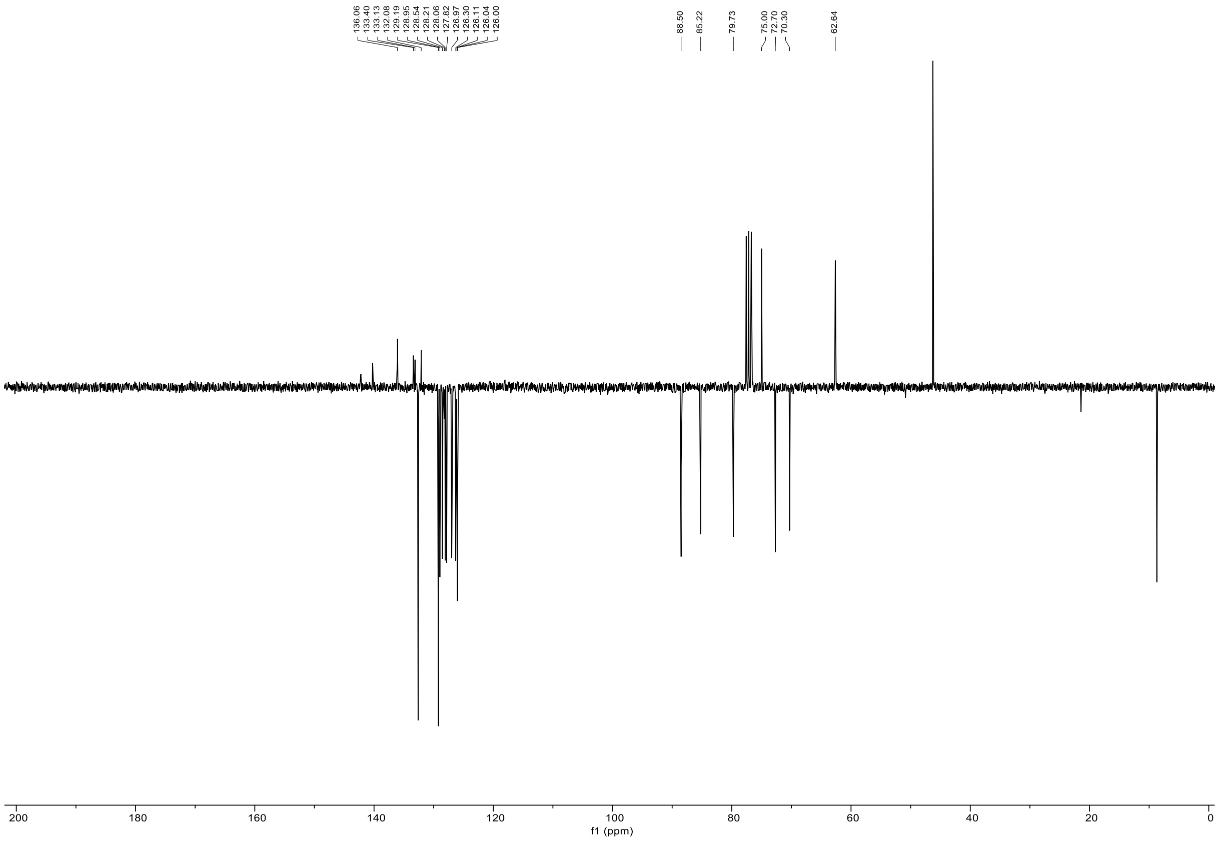

Supplementary Figure S37. $^{13}C\{^{1}H\}$ NMR, 75 MHz, $CDCl_3$ of compound **S4**

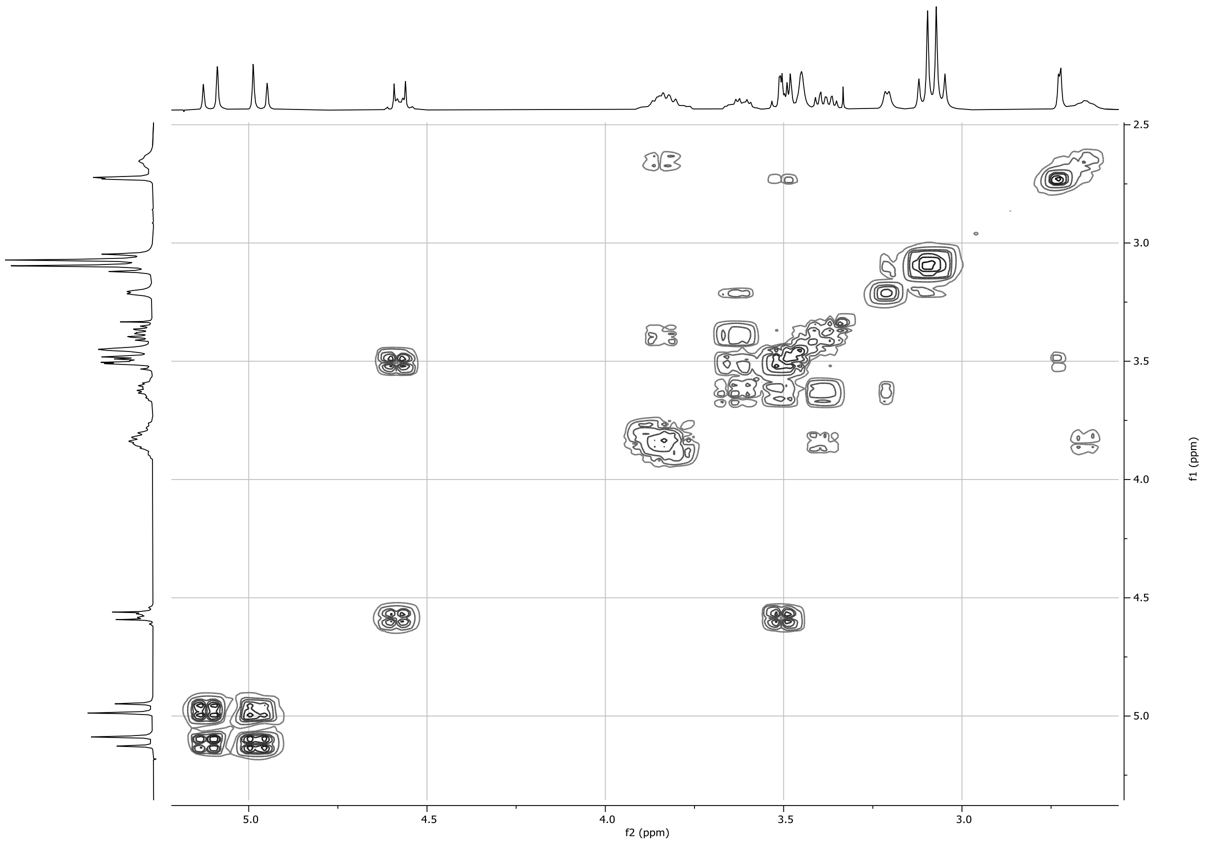

Supplementary Figure S38. HH-COSY NMR, $CDCl_3$ of compound **S4**

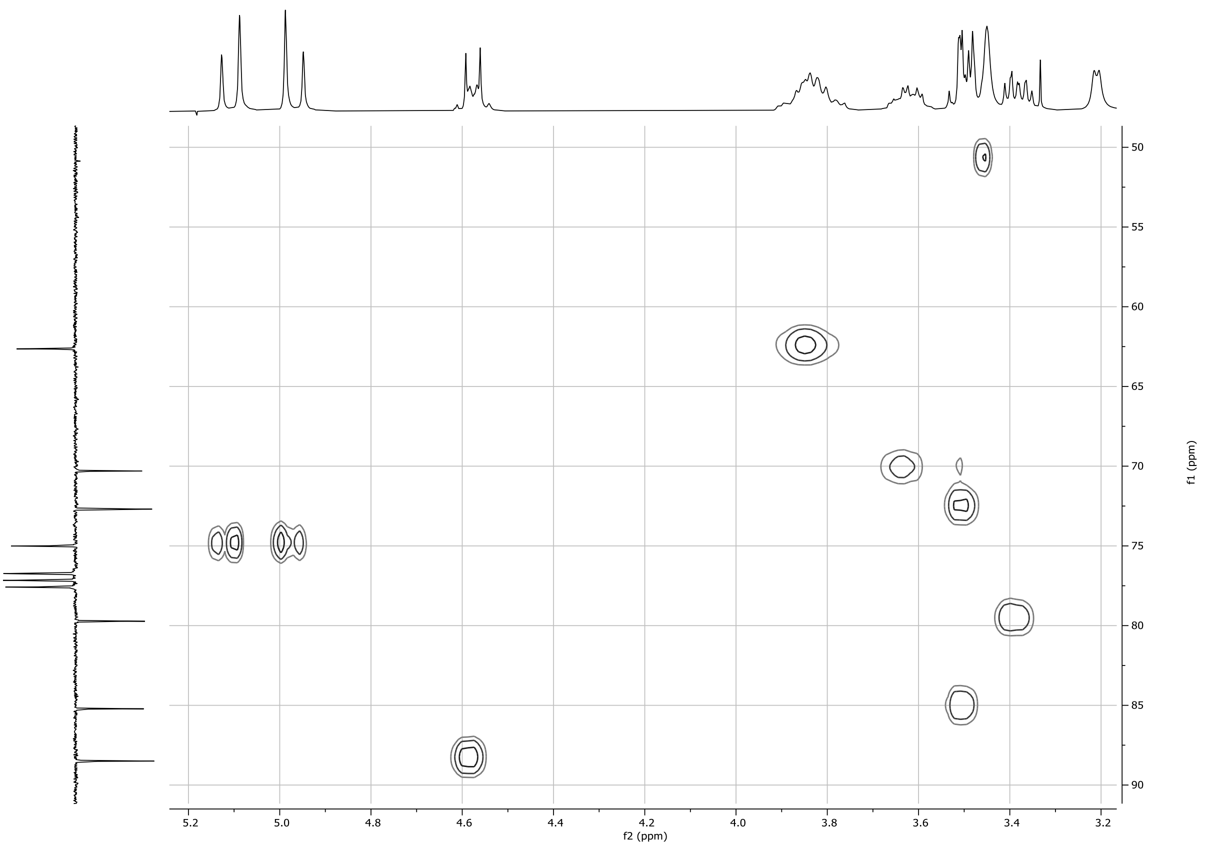

Supplementary Figure S39. HSQC{¹H} NMR, $CDCl_3$ of compound **S4**

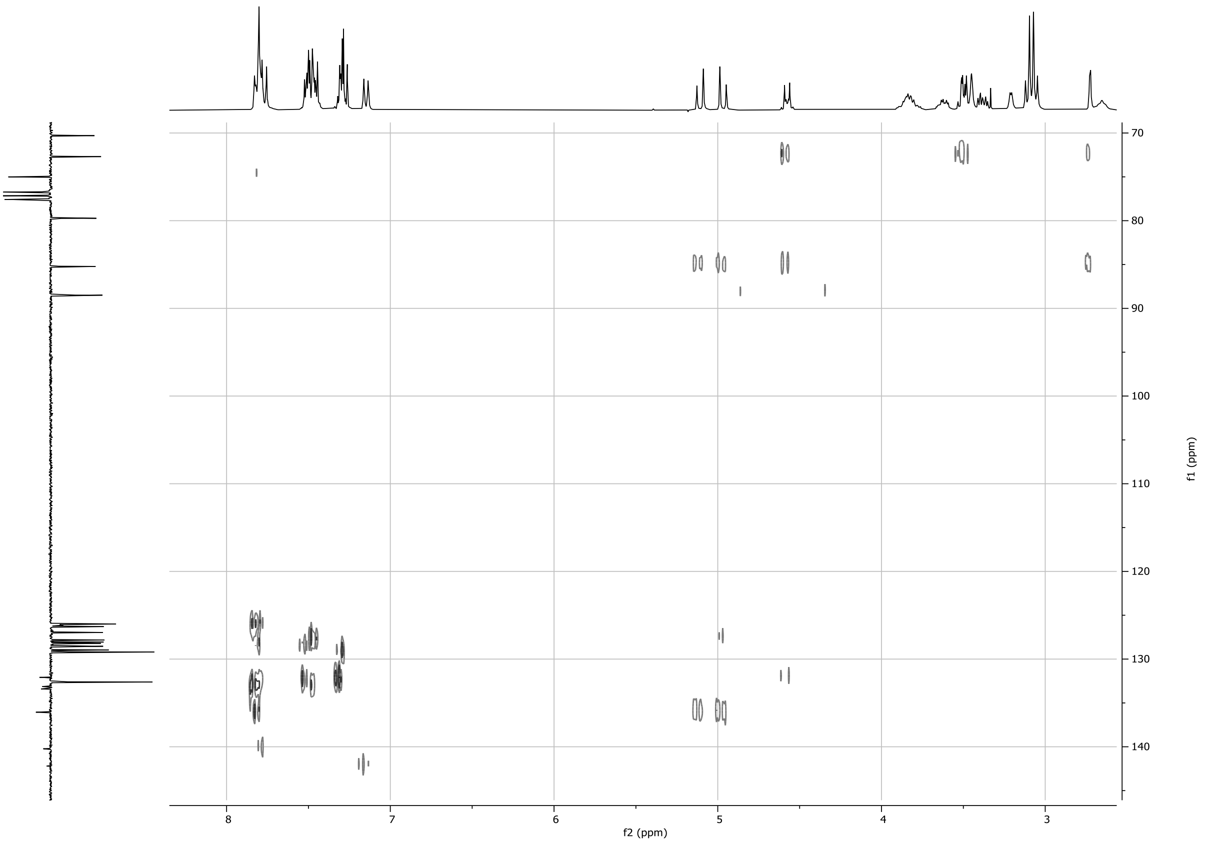

Supplementary Figure S40. HMBC{¹H} NMR, $CDCl_3$ of compound **S4**

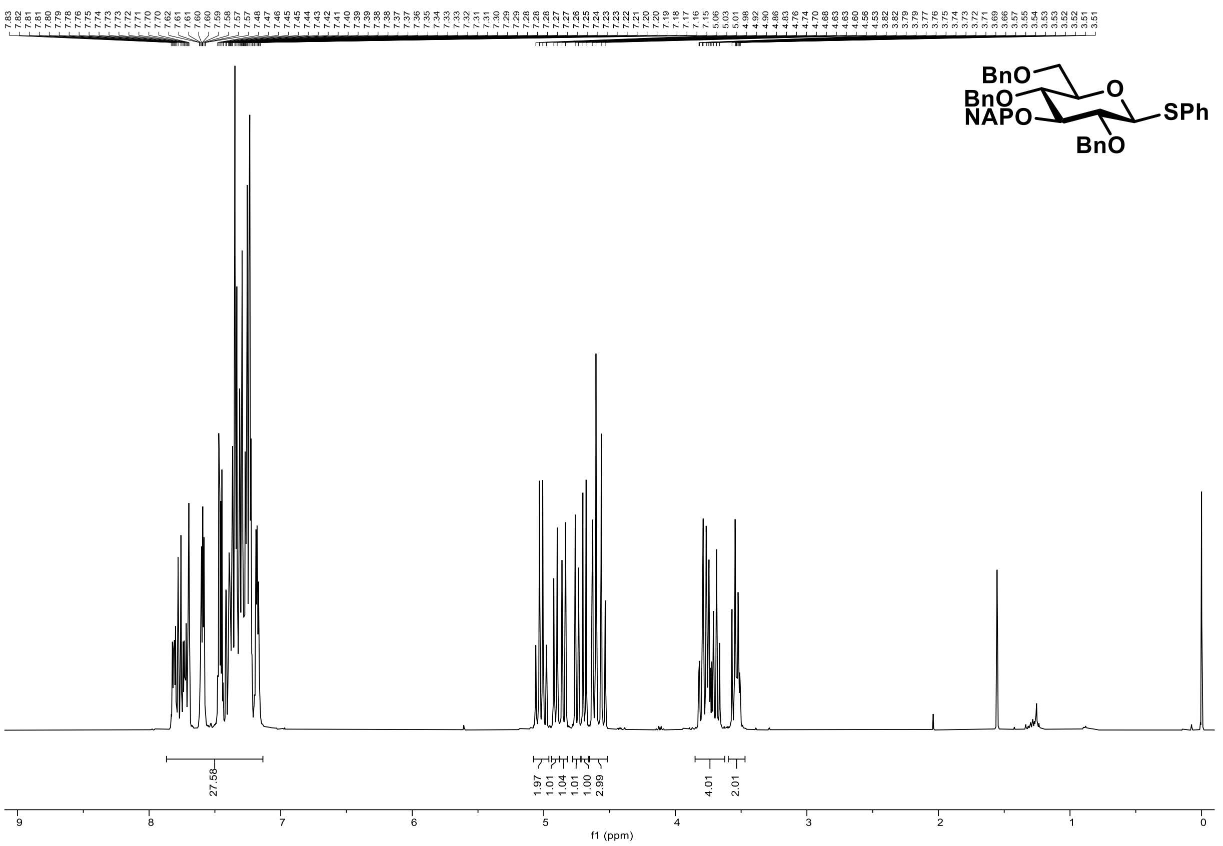

Supplementary Figure S41. ^{1}H NMR, 400 MHz, $CDCl_3$ of compound **S5**

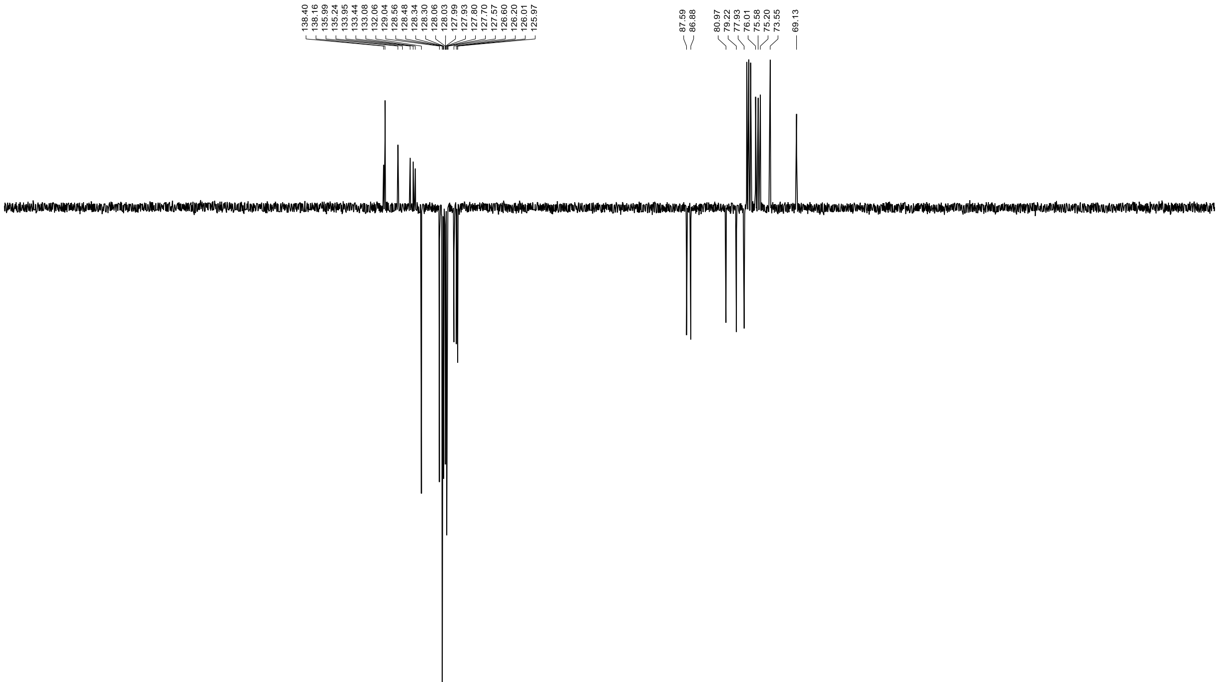

Supplementary Figure S42. $^{13}C\{^{1}H\}$ NMR, 101 MHz, $CDCl_3$ of compound **S5**

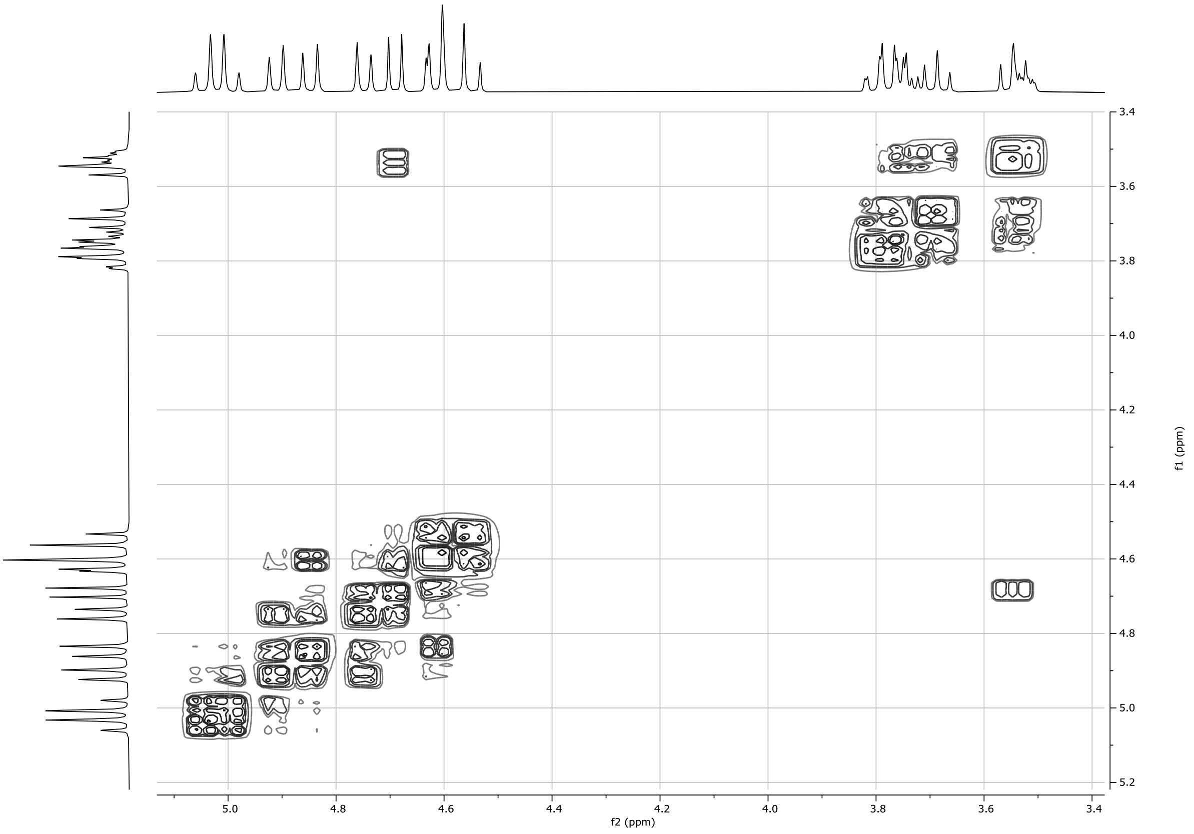

Supplementary Figure S43. HH-COSY NMR, $CDCl_3$ of compound **S5**

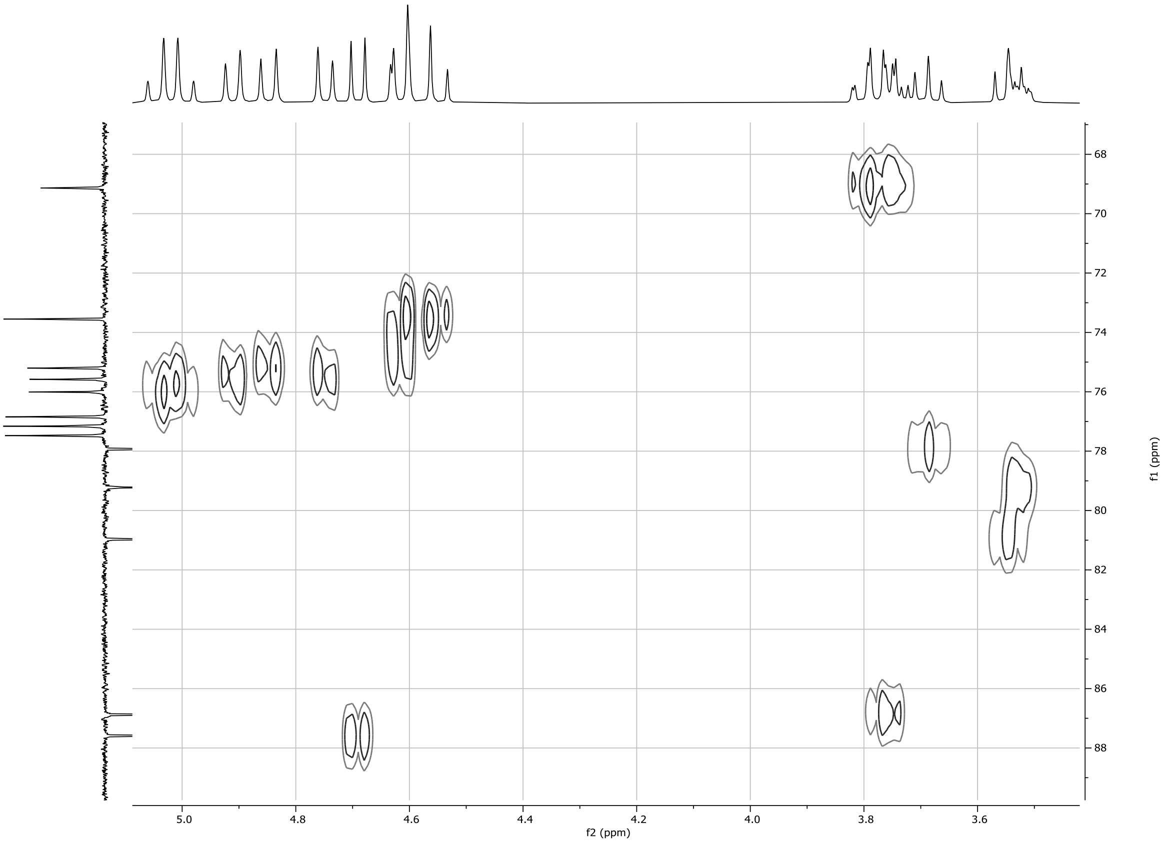

Supplementary Figure S44. HSQC{^{1}H} NMR, $CDCl_3$ of compound **S5**

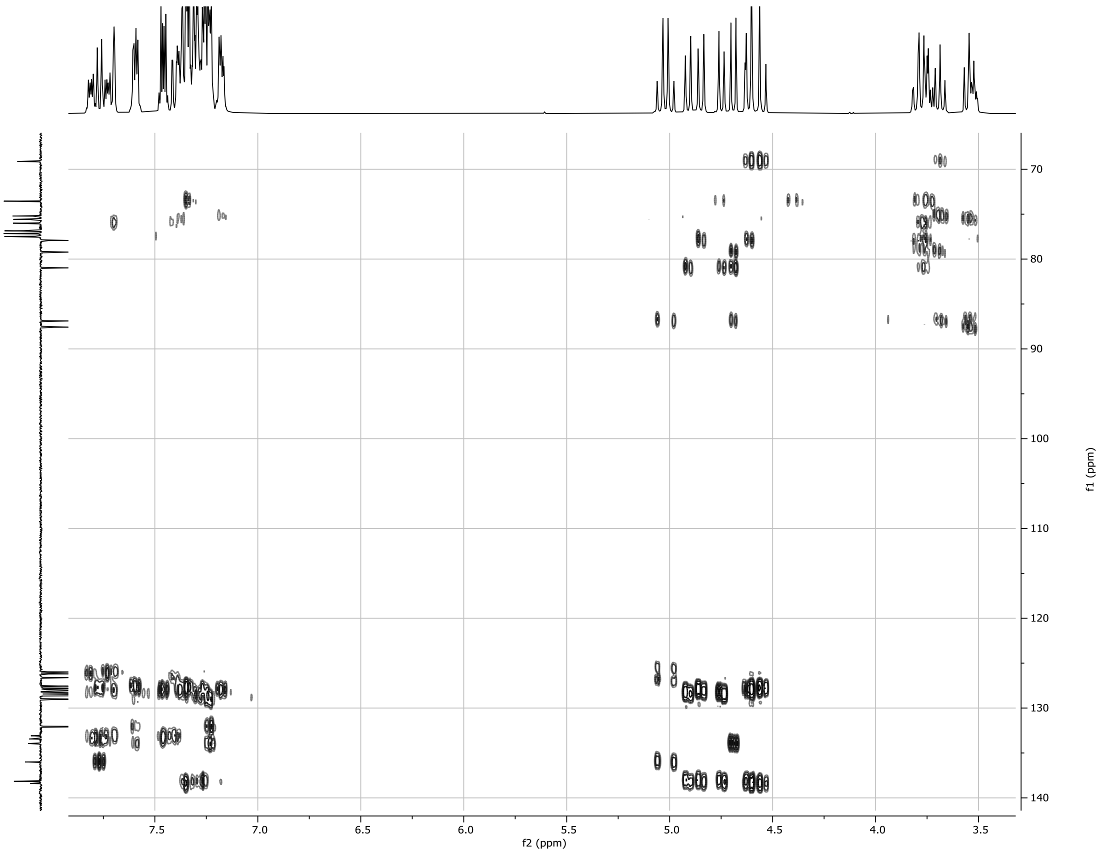

Supplementary Figure S45. HMBC{^{1}H} NMR, $CDCl_3$ of compound **S5**

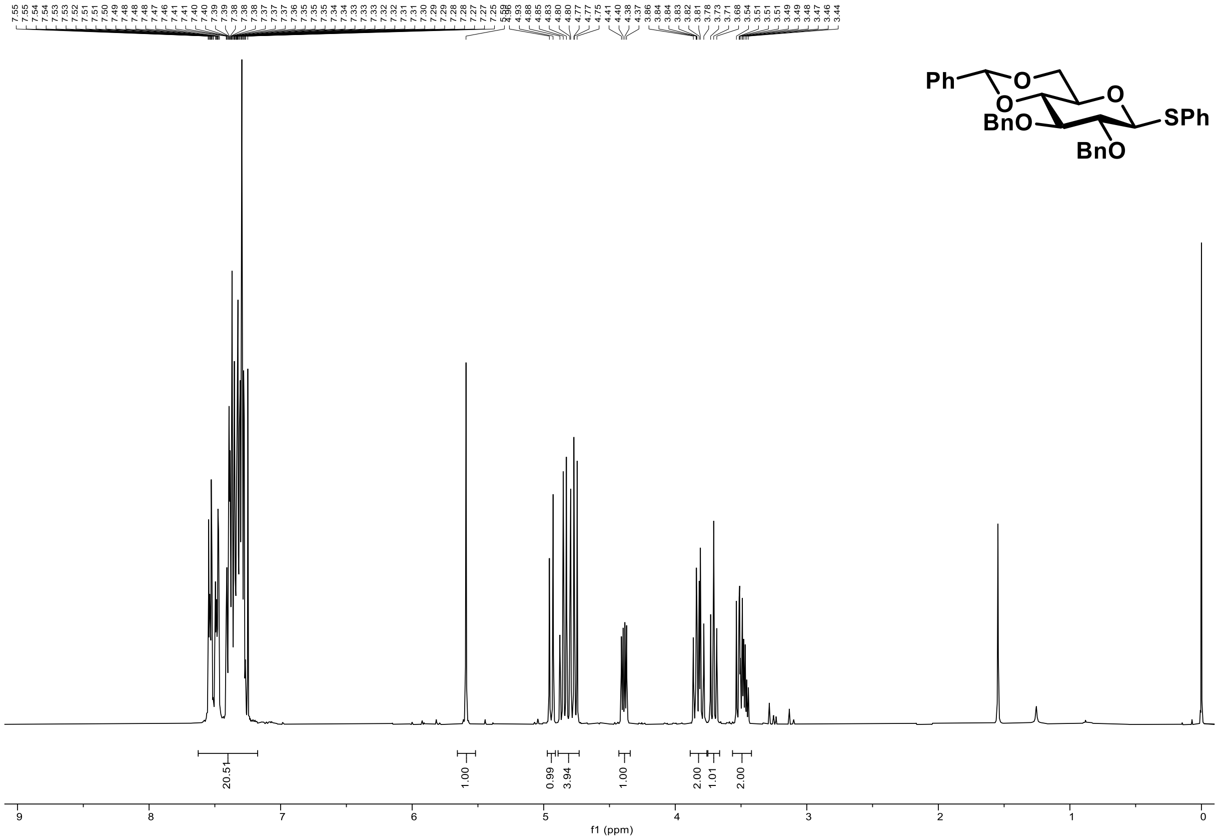

Supplementary Figure S46. ^{1}H NMR, 400 MHz, $CDCl_3$ of compound **S6**

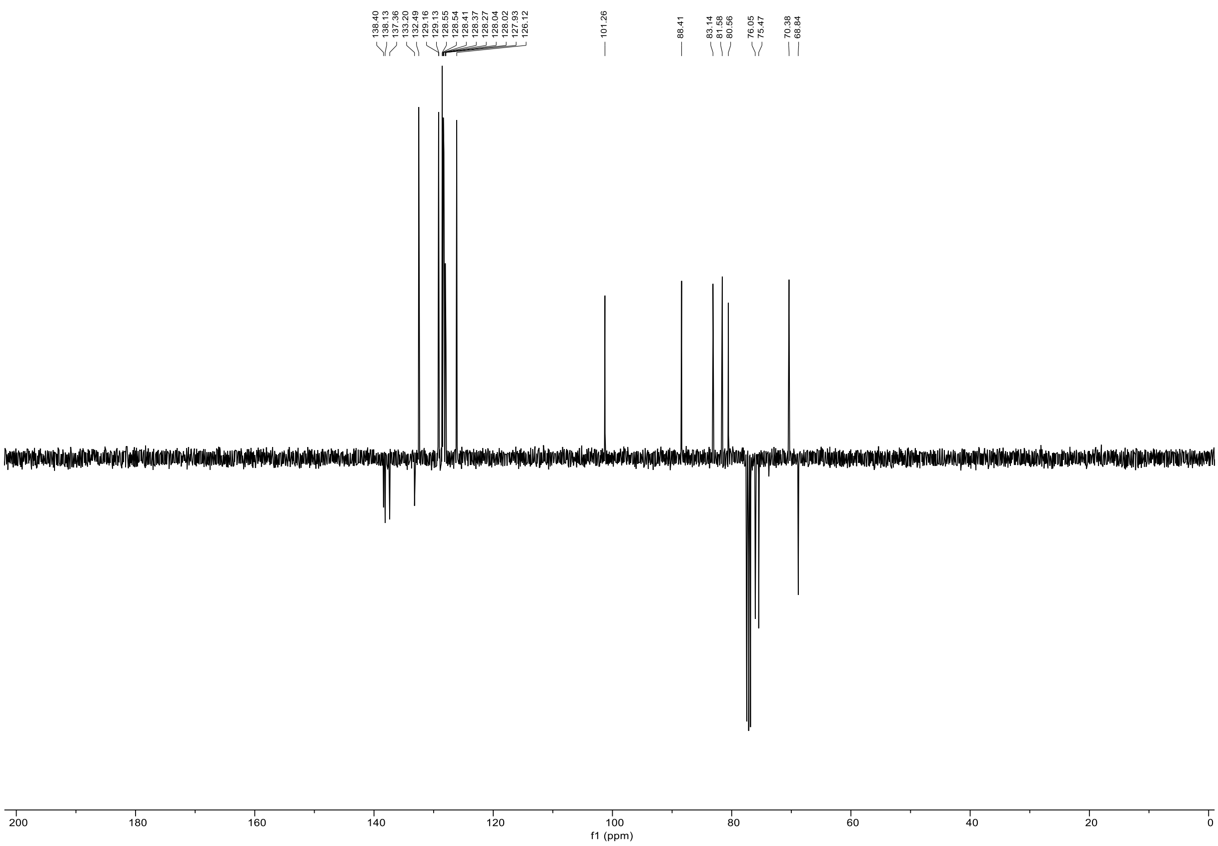

Supplementary Figure S47. $^{13}C\{^{1}H\}$ NMR, 101 MHz, $CDCl_3$ of compound **S6**

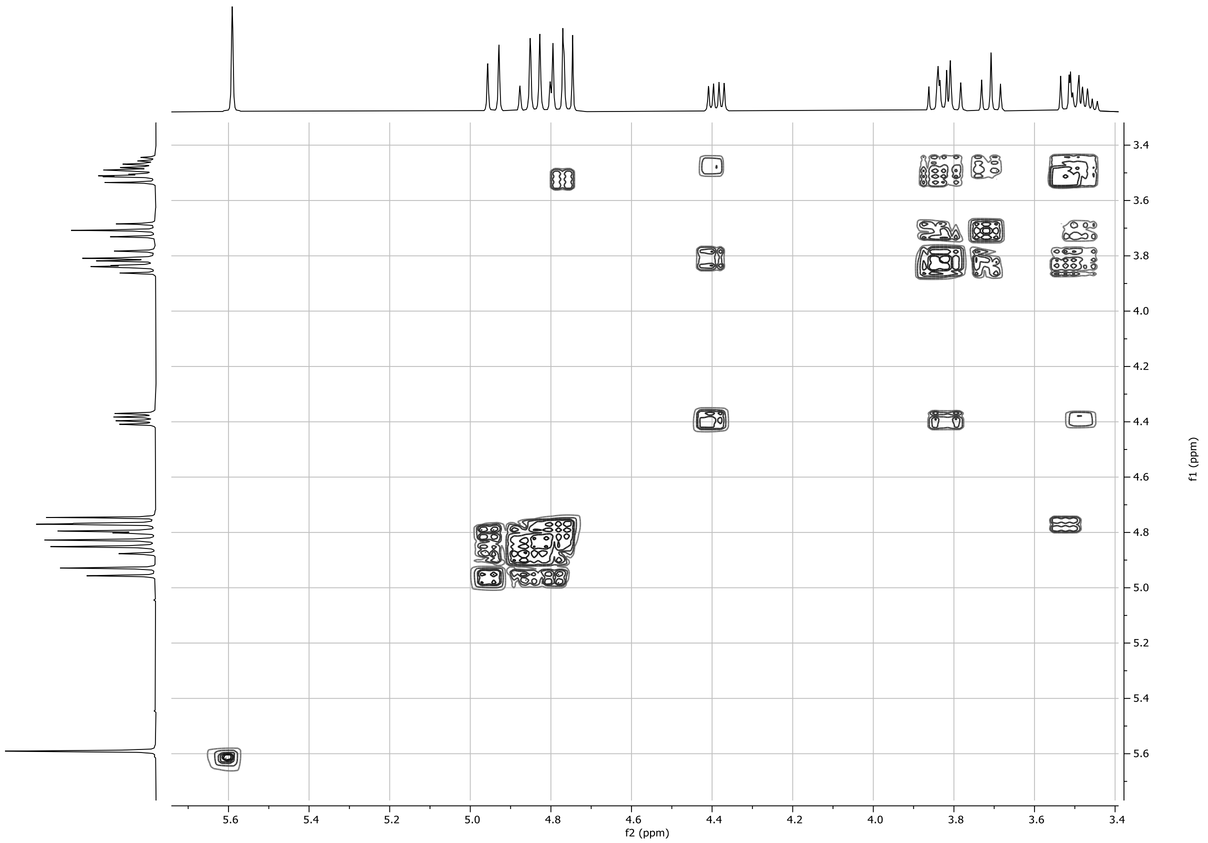

Supplementary Figure S48. HH-COSY NMR, $CDCl_3$ of compound **S6**

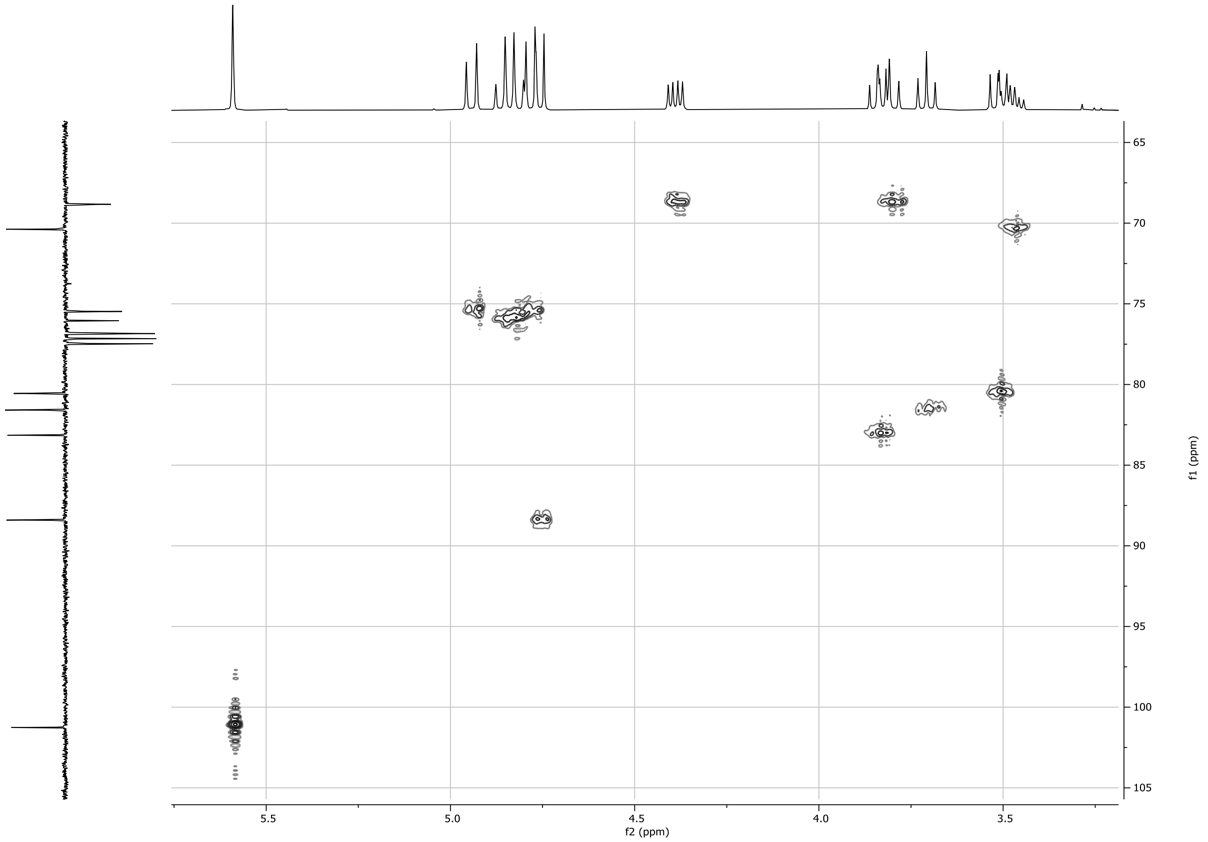

Supplementary Figure S49. HSQC{¹H} NMR, $CDCl_3$ of compound **S6**

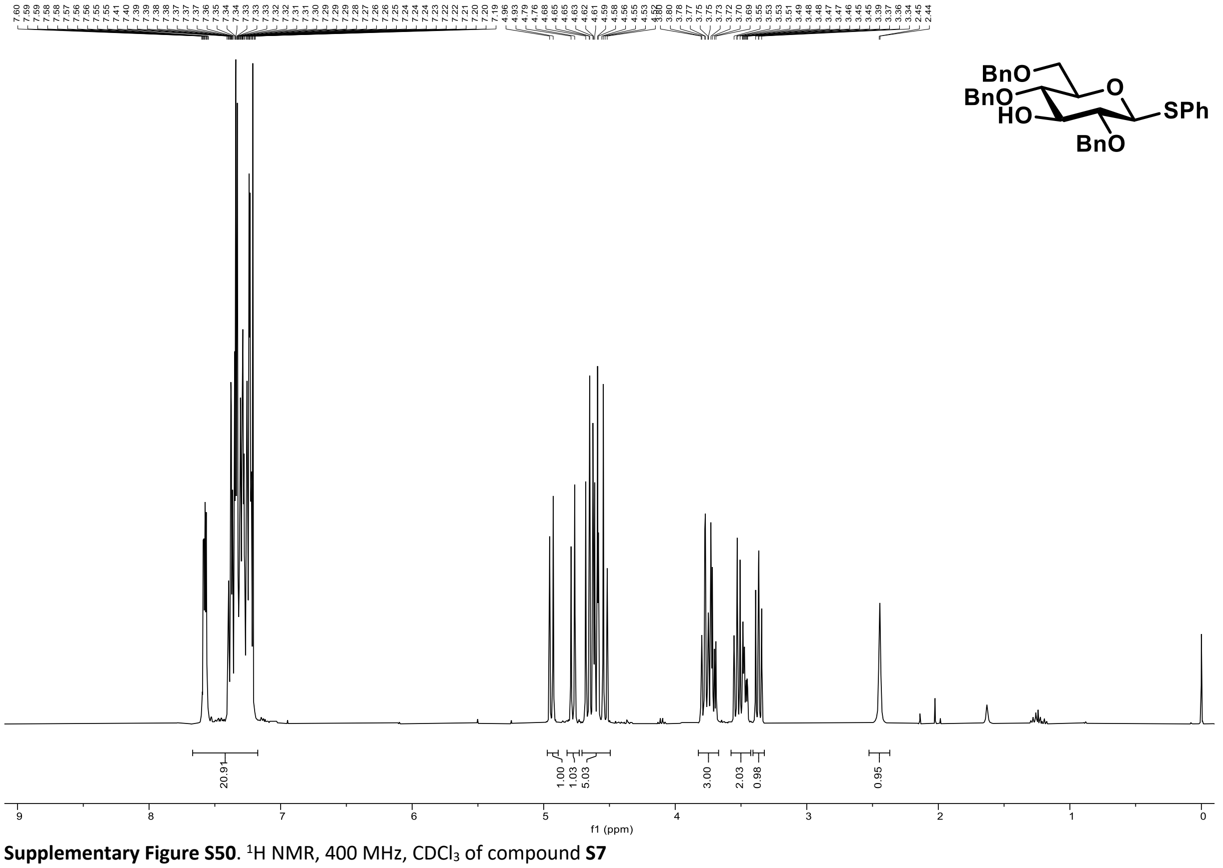

Supplementary Figure S50. ¹H NMR, 400 MHz, $CDCl_3$ of compound **S7**

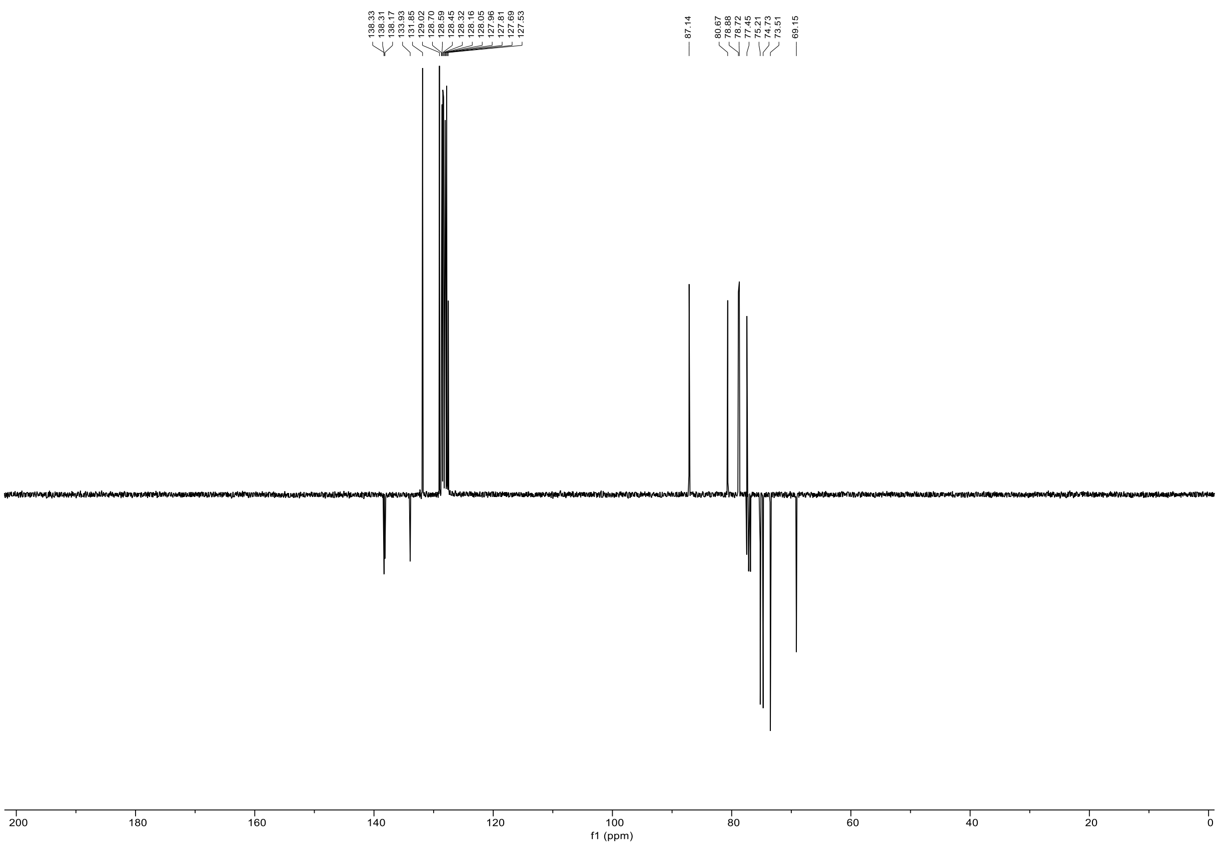

Supplementary Figure S51. $^{13}C\{^{1}H\}$ NMR, 101 MHz, $CDCl_3$ of compound **S7**

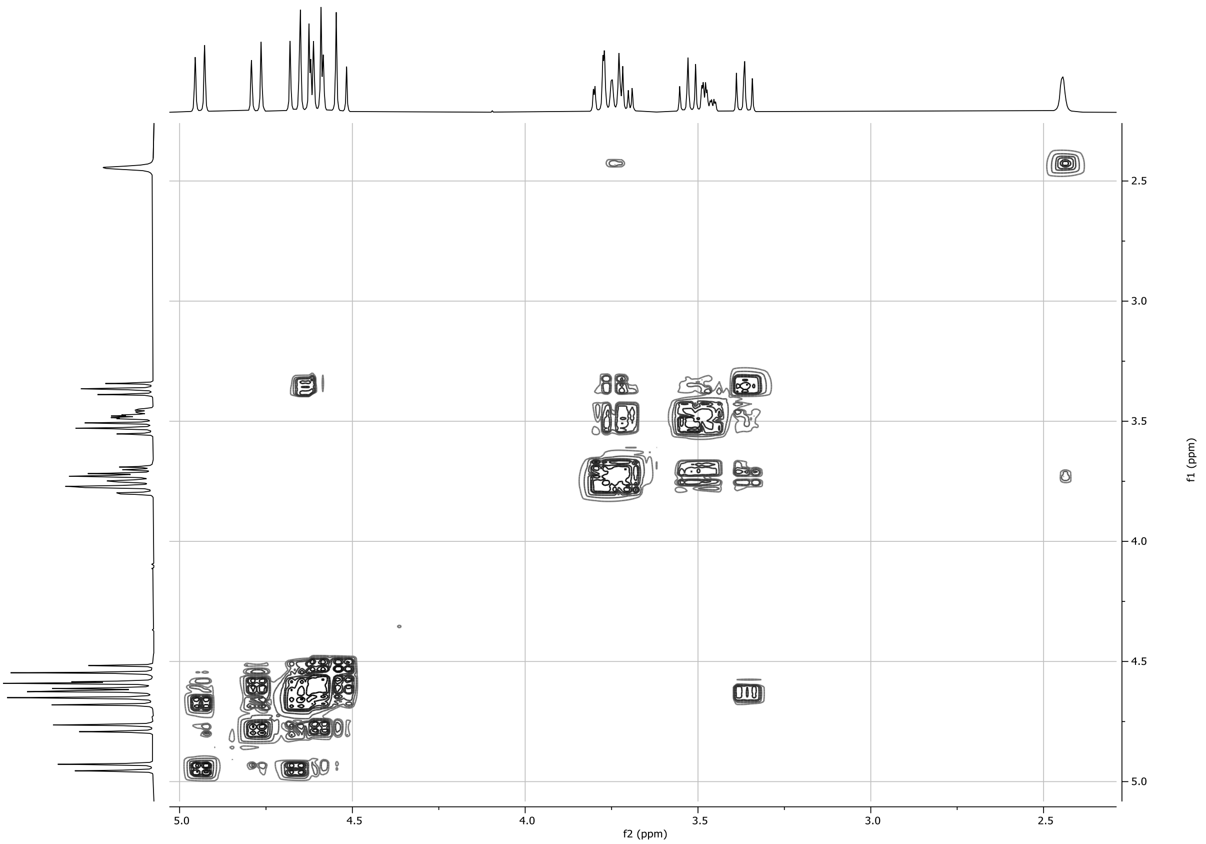

Supplementary Figure S52. HH-COSY NMR, $CDCl_3$ of compound **S7**

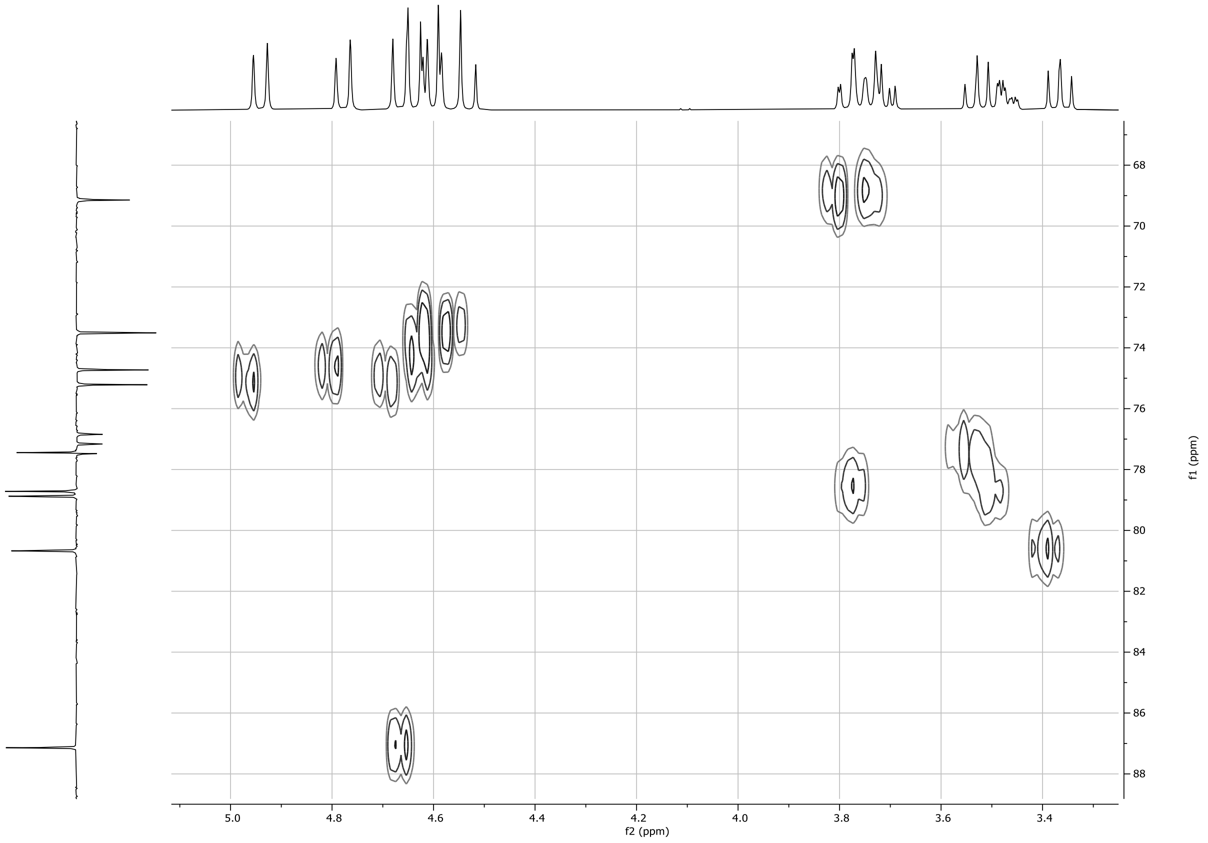

Supplementary Figure S53. HSQC{^{1}H} NMR, $CDCl_3$ of compound **S7**

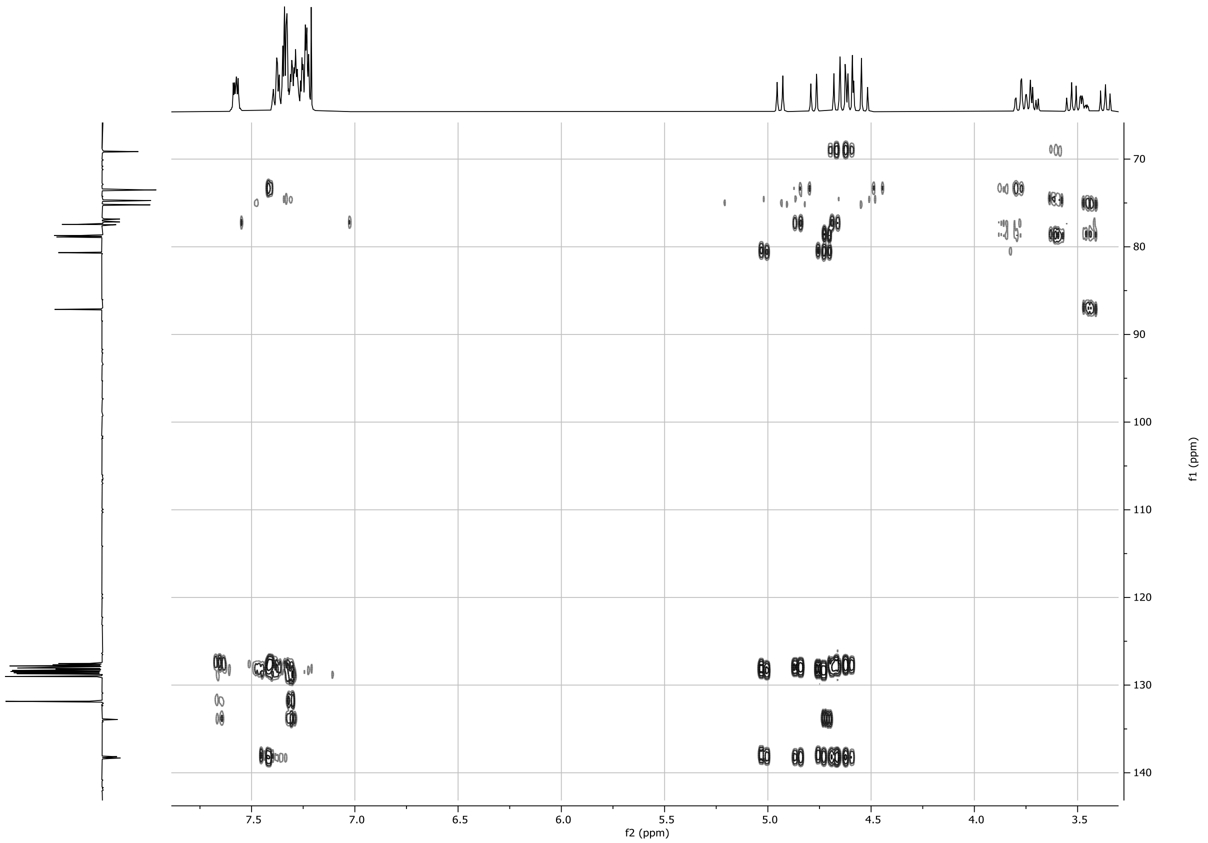

Supplementary Figure S54. HMBC{^{1}H} NMR, $CDCl_3$ of compound **S7**

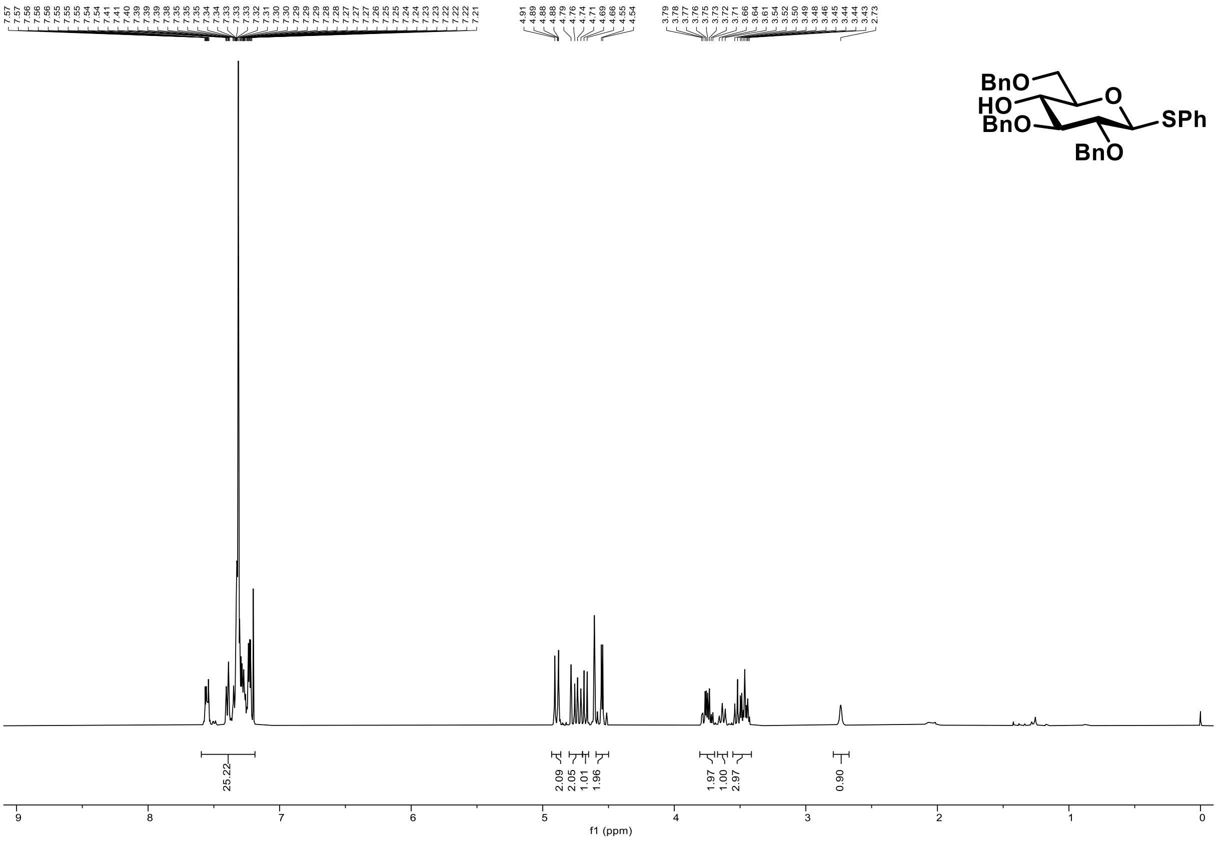

Supplementary Figure S55. ^{1}H NMR, 400 MHz, $CDCl_3$ of compound **S8**

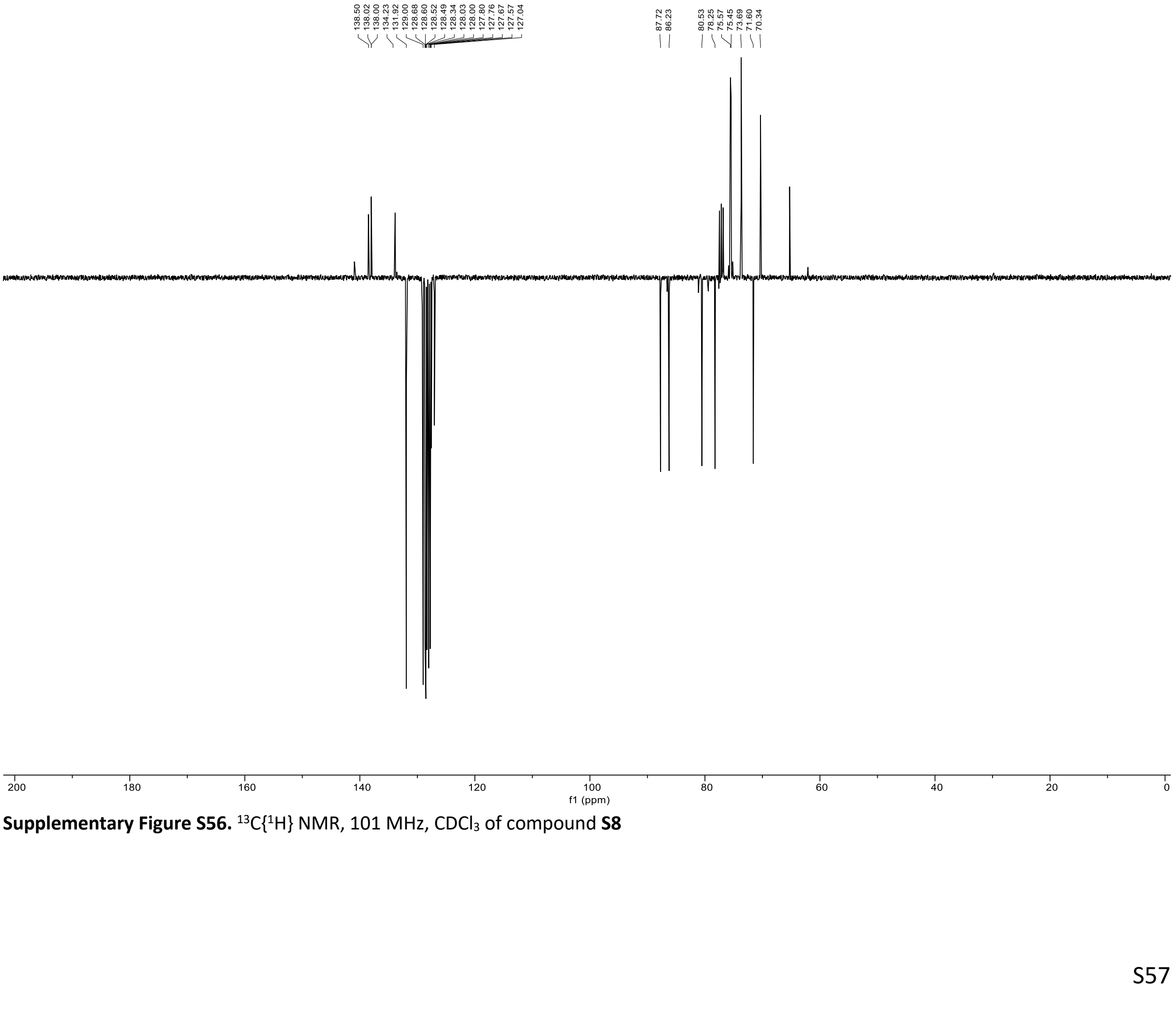

Supplementary Figure S56. $^{13}C\{^{1}H\}$ NMR, 101 MHz, $CDCl_3$ of compound **S8**

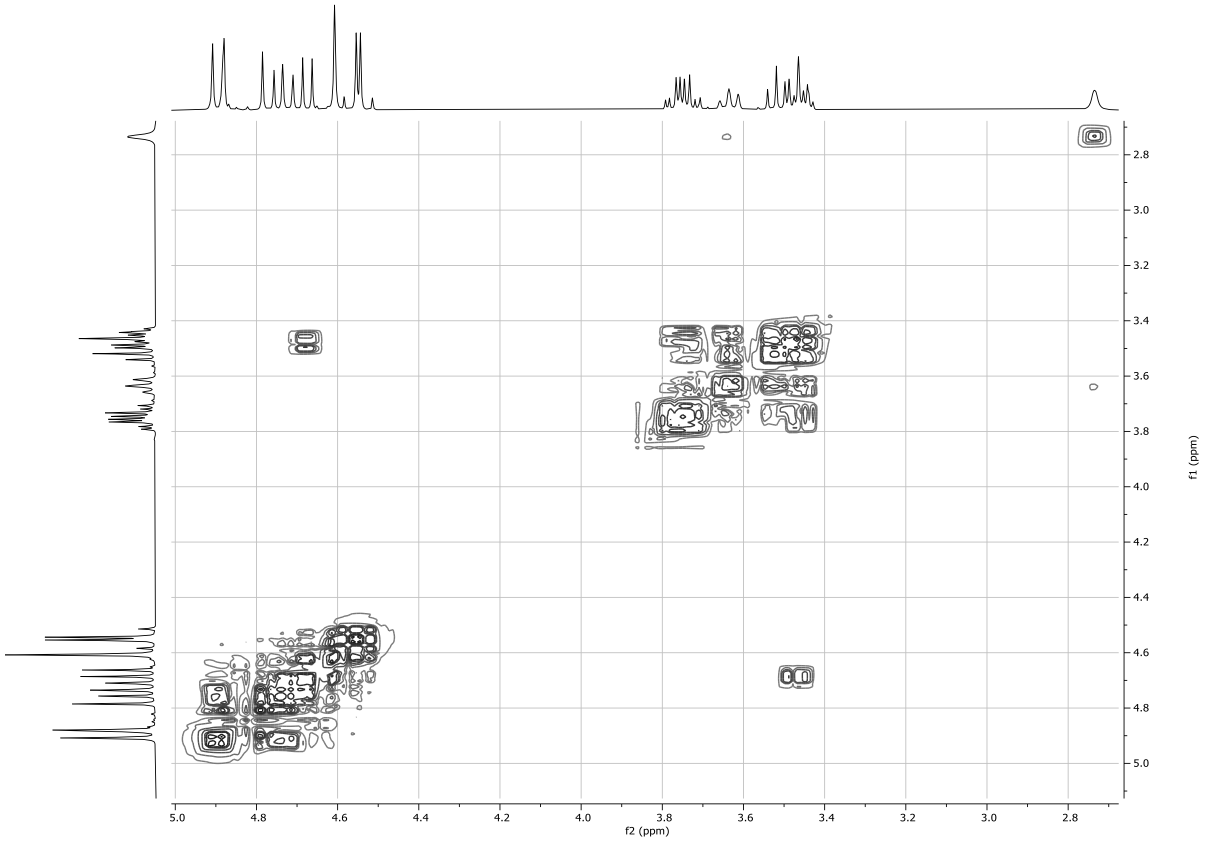

Supplementary Figure S57. HH-COSY NMR, $CDCl_3$ of compound **S8**

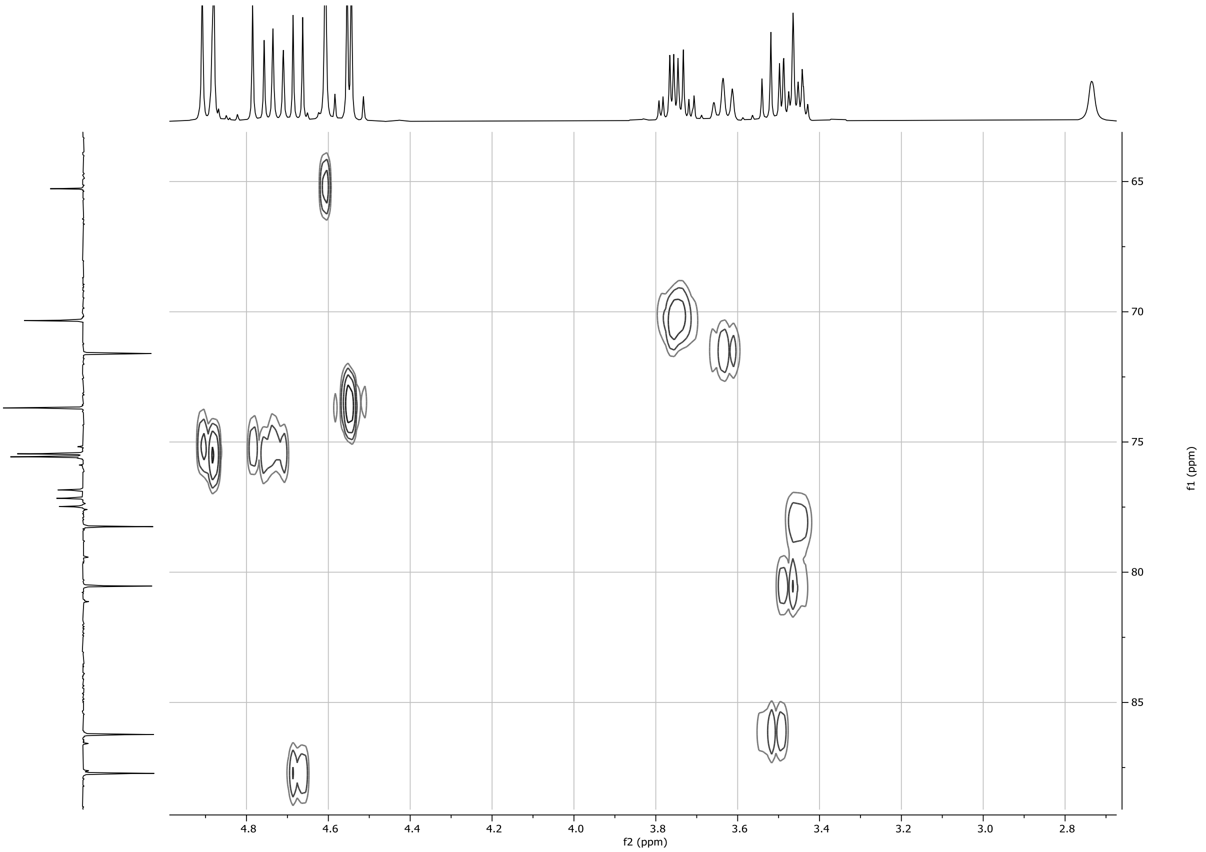

Supplementary Figure S58. HSQC{^{1}H} NMR, $CDCl_3$ of compound **S8**

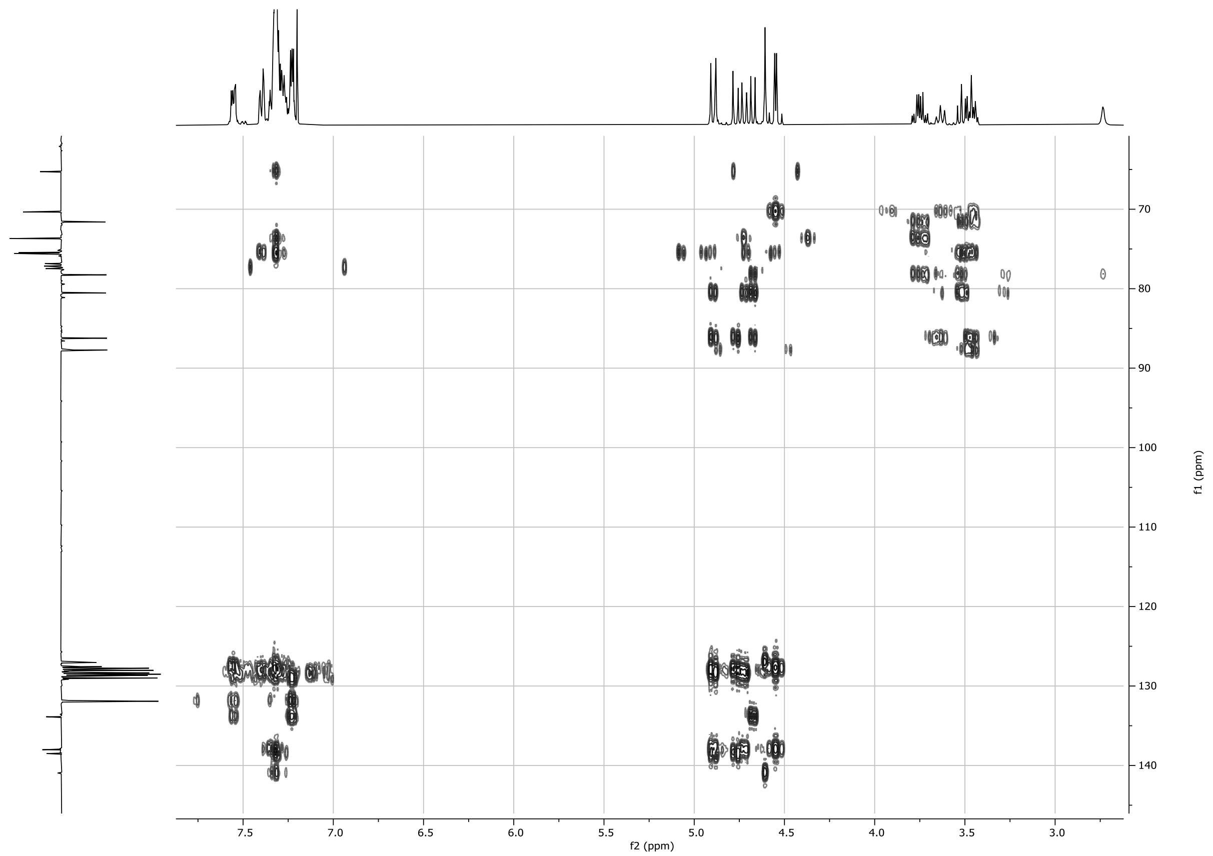

Supplementary Figure S59. HMBC{^{1}H} NMR, $CDCl_3$ of compound **S8**

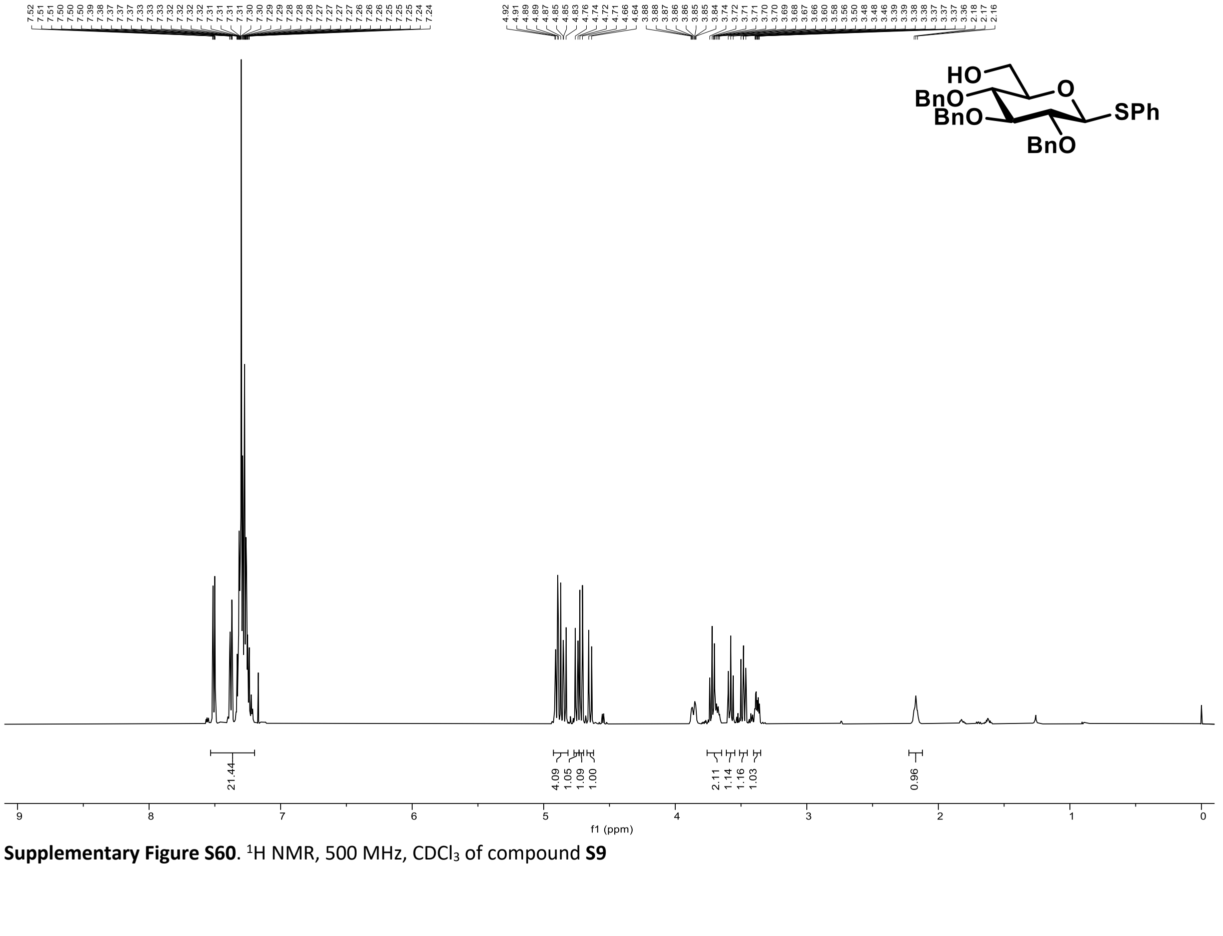

Supplementary Figure S60. ^{1}H NMR, 500 MHz, $CDCl_3$ of compound **S9**

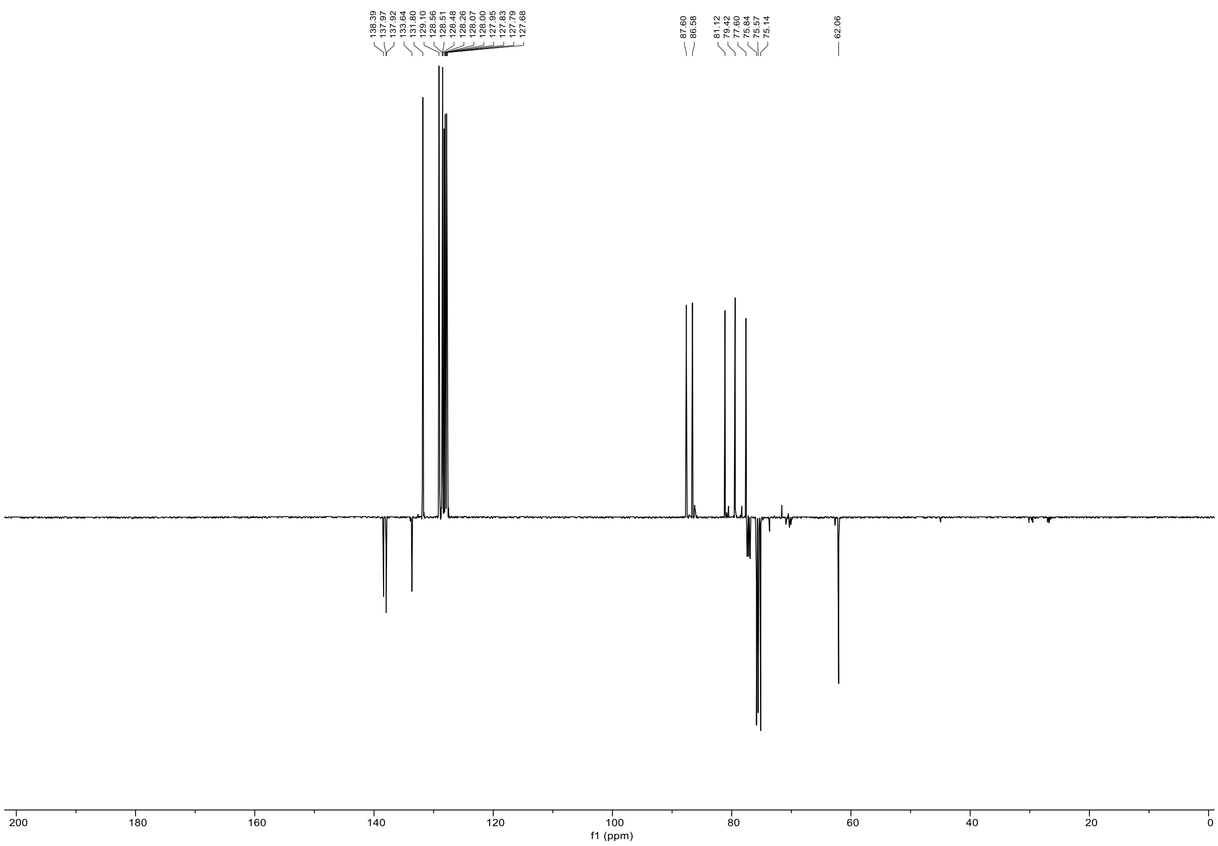

Supplementary Figure S61. $^{13}C\{^1H\}$ NMR, 126 MHz, $CDCl_3$ of compound **S9**

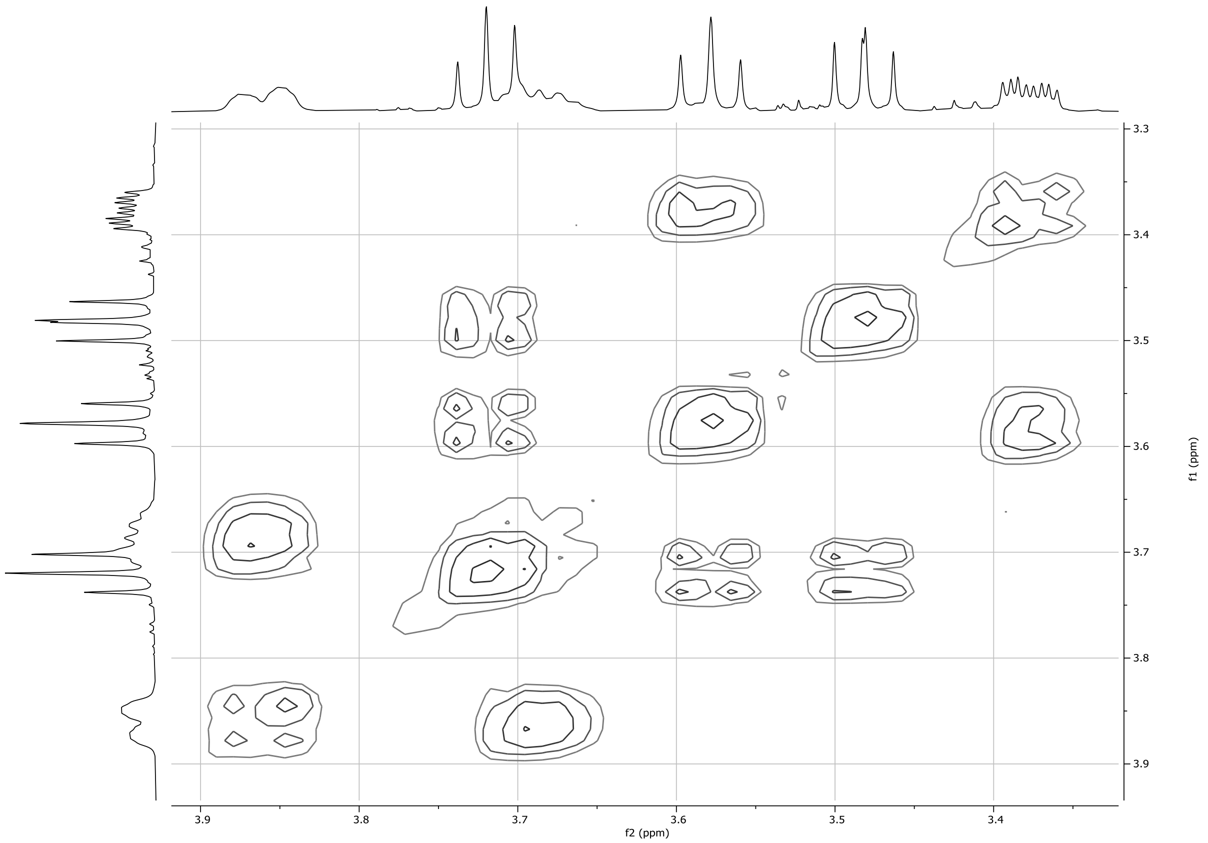

Supplementary Figure S62. HH-COSY NMR, $CDCl_3$ of compound **S9**

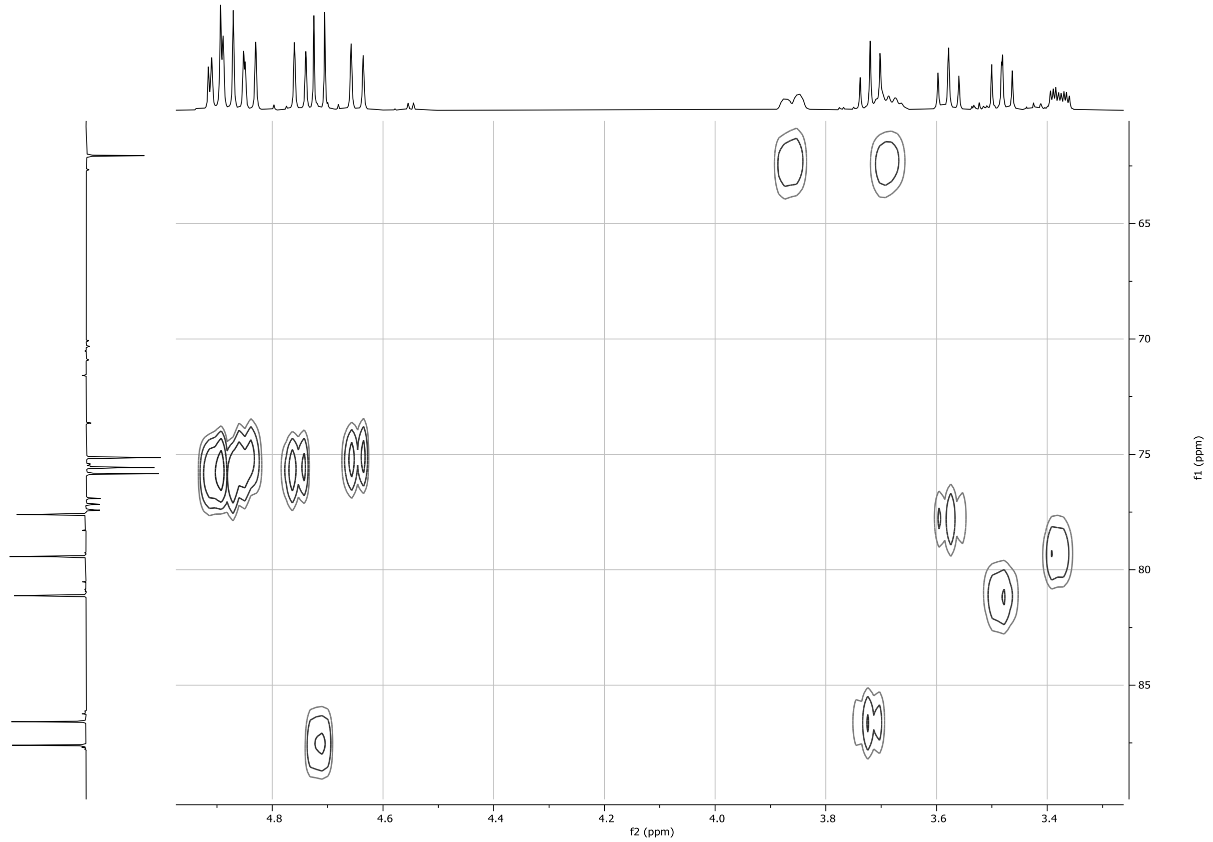

Supplementary Figure S63. HSQC{^{1}H} NMR, $CDCl_3$ of compound **S9**

Methylated donor intermediates NMR spectra

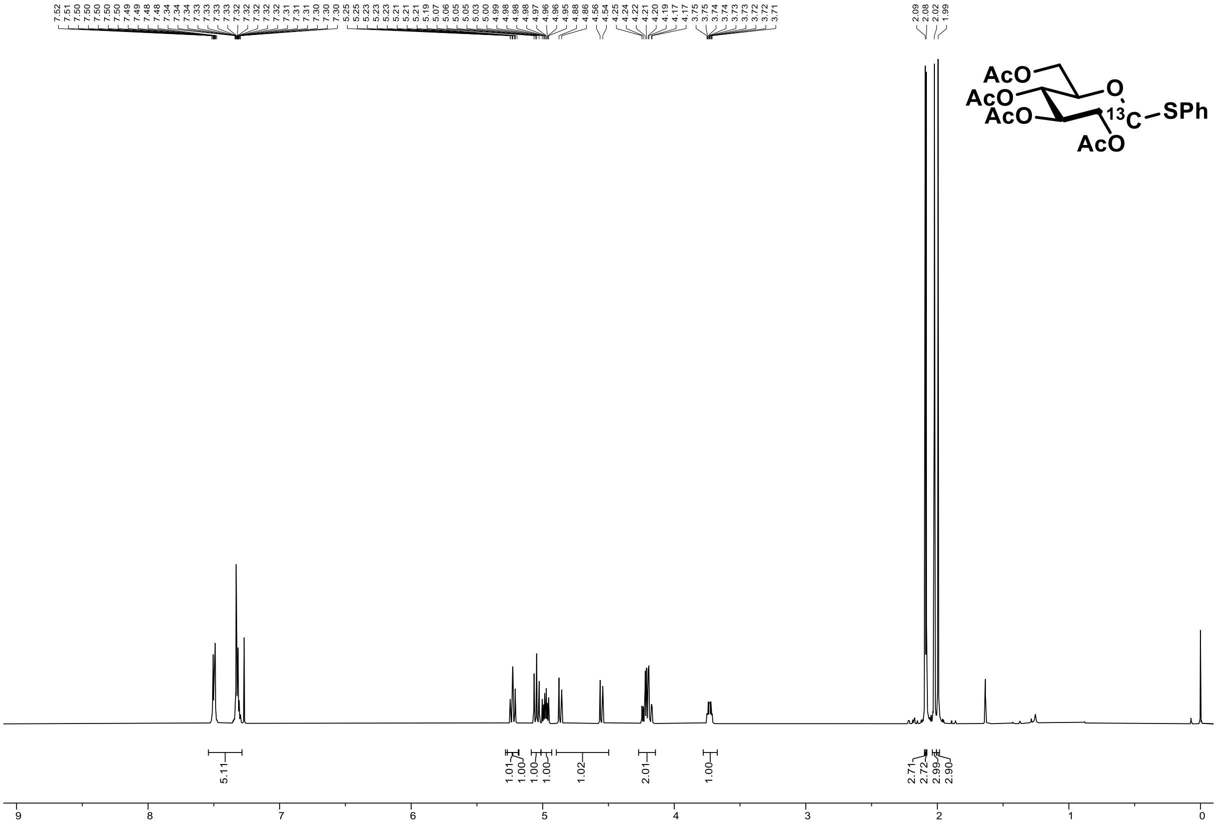

Supplementary Figure S64. ^{1}H NMR, 500 MHz, $CDCl_3$ of compound **S10**

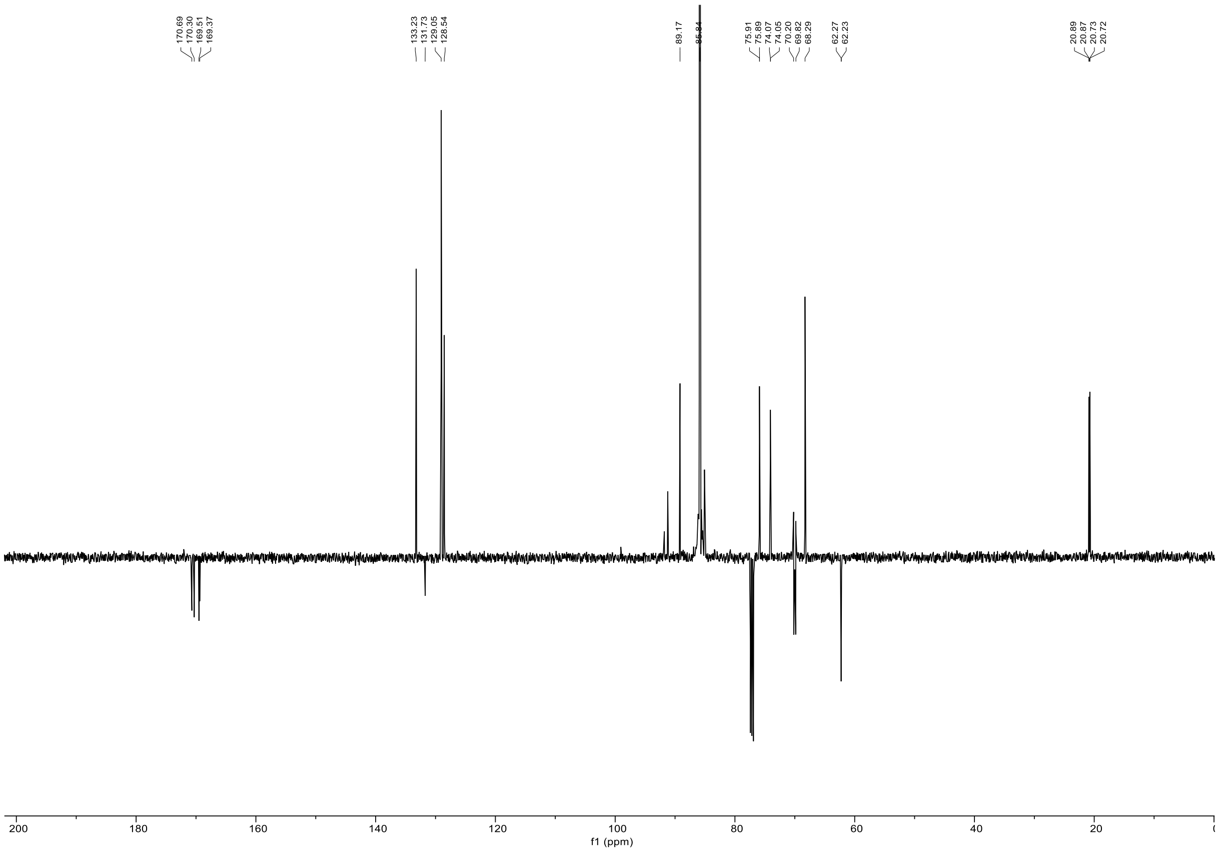

Supplementary Figure S65. $^{13}C\{^{1}H\}$ NMR, 126 MHz, $CDCl_3$ of compound **S10**

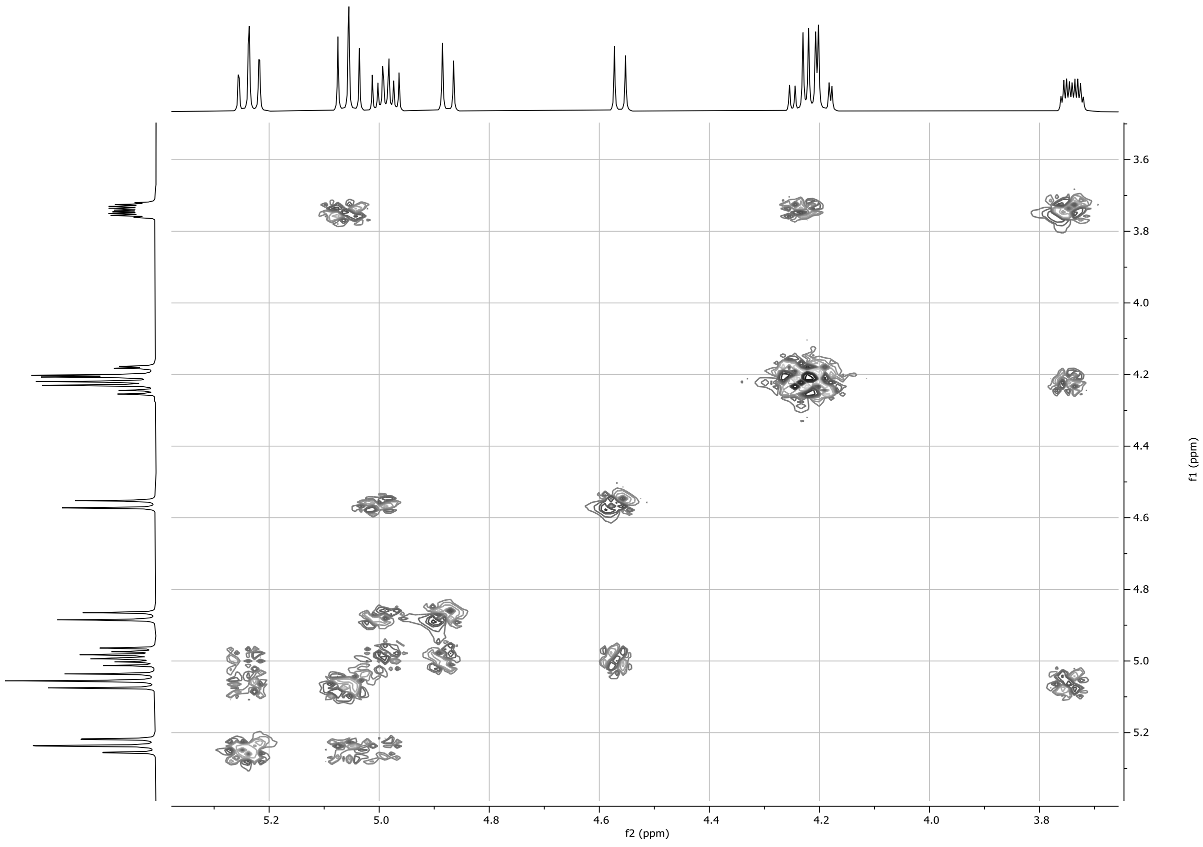

Supplementary Figure S66. HH-COSY NMR, $CDCl_3$ of compound **S10**

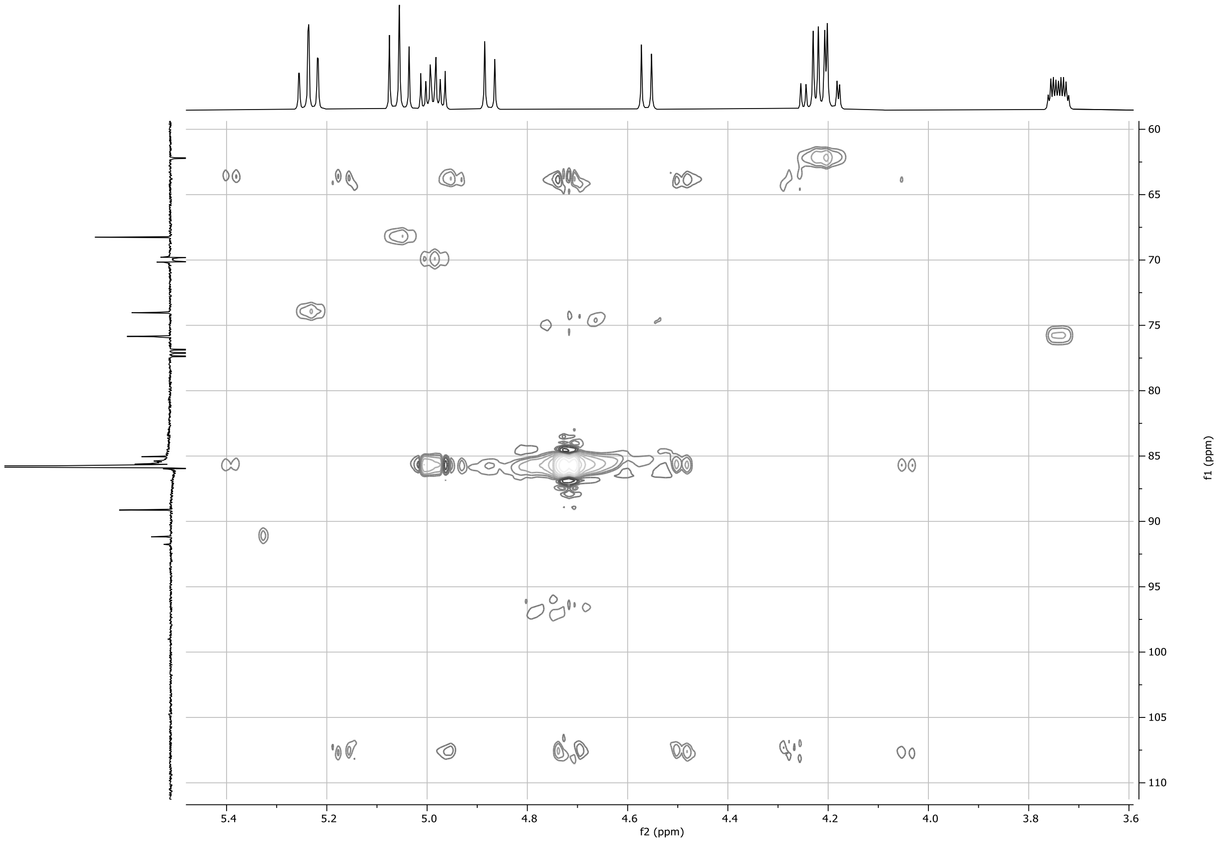

Supplementary Figure S67. HSQC{^{1}H} NMR, $CDCl_3$ of compound **S10**

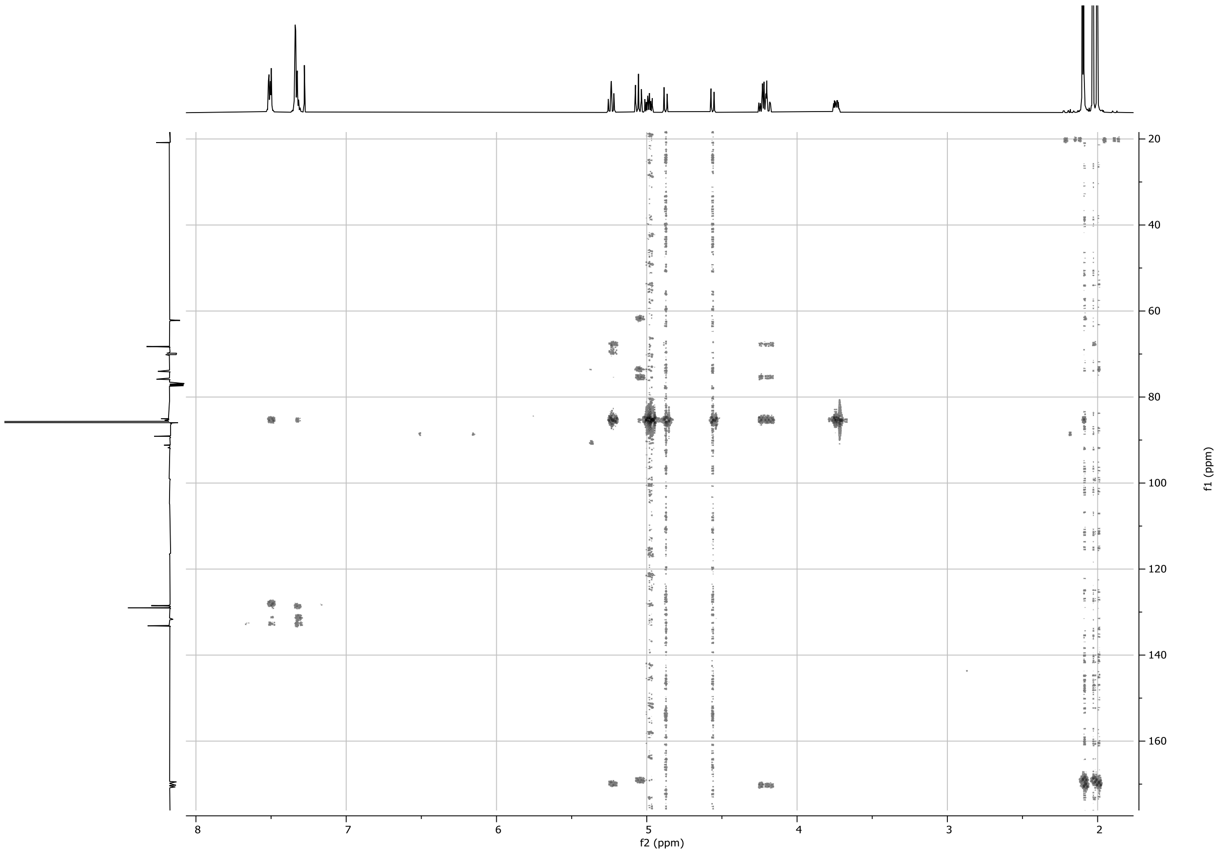

Supplementary Figure S68. HMBC{^{1}H} NMR, $CDCl_3$ of compound **S10**

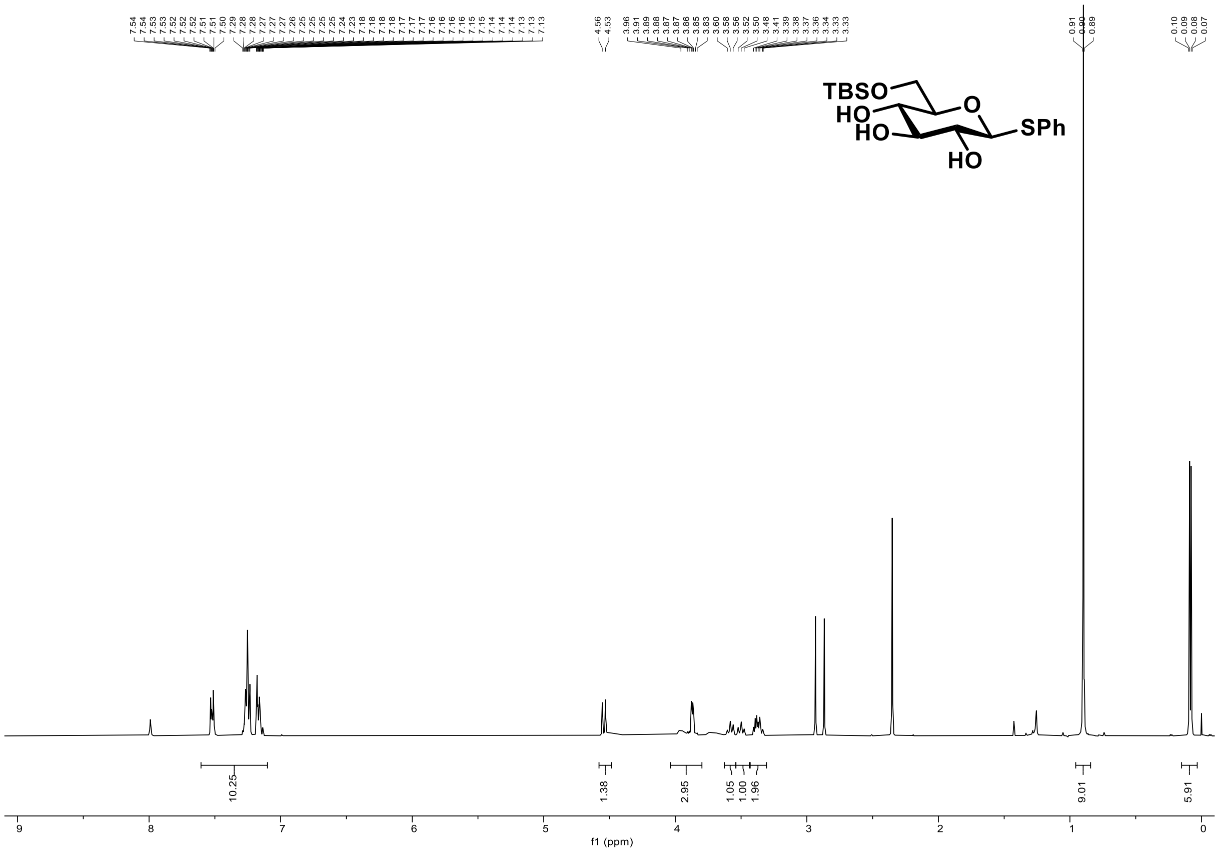

Supplementary Figure S69. ^{1}H NMR, 400 MHz, $CDCl_3$ of compound **S11**

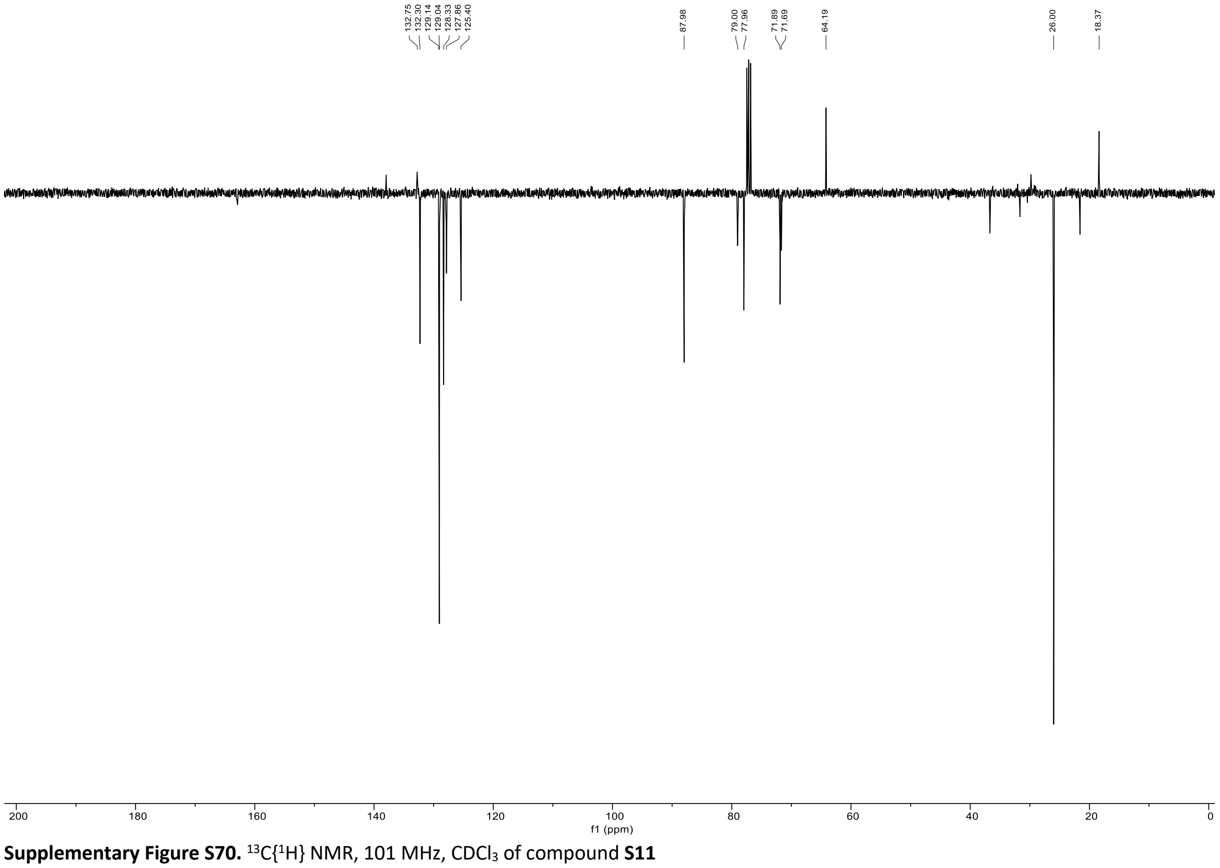

Supplementary Figure S70. $^{13}C\{^{1}H\}$ NMR, 101 MHz, $CDCl_3$ of compound **S11**

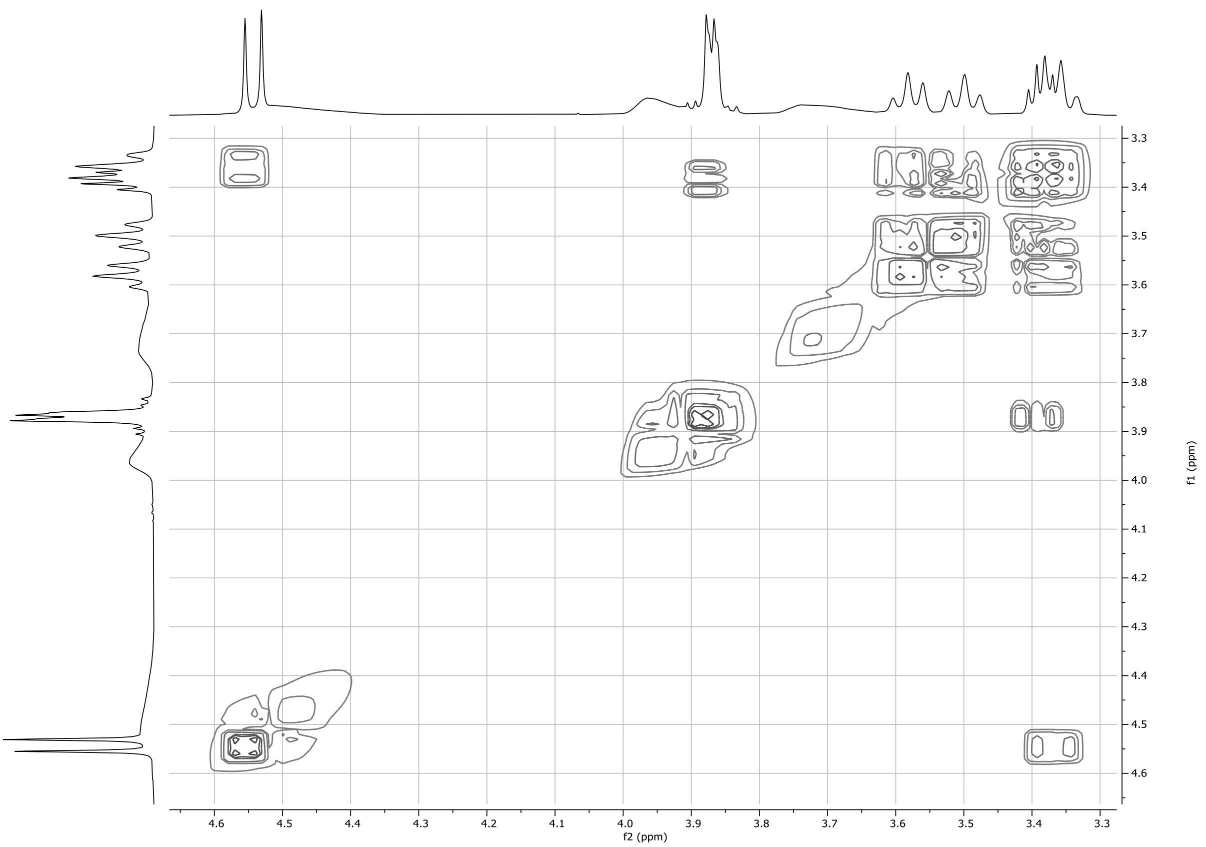

Supplementary Figure S71. HH-COSY NMR, $CDCl_3$ of compound **S11**

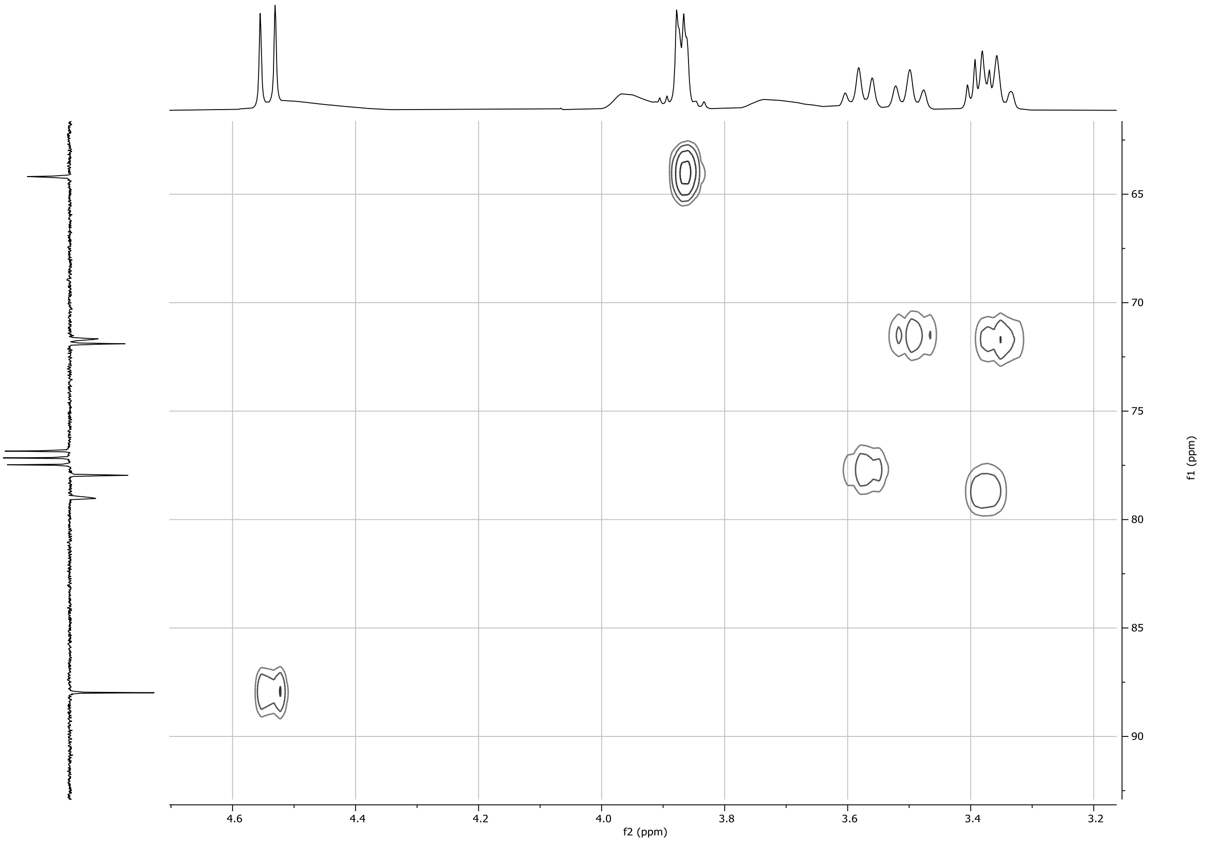

Supplementary Figure S72. HSQC{^{1}H} NMR, $CDCl_3$ of compound **S11**

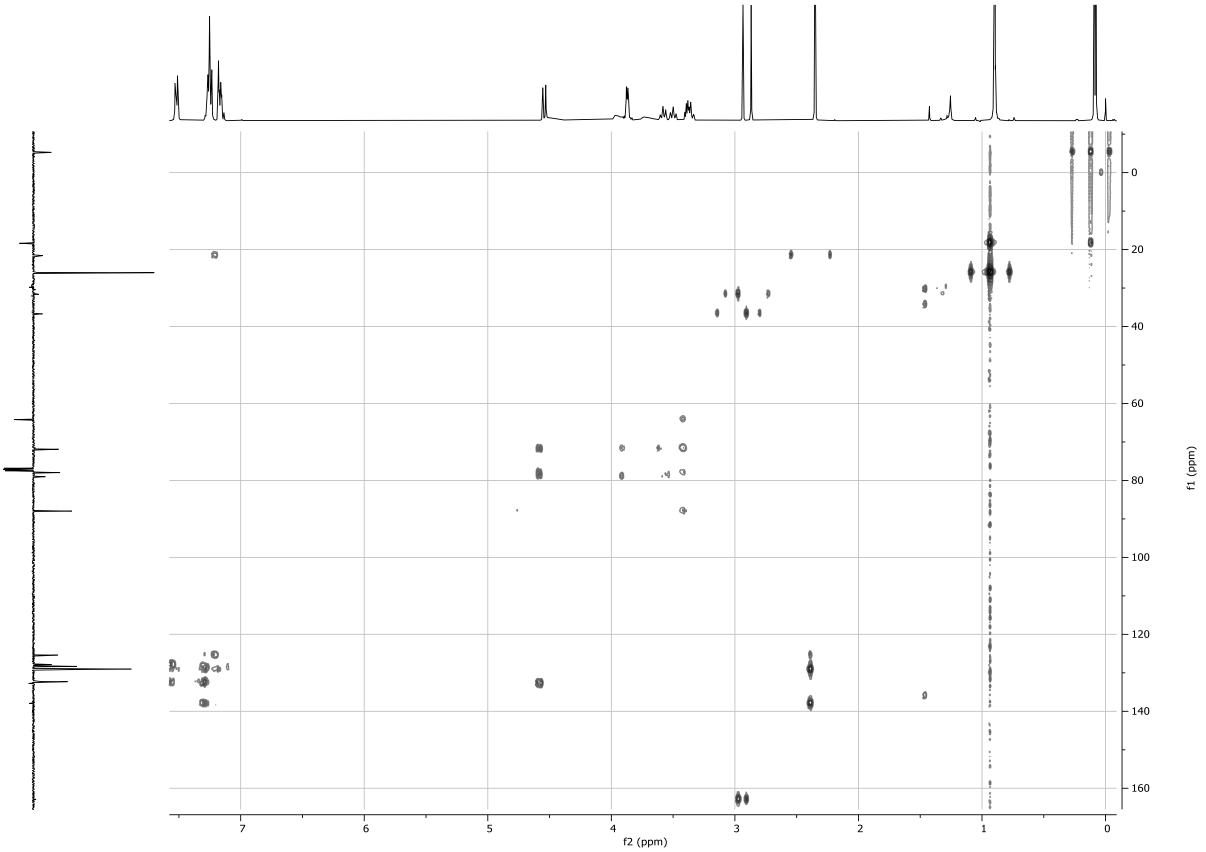

Supplementary Figure S73. HMBC{^{1}H} NMR, $CDCl_3$ of compound **S11**

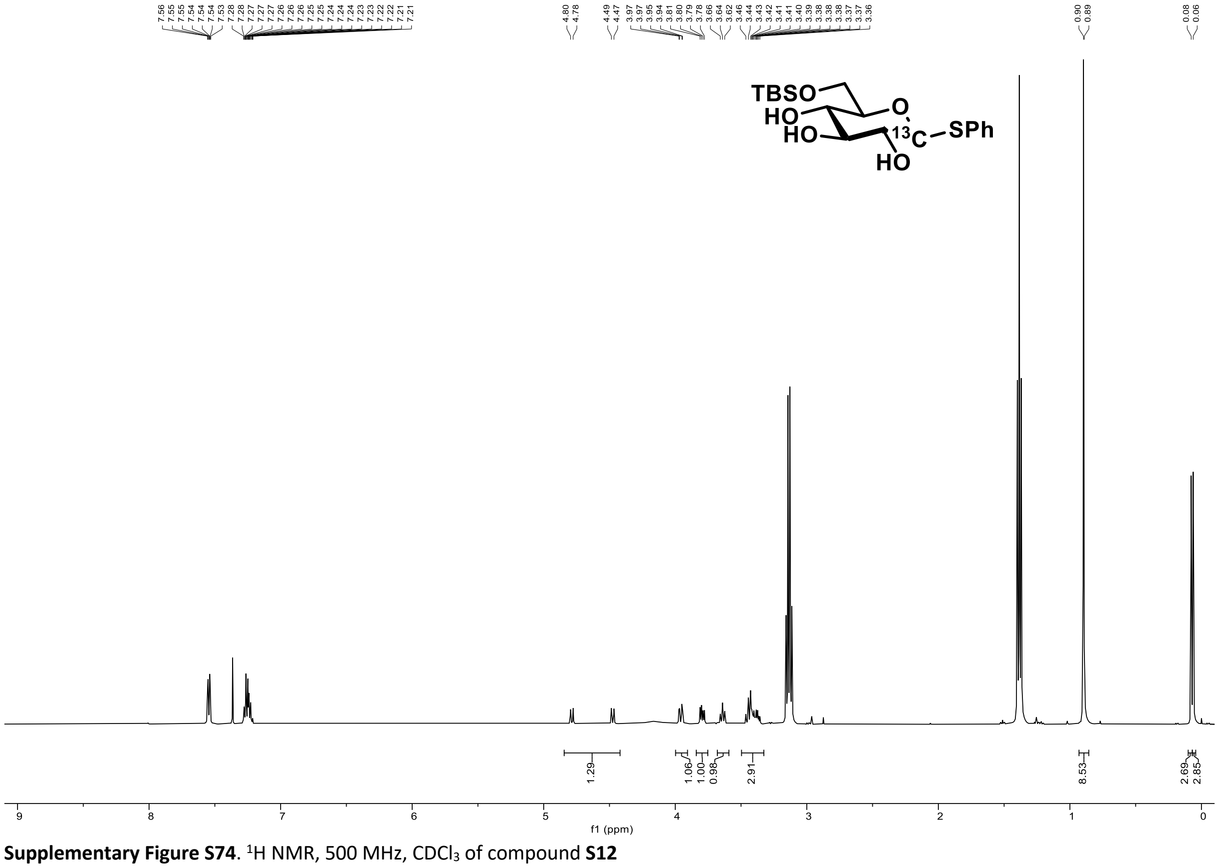

Supplementary Figure S74. ^{1}H NMR, 500 MHz, $CDCl_3$ of compound **S12**

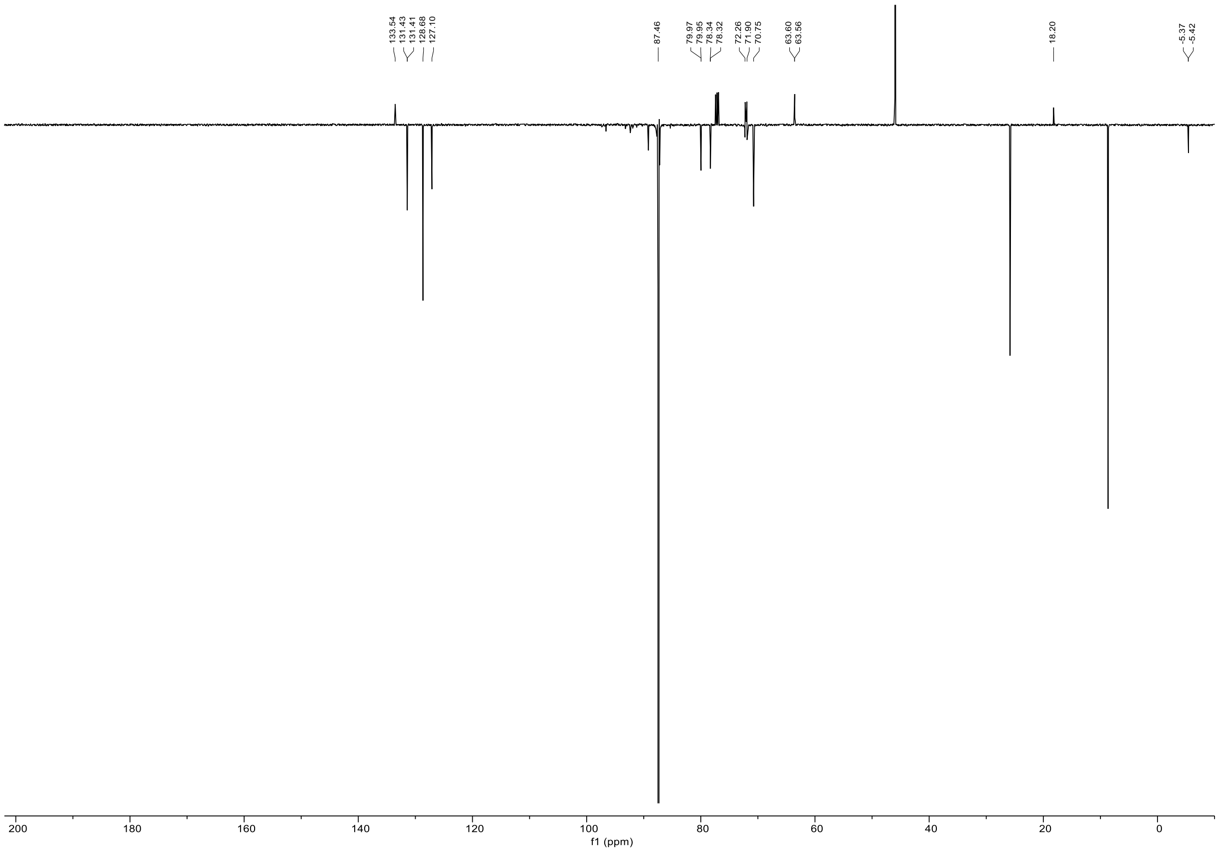

Supplementary Figure S75. $^{13}C\{^{1}H\}$ NMR, 126 MHz, $CDCl_3$ of compound **S12**

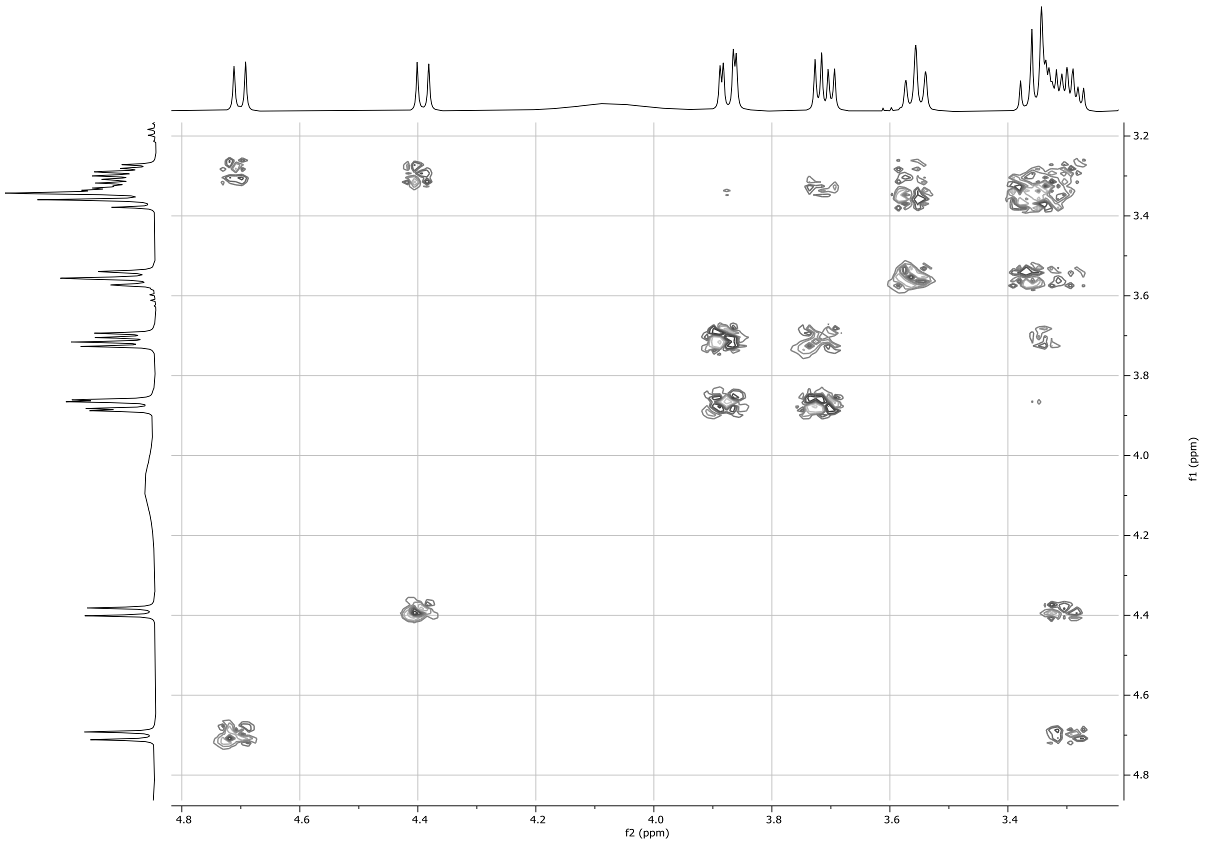

Supplementary Figure S76. HH-COSY NMR, $CDCl_3$ of compound **S12**

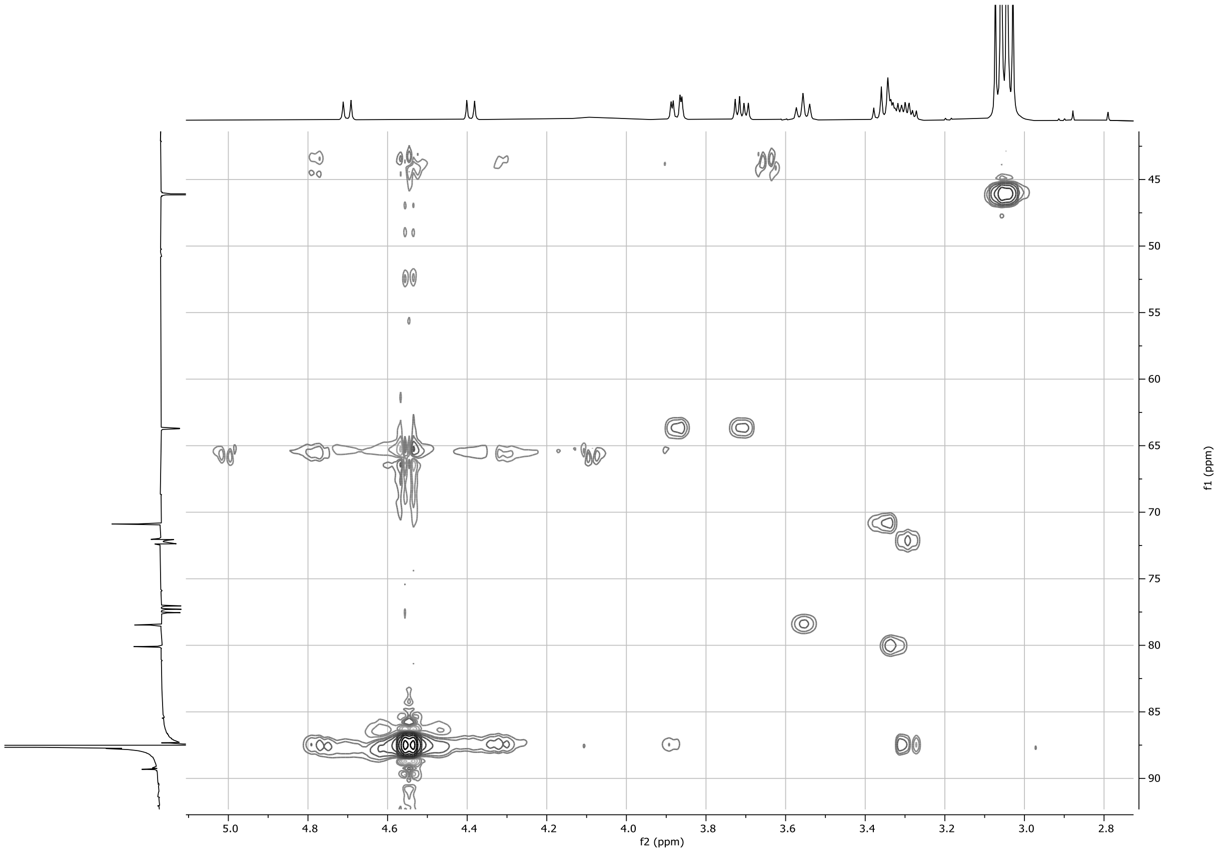

Supplementary Figure S77. HSQC{^{1}H} NMR, $CDCl_3$ of compound **S12**

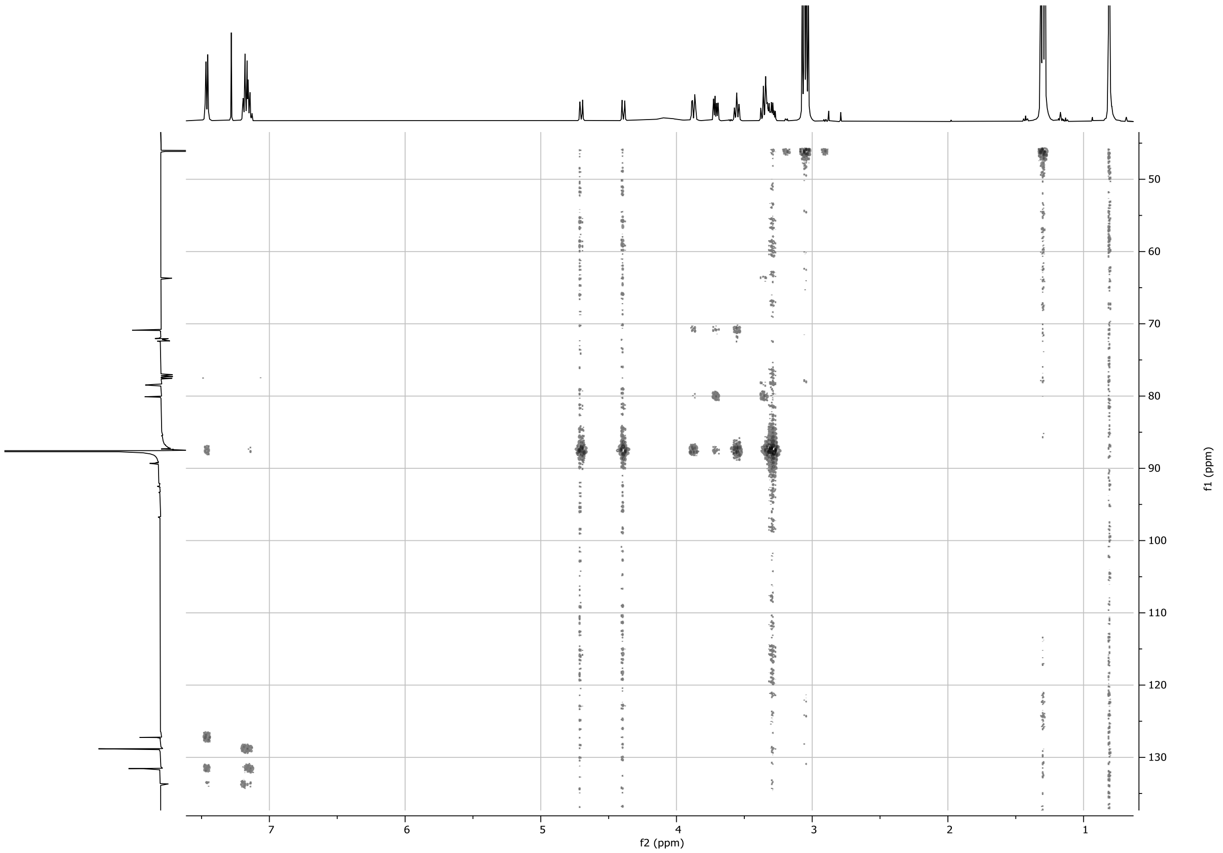

Supplementary Figure S78. HMBC{^{1}H} NMR, $CDCl_3$ of compound **S12**

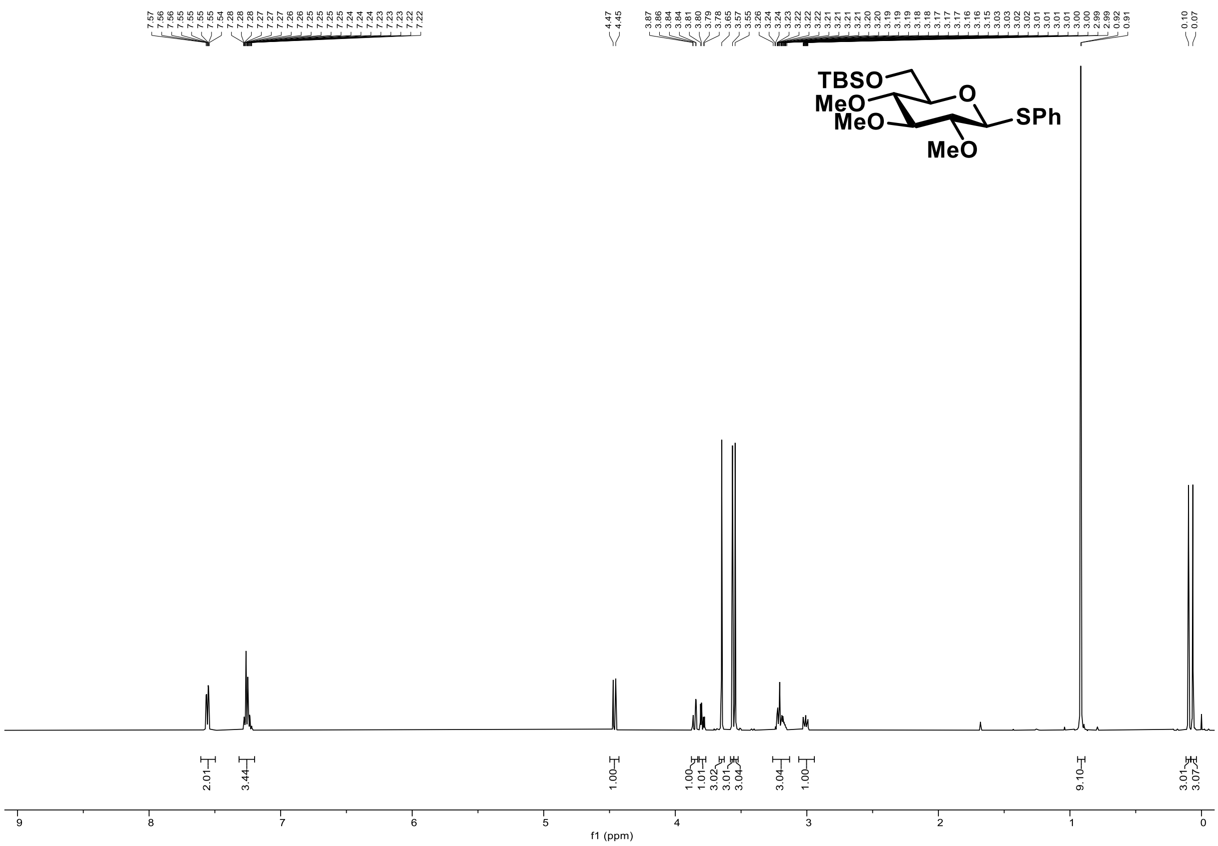

Supplementary Figure S79. ^{1}H NMR, 500 MHz, $CDCl_3$ of compound **S13**

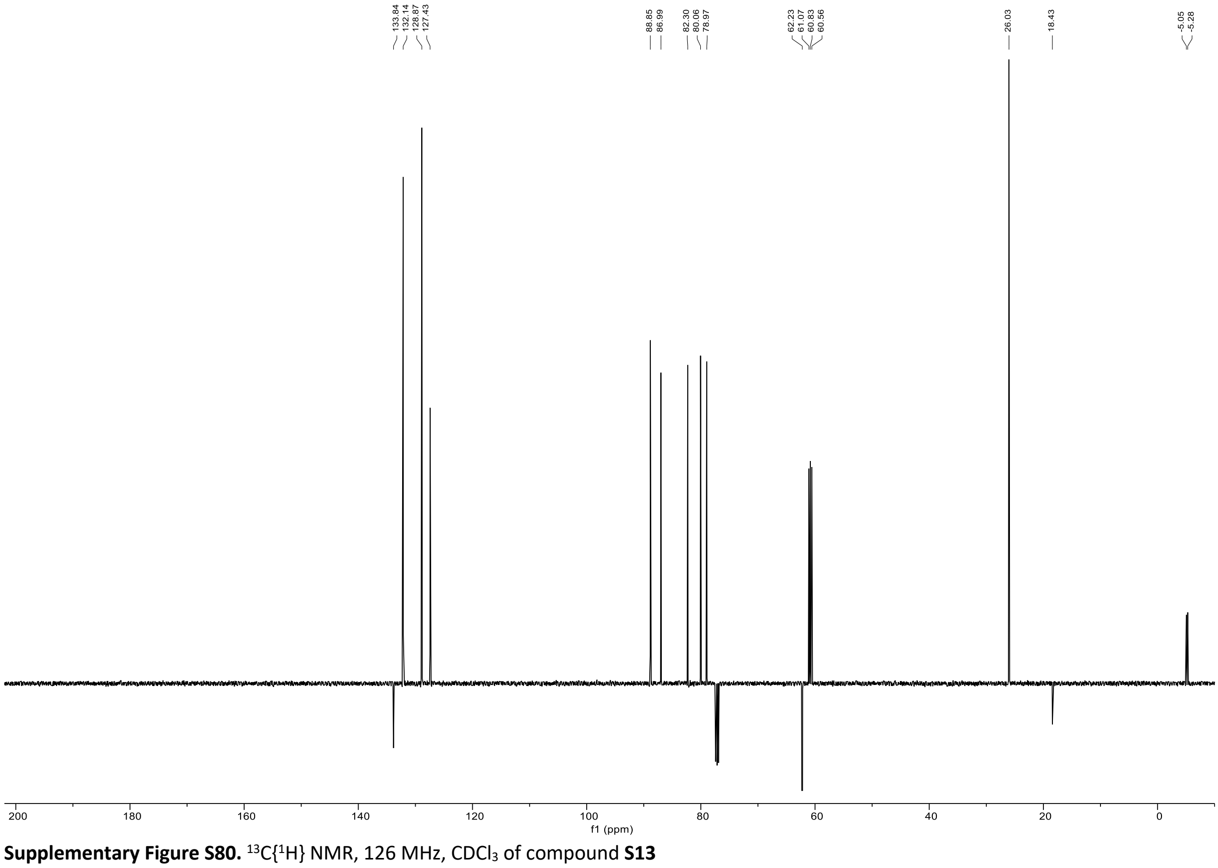

Supplementary Figure S80. $^{13}C\{^{1}H\}$ NMR, 126 MHz, $CDCl_3$ of compound **S13**

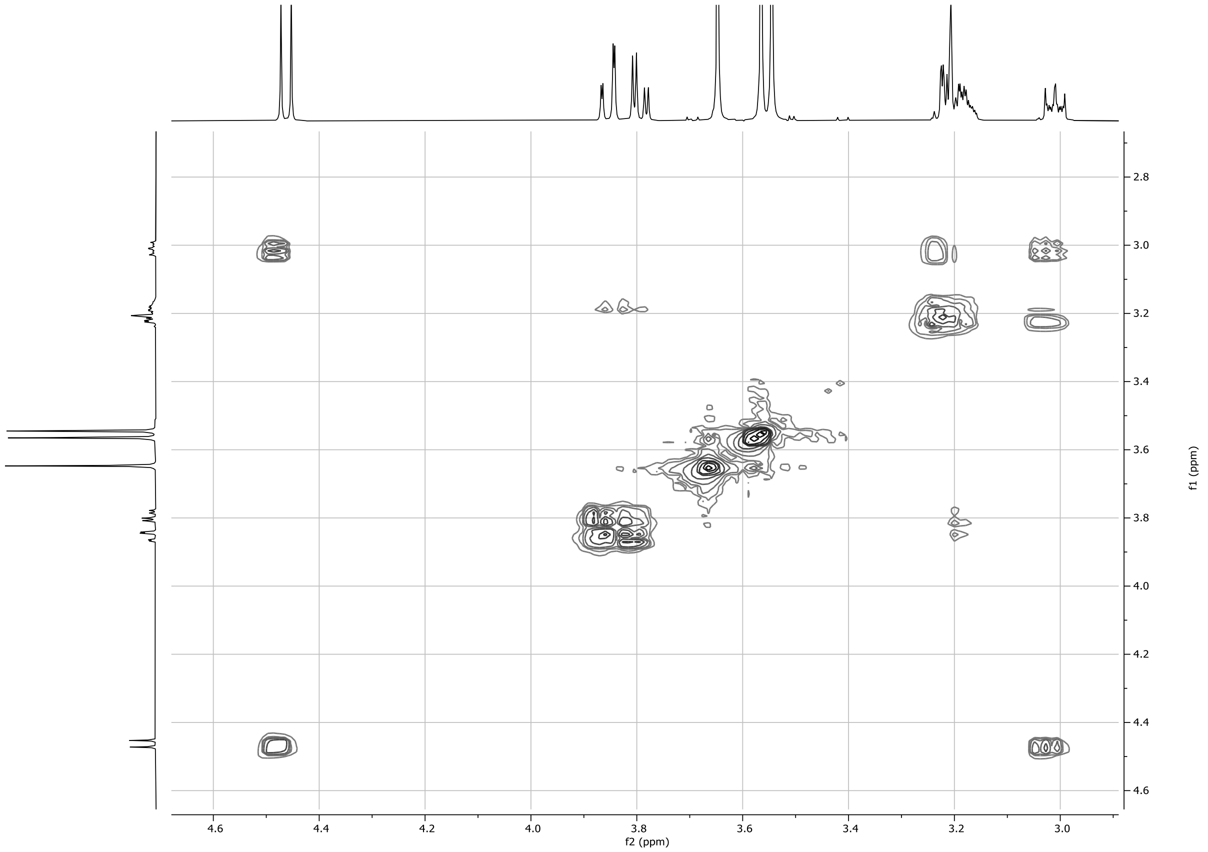

Supplementary Figure S81. HH-COSY NMR, $CDCl_3$ of compound **S13**

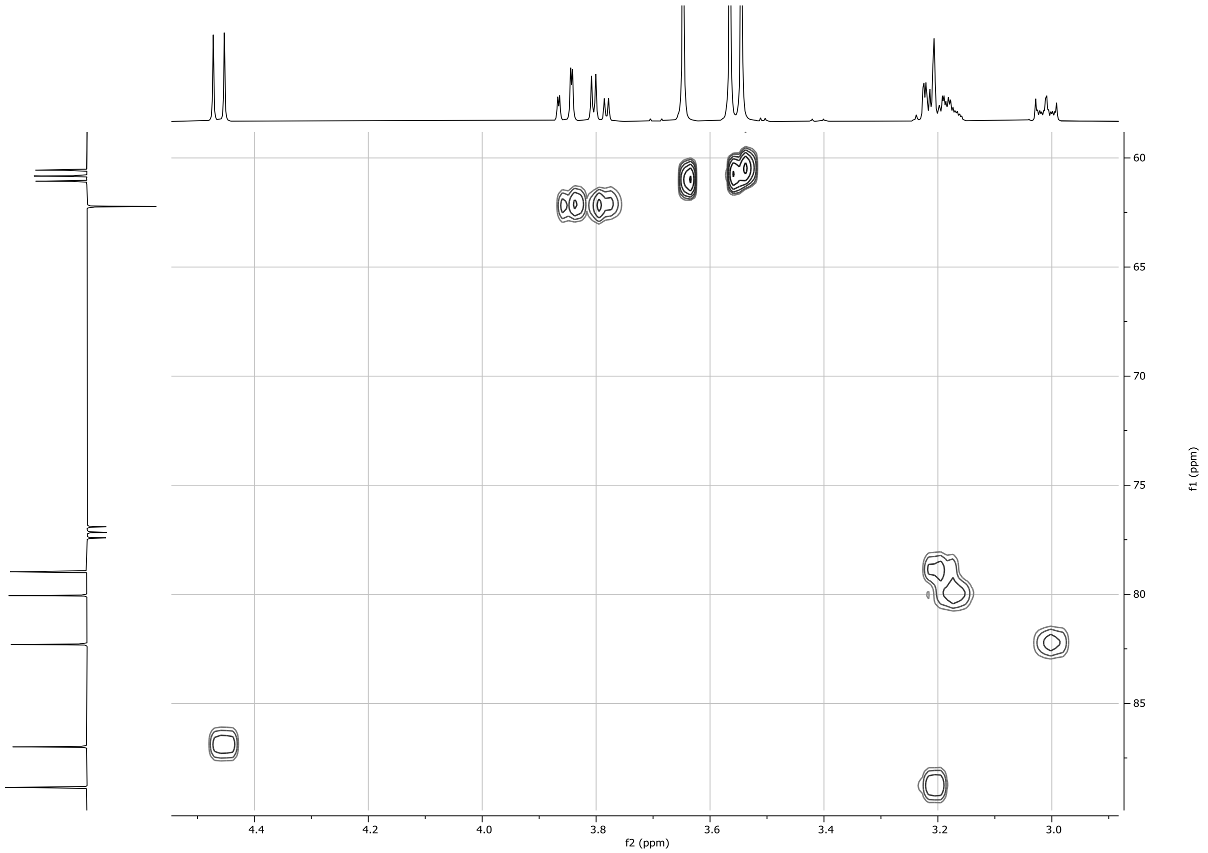

Supplementary Figure S82. HSQC{^{1}H} NMR, $CDCl_3$ of compound **S13**

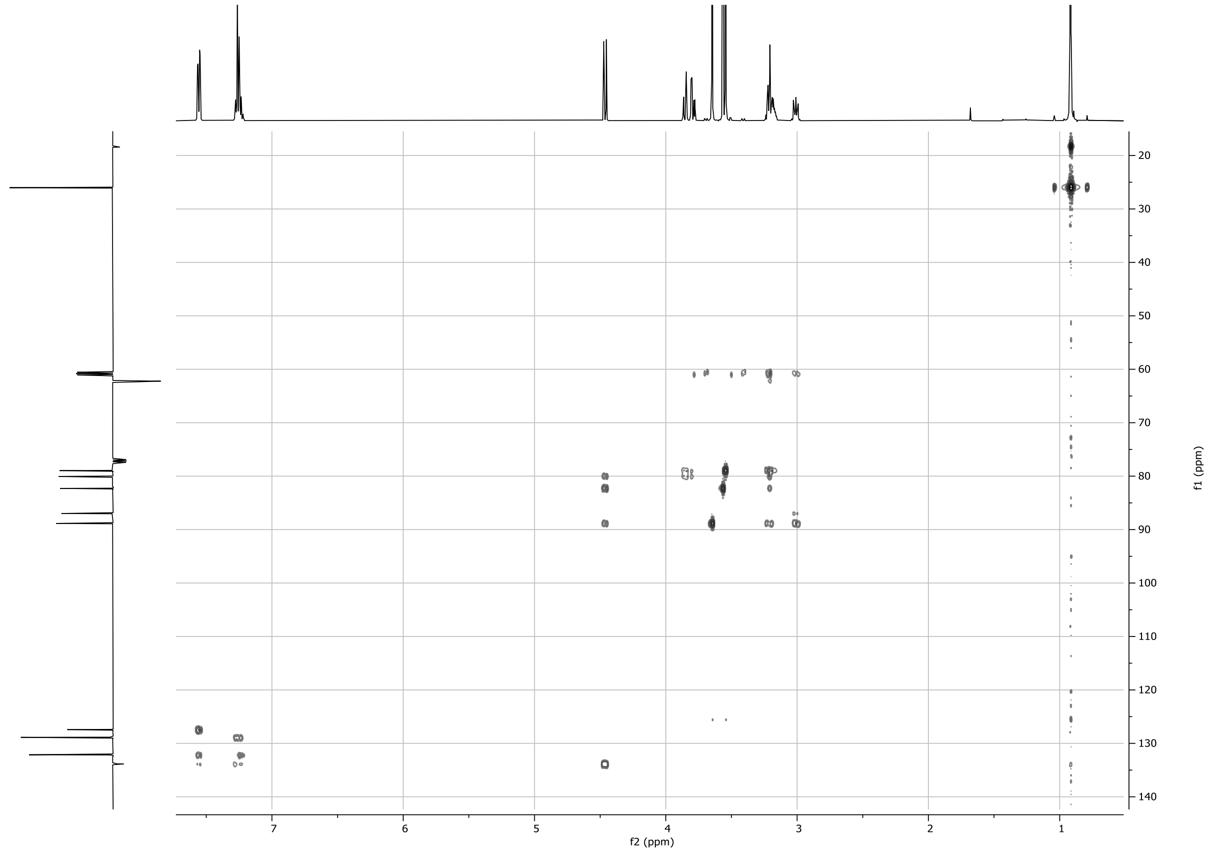

Supplementary Figure S83. HMBC{^{1}H} NMR, $CDCl_3$ of compound **S13**

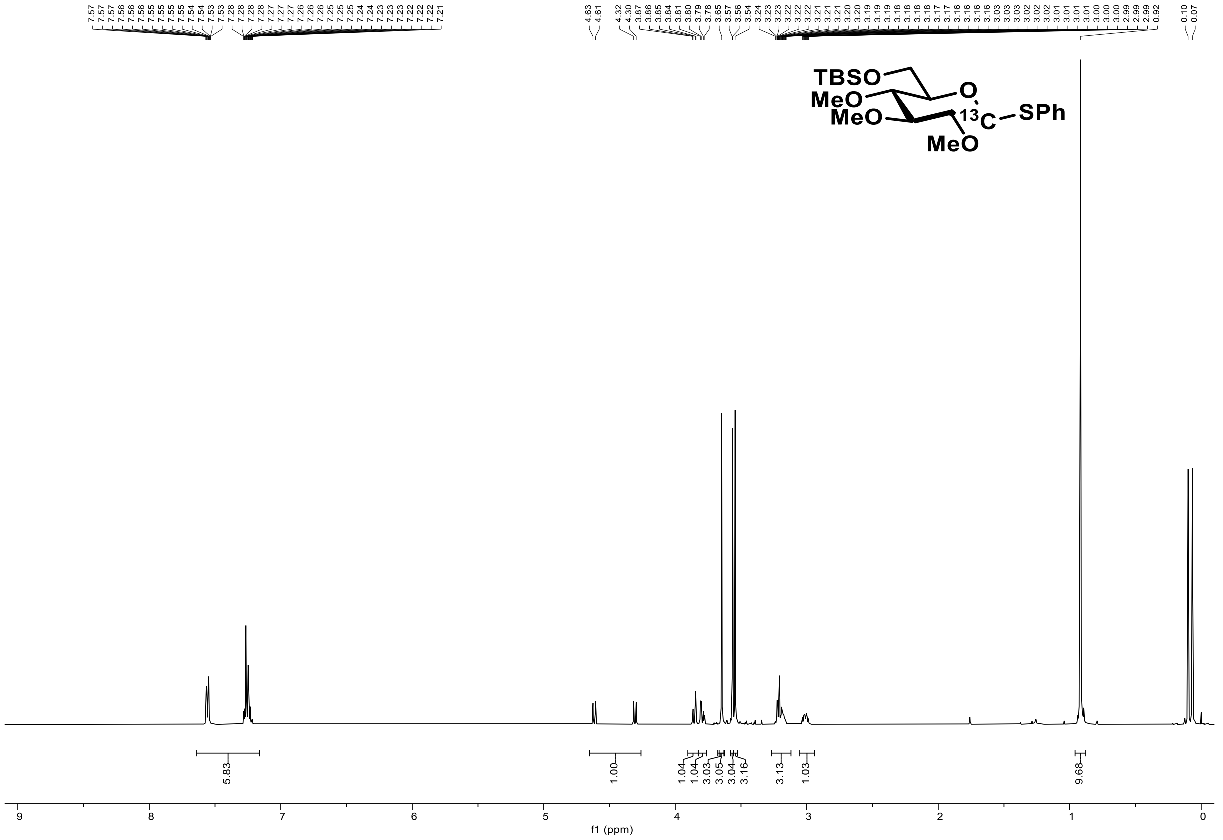

Supplementary Figure S84. ^{1}H NMR, 500 MHz, $CDCl_3$ of compound **S14**

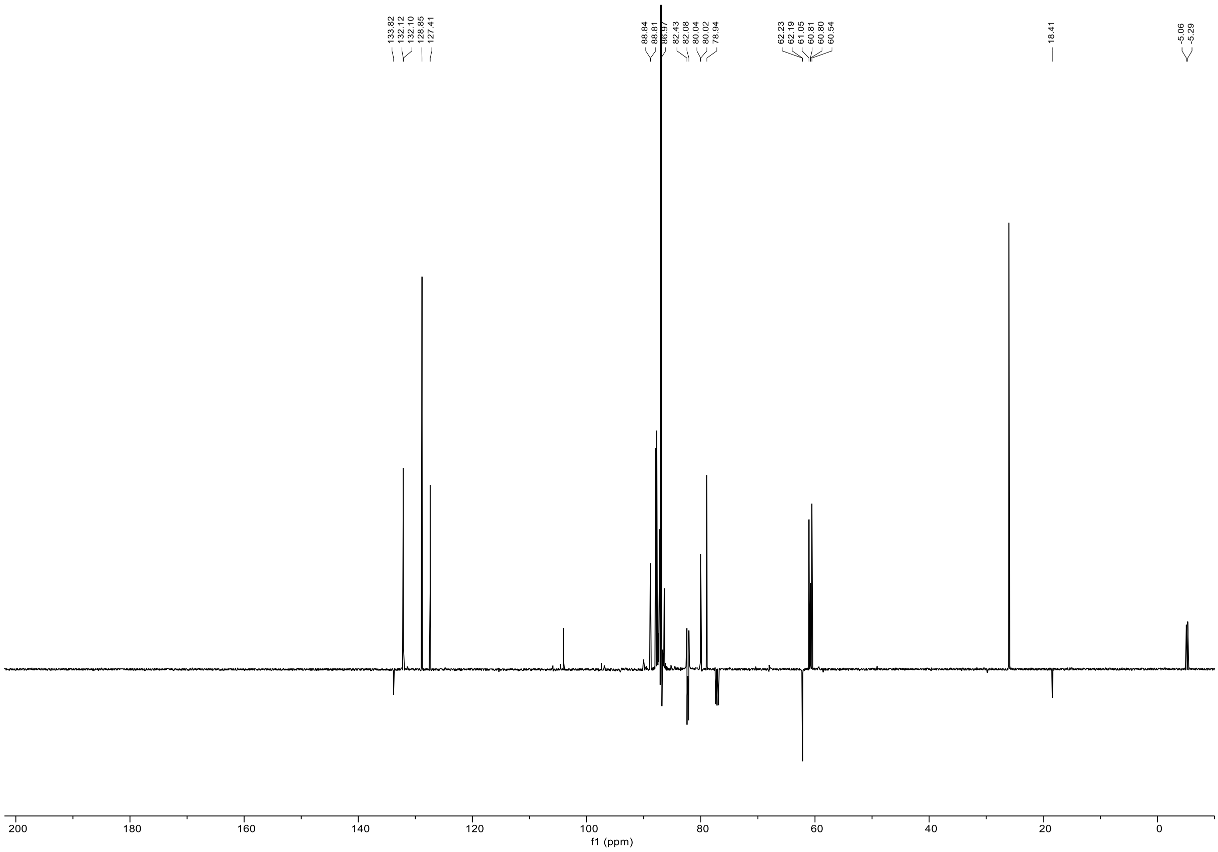

Supplementary Figure S85. $^{13}C\{^{1}H\}$ NMR, 126 MHz, $CDCl_3$ of compound **S14**

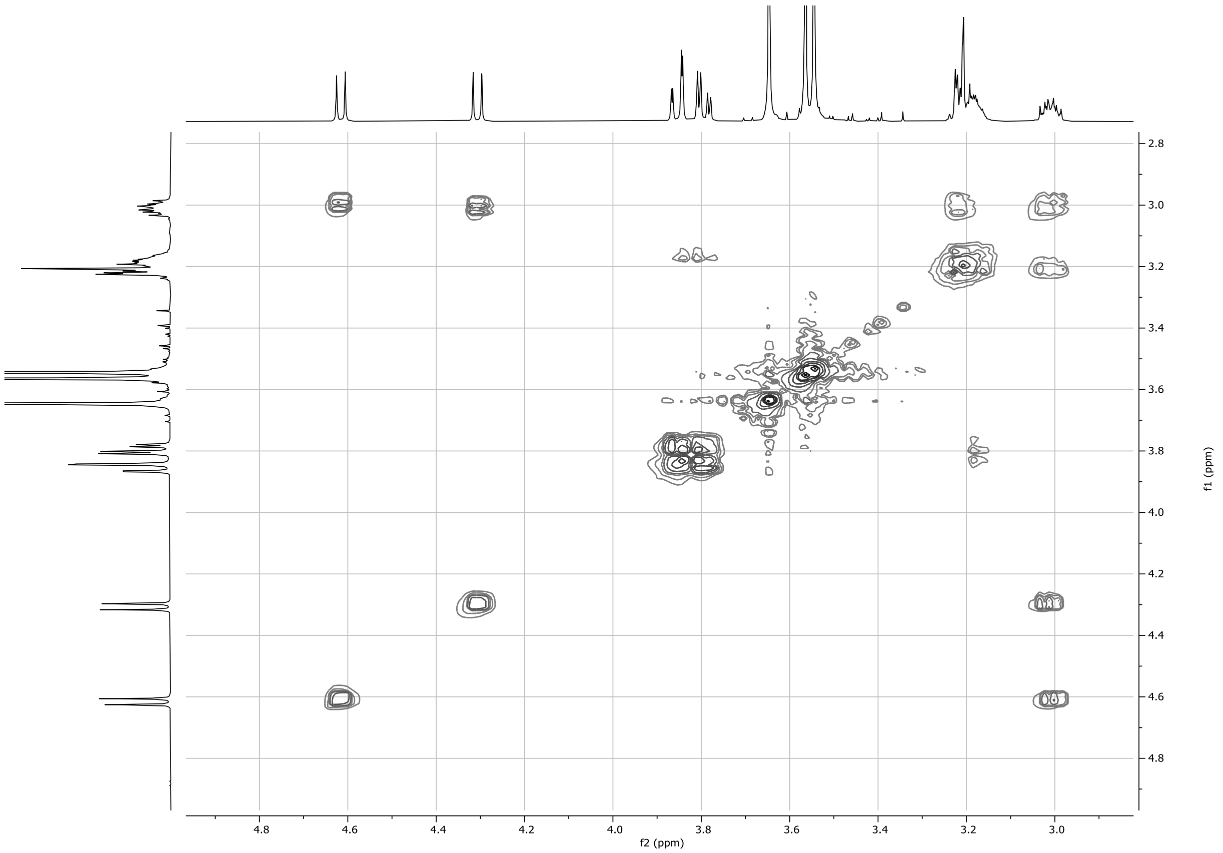

Supplementary Figure S86. HH-COSY NMR, $CDCl_3$ of compound **S14**

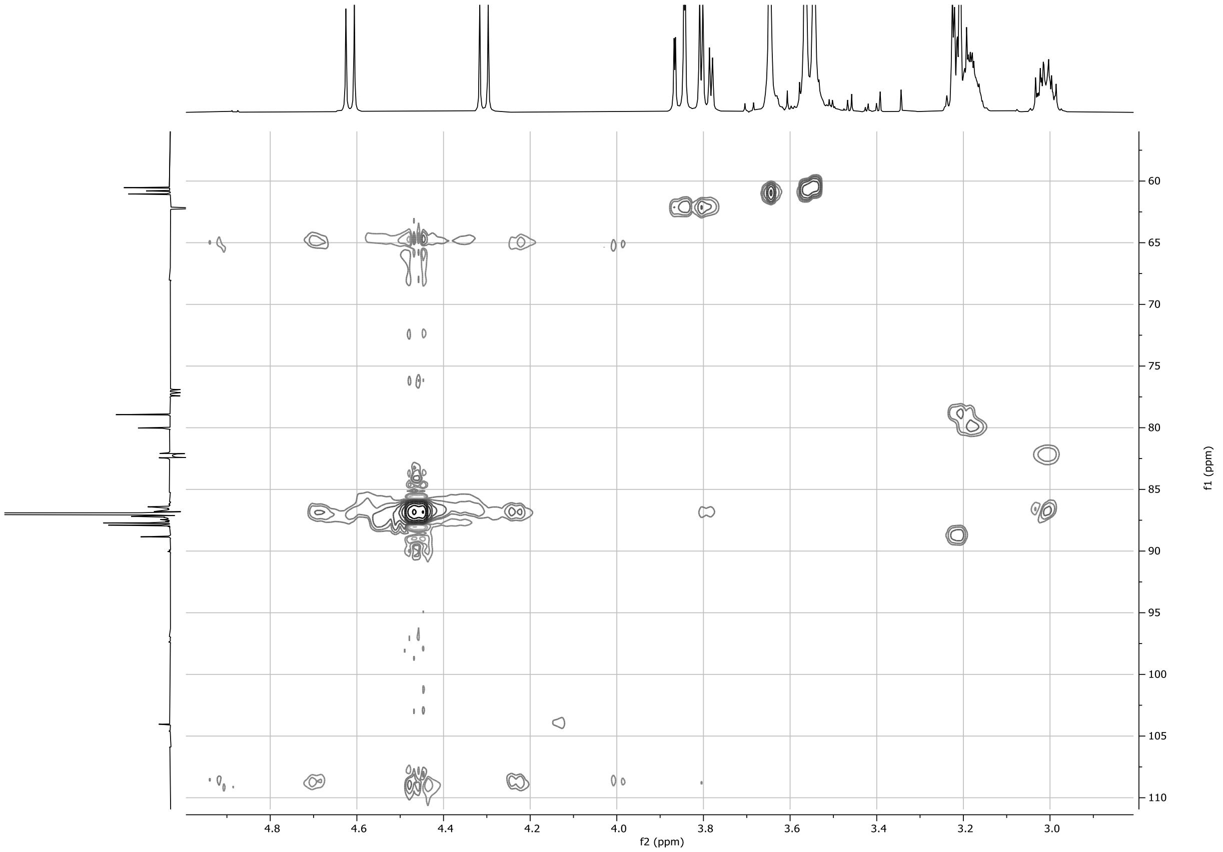

Supplementary Figure S87. HSQC{^{1}H} NMR, $CDCl_3$ of compound **S14**

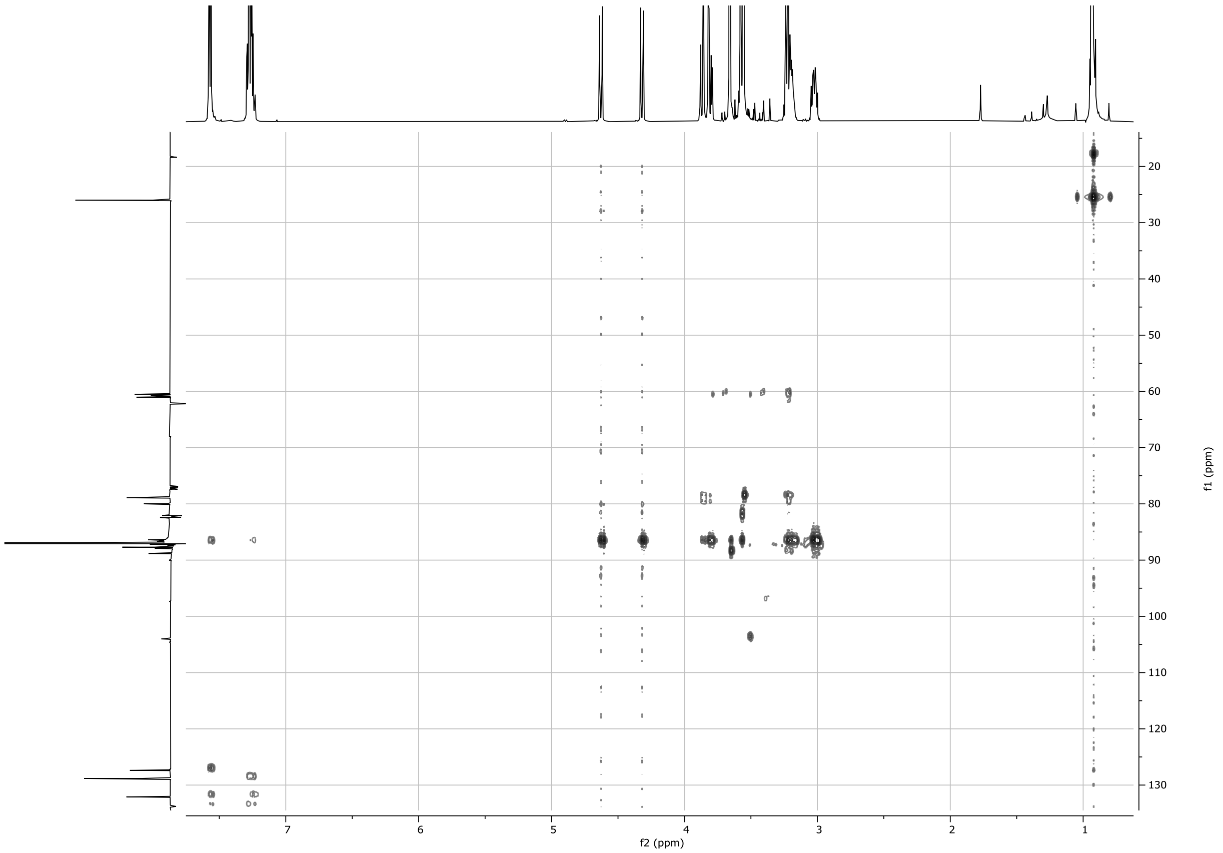

Supplementary Figure S88. HMBC{^{1}H} NMR, $CDCl_3$ of compound **S14**

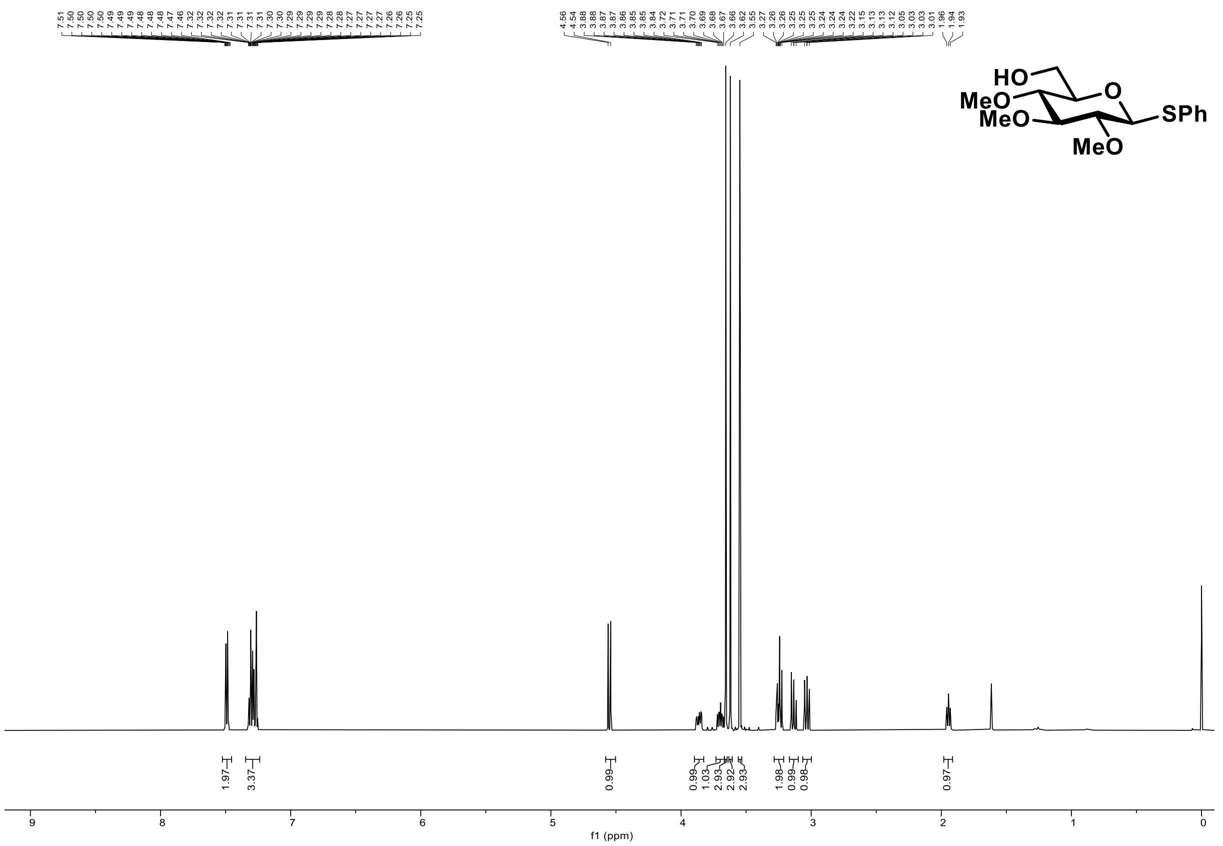

Supplementary Figure S89. ^{1}H NMR, 500 MHz, $CDCl_3$ of compound **S15**

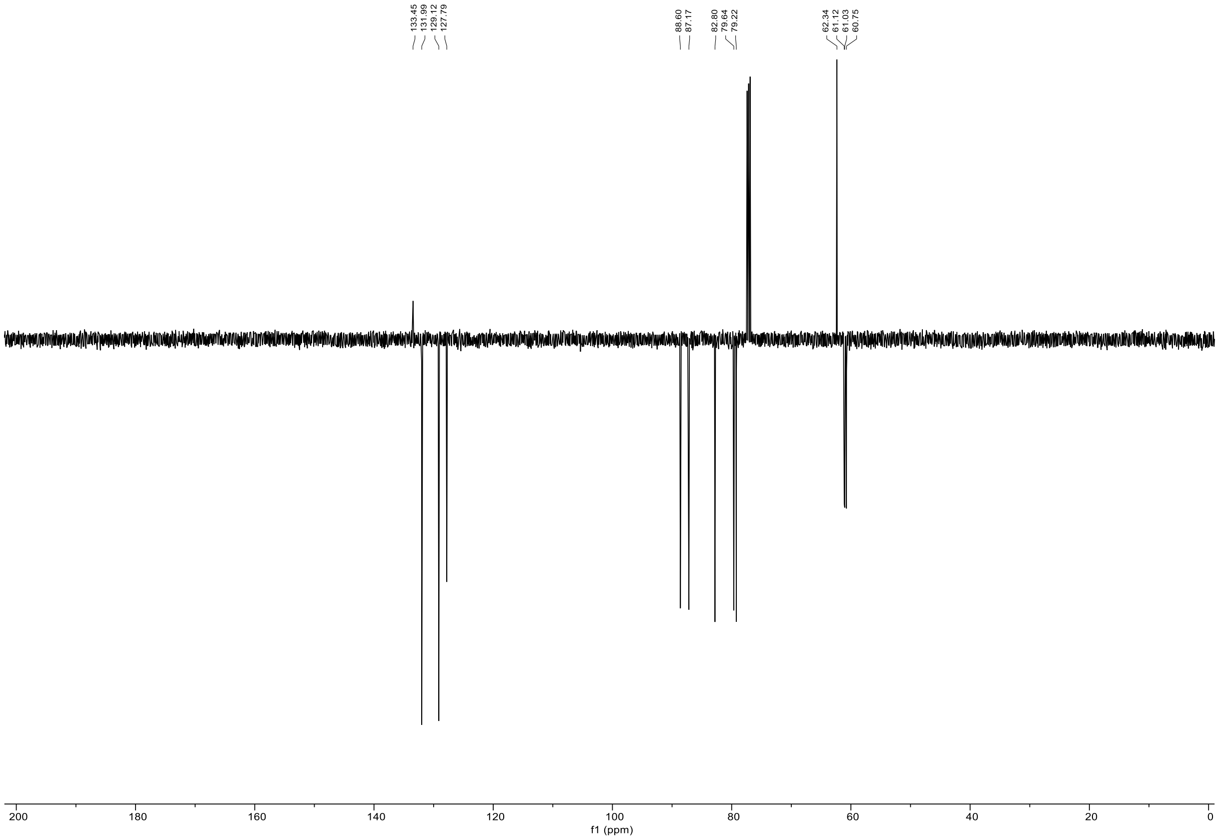

Supplementary Figure S90. $^{13}C\{^{1}H\}$ NMR, 126 MHz, $CDCl_3$ of compound **S15**

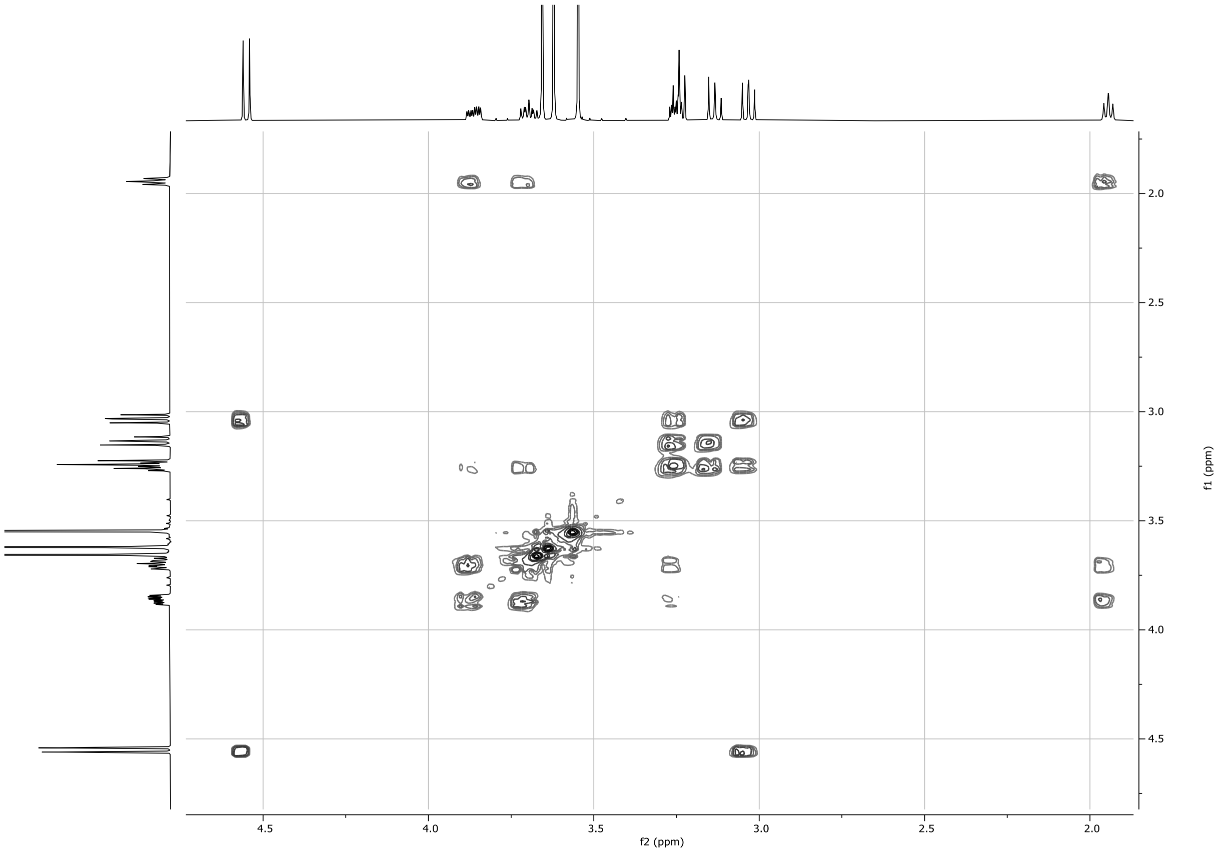

Supplementary Figure S91. HH-COSY NMR, $CDCl_3$ of compound **S15**

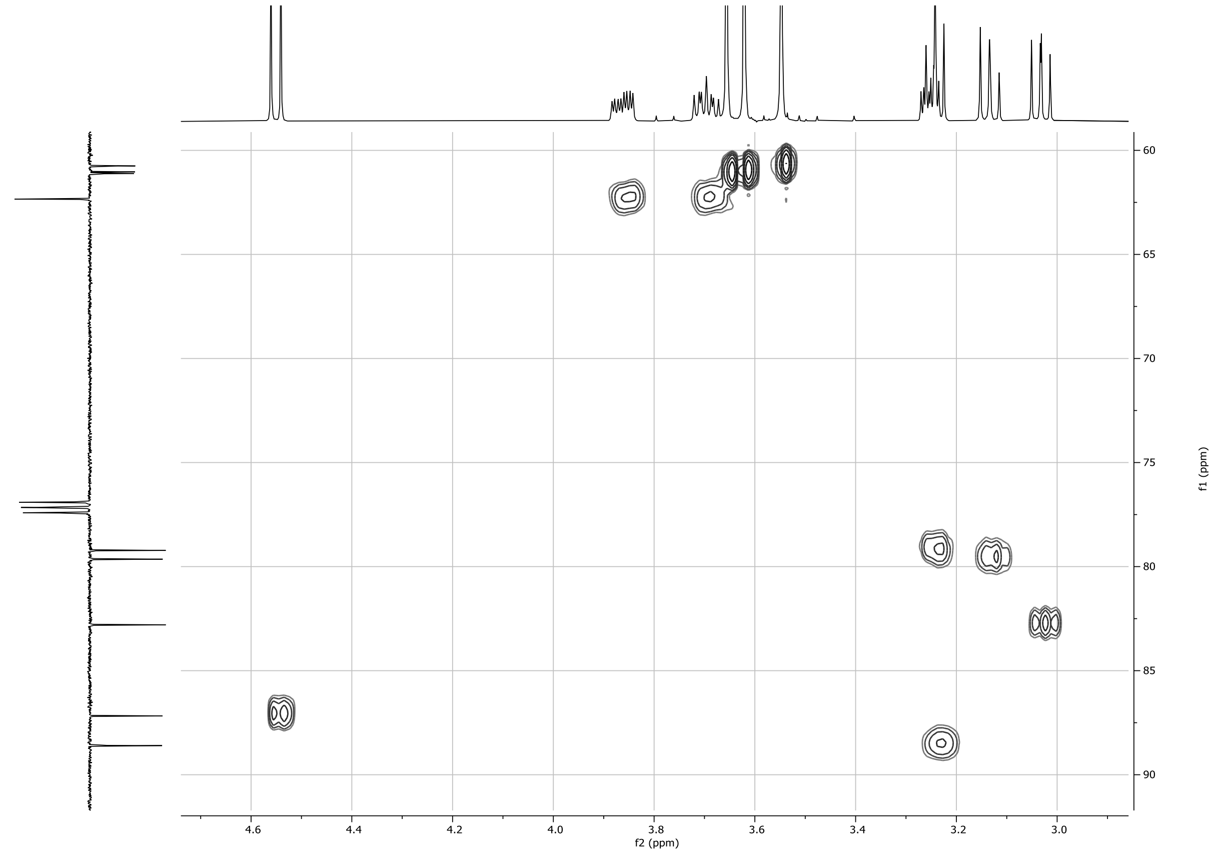

Supplementary Figure S92. HSQC{^{1}H} NMR, $CDCl_3$ of compound **S15**

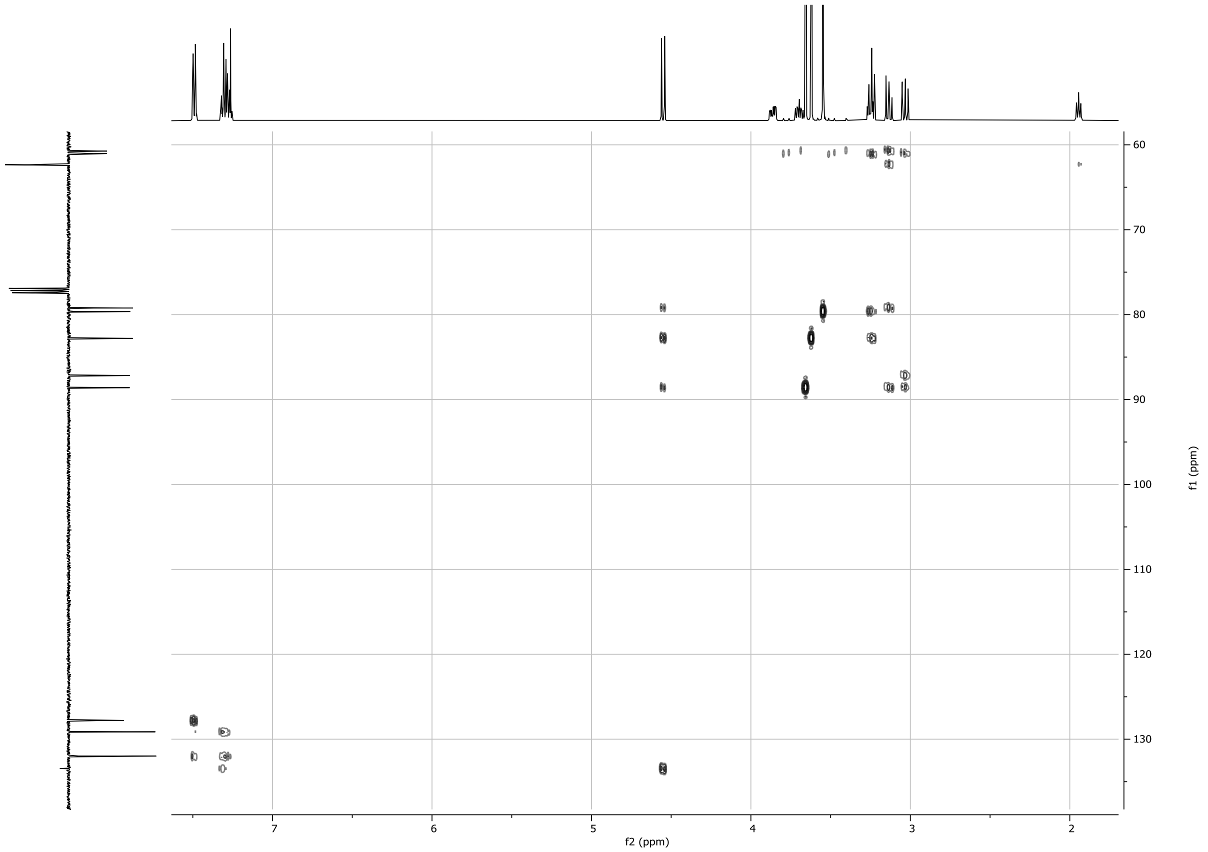

Supplementary Figure S93. HMBC{^{1}H} NMR, $CDCl_3$ of compound **S15**

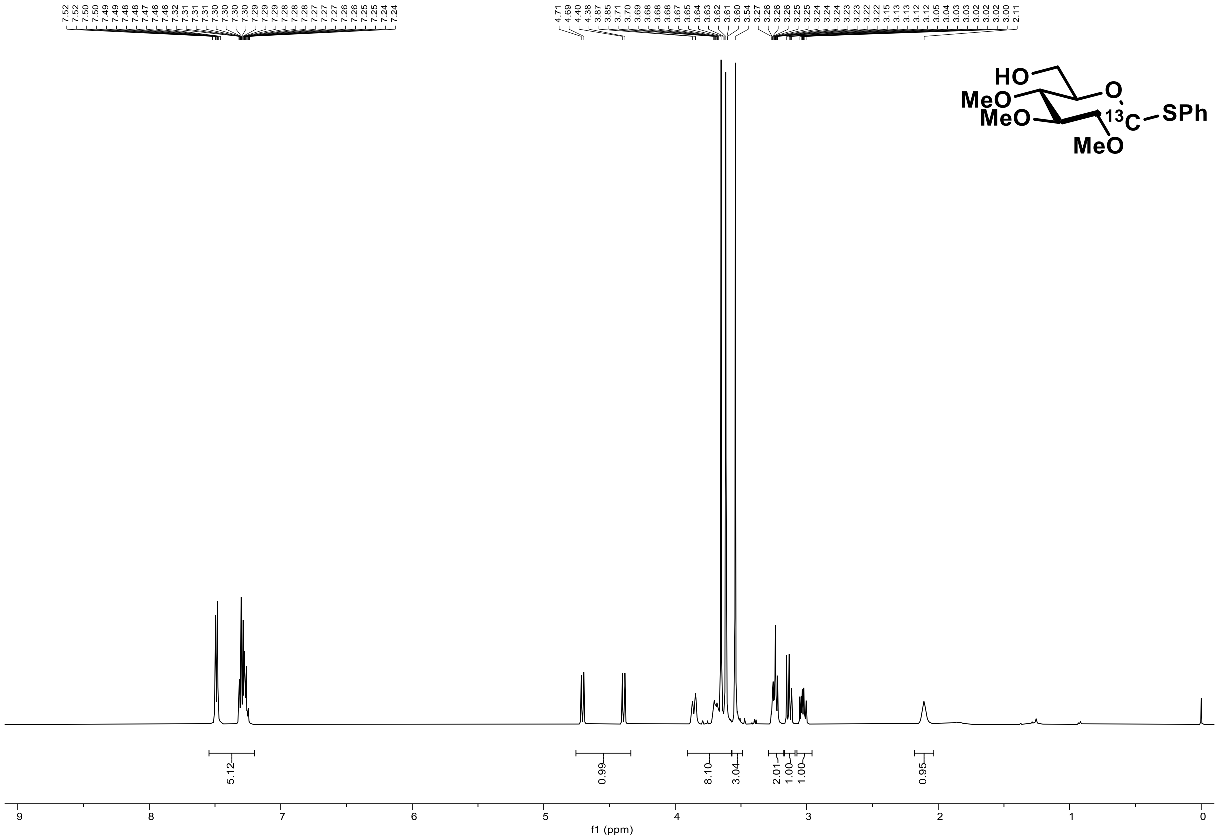

Supplementary Figure S94. ^{1}H NMR, 500 MHz, $CDCl_3$ of compound **S16**

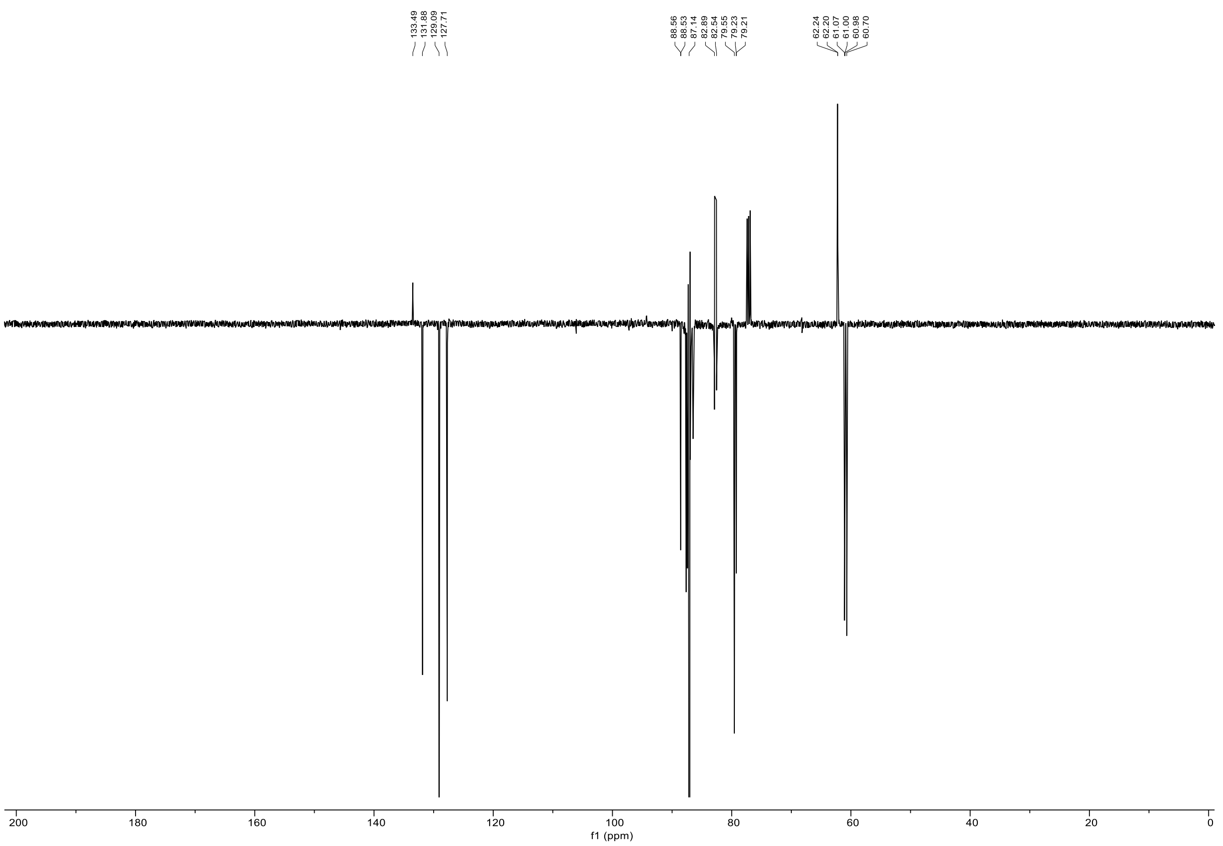

Supplementary Figure S95. $^{13}C\{^{1}H\}$ NMR, 126 MHz, $CDCl_3$ of compound **S16**

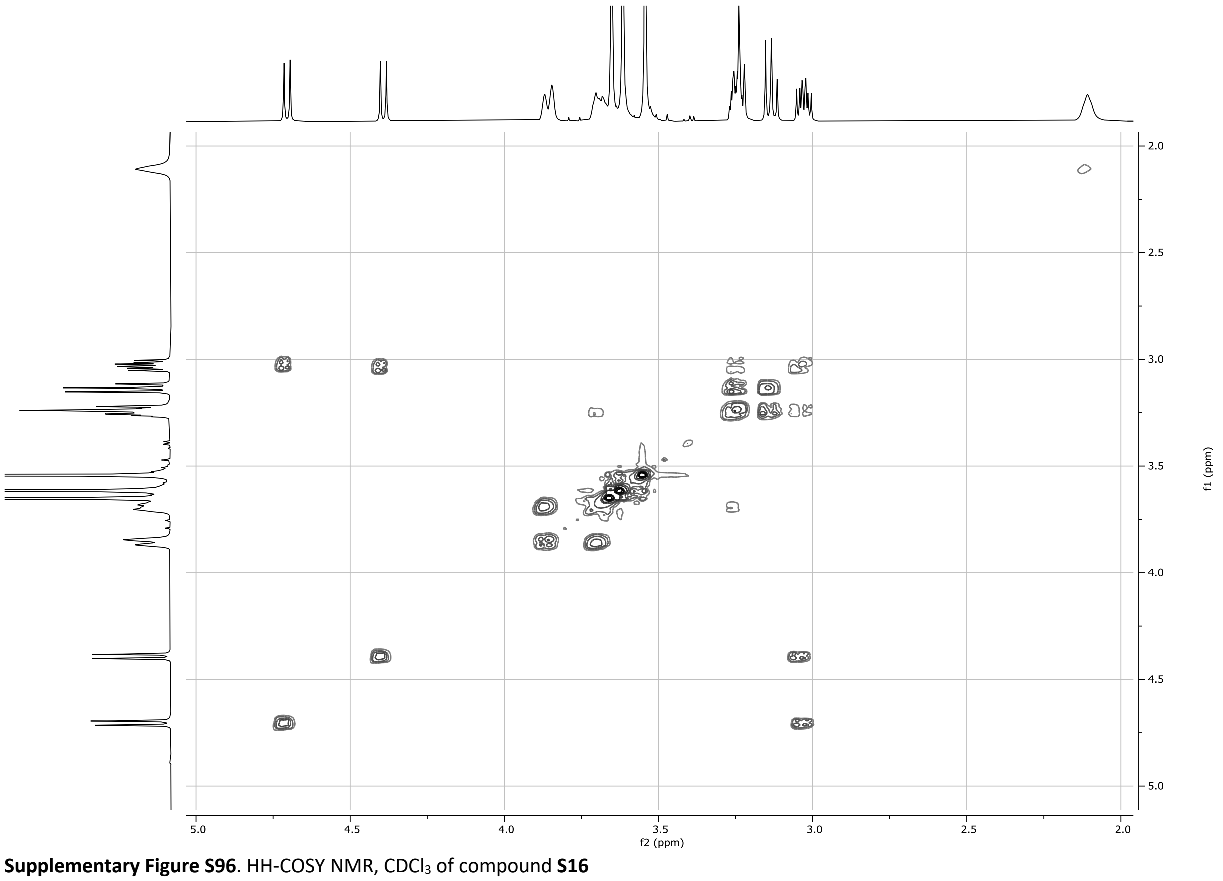

Supplementary Figure S96. HH-COSY NMR, $CDCl_3$ of compound **S16**

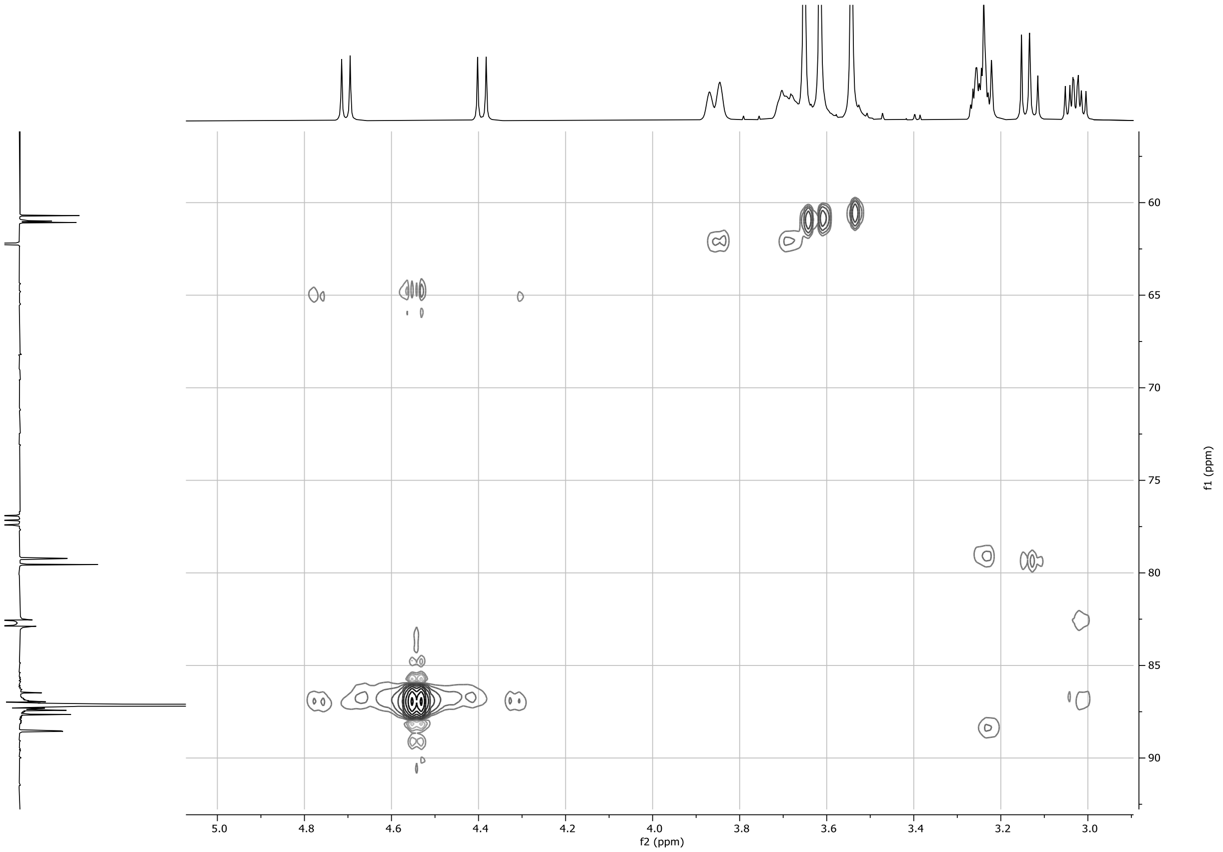

Supplementary Figure S97. HSQC{^{1}H} NMR, $CDCl_3$ of compound **S16**

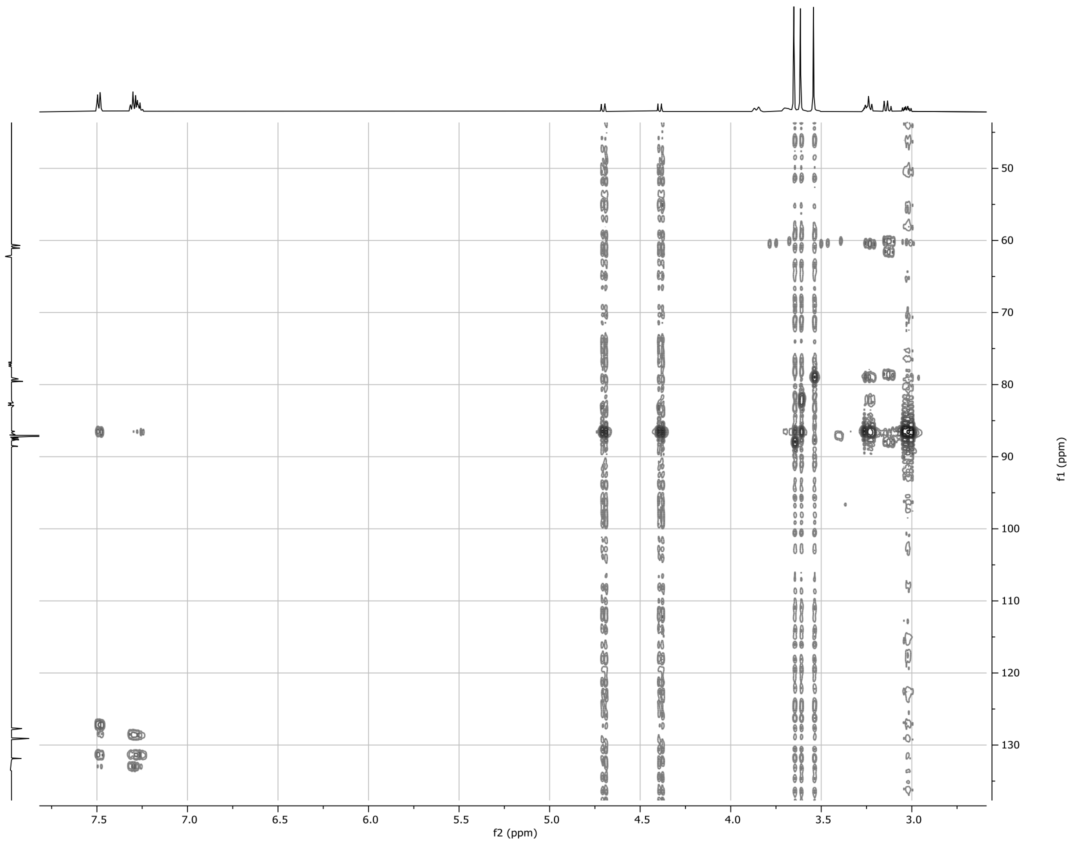

Supplementary Figure S98. HMBC{^{1}H} NMR, $CDCl_3$ of compound **S16**

Methylated donor NMR spectra

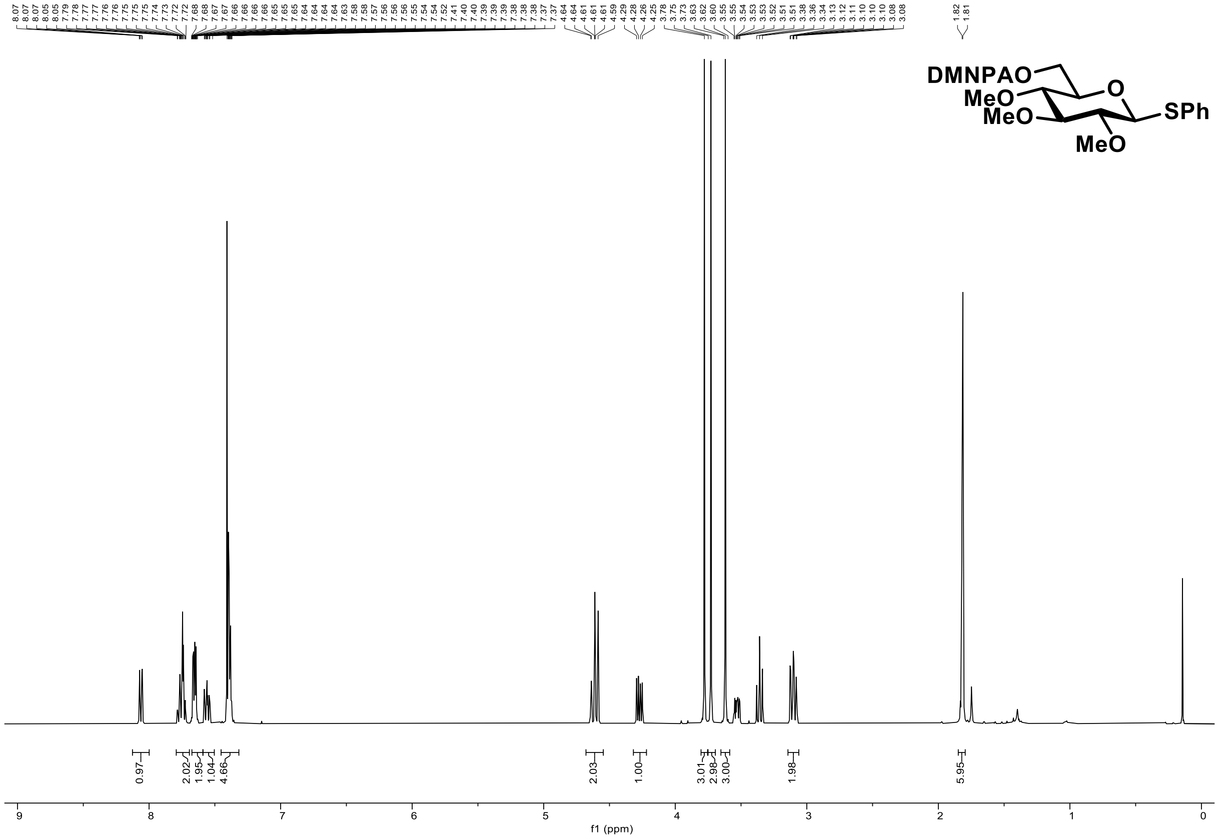

Supplementary Figure S99. ^{1}H NMR, 400 MHz, $CDCl_3$ of compound **S17**

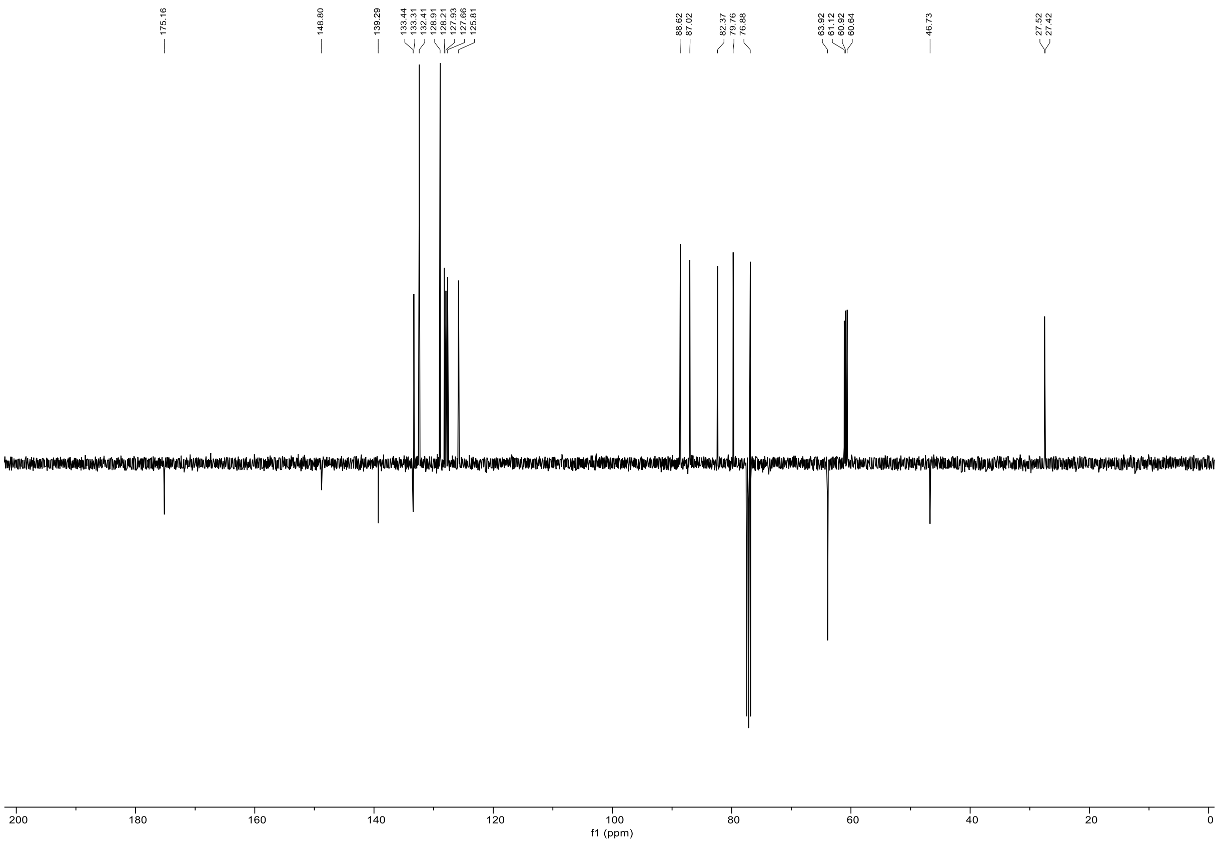

Supplementary Figure S100. $^{13}C\{^{1}H\}$ NMR, 101 MHz, $CDCl_3$ of compound **S17**

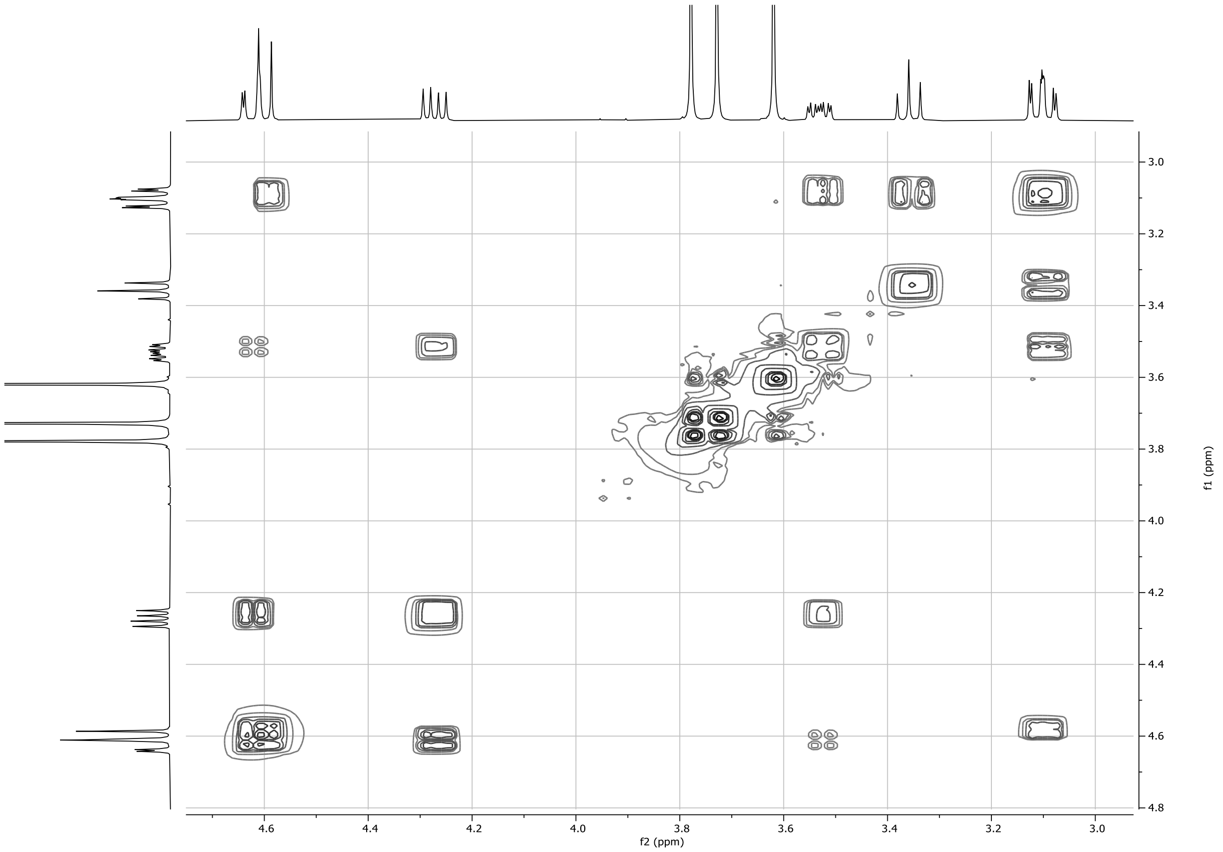

Supplementary Figure S101. HH-COSY NMR, $CDCl_3$ of compound **S17**

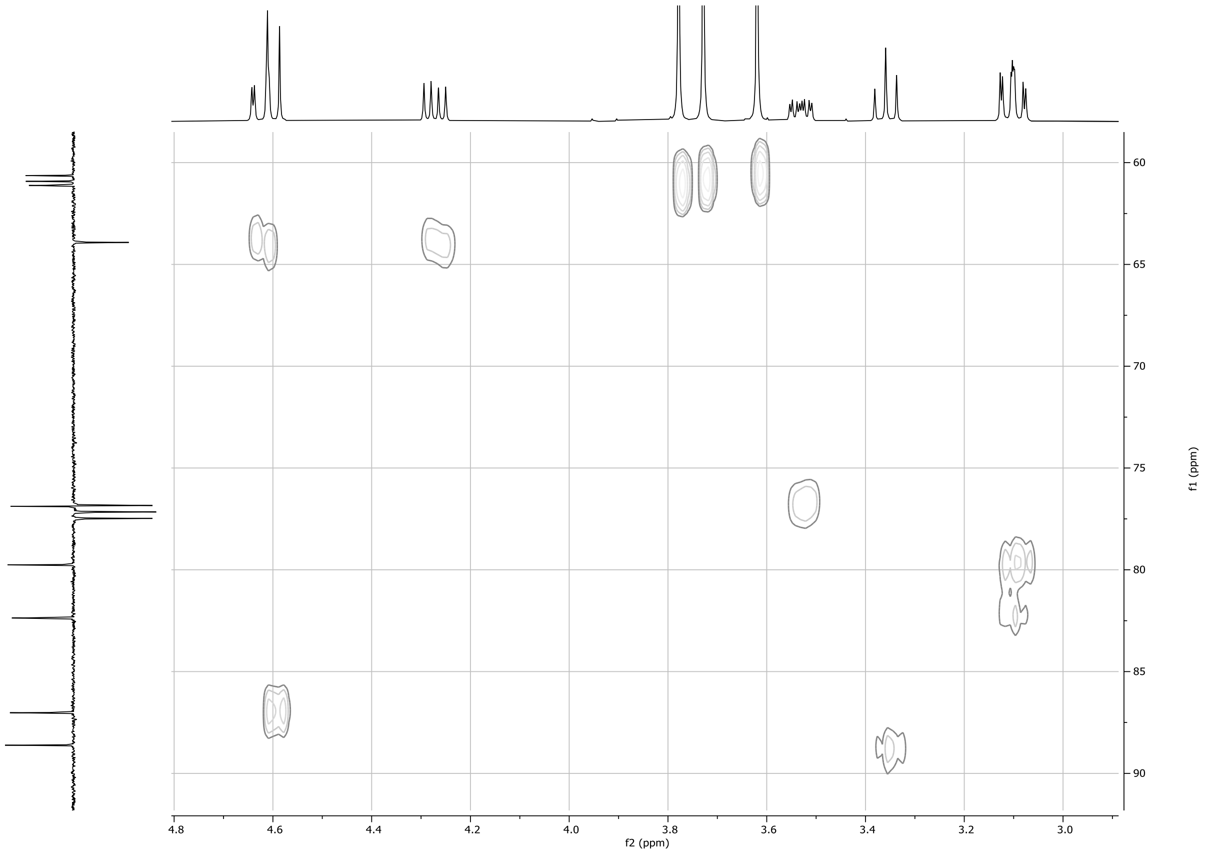

Supplementary Figure S102. HSQC{^{1}H} NMR, $CDCl_3$ of compound **S17**

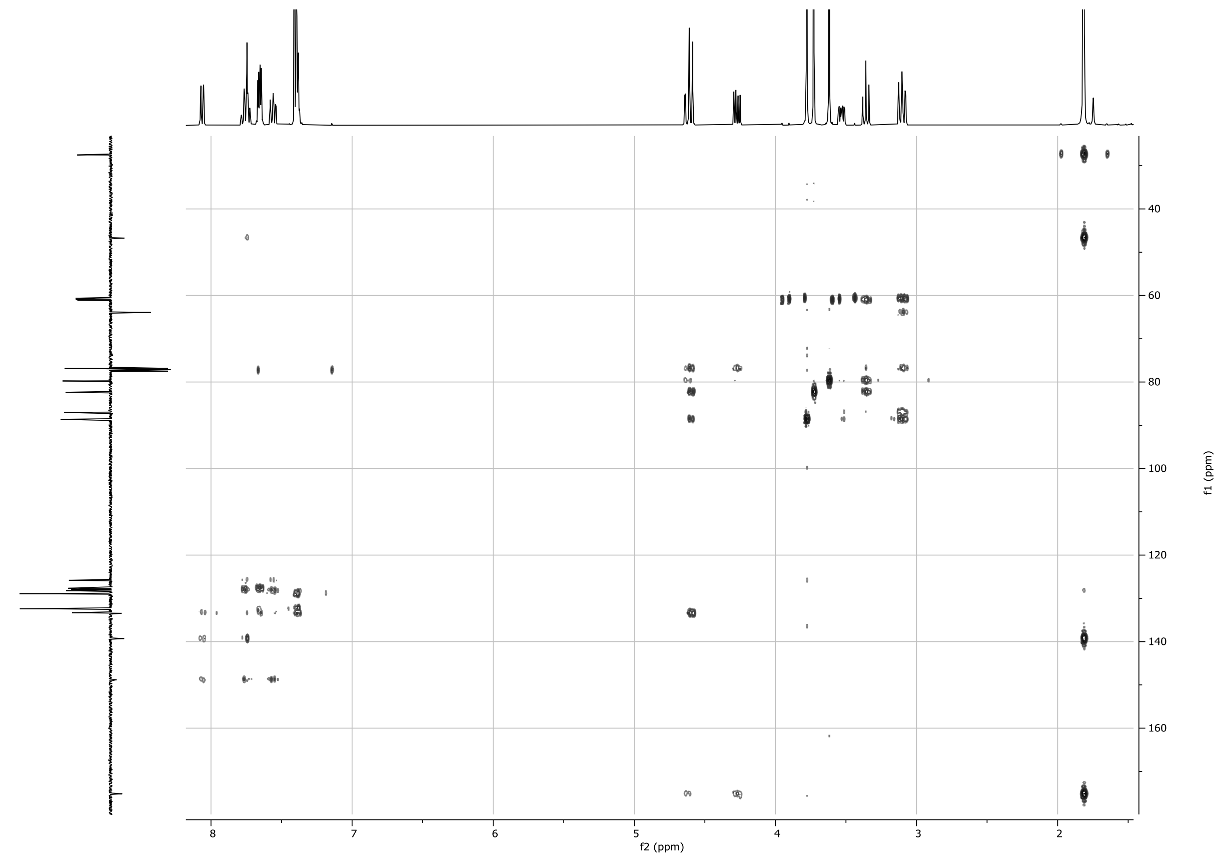

Supplementary Figure S103. HMBC{^{1}H} NMR, $CDCl_3$ of compound **S17**

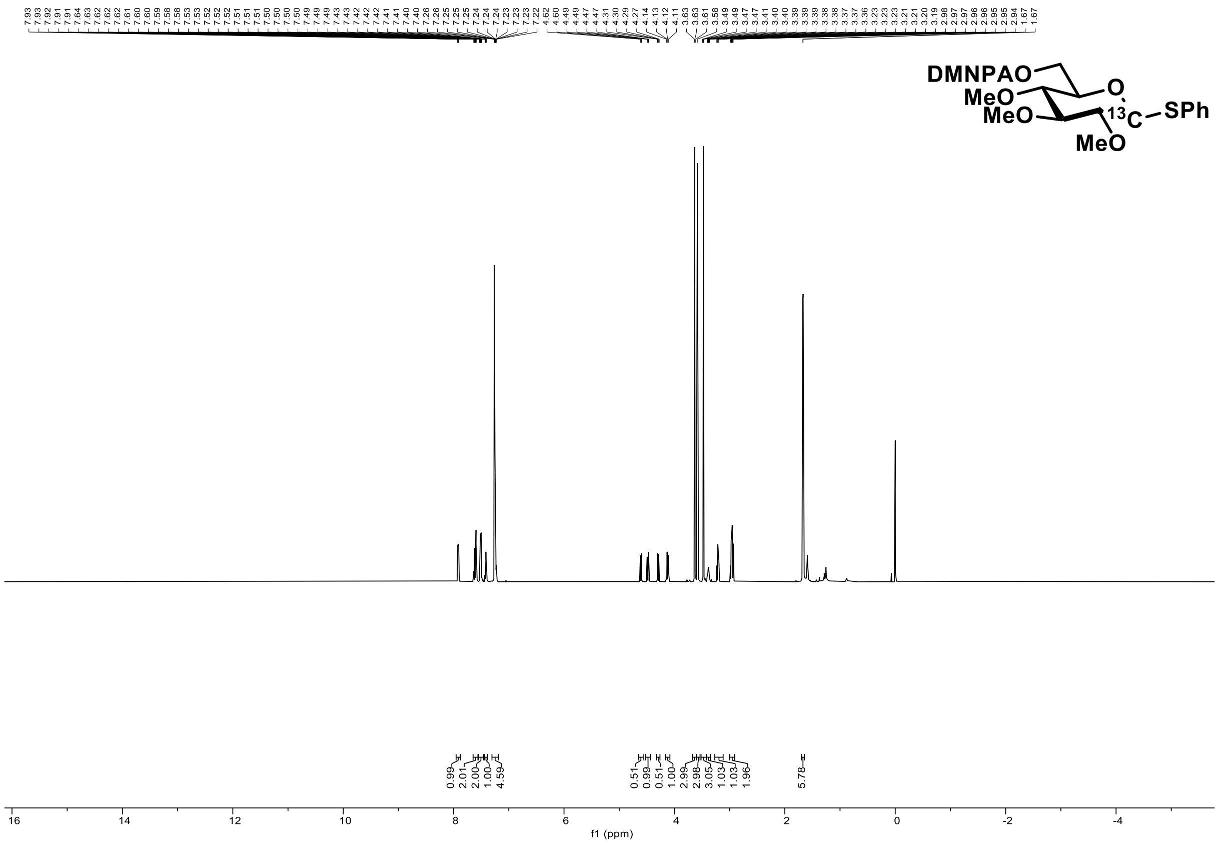

Supplementary Figure S104. ^{1}H NMR, 500 MHz, $CDCl_3$ of compound **S18**

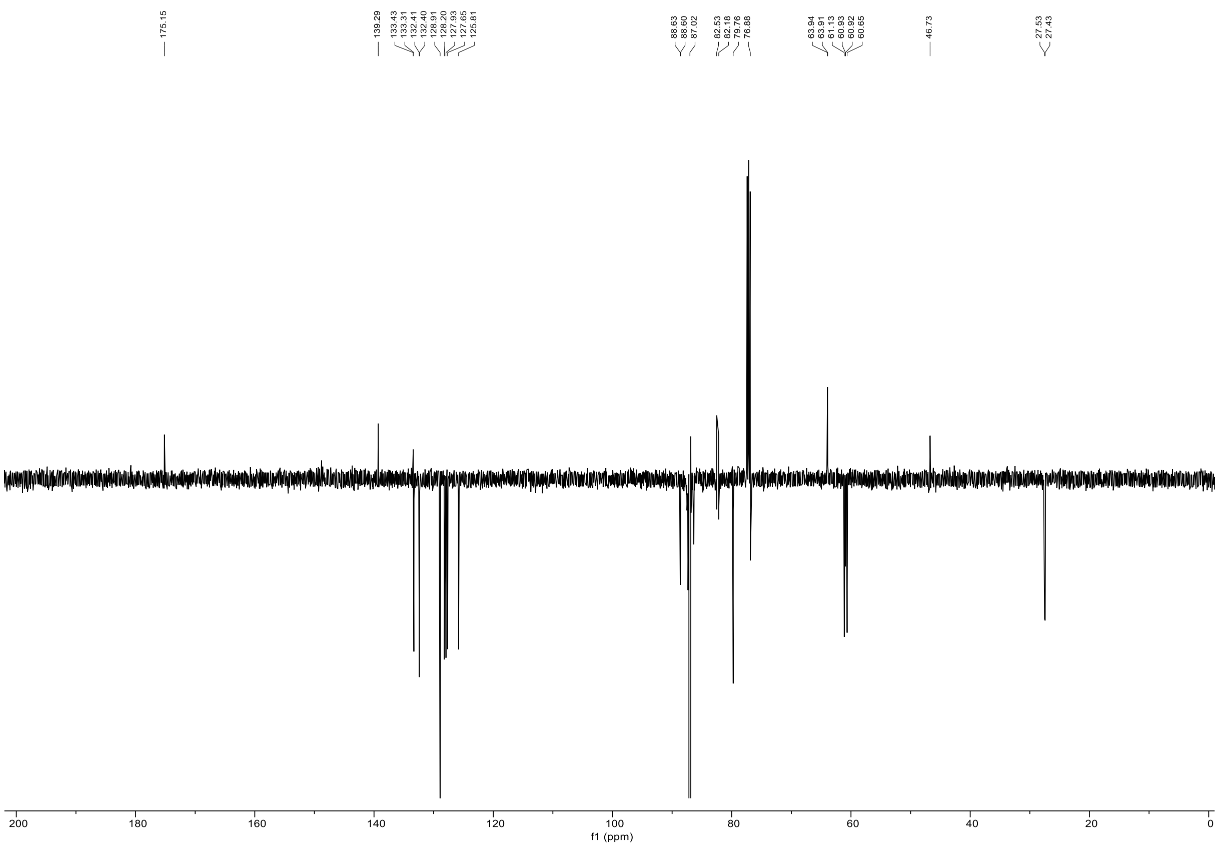

Supplementary Figure S105. $^{13}C\{^{1}H\}$ NMR, 126 MHz, $CDCl_3$ of compound **S18**

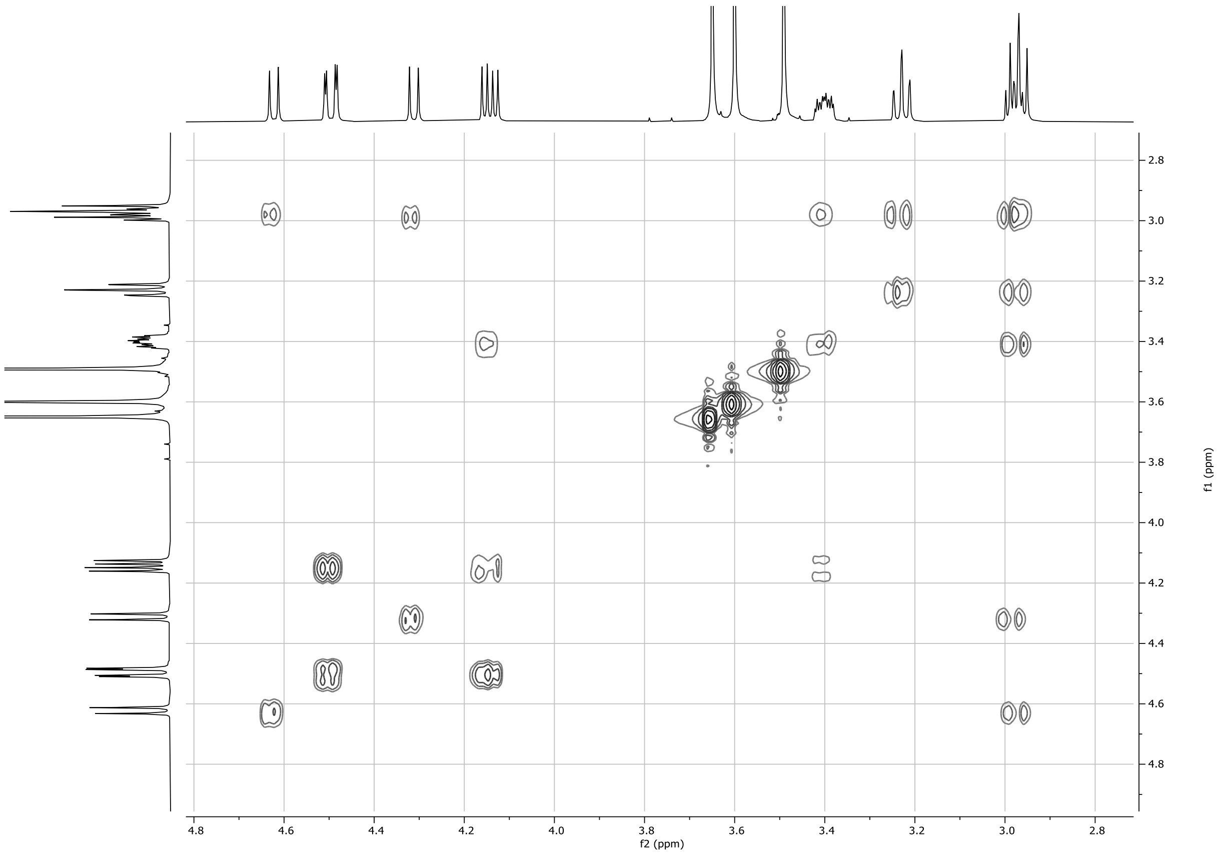

Supplementary Figure S106. HH-COSY NMR, $CDCl_3$ of compound **S18**

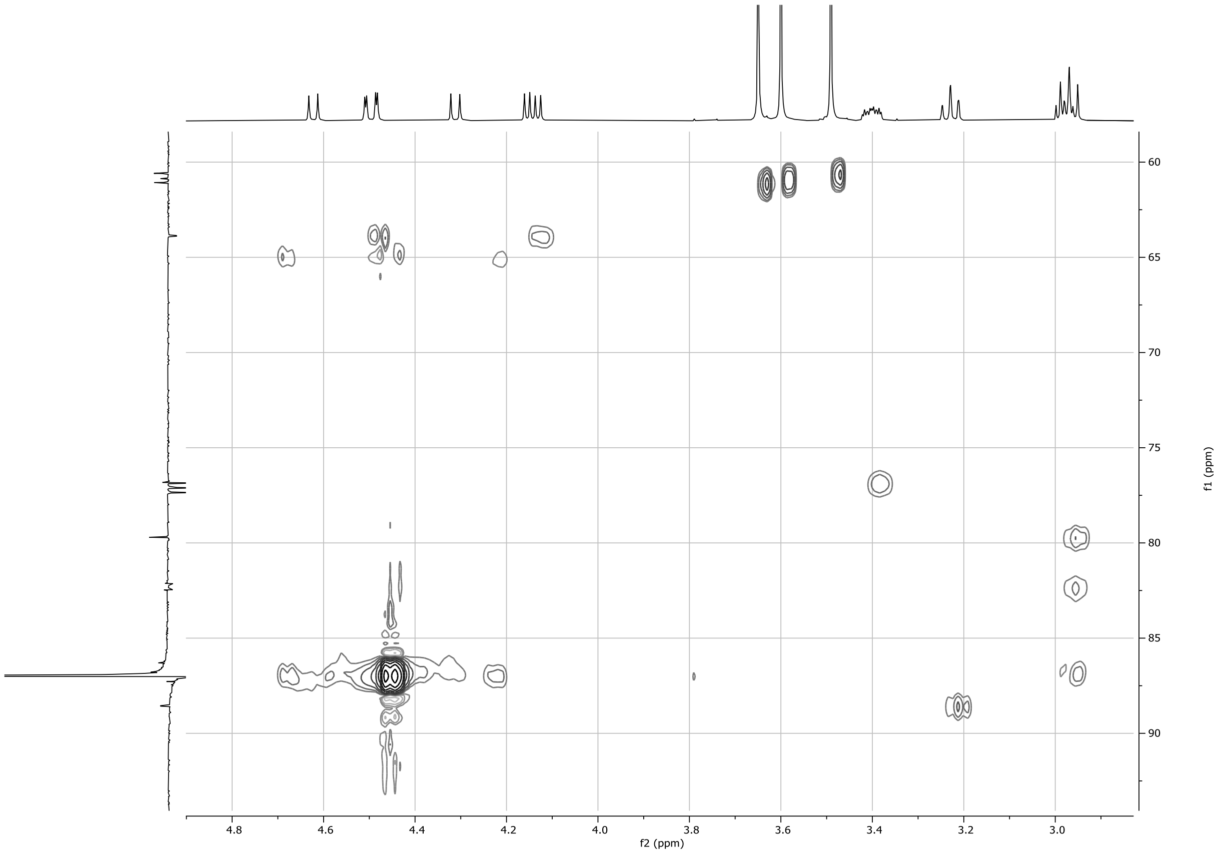

Supplementary Figure S107. HSQC{^{1}H} NMR, $CDCl_3$ of compound **S18**

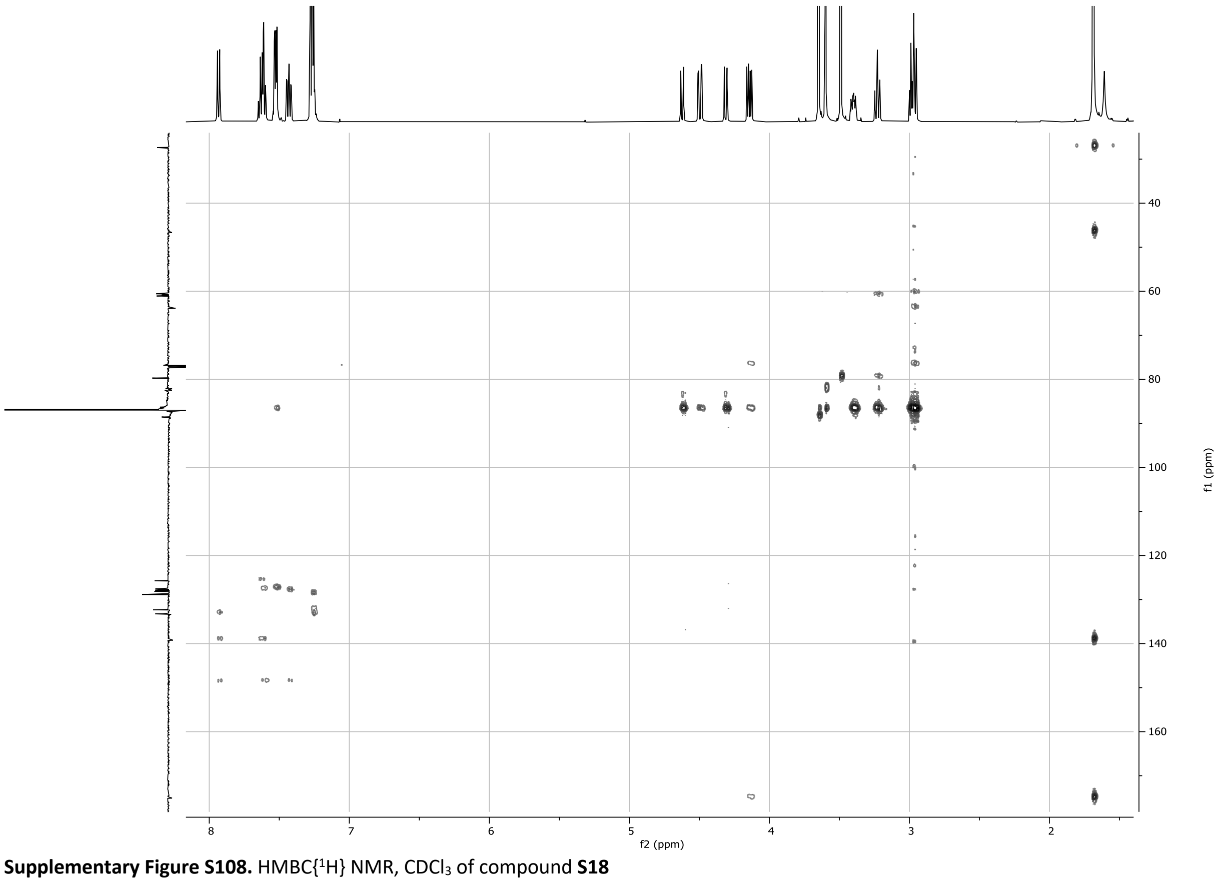

Supplementary Figure S108. HMBC{^{1}H} NMR, $CDCl_3$ of compound **S18**

DMNPA reagent and intermediates NMR spectra

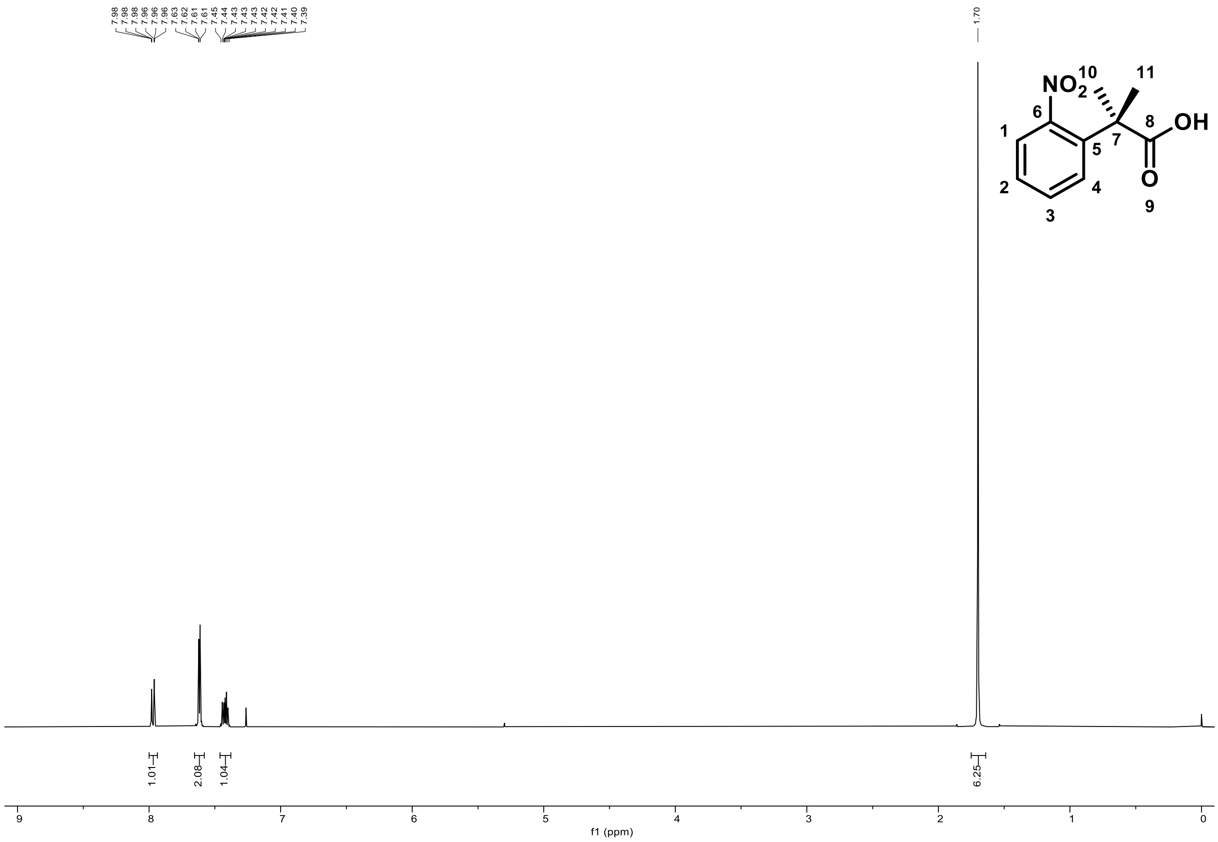

Supplementary Figure S109. ^{1}H NMR, 400 MHz, $CDCl_3$ of compound **S19**

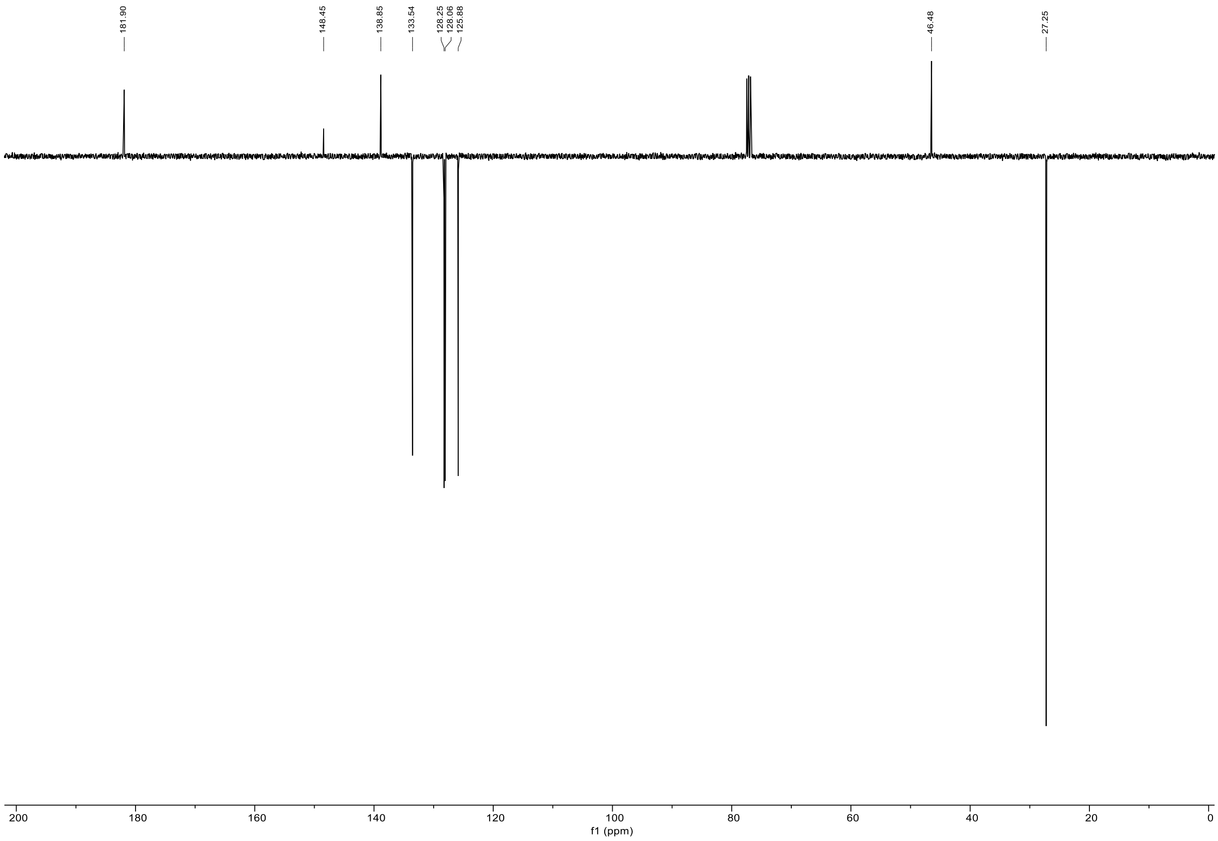

Supplementary Figure S110. $^{13}C\{^{1}H\}$ NMR, 101 MHz, $CDCl_3$ of compound **S19**

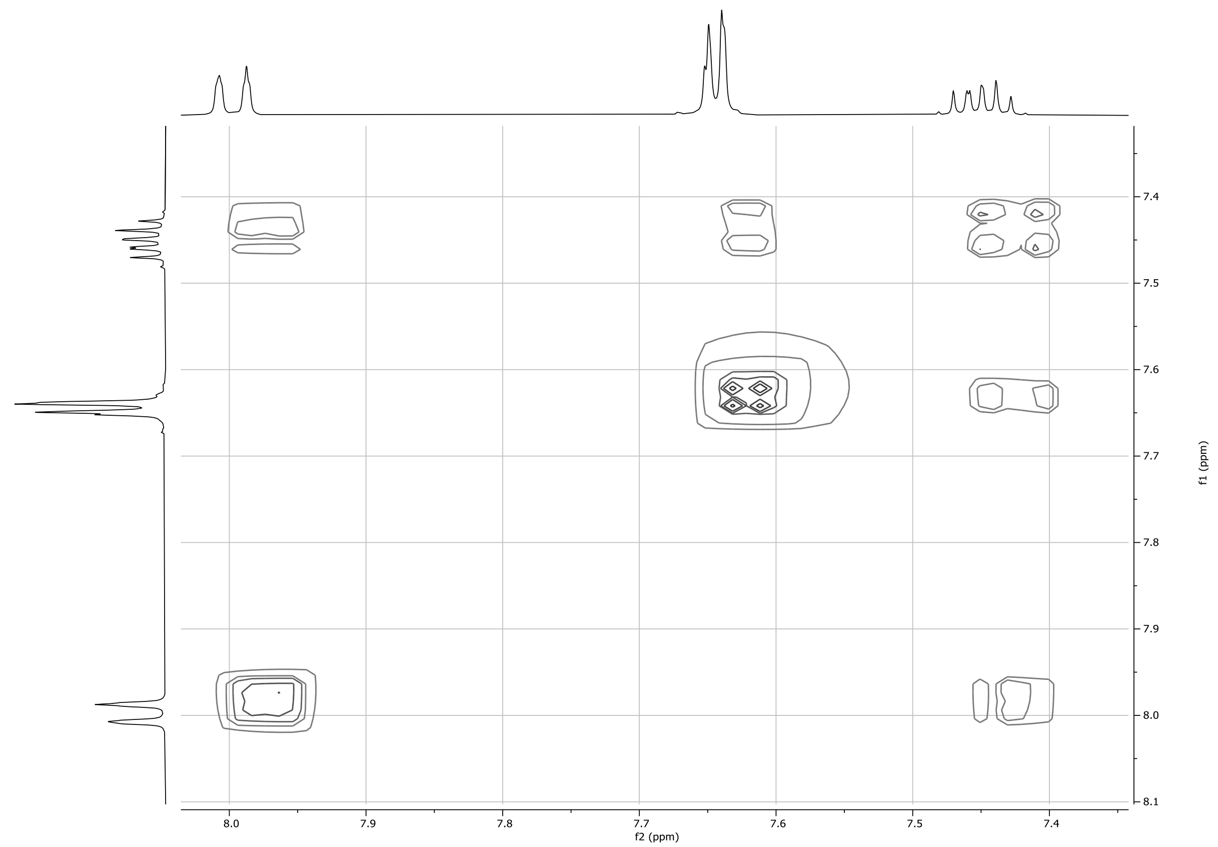

Supplementary Figure S111. HH-COSY NMR, $CDCl_3$ of compound **S19**

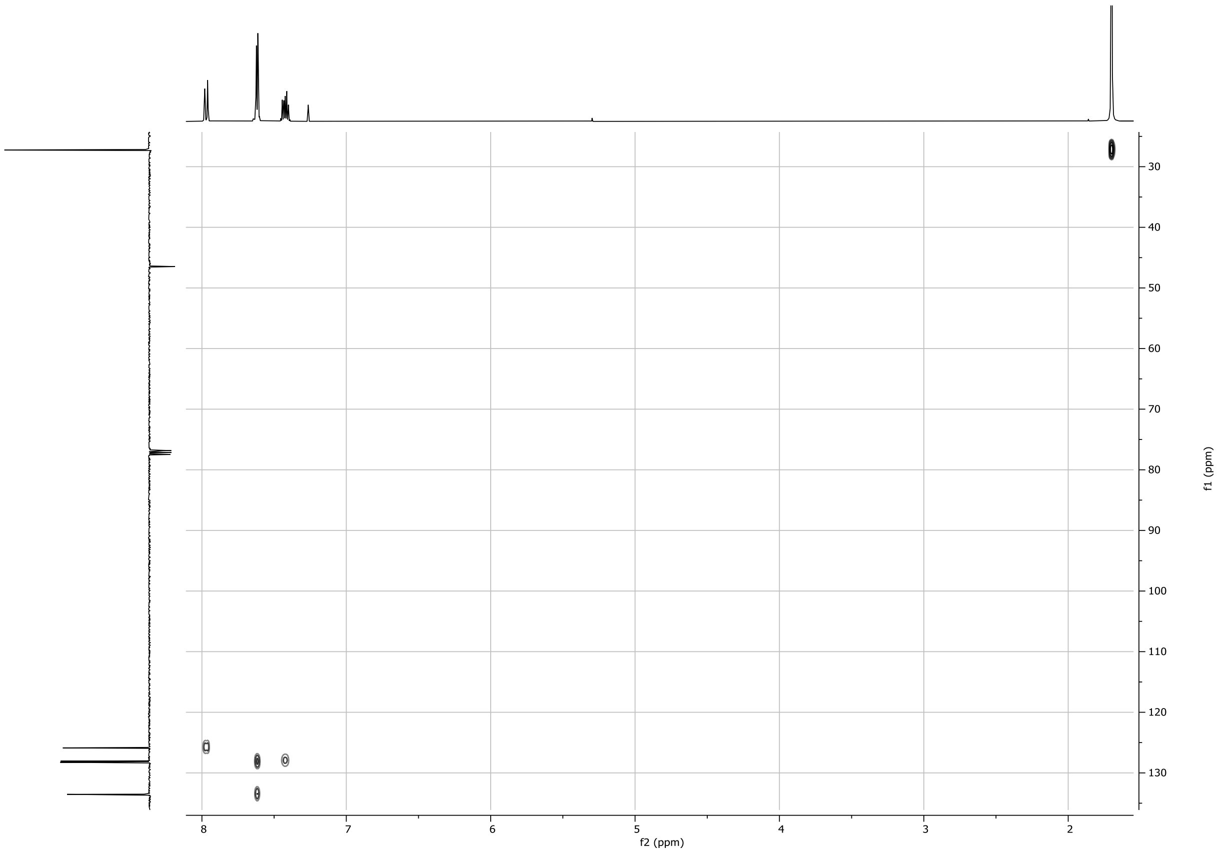

Supplementary Figure S112. HSQC{^1H} NMR, $CDCl_3$ of compound **S19**

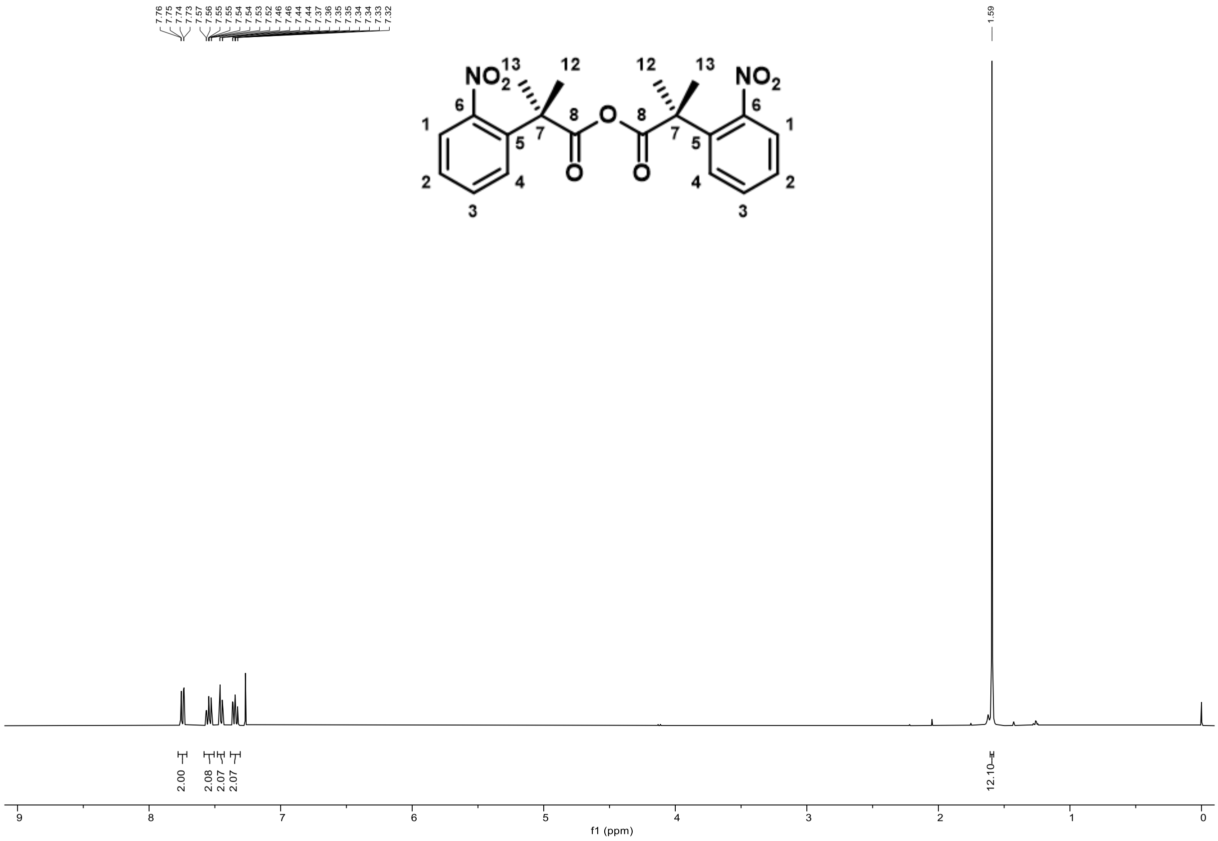

Supplementary Figure S113. ^{1}H NMR, 400 MHz, $CDCl_3$ of compound **S20**

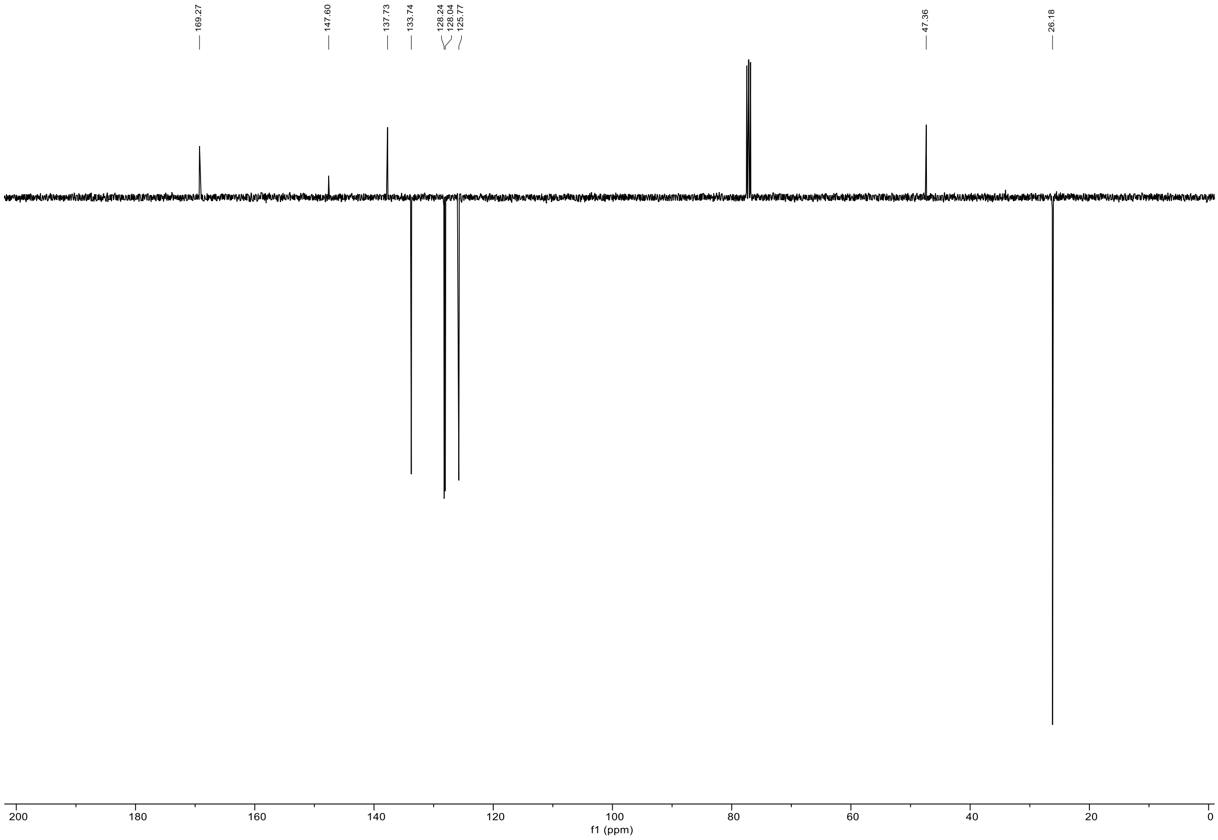

Supplementary Figure S114. $^{13}C\{^{1}H\}$ NMR, 101 MHz, $CDCl_3$ of compound **S20**

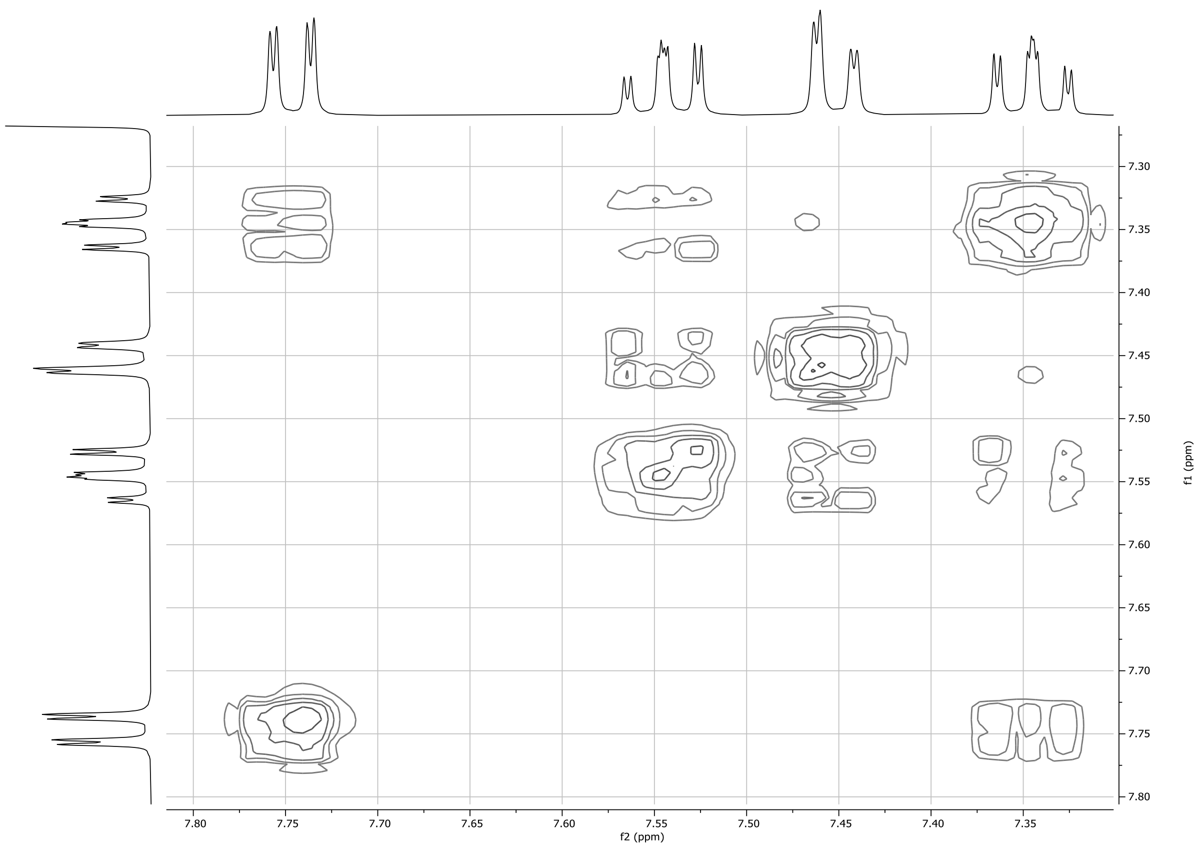

Supplementary Figure S115. HH-COSY NMR, $CDCl_3$ of compound **S20**

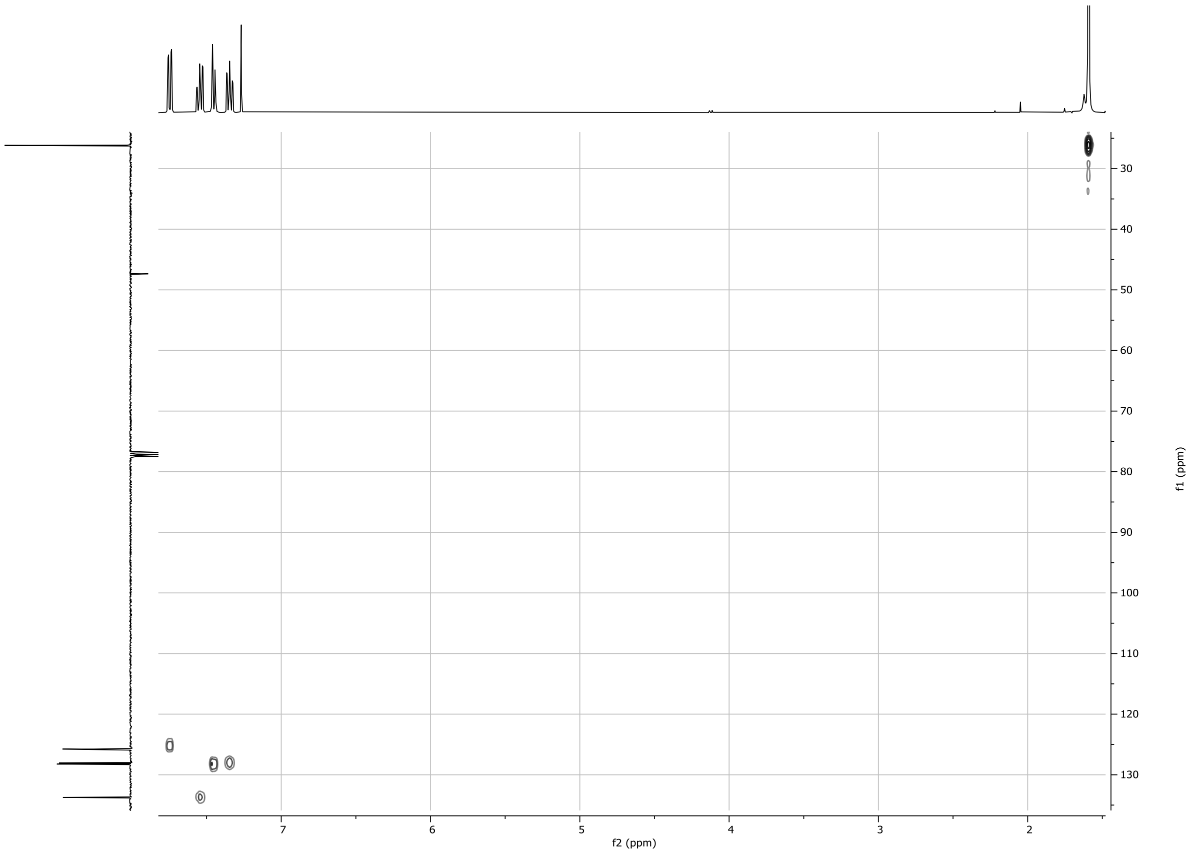

Supplementary Figure S116. HSQC{^{1}H} NMR, $CDCl_3$ of compound **S20**

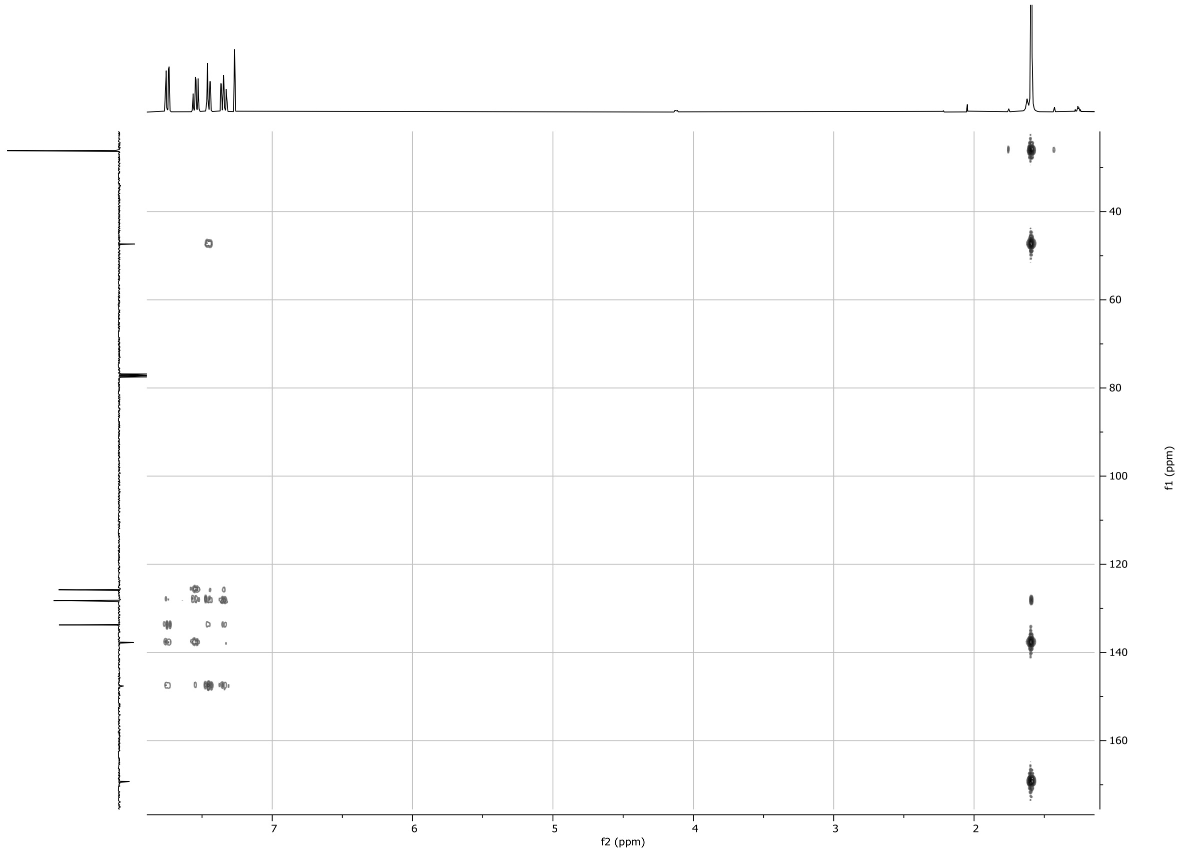

Supplementary Figure S117. HMBC{^{1}H} NMR, $CDCl_3$ of compound **S20**

Model glycosylation NMR spectra

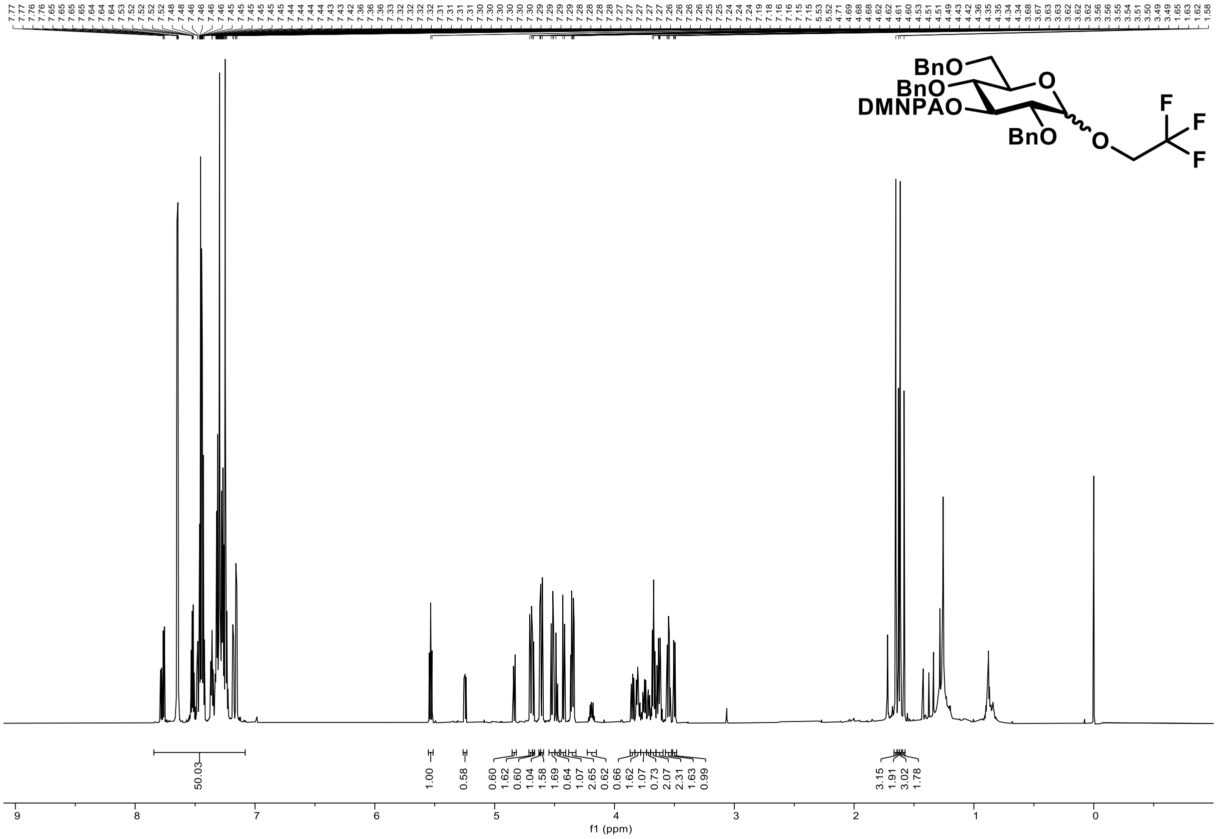

Supplementary Figure S118. ^{1}H NMR, 850 MHz, $CDCl_3$ of compound **S21**

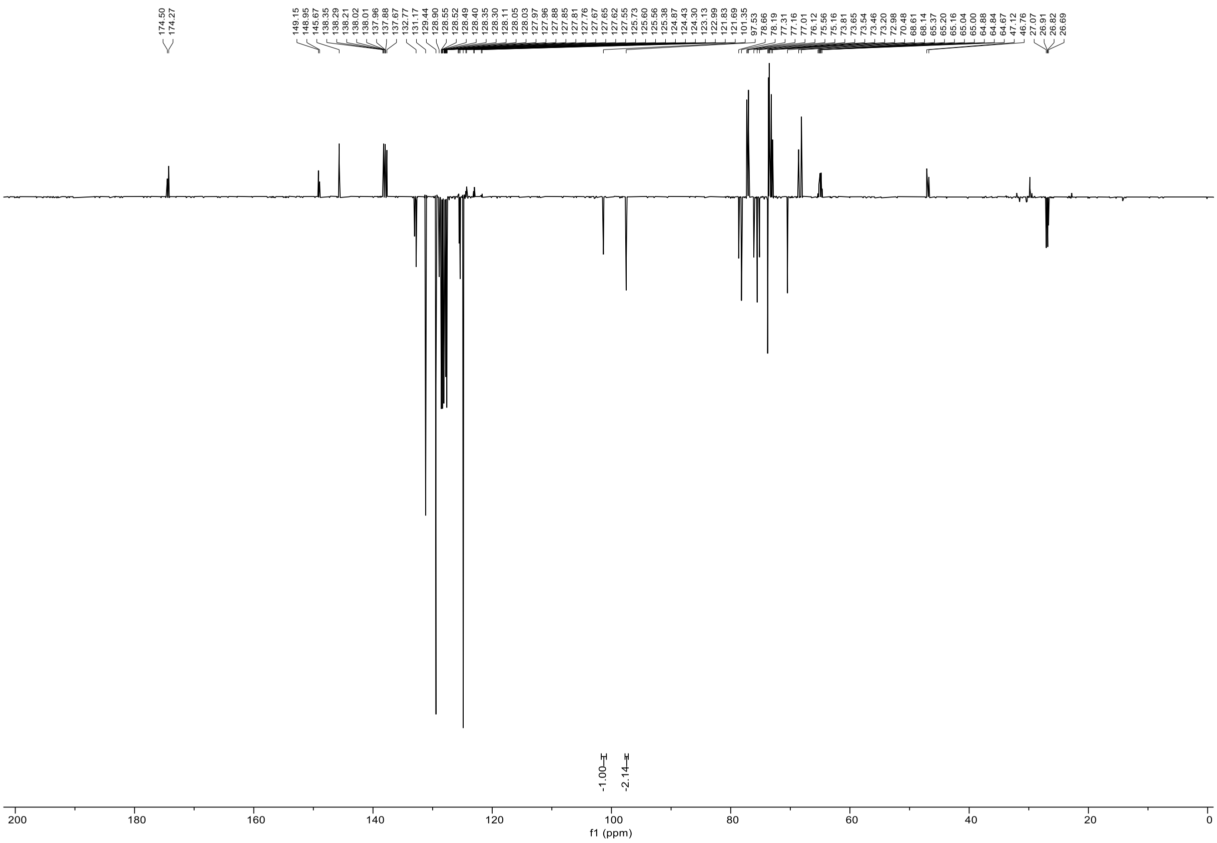

Supplementary Figure S119. $^{13}C\{^{1}H\}$ NMR, 214 MHz, $CDCl_3$ of compound **S21**

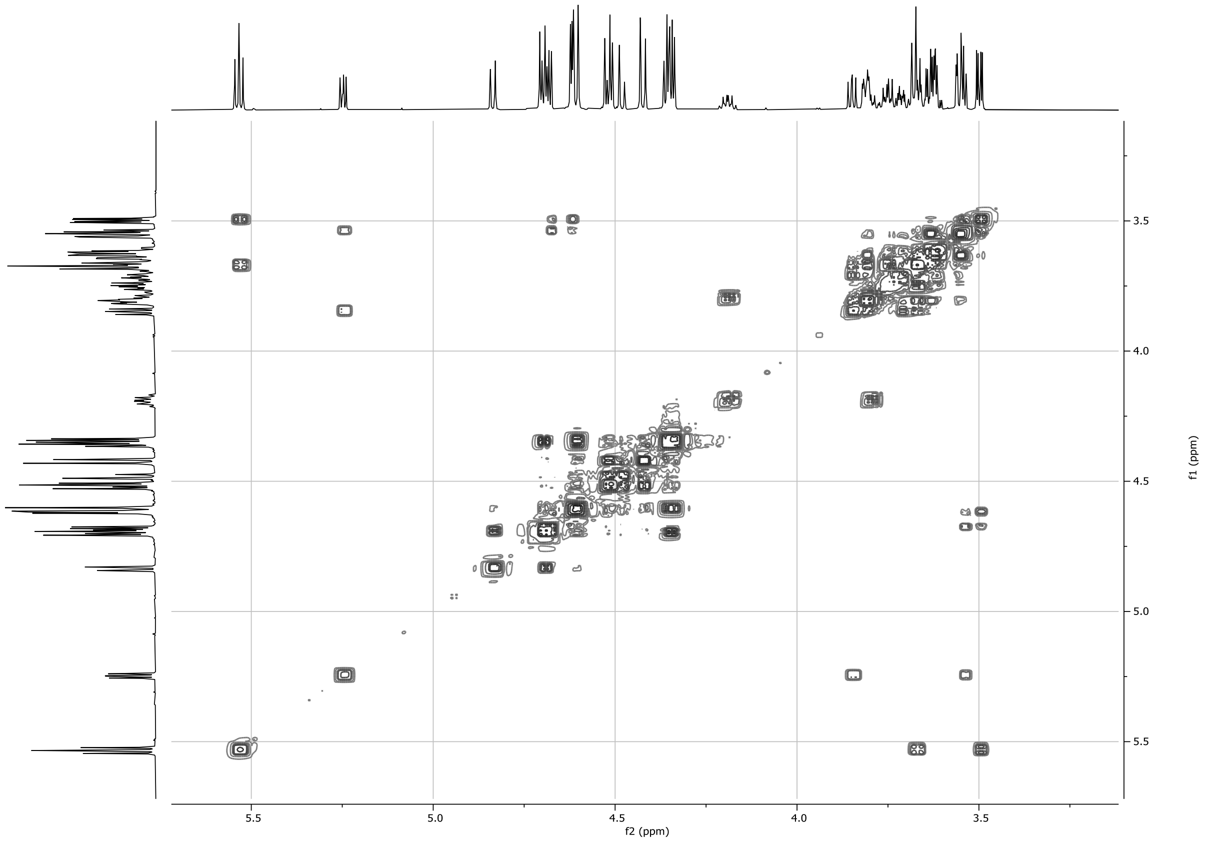

Supplementary Figure S120. HH-COSY NMR, $CDCl_3$ of compound **S21**

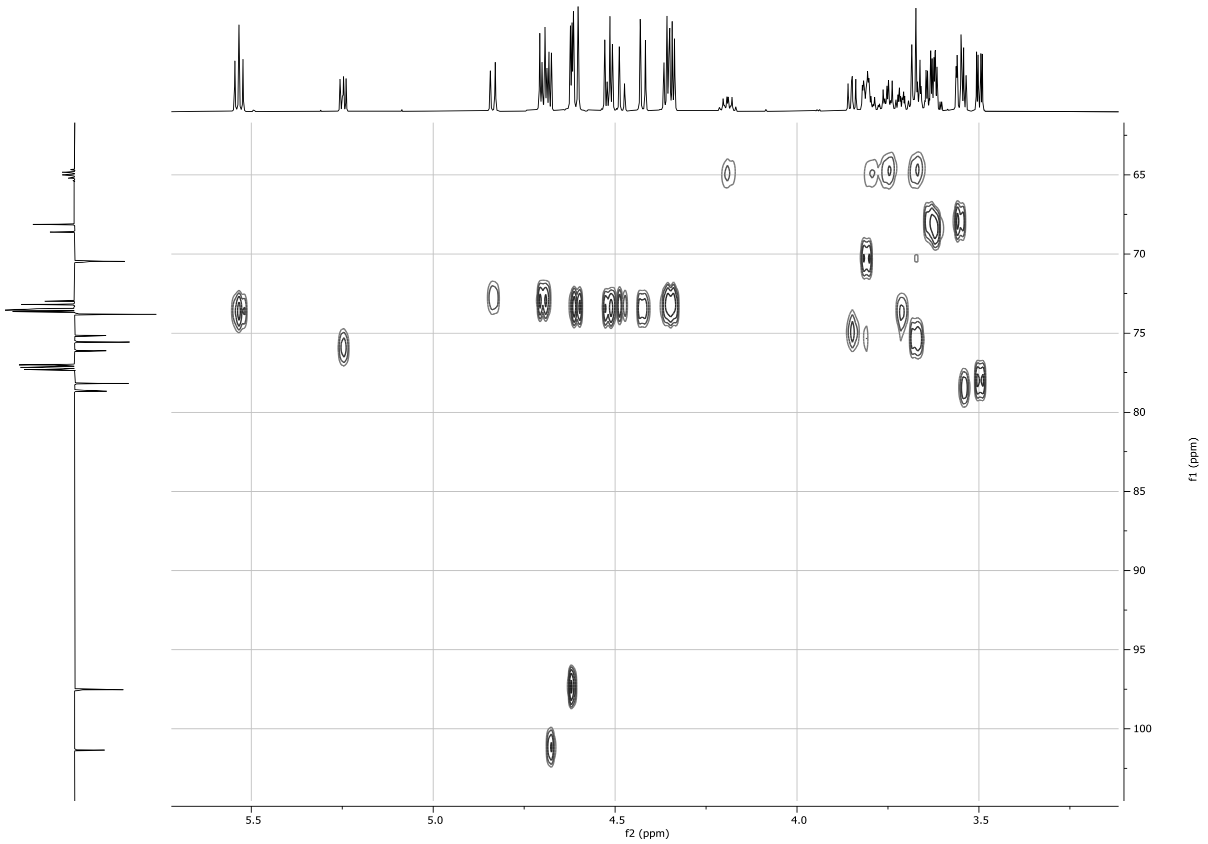

Supplementary Figure S121. HSQC{^{1}H} NMR, $CDCl_3$ of compound **S21**

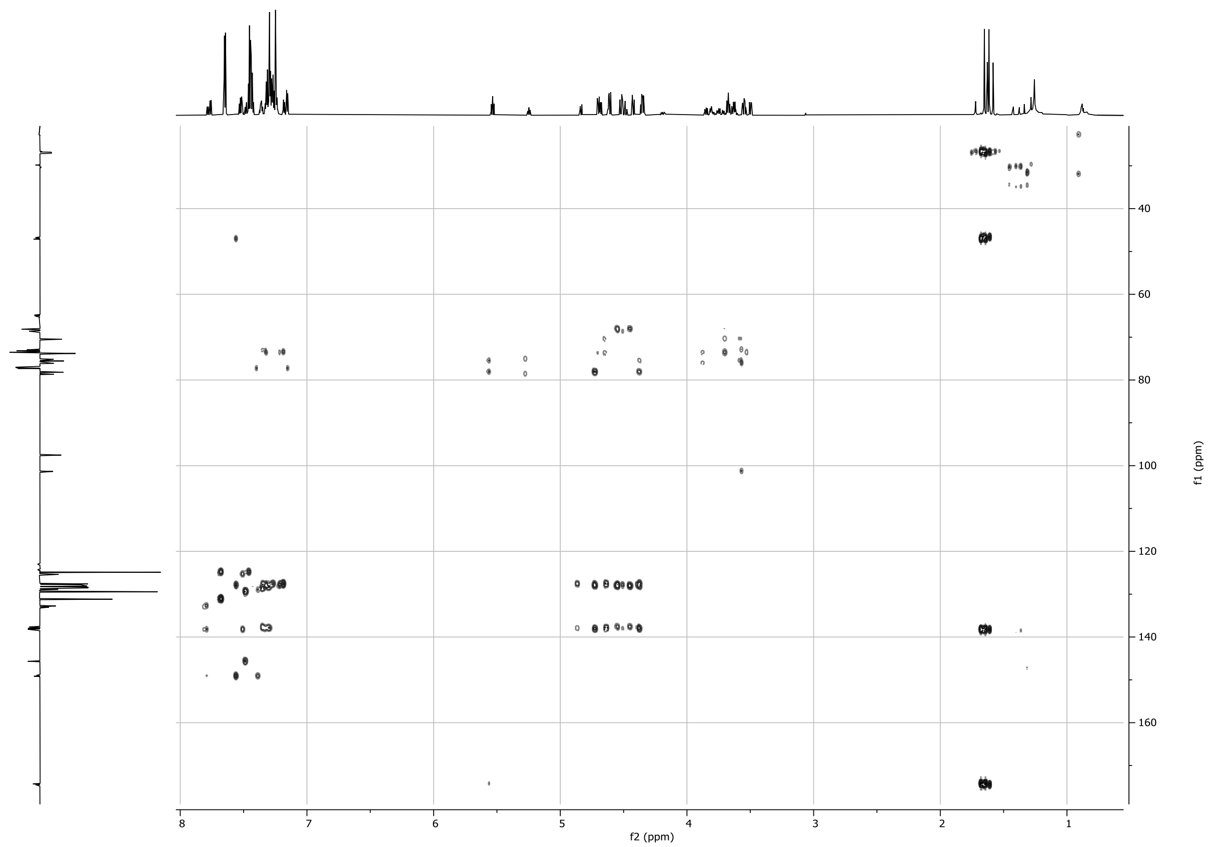

Supplementary Figure S122. HMBC{^{1}H} NMR, $CDCl_3$ of compound **S21**

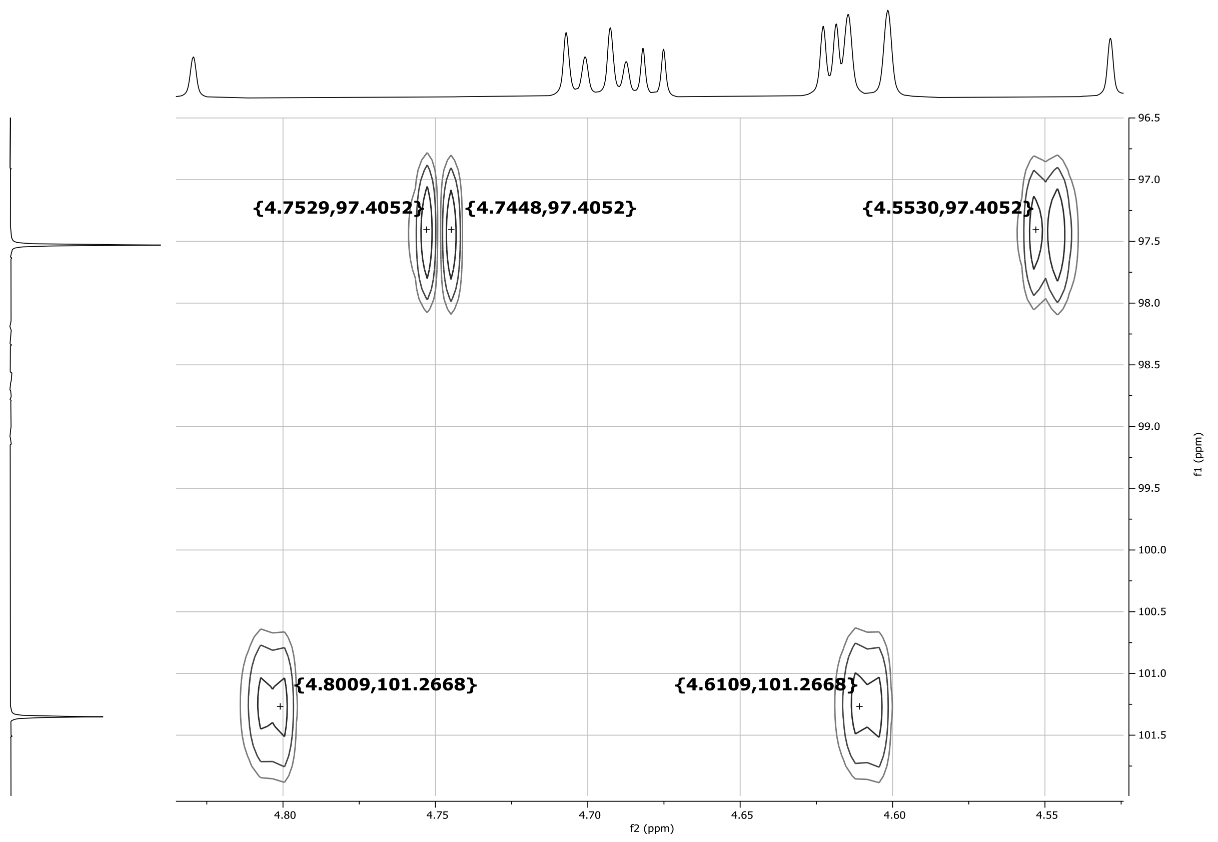

Supplementary Figure S123. HMBC-Gated NMR, $CDCl_3$ of compound **S21**

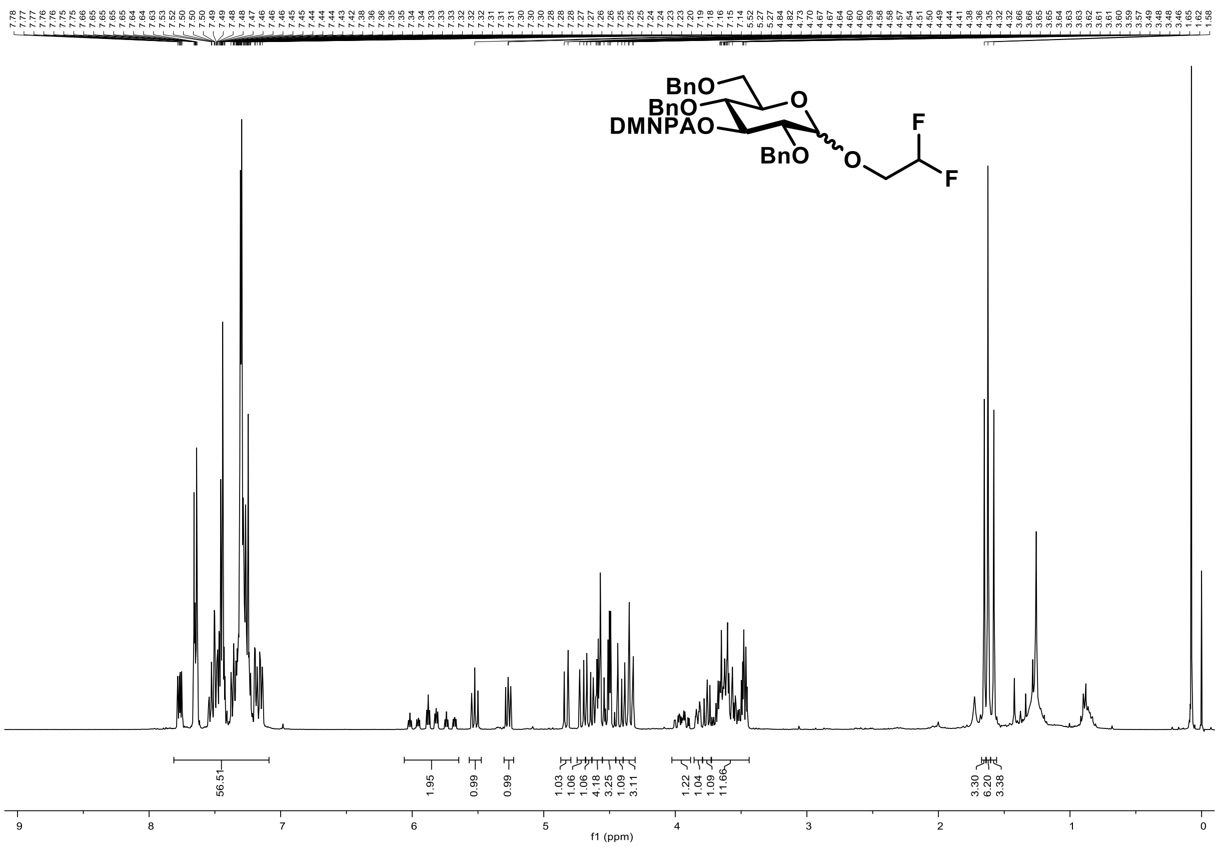

Supplementary Figure S124. ^{1}H NMR, 400 MHz, $CDCl_3$ of compound **S22**

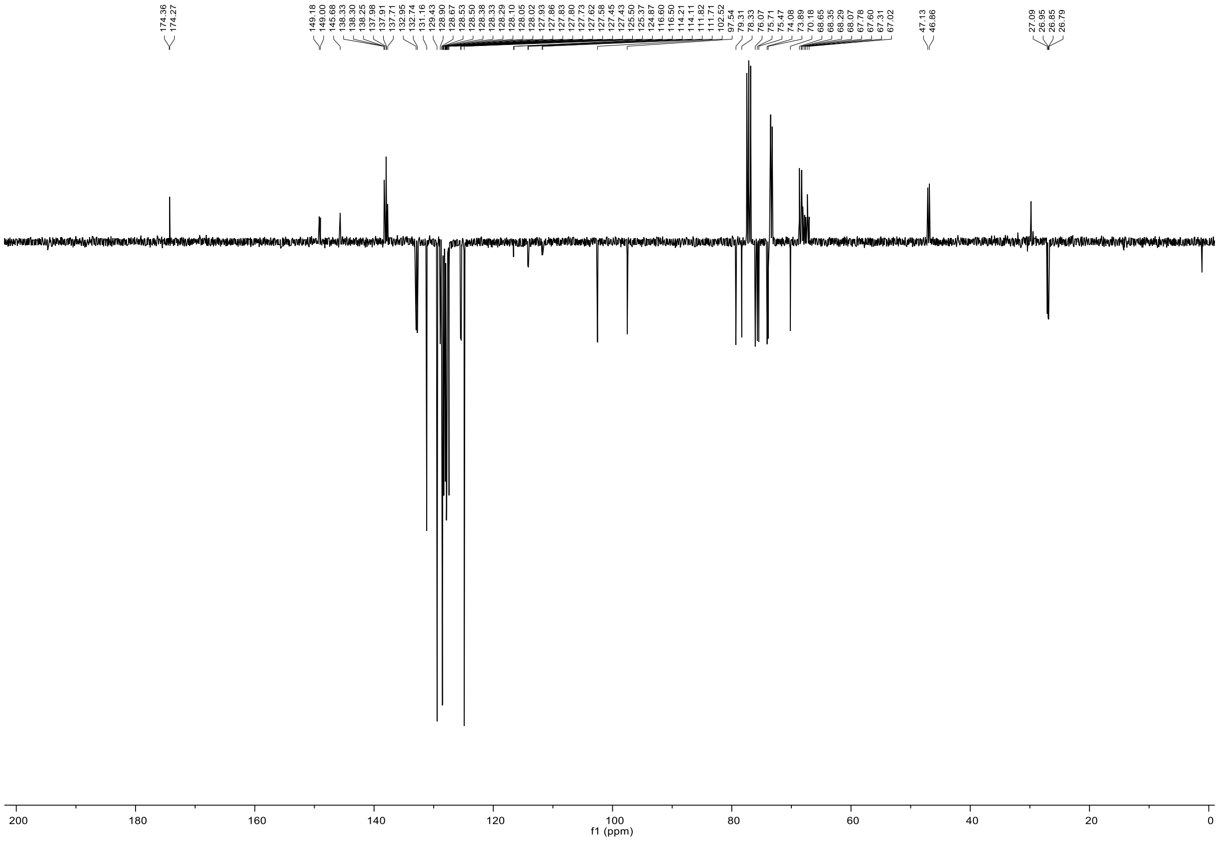

Supplementary Figure S125. $^{13}C\{^{1}H\}$ NMR, 101 MHz, $CDCl_3$ of compound **S22**

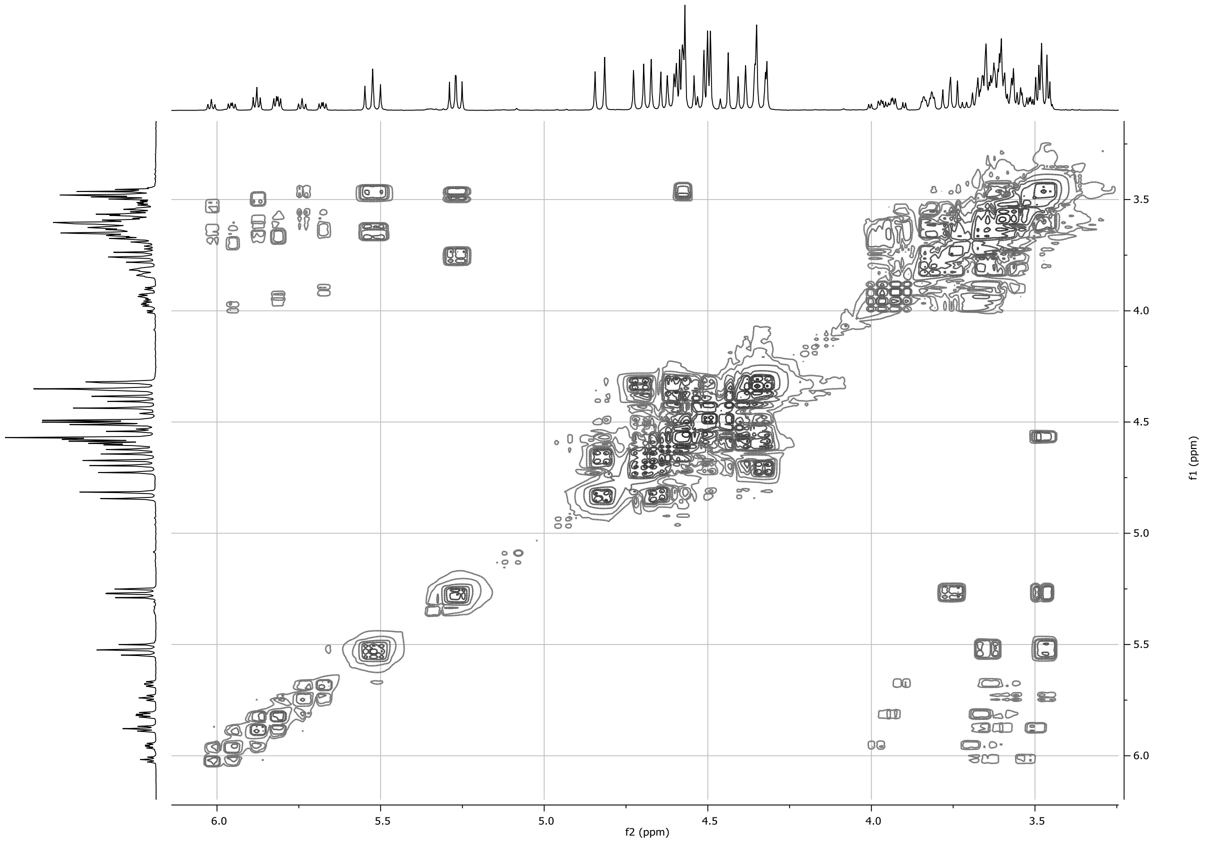

Supplementary Figure S126. HH-COSY NMR, $CDCl_3$ of compound **S22**

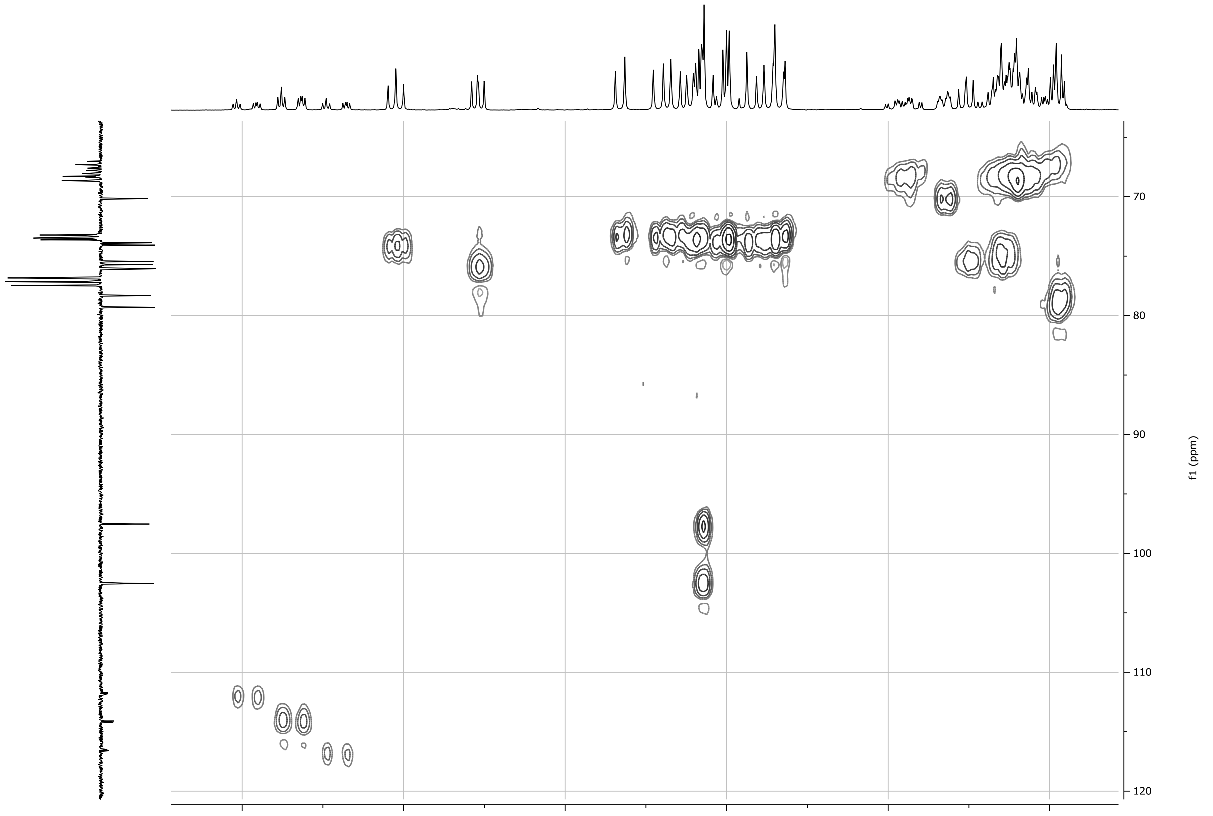

Supplementary Figure S127. HSQC{^{1}H} NMR, $CDCl_3$ of compound **S22**

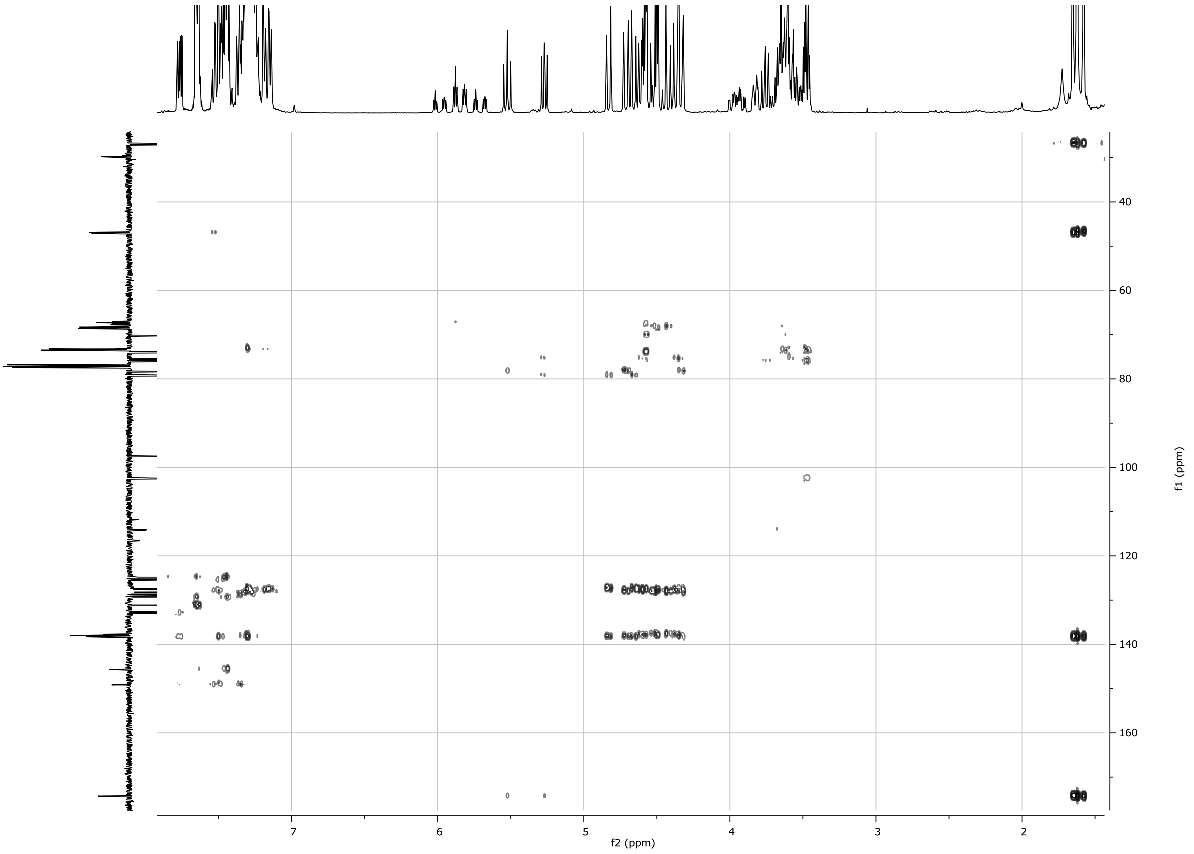

Supplementary Figure S128. HMBC{^{1}H} NMR, $CDCl_3$ of compound **S22**

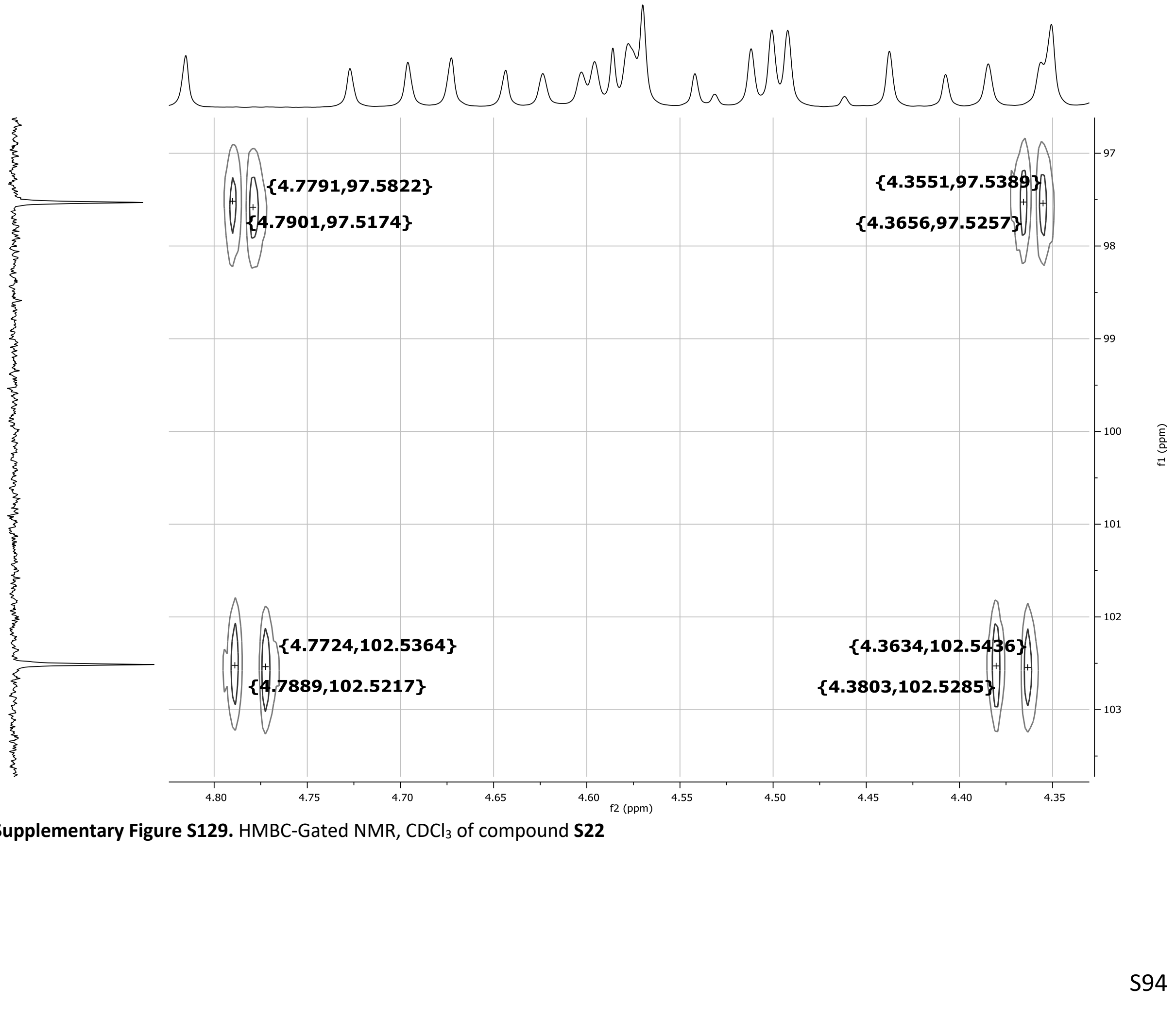

Supplementary Figure S129. HMBC-Gated NMR, $CDCl_3$ of compound **S22**

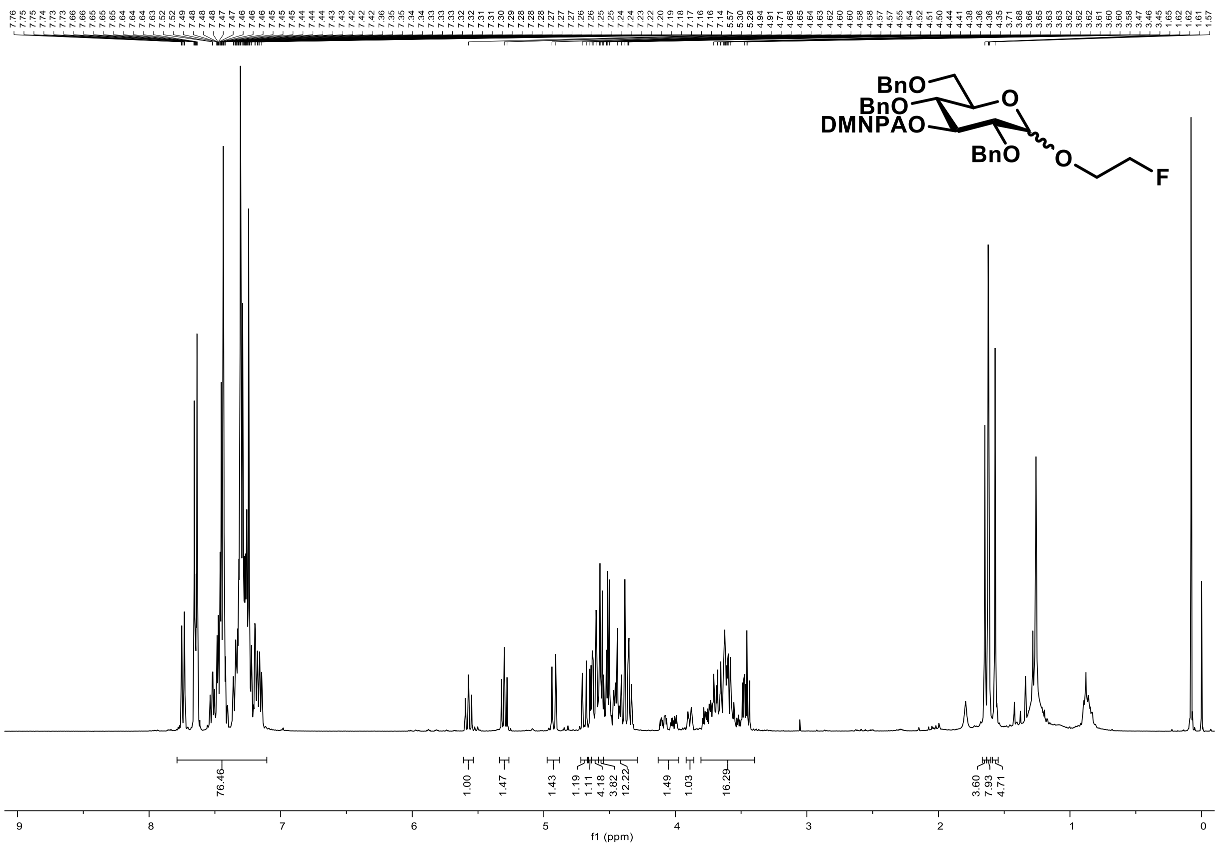

Supplementary Figure S130. ^{1}H NMR, 400 MHz, $CDCl_3$ of compound **S23**

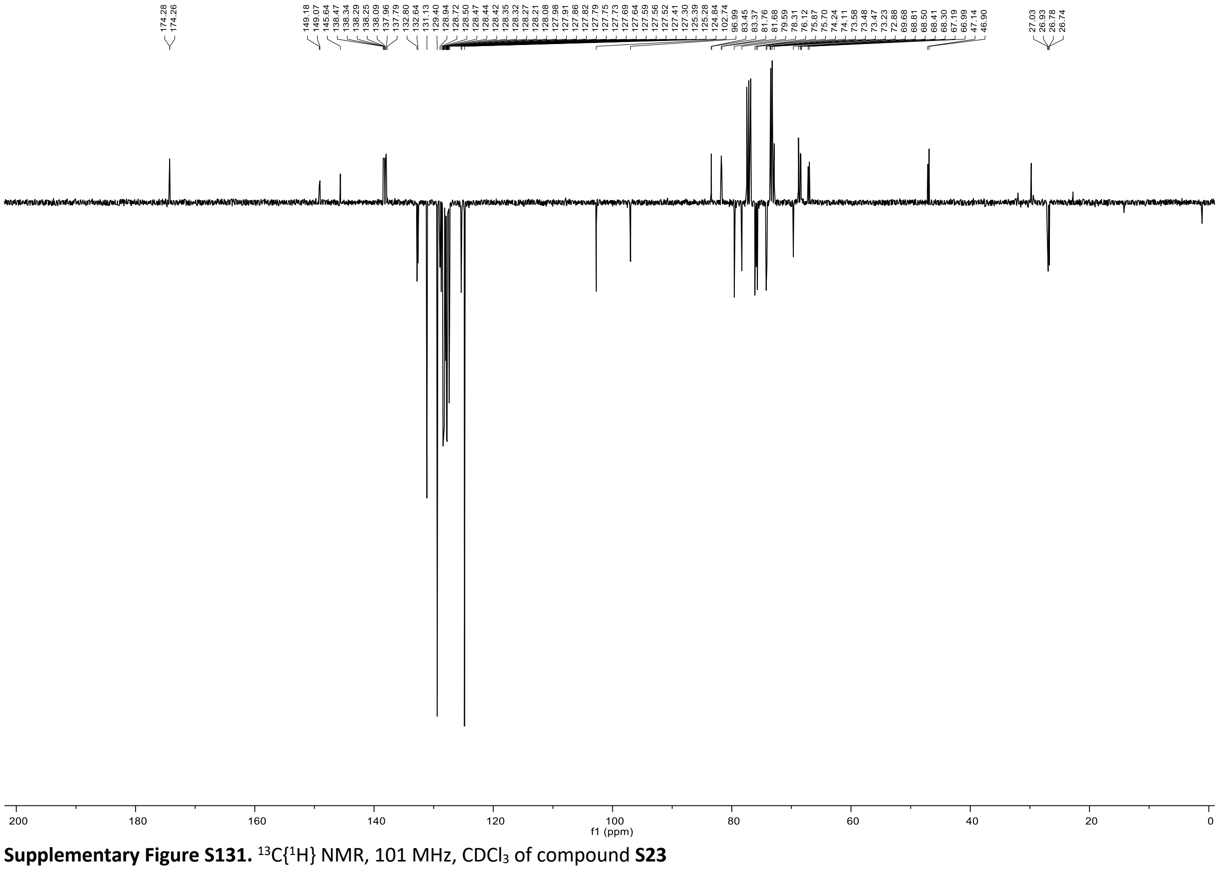

Supplementary Figure S131. $^{13}C\{^{1}H\}$ NMR, 101 MHz, $CDCl_3$ of compound **S23**

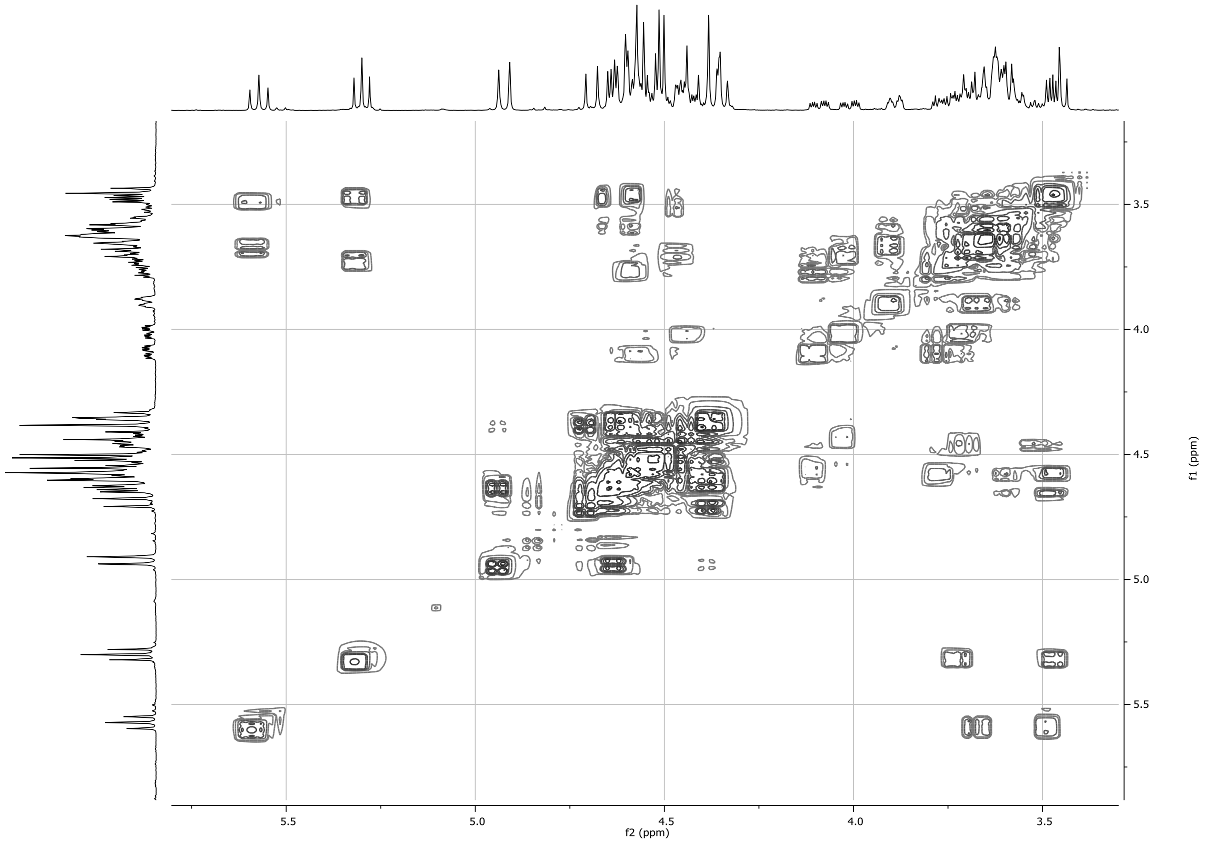

Supplementary Figure S132. HH-COSY NMR, $CDCl_3$ of compound **S23**

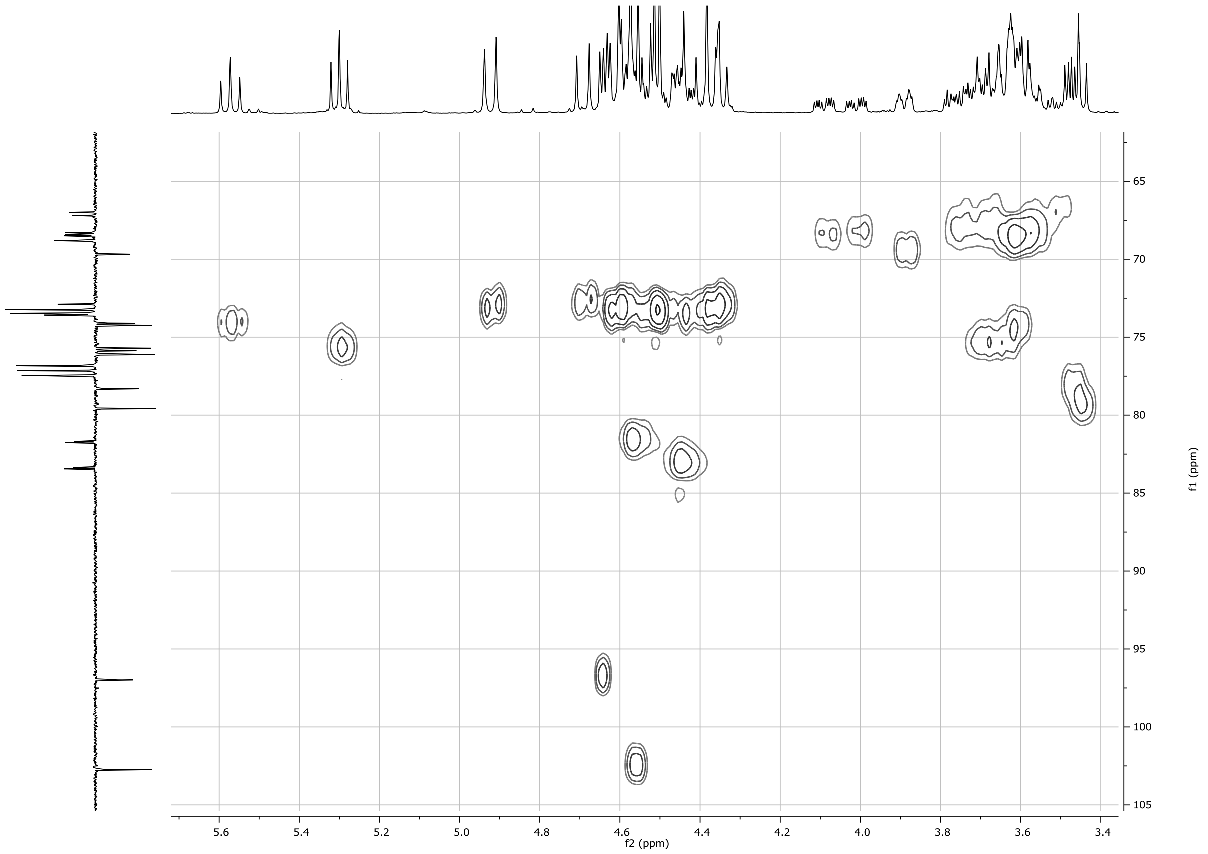

Supplementary Figure S133. HSQC{^{1}H} NMR, $CDCl_3$ of compound **S23**

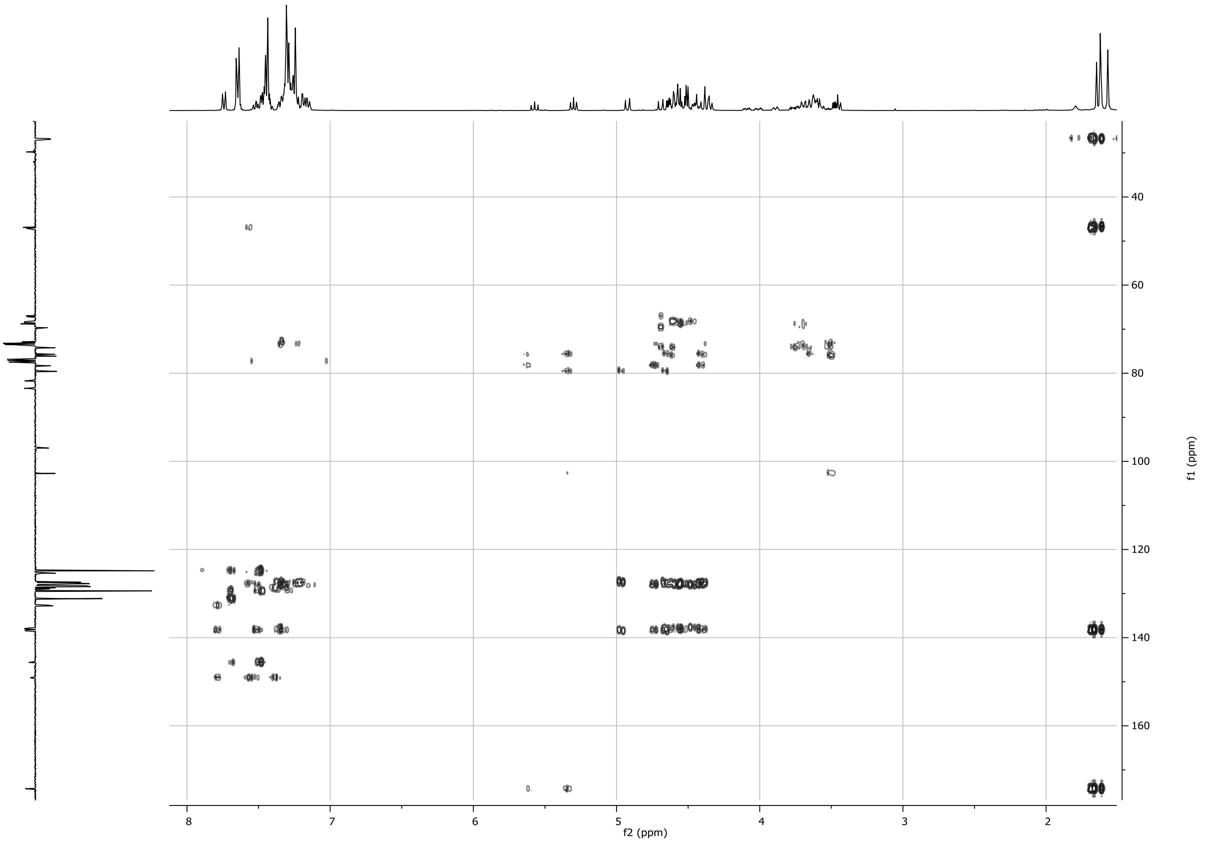

Supplementary Figure S134. HMBC{^{1}H} NMR, $CDCl_3$ of compound **S23**

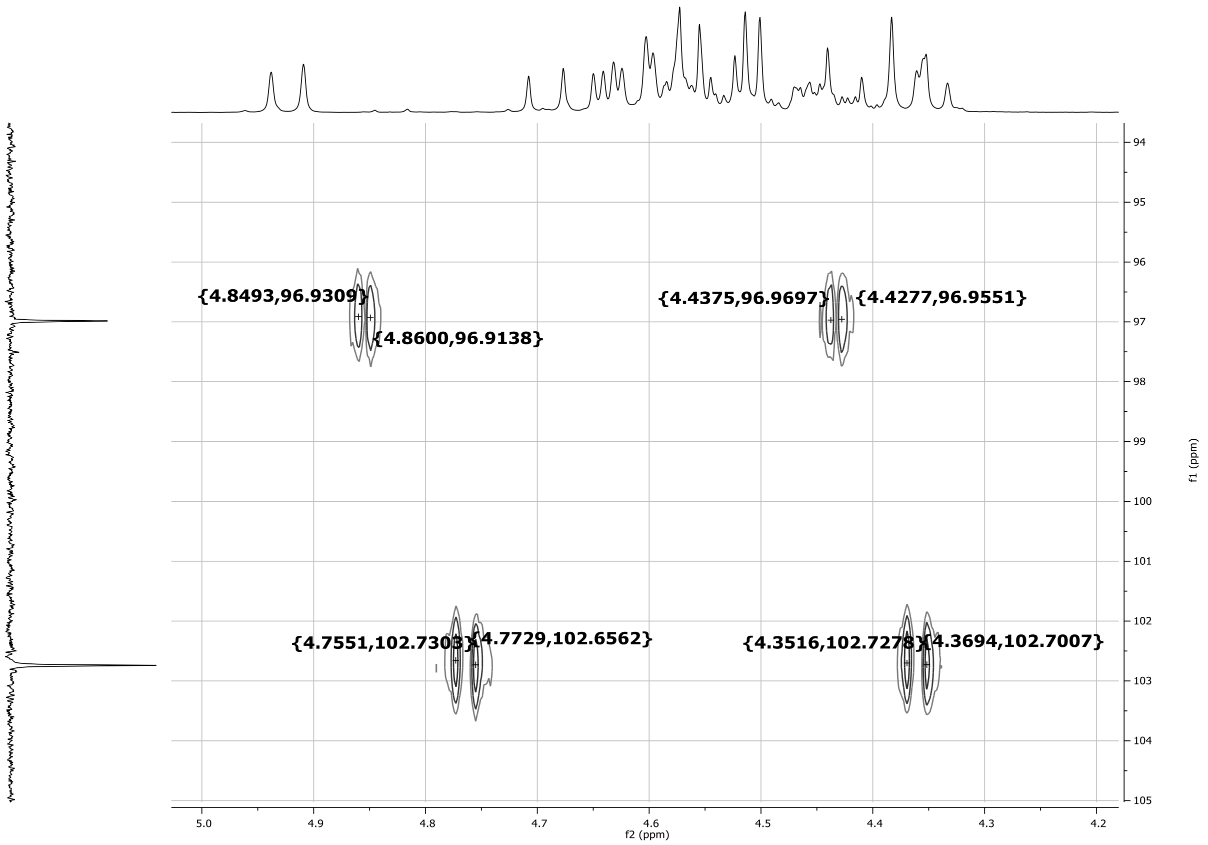

Supplementary Figure S135. HMBC-Gated NMR, $CDCl_3$ of compound **S23**

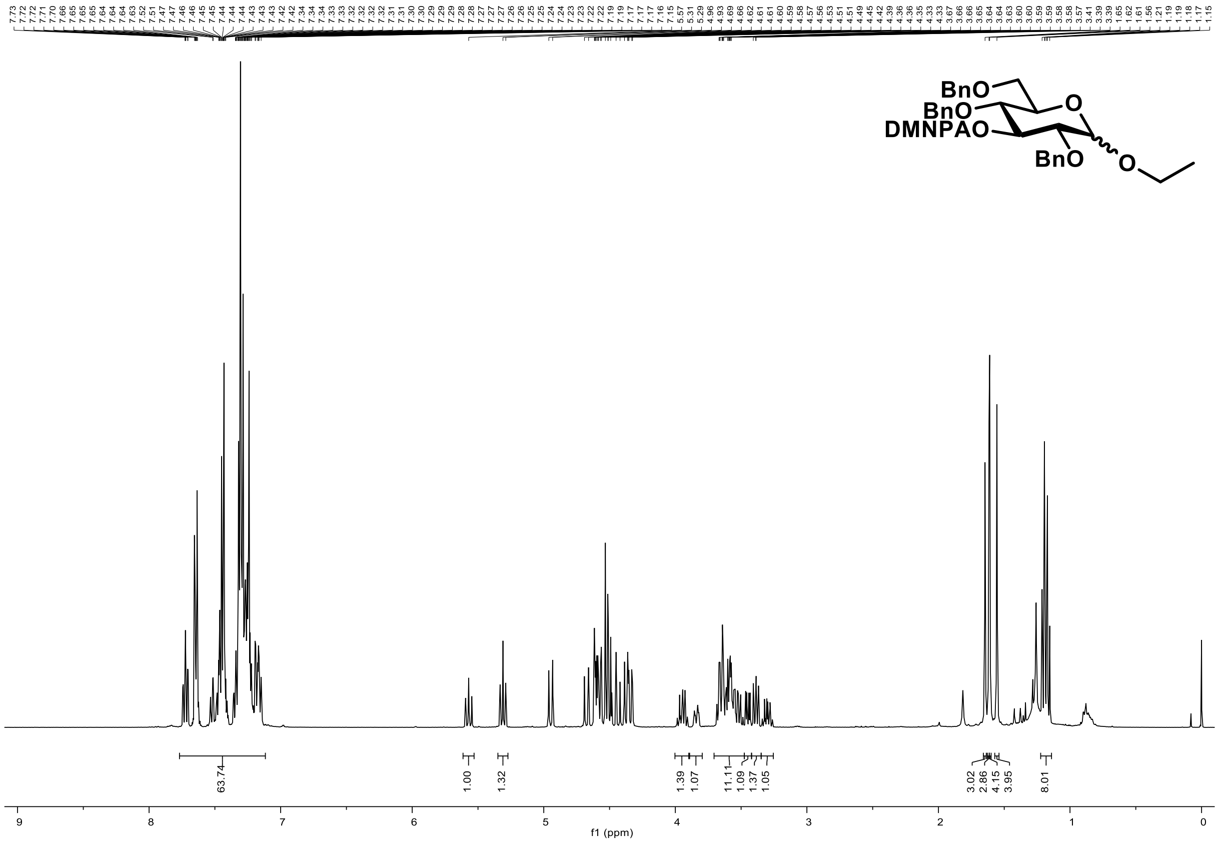

Supplementary Figure S136. ^{1}H NMR, 400 MHz, $CDCl_3$ of compound **S24**

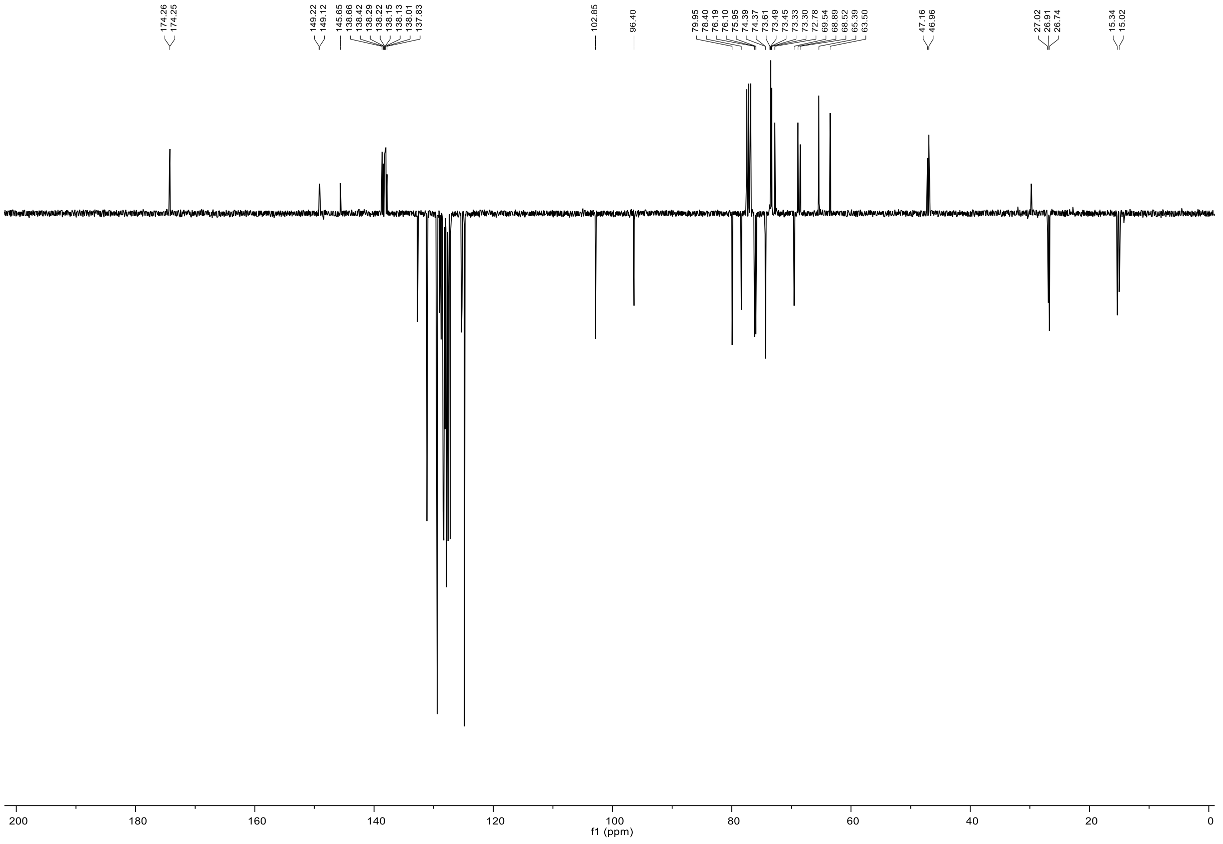

Supplementary Figure S137. $^{13}C\{^{1}H\}$ NMR, 101 MHz, $CDCl_3$ of compound **S24**

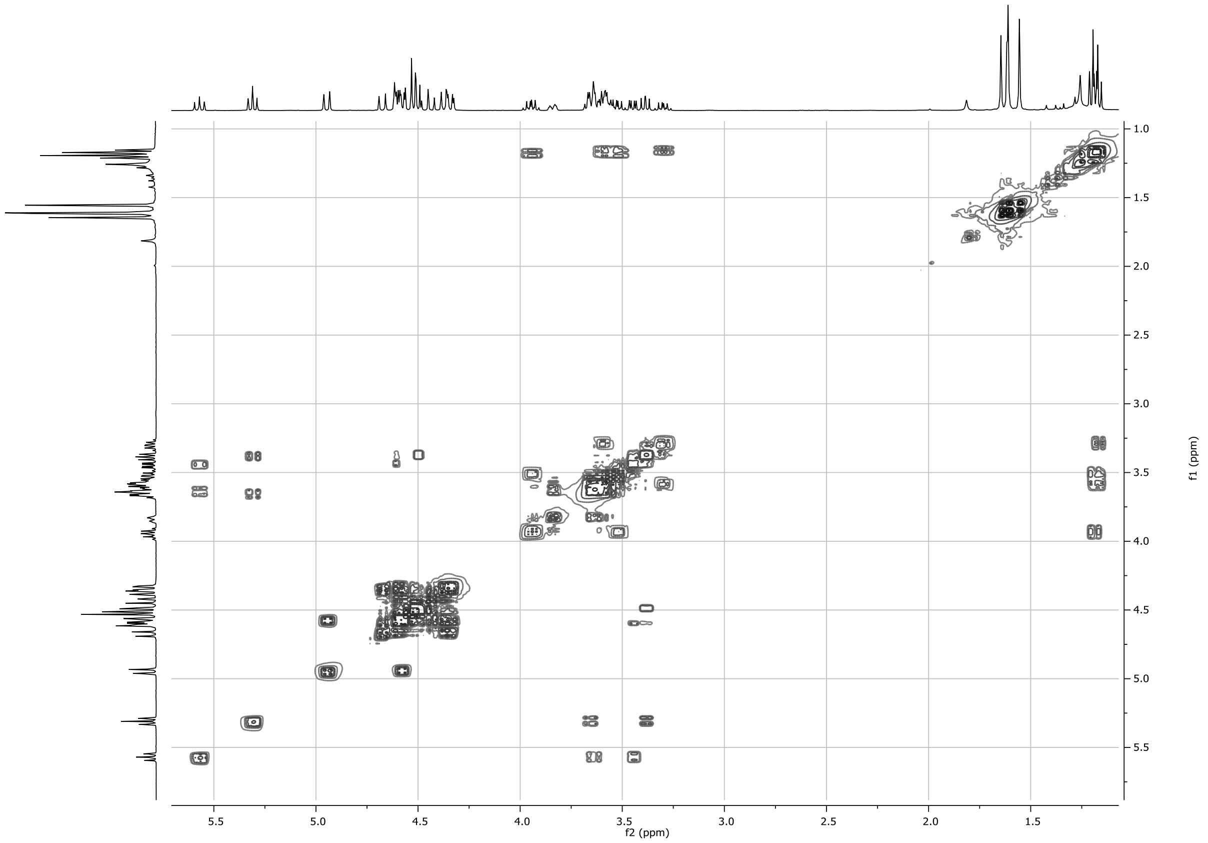

Supplementary Figure S138. HH-COSY NMR, $CDCl_3$ of compound **S24**

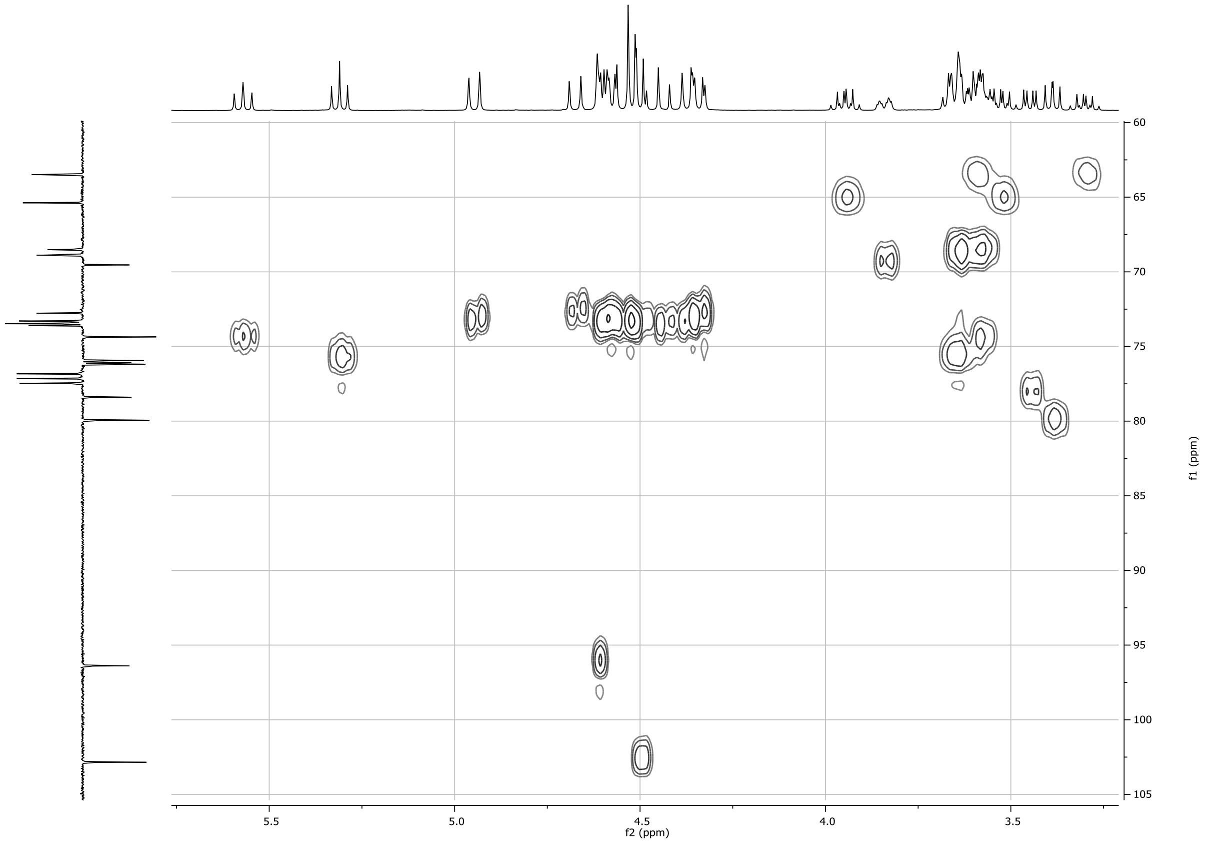

Supplementary Figure S139. HSQC{^{1}H} NMR, $CDCl_3$ of compound **S24**

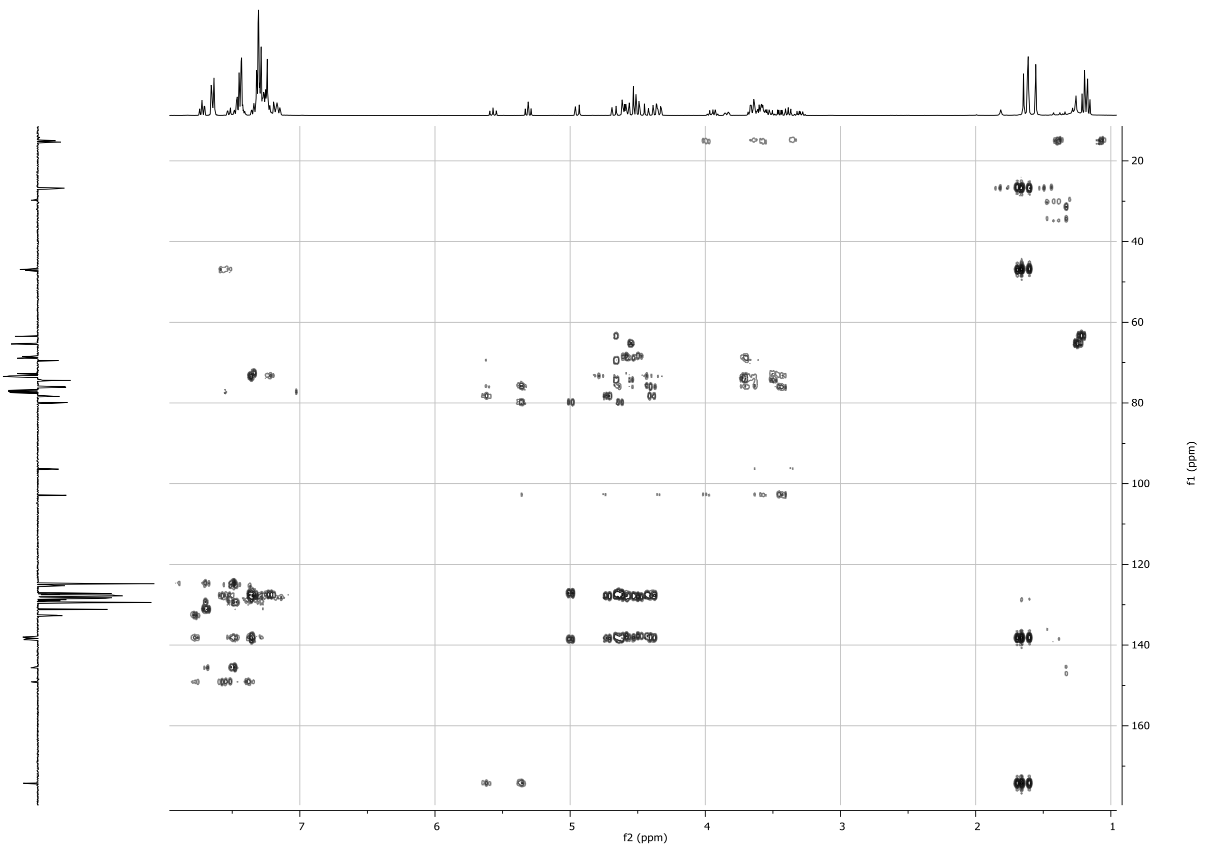

Supplementary Figure S140. HMBC{^{1}H} NMR, $CDCl_3$ of compound **S24**

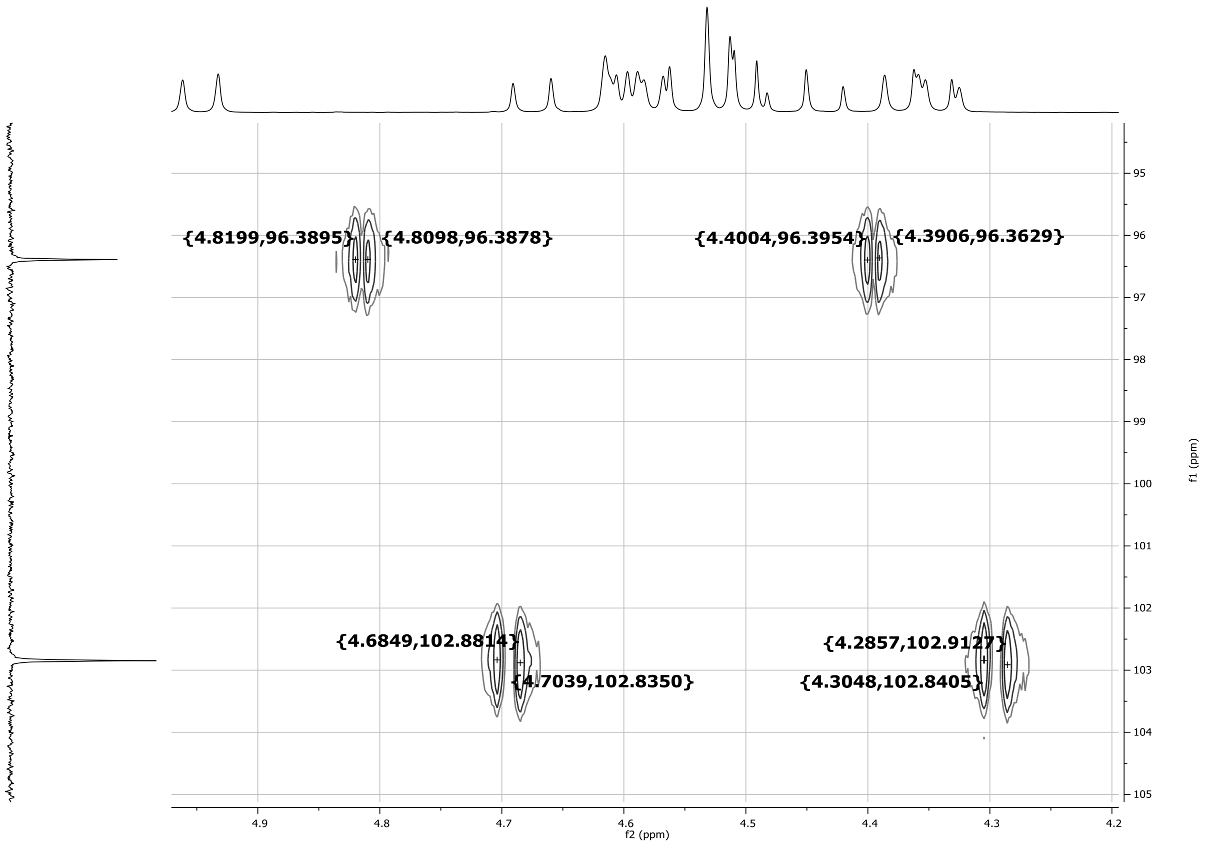

Supplementary Figure S141. HMBC-Gated NMR, $CDCl_3$ of compound **S24**

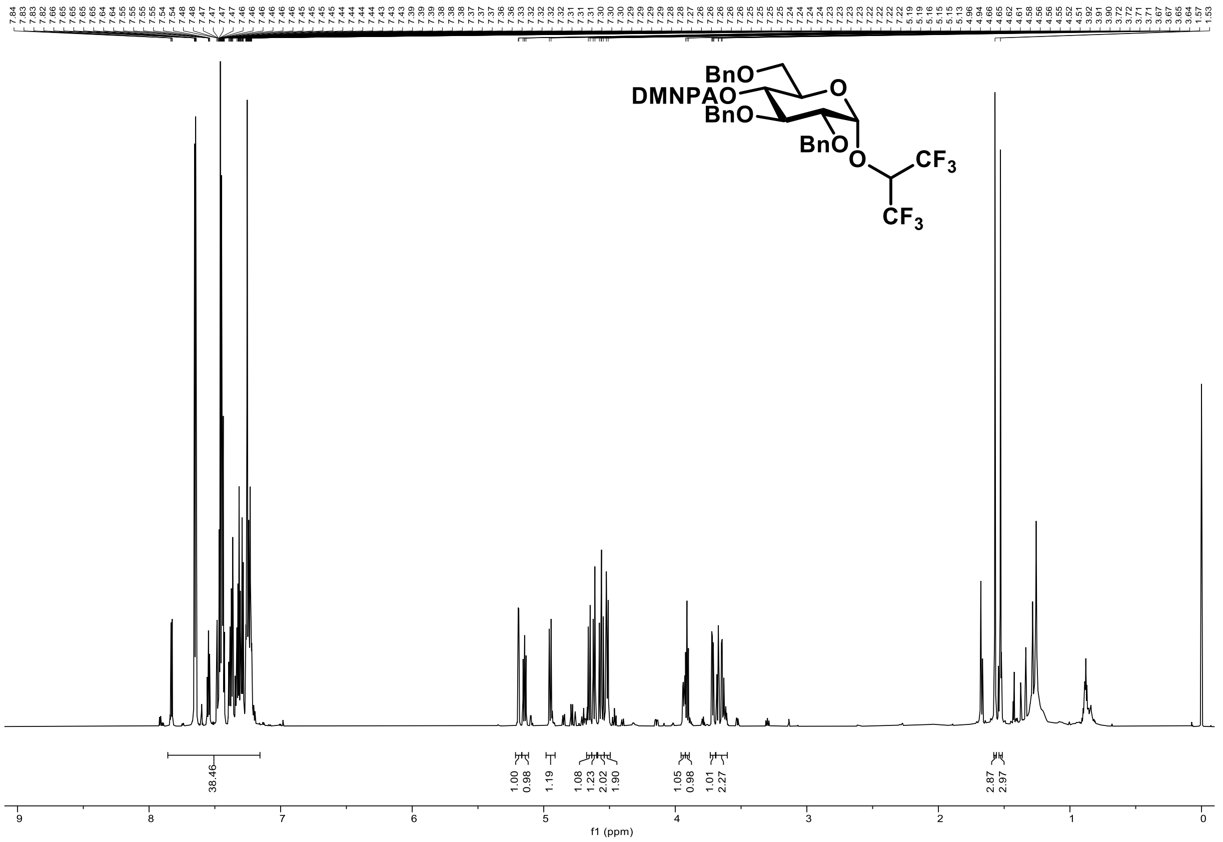

Supplementary Figure S142. ^{1}H NMR, 850 MHz, $CDCl_3$ of compound **S25**

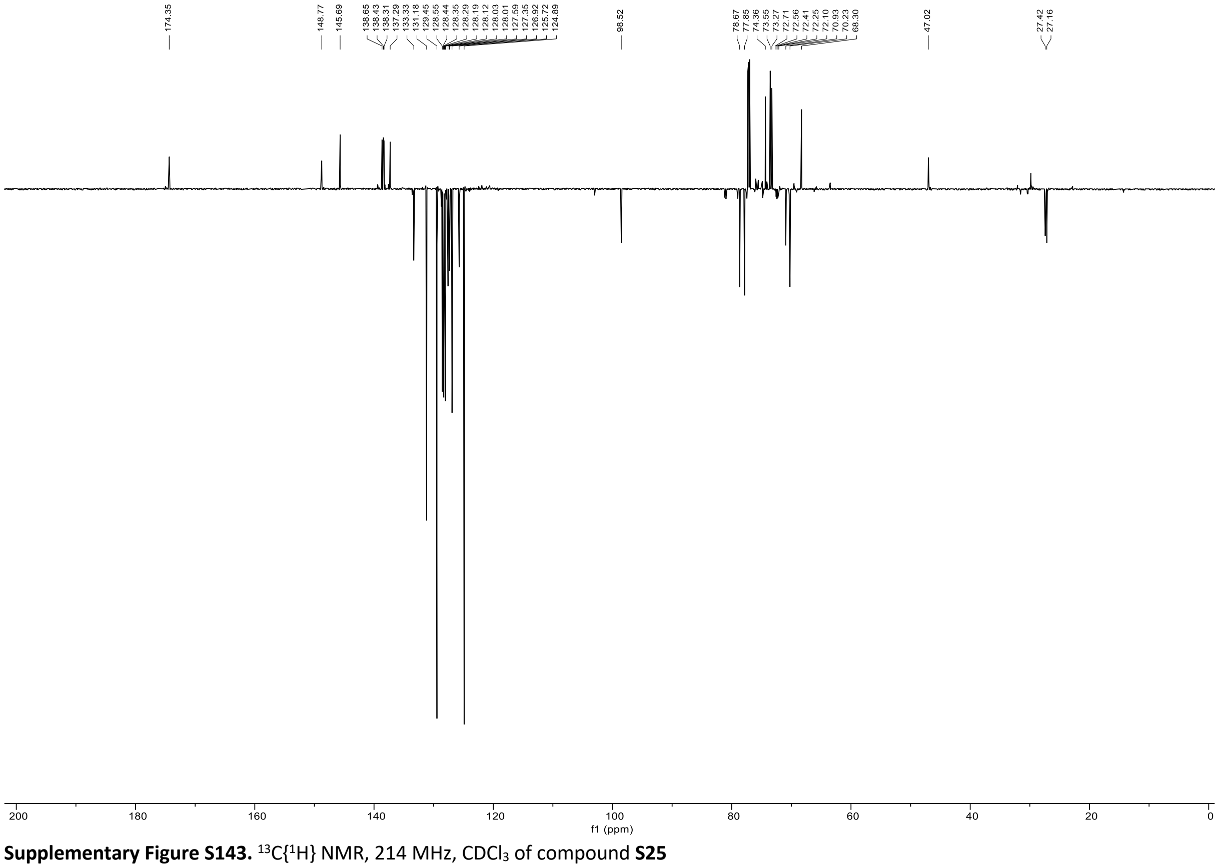

Supplementary Figure S143. $^{13}C\{^{1}H\}$ NMR, 214 MHz, $CDCl_3$ of compound **S25**

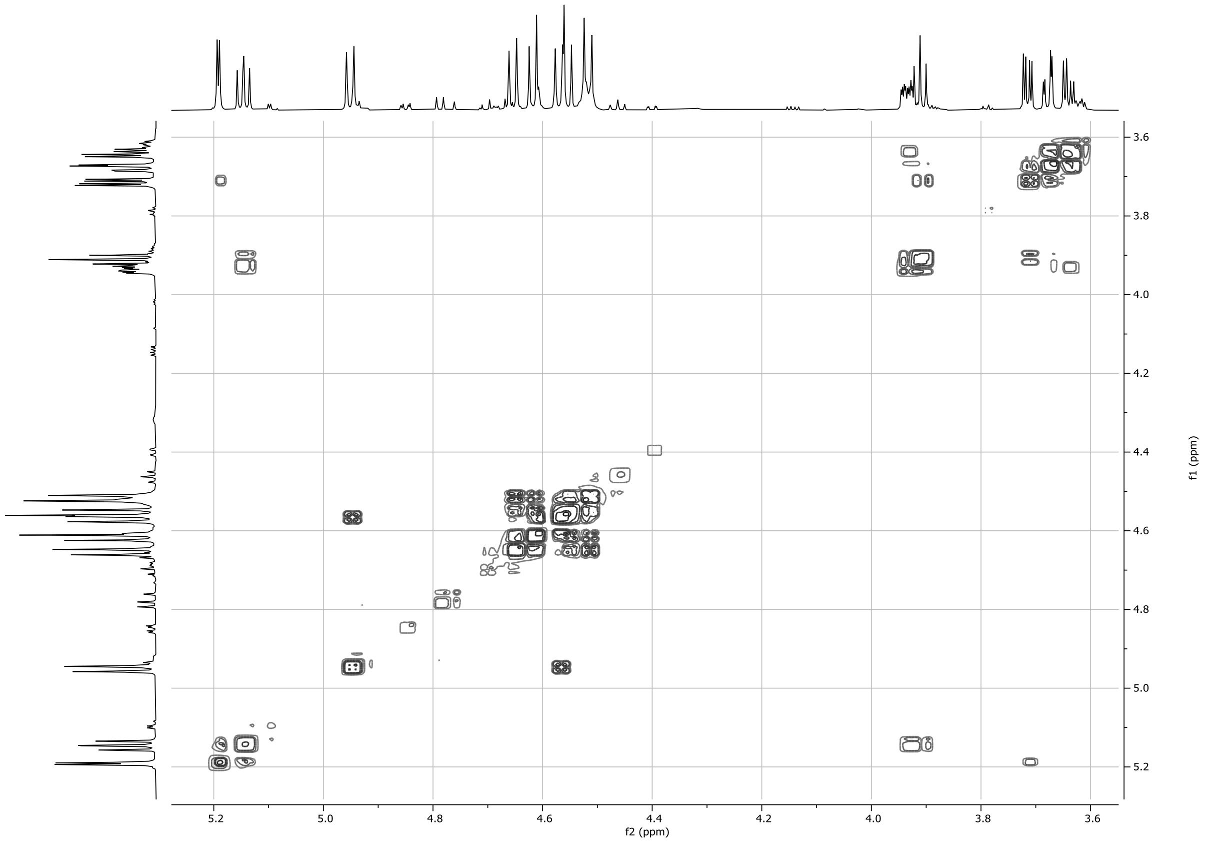

Supplementary Figure S144. HH-COSY NMR, $CDCl_3$ of compound **S25**

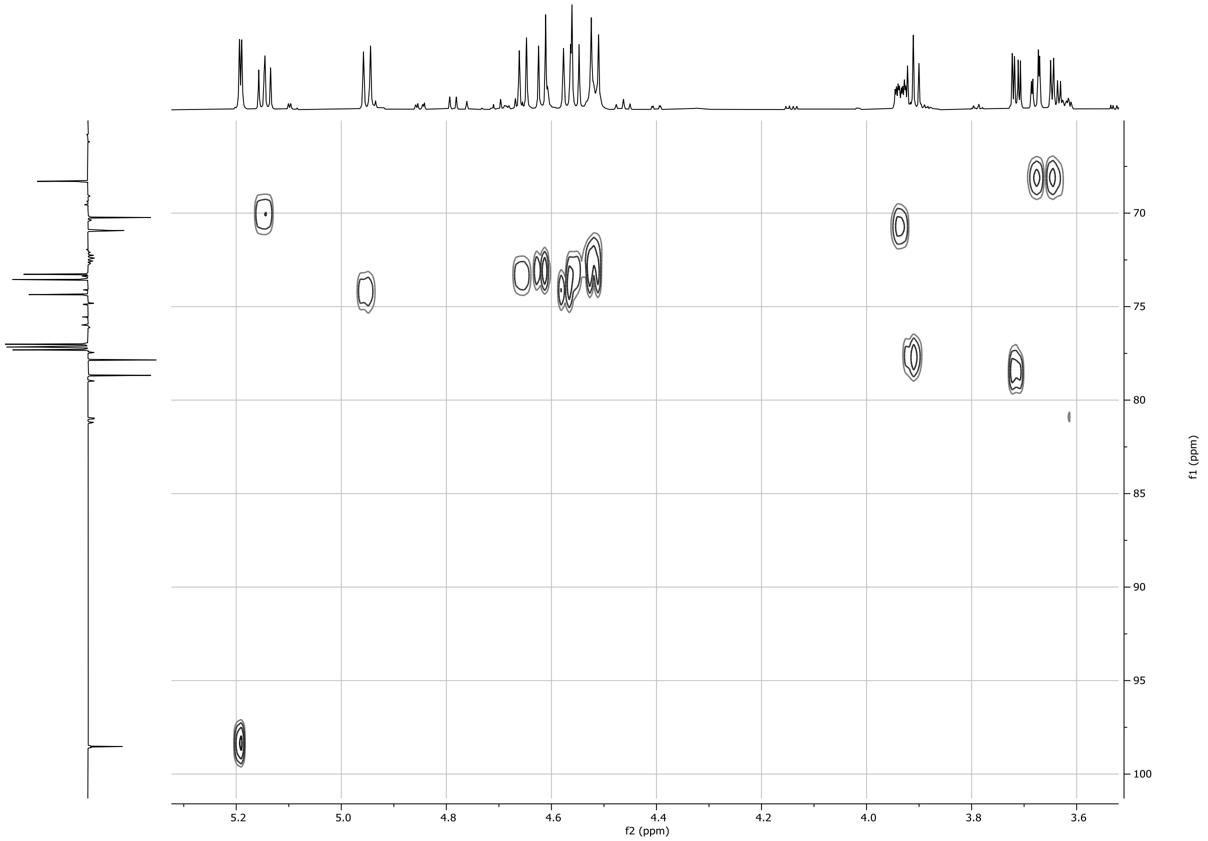

Supplementary Figure S145. HSQC{^{1}H} NMR, $CDCl_3$ of compound **S25**

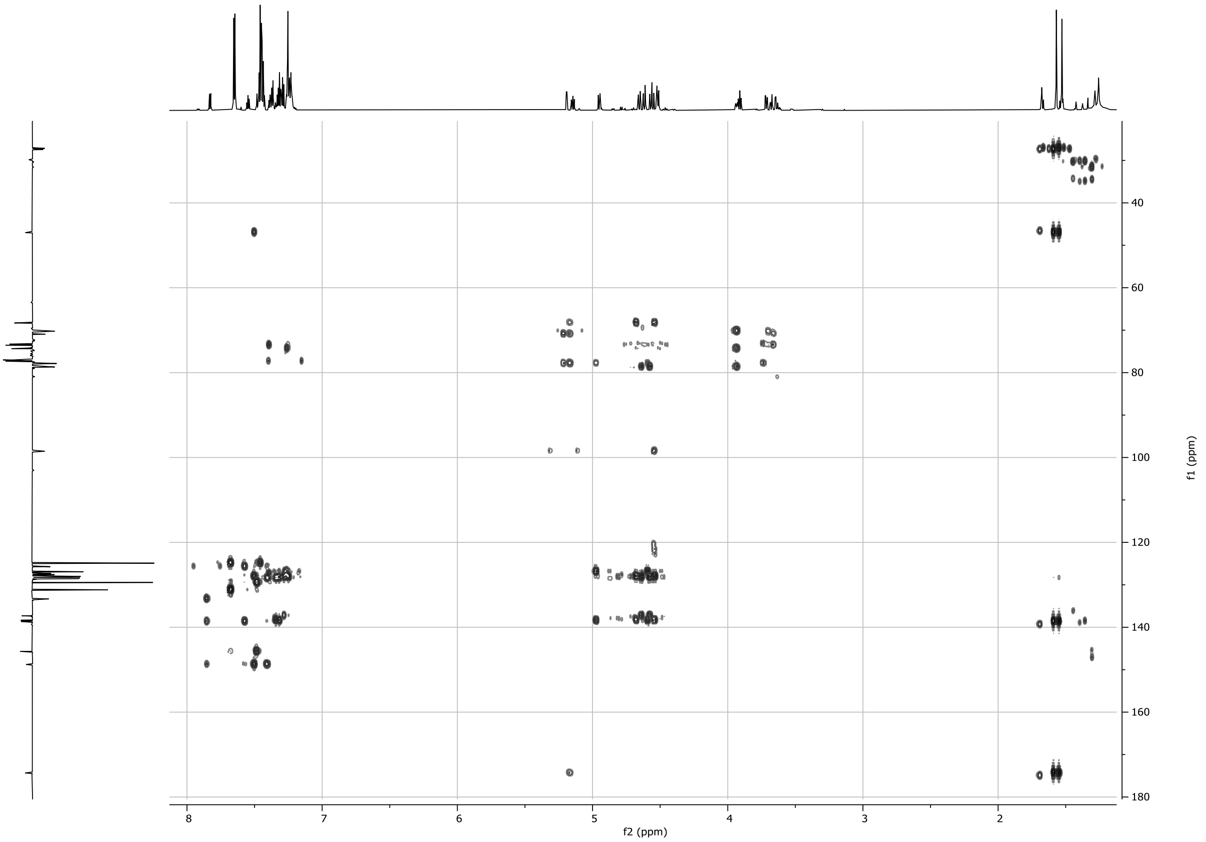

Supplementary Figure S146. HMBC{^{1}H} NMR, $CDCl_3$ of compound **S25**

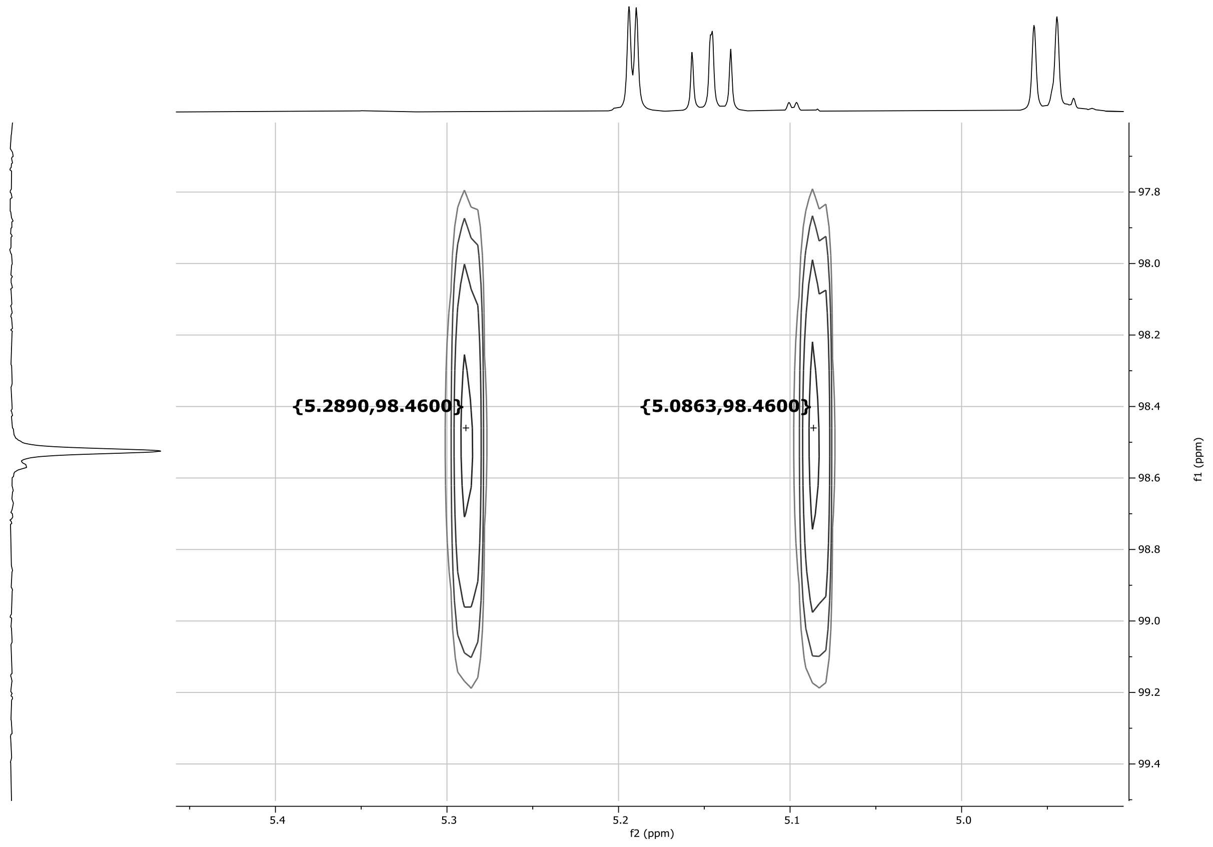

Supplementary Figure S147. HMBC-Gated NMR, $CDCl_3$ of compound **S25**

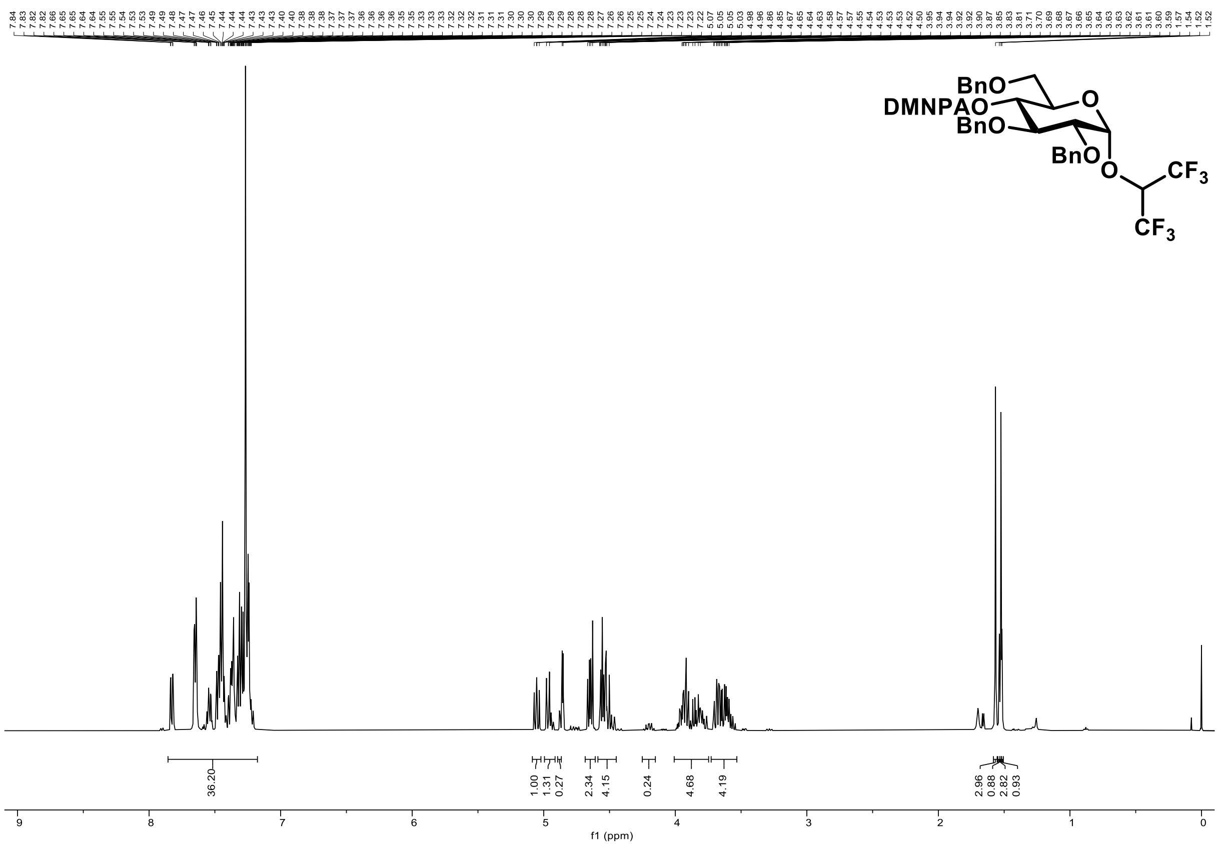

Supplementary Figure S148. ^{1}H NMR, 500 MHz, $CDCl_3$ of compound **S26**

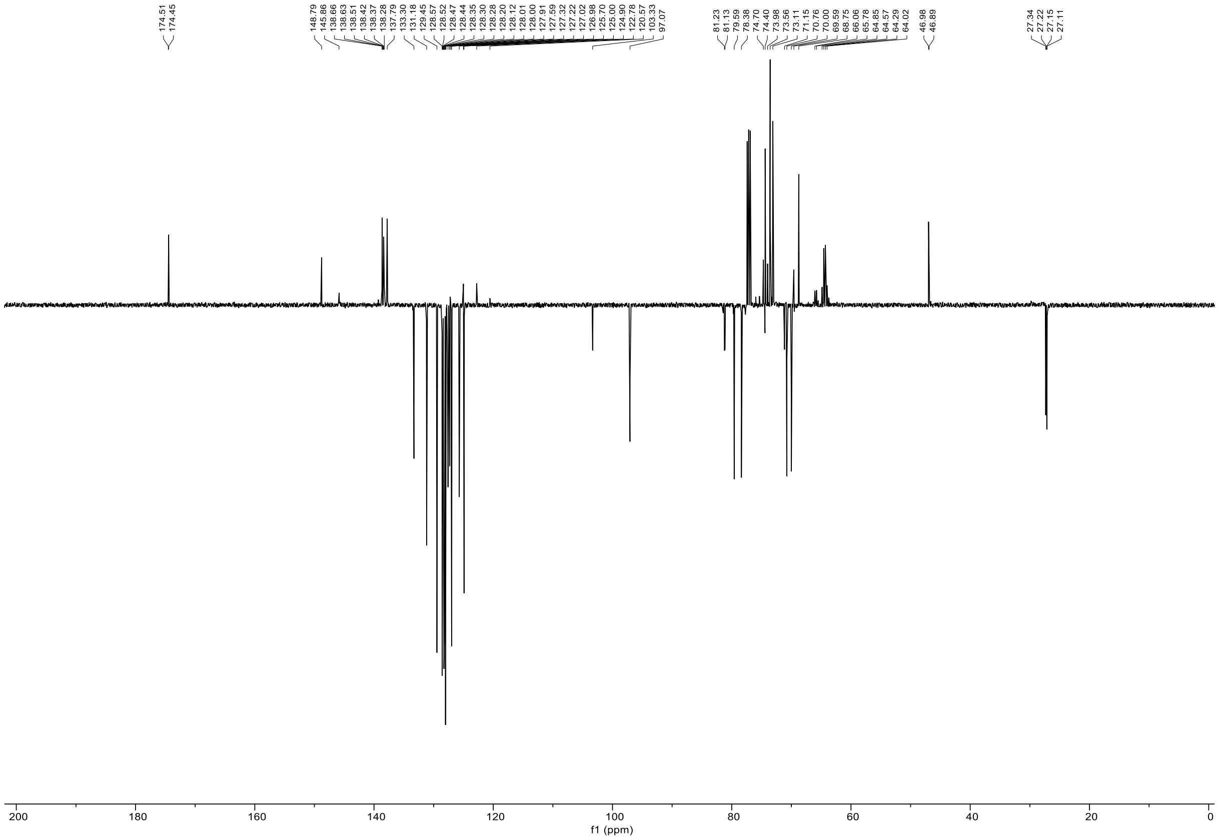

Supplementary Figure S149. $^{13}C\{^1H\}$ NMR, 126 MHz, $CDCl_3$ of compound **S26**

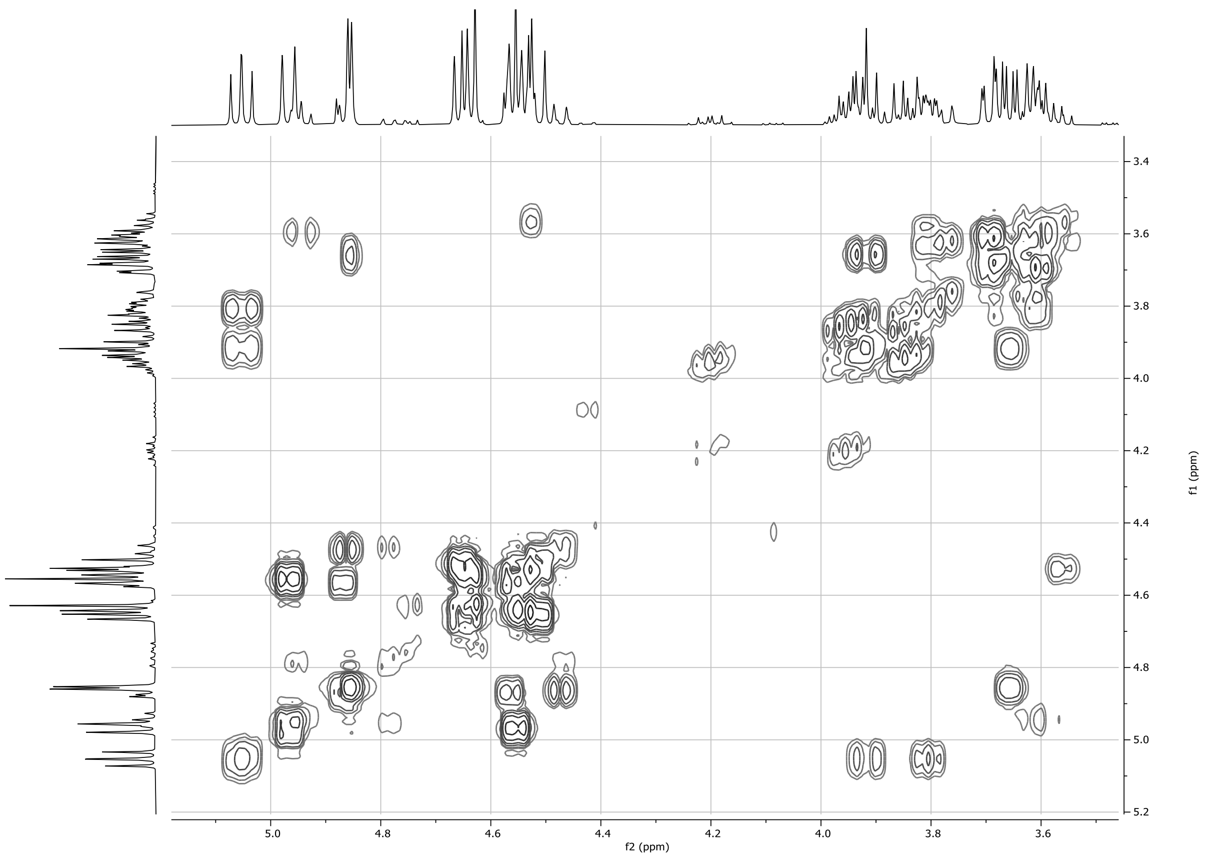

Supplementary Figure S150. HH-COSY NMR, $CDCl_3$ of compound **S26**

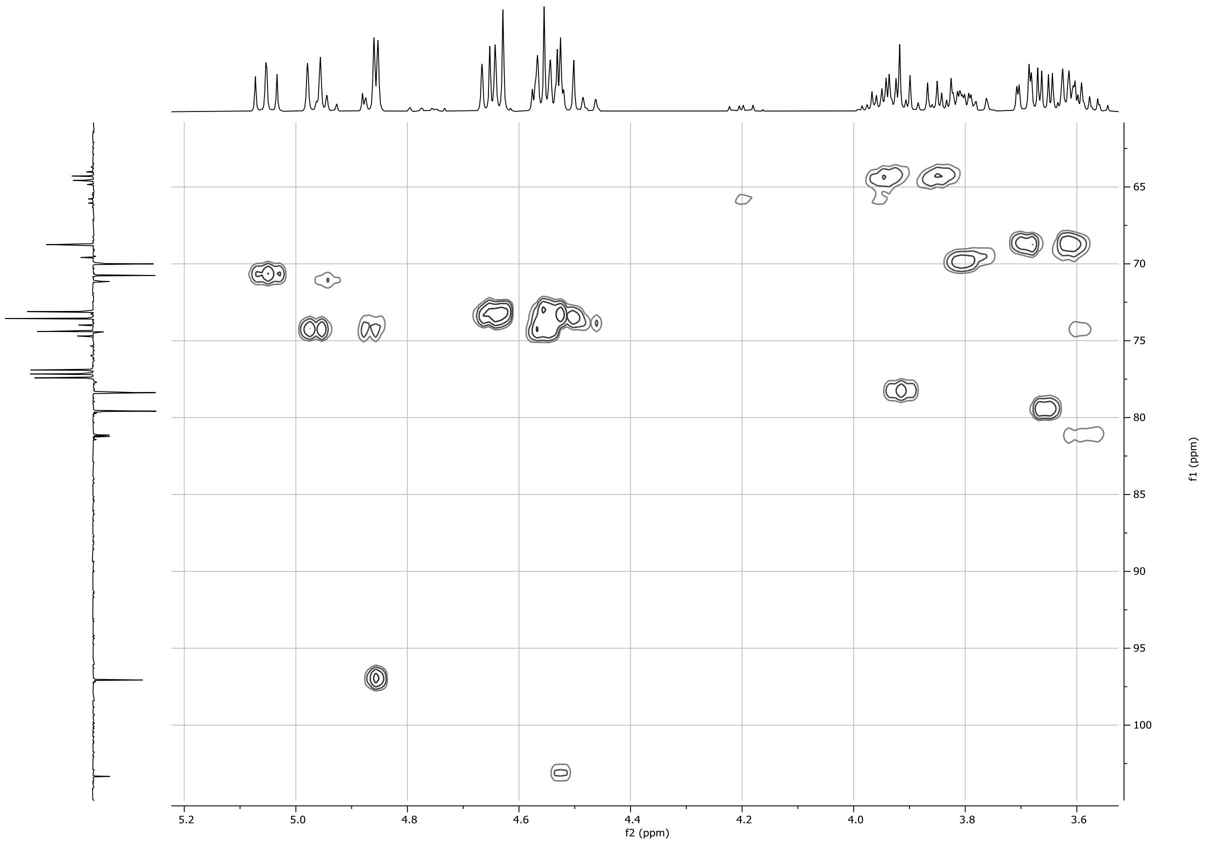

Supplementary Figure S151. HSQC{^{1}H} NMR, $CDCl_3$ of compound **S26**

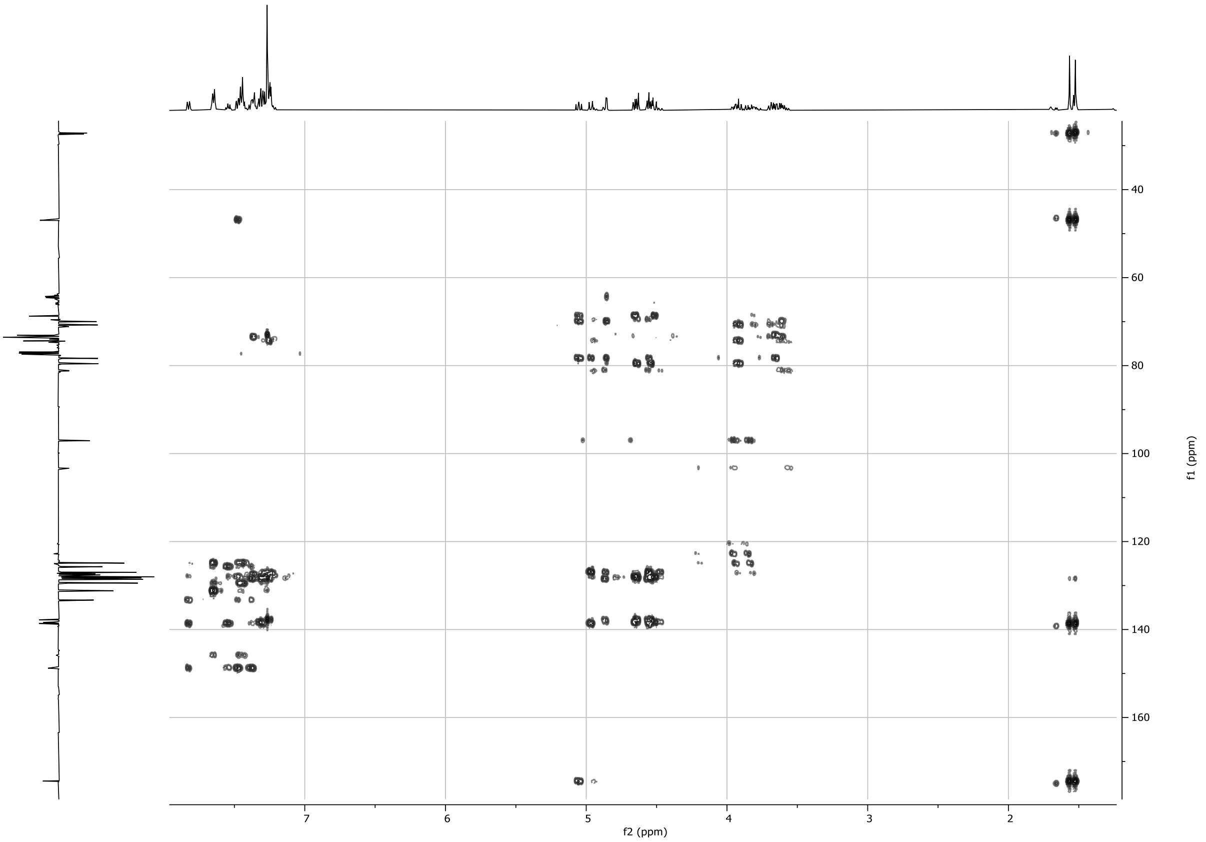

Supplementary Figure S152. HMBC{^{1}H} NMR, $CDCl_3$ of compound **S26**

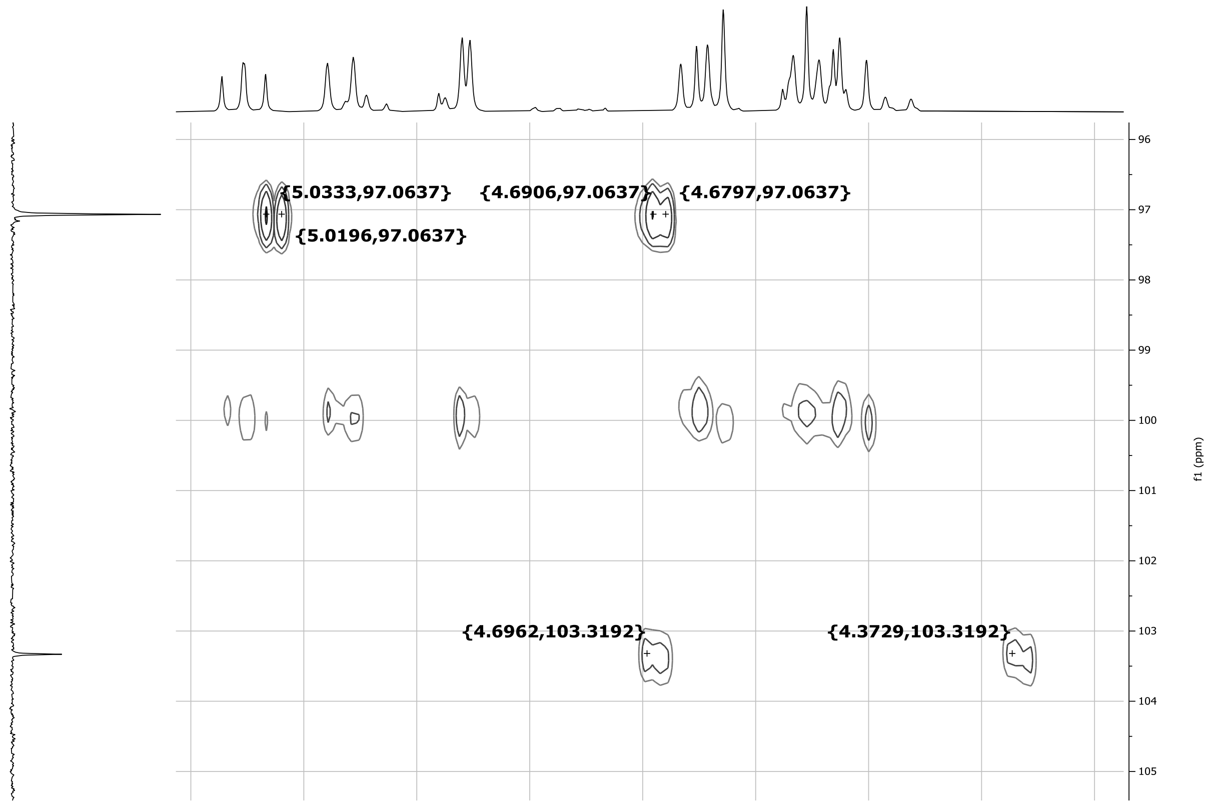

Supplementary Figure S153. HMBC-Gated NMR, $CDCl_3$ of compound **S26**

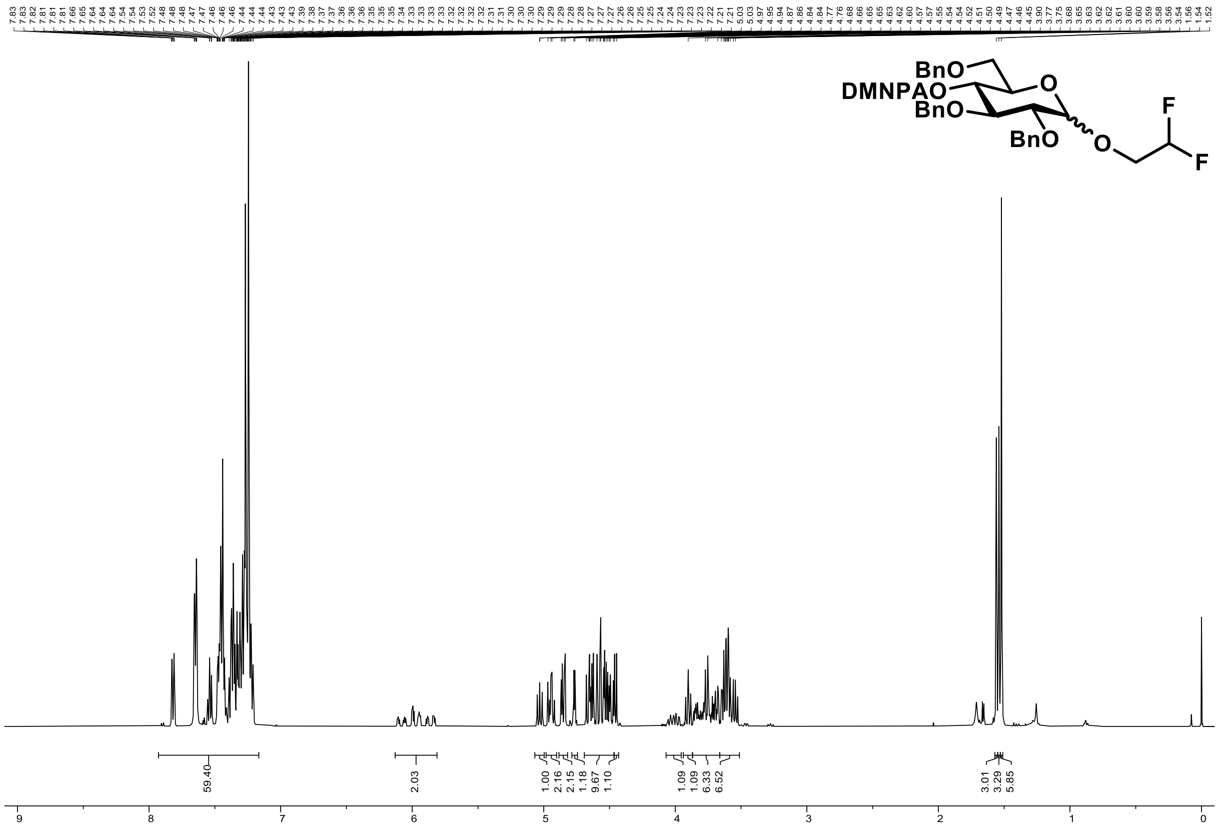

Supplementary Figure S154. ^{1}H NMR, 500 MHz, $CDCl_3$ of compound **S27**

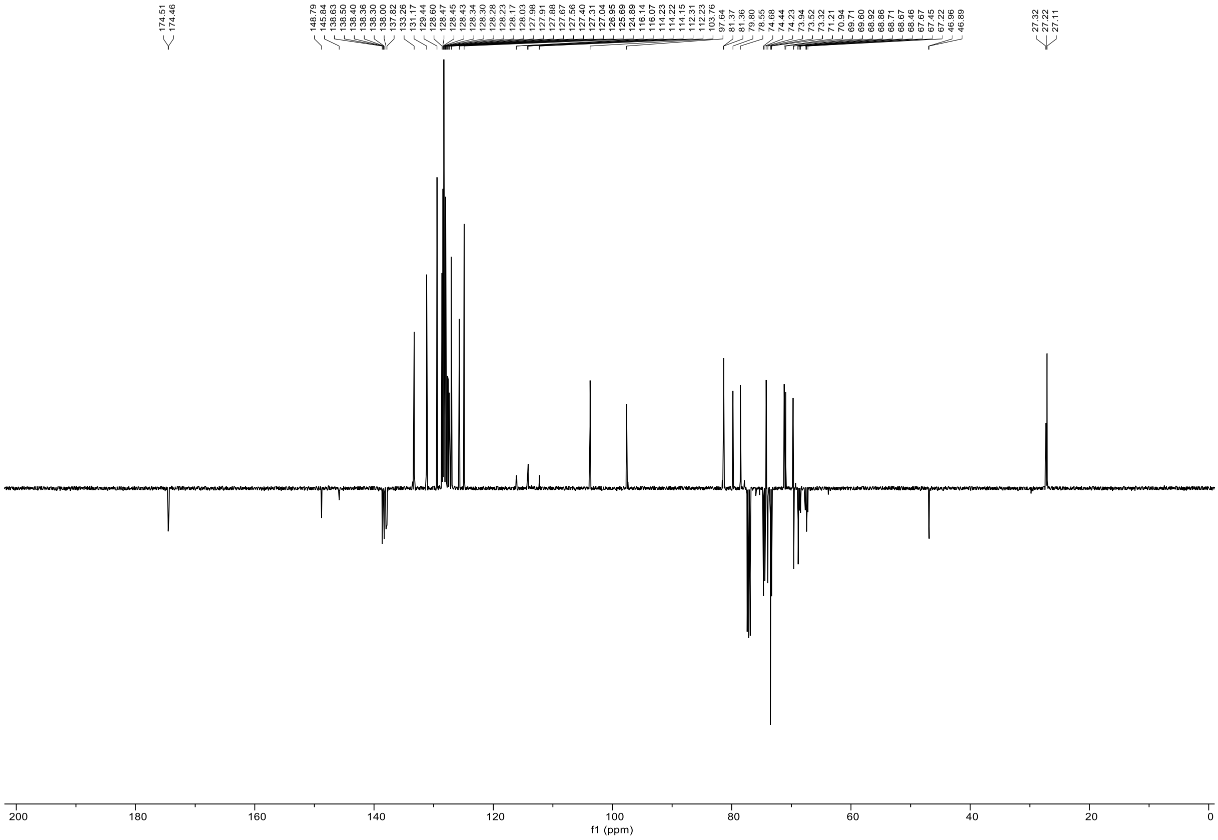

Supplementary Figure S155. $^{13}C\{^{1}H\}$ NMR, 126 MHz, $CDCl_3$ of compound **S27**

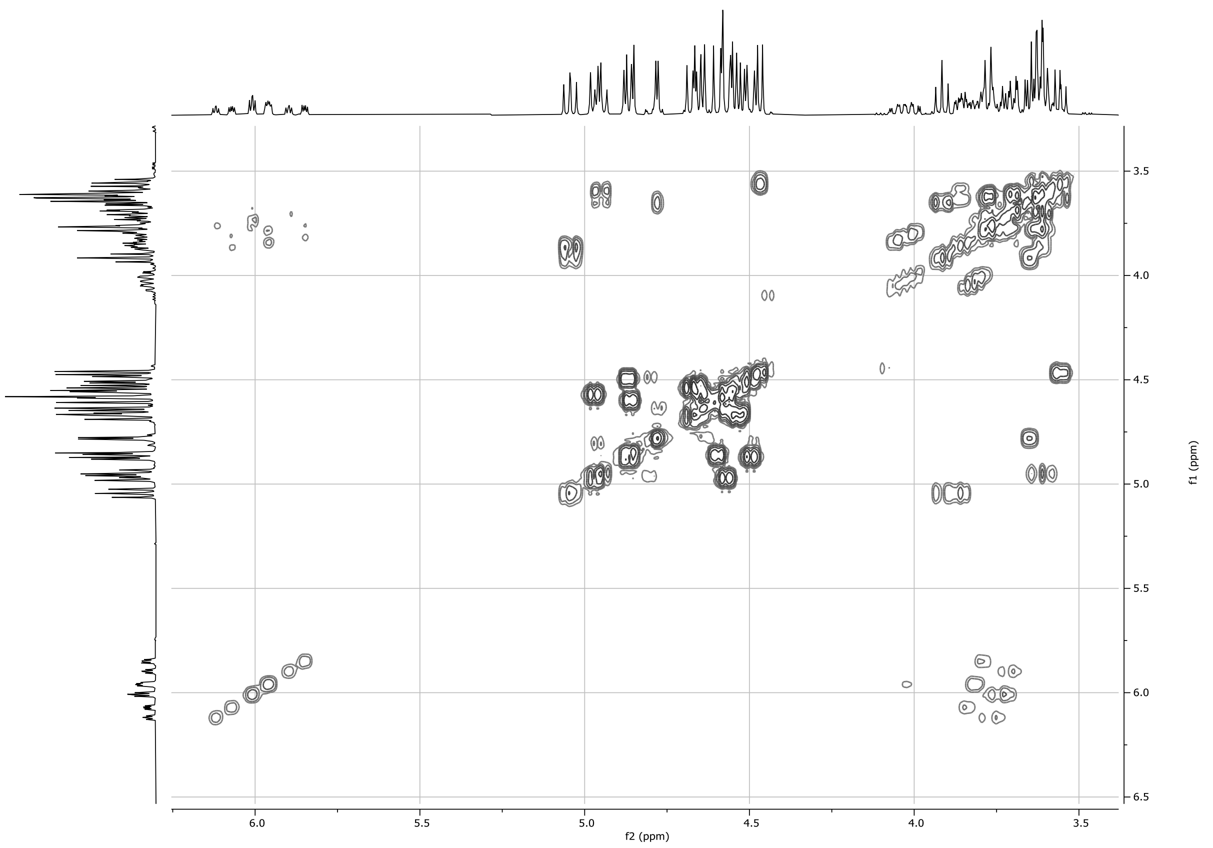

Supplementary Figure S156. HH-COSY NMR, $CDCl_3$ of compound **S27**

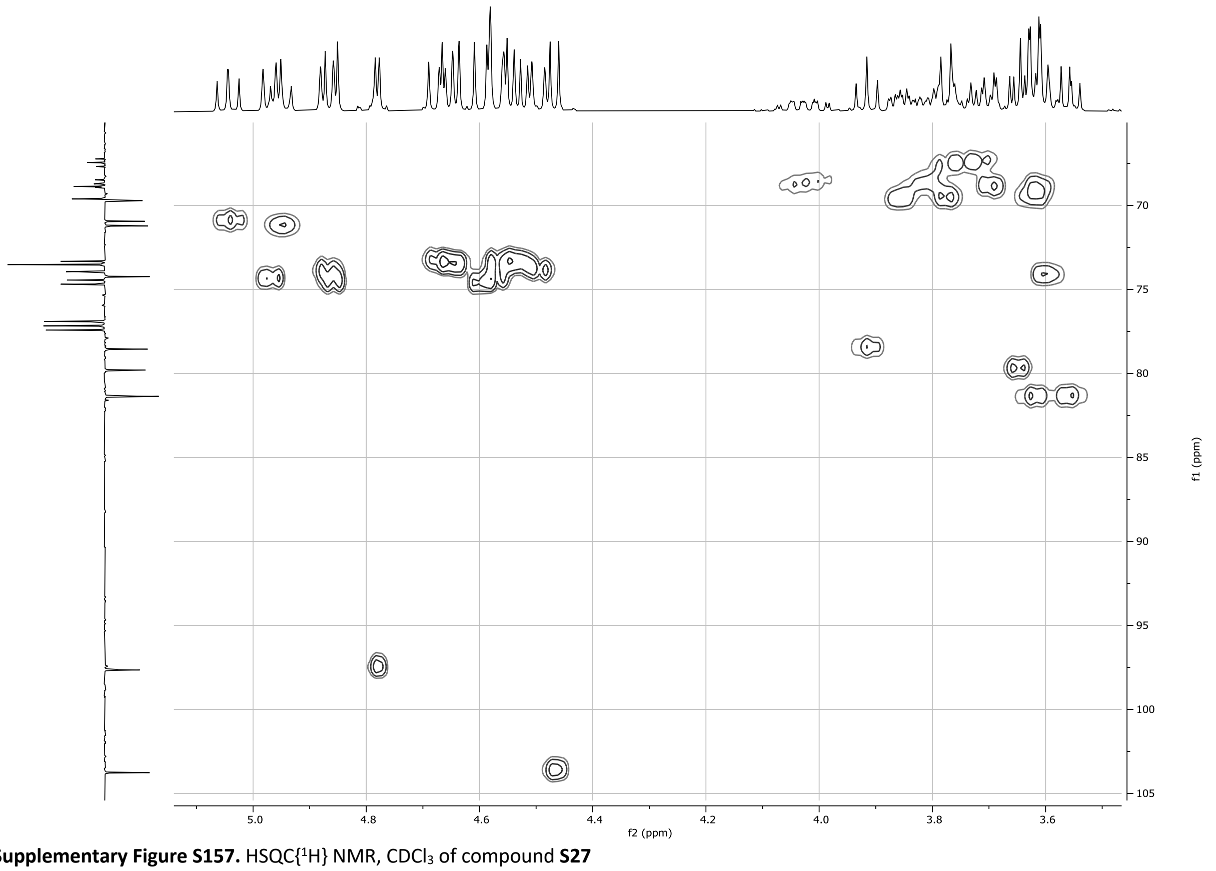

Supplementary Figure S157. HSQC{^{1}H} NMR, $CDCl_3$ of compound **S27**

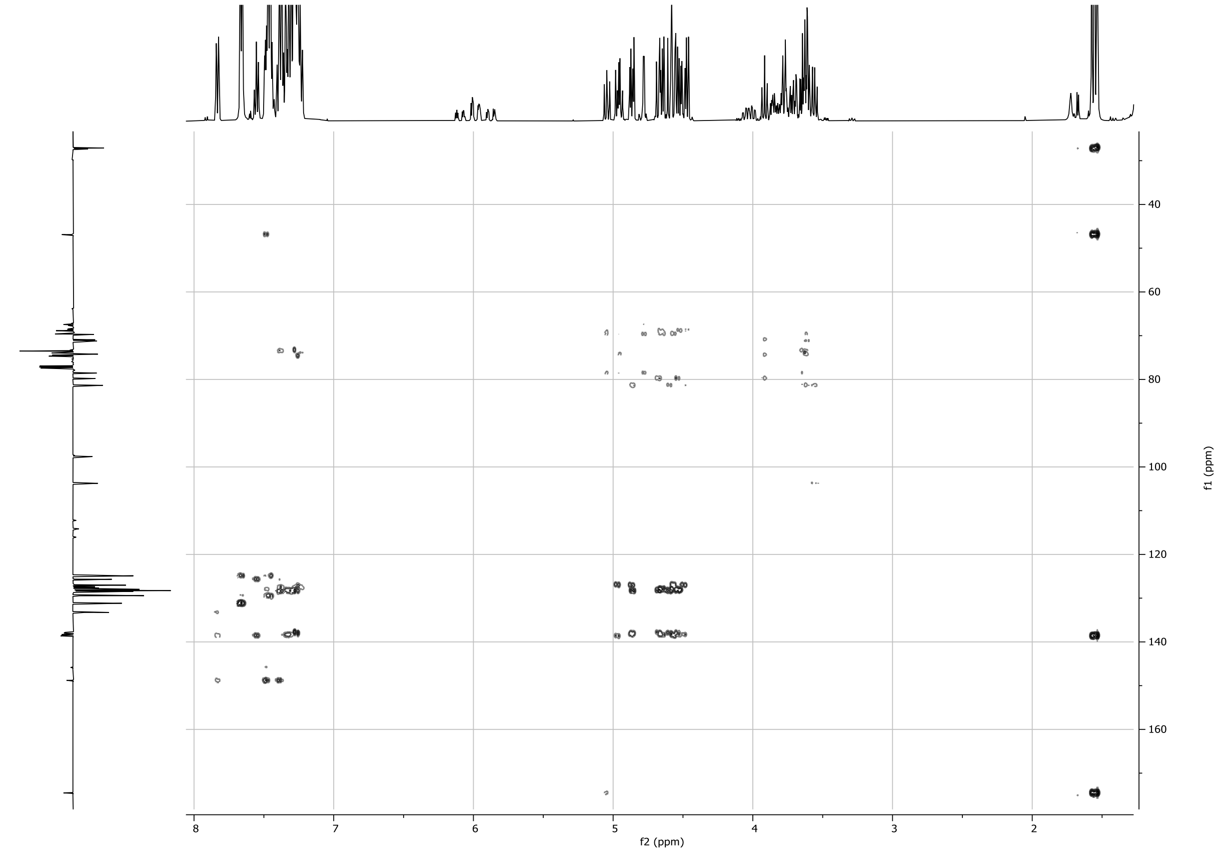

Supplementary Figure S158. HMBC{^{1}H} NMR, $CDCl_3$ of compound **S27**

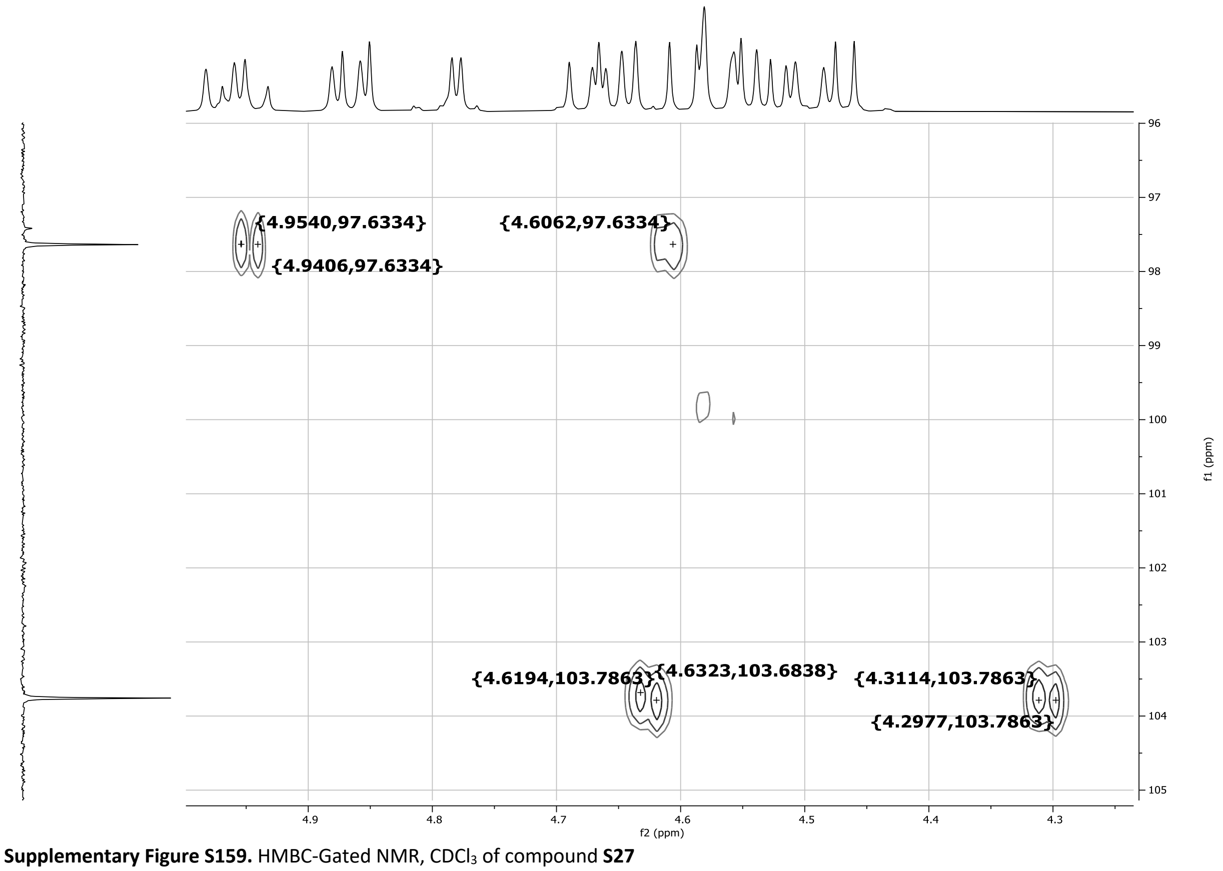

Supplementary Figure S159. HMBC-Gated NMR, $CDCl_3$ of compound **S27**

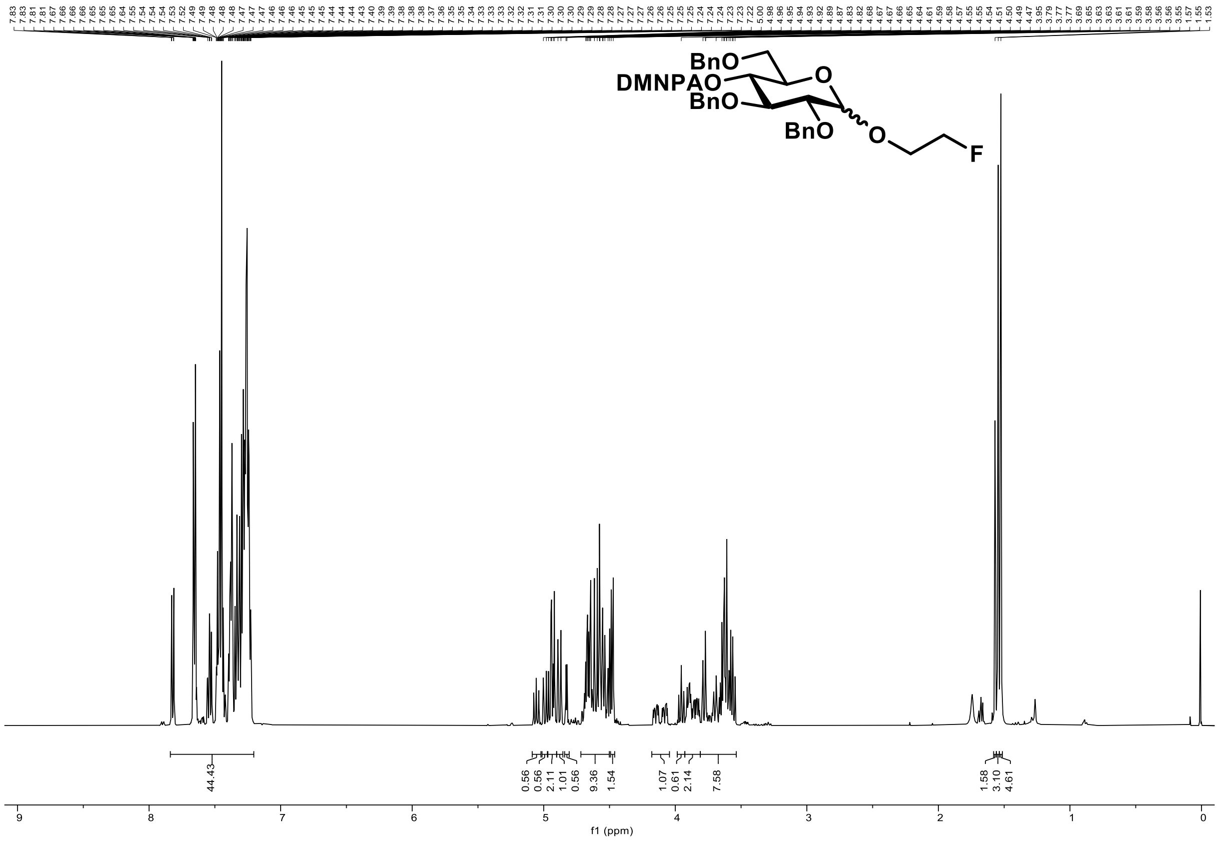

Supplementary Figure S160. ^{1}H NMR, 500 MHz, $CDCl_3$ of compound **S28**

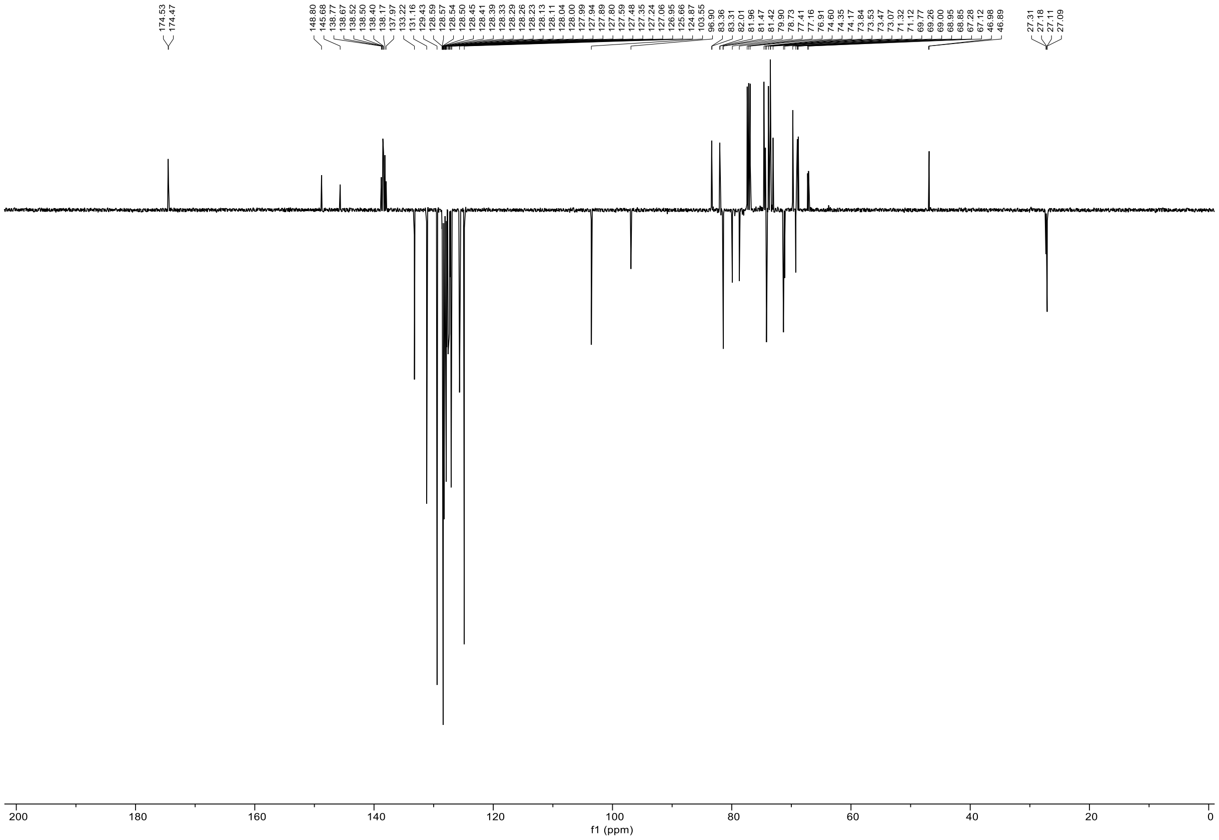

Supplementary Figure S161. ^{13}C{^{1}H} NMR, 126 MHz, $CDCl_3$ of compound **S28**

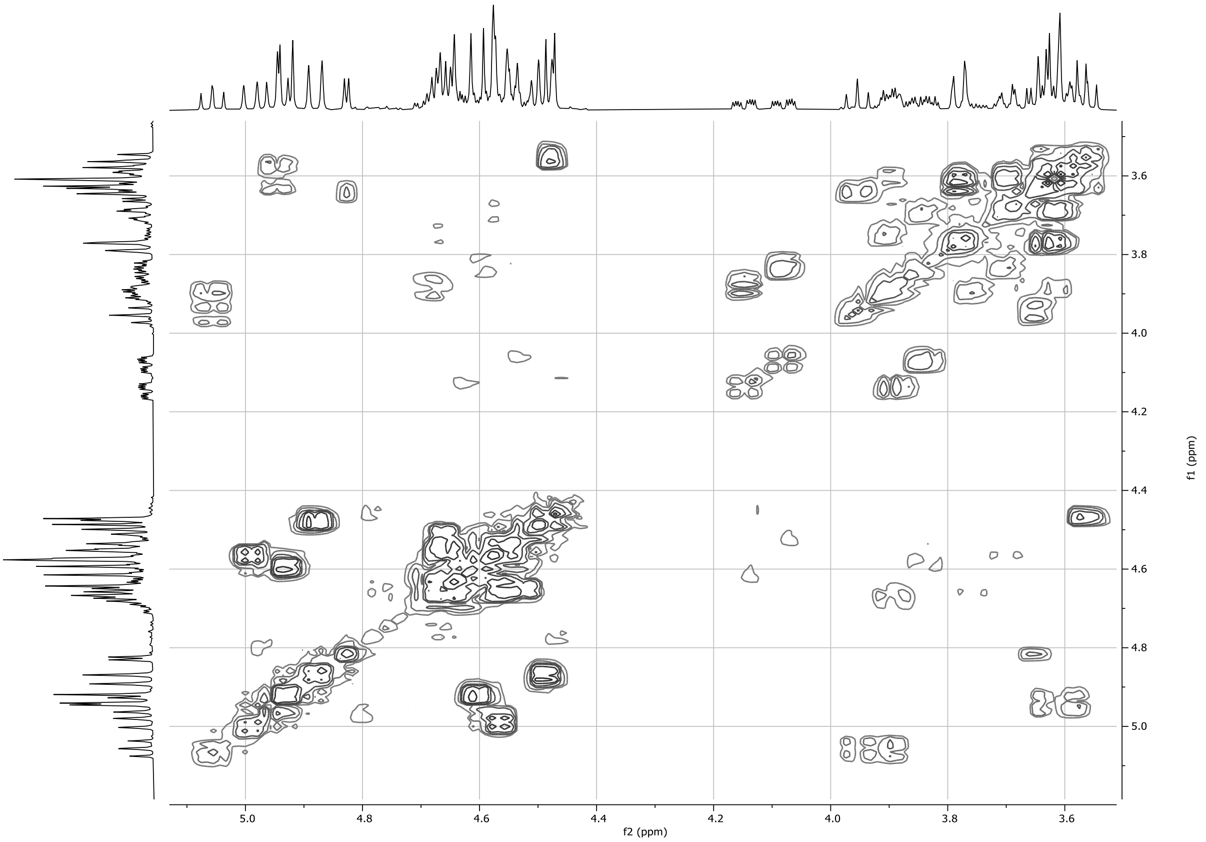

Supplementary Figure S162. HH-COSY NMR, $CDCl_3$ of compound **S28**

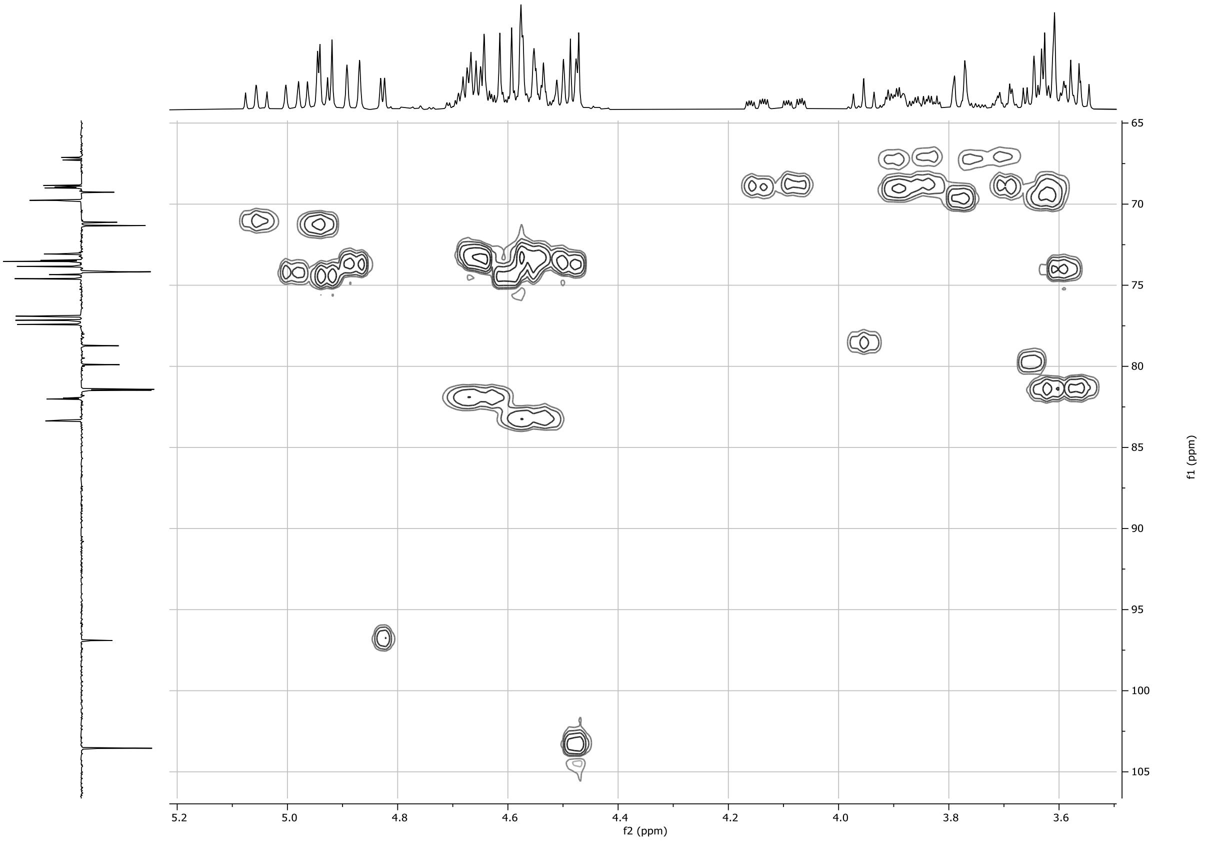

Supplementary Figure S163. HSQC{^{1}H} NMR, $CDCl_3$ of compound **S28**

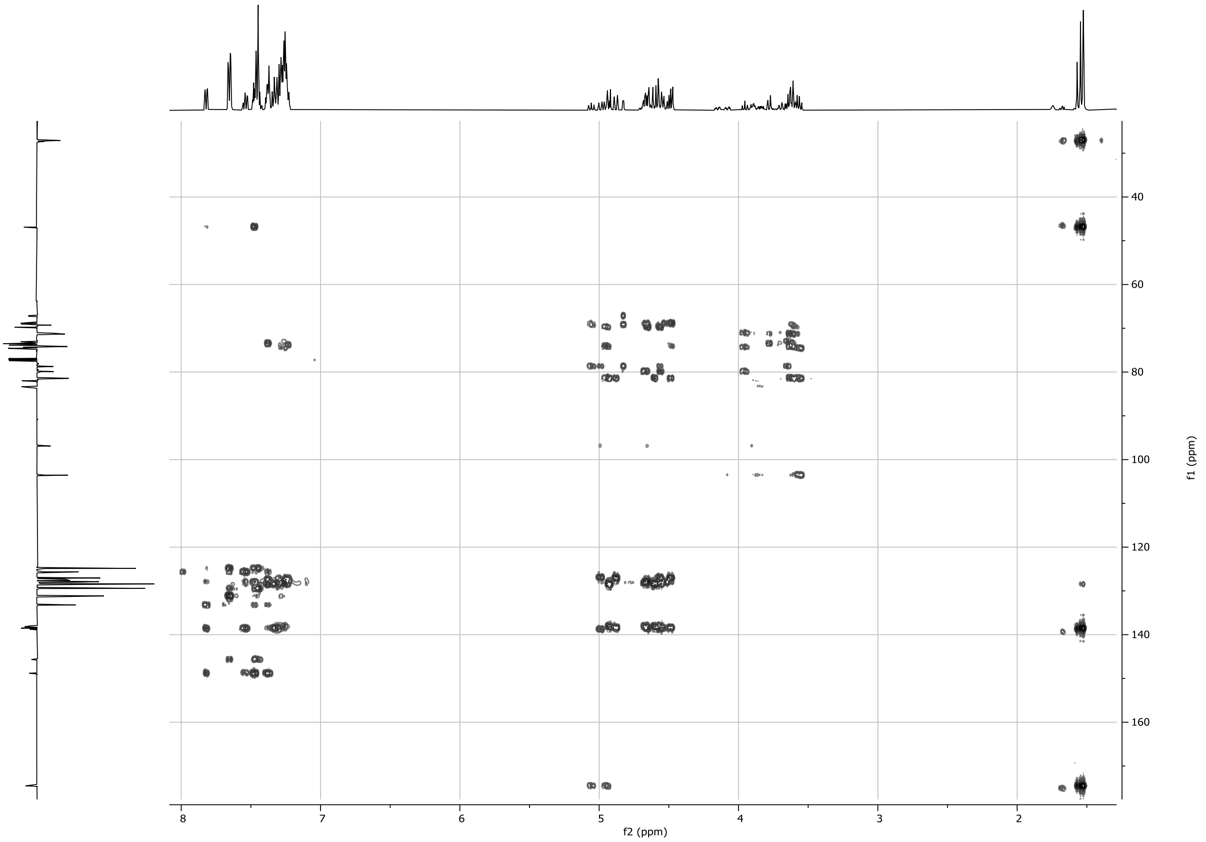

Supplementary Figure S164. HMBC{^{1}H} NMR, $CDCl_3$ of compound **S28**

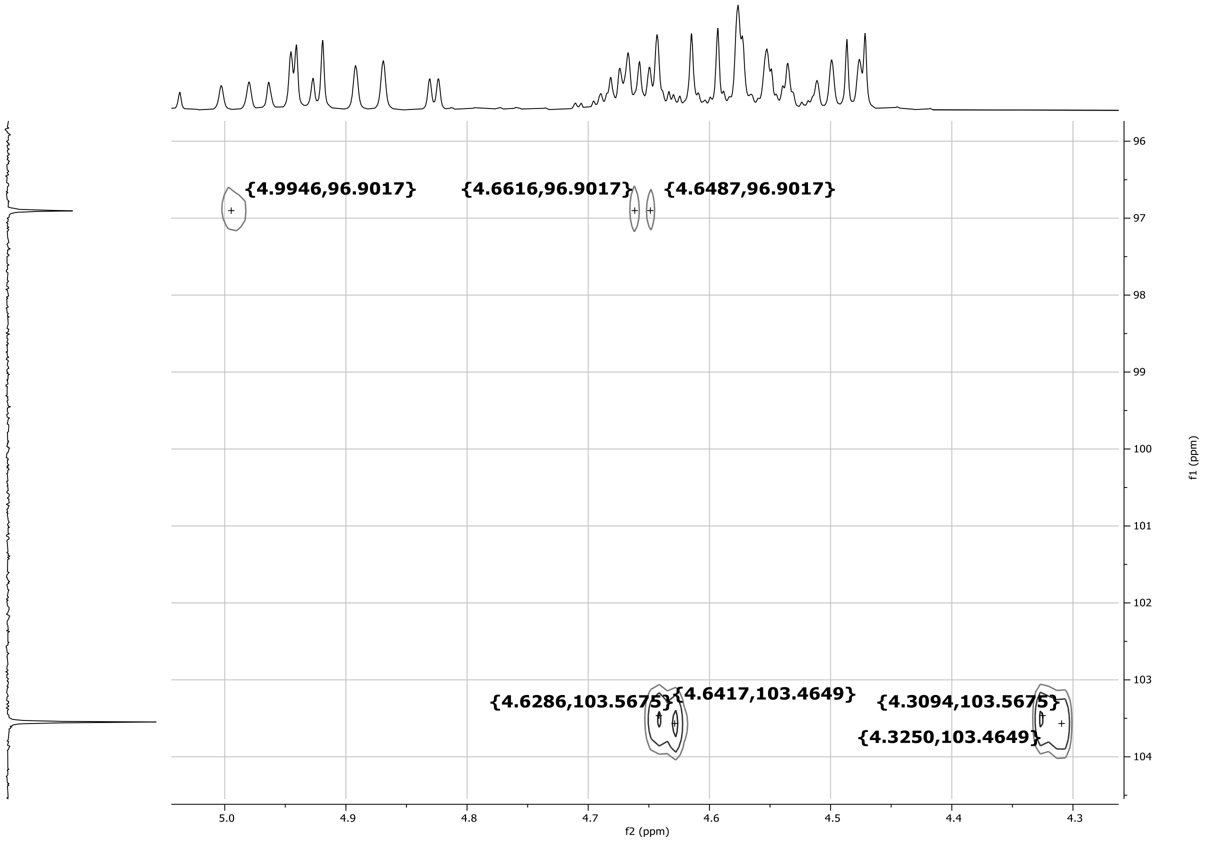

Supplementary Figure S165. HMBC-Gated NMR, $CDCl_3$ of compound **S28**

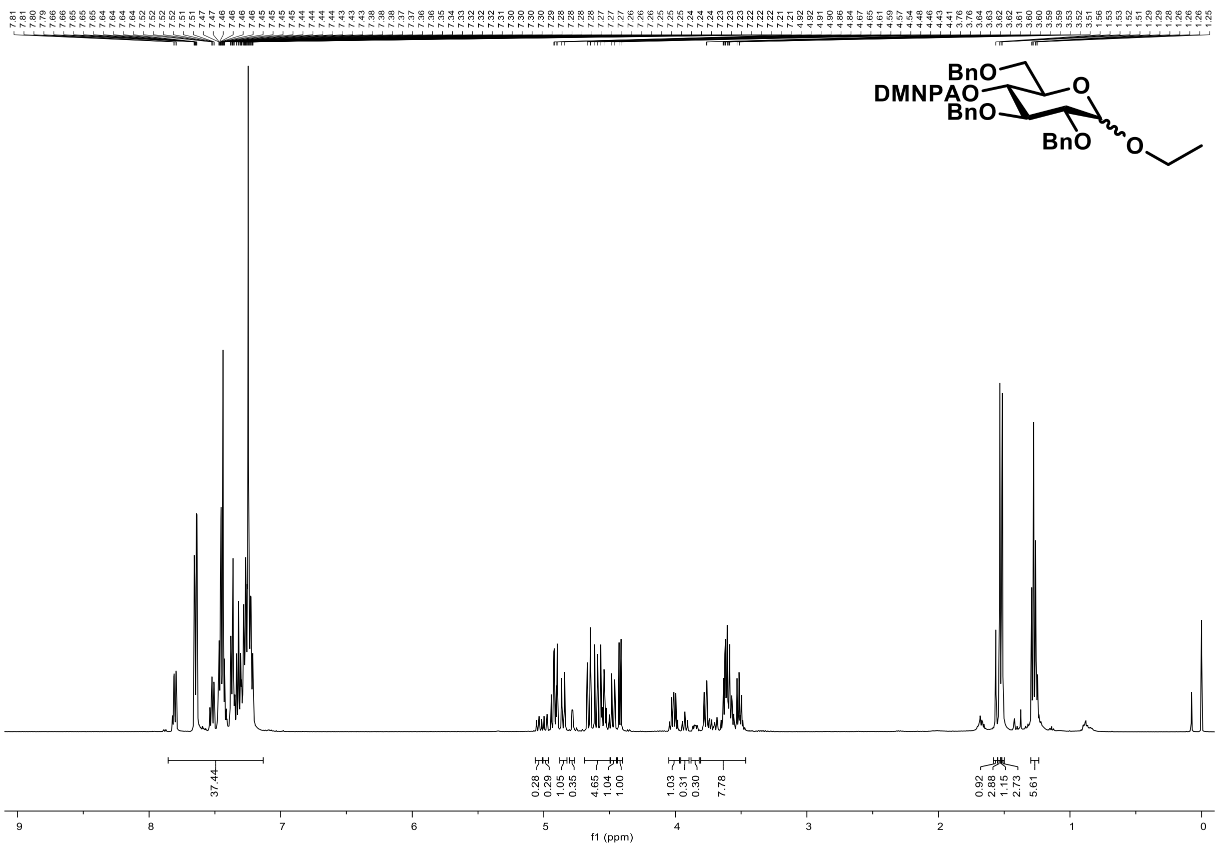

Supplementary Figure S166. ^{1}H NMR, 500 MHz, $CDCl_3$ of compound **S29**

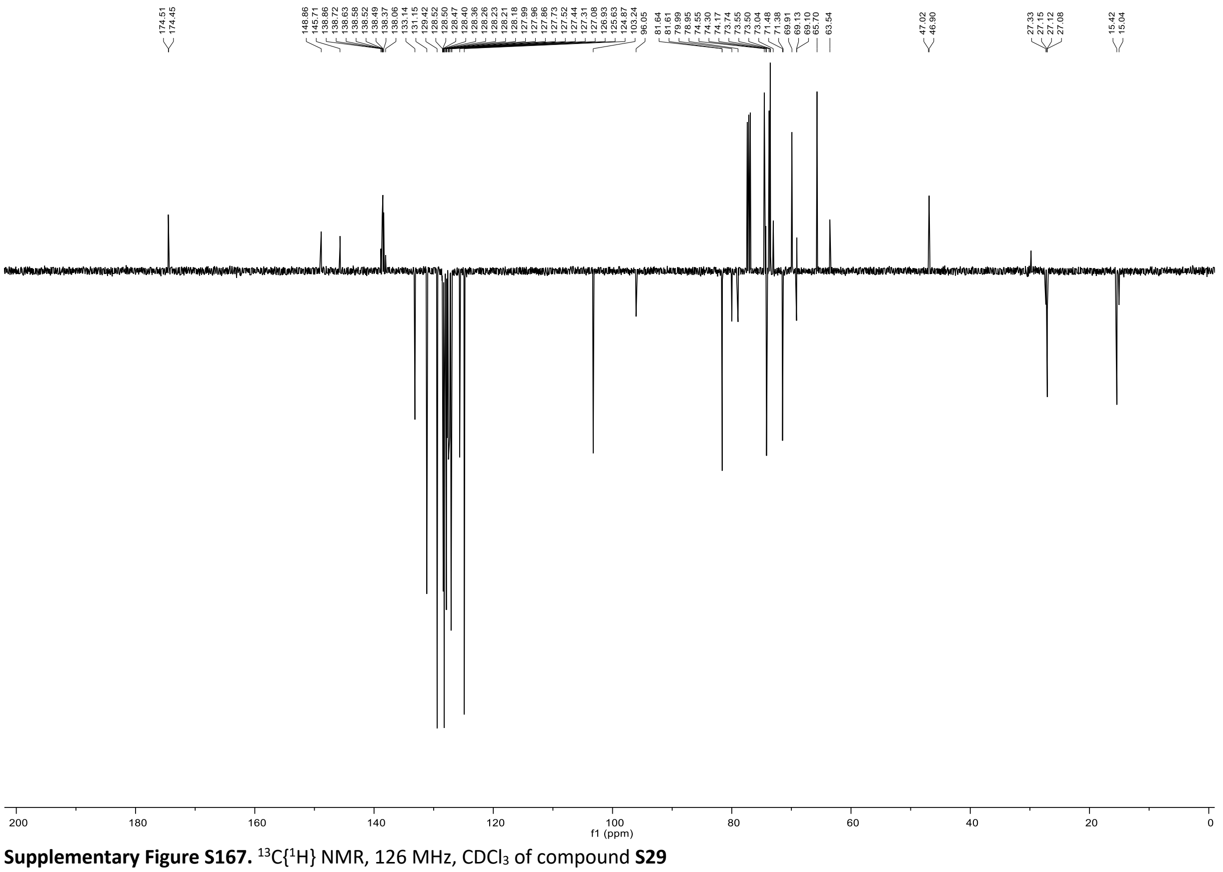

Supplementary Figure S167. $^{13}C\{^{1}H\}$ NMR, 126 MHz, $CDCl_3$ of compound **S29**

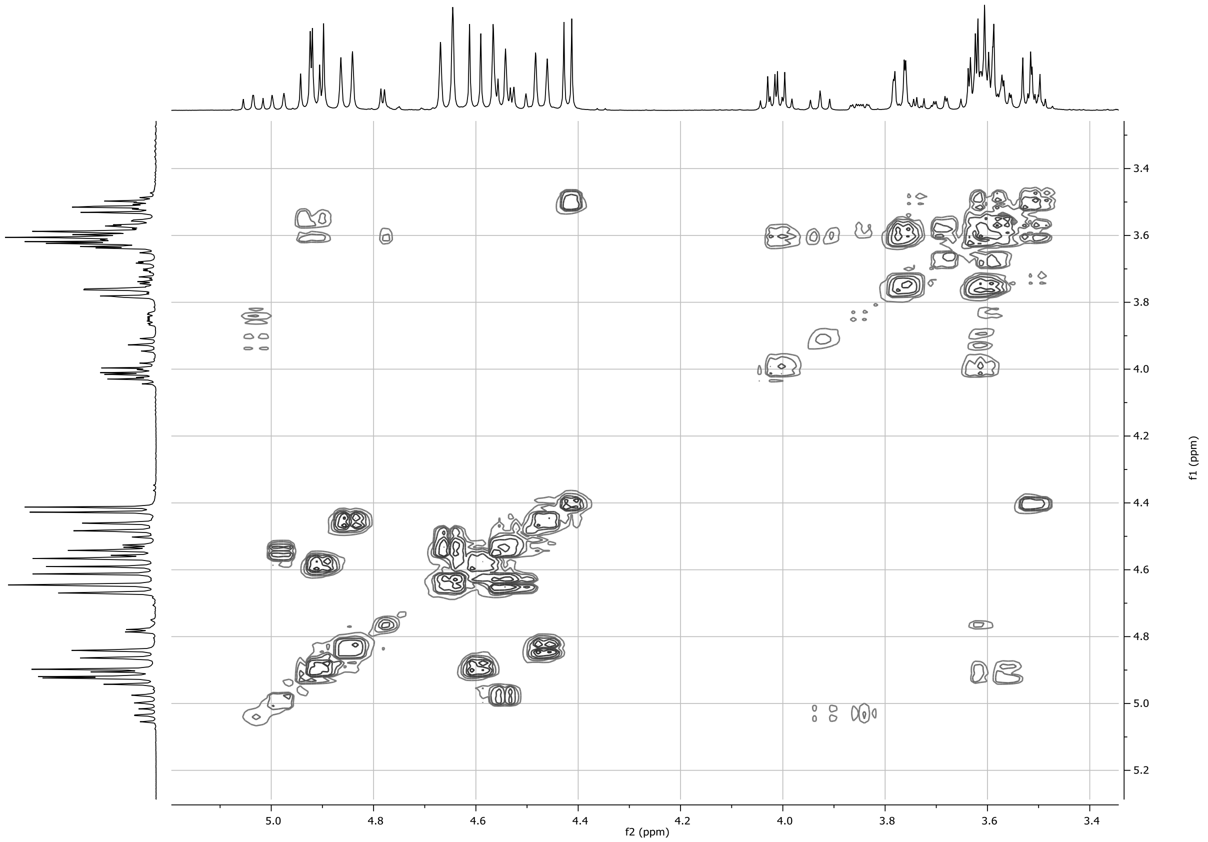

Supplementary Figure S168. HH-COSY NMR, $CDCl_3$ of compound **S29**

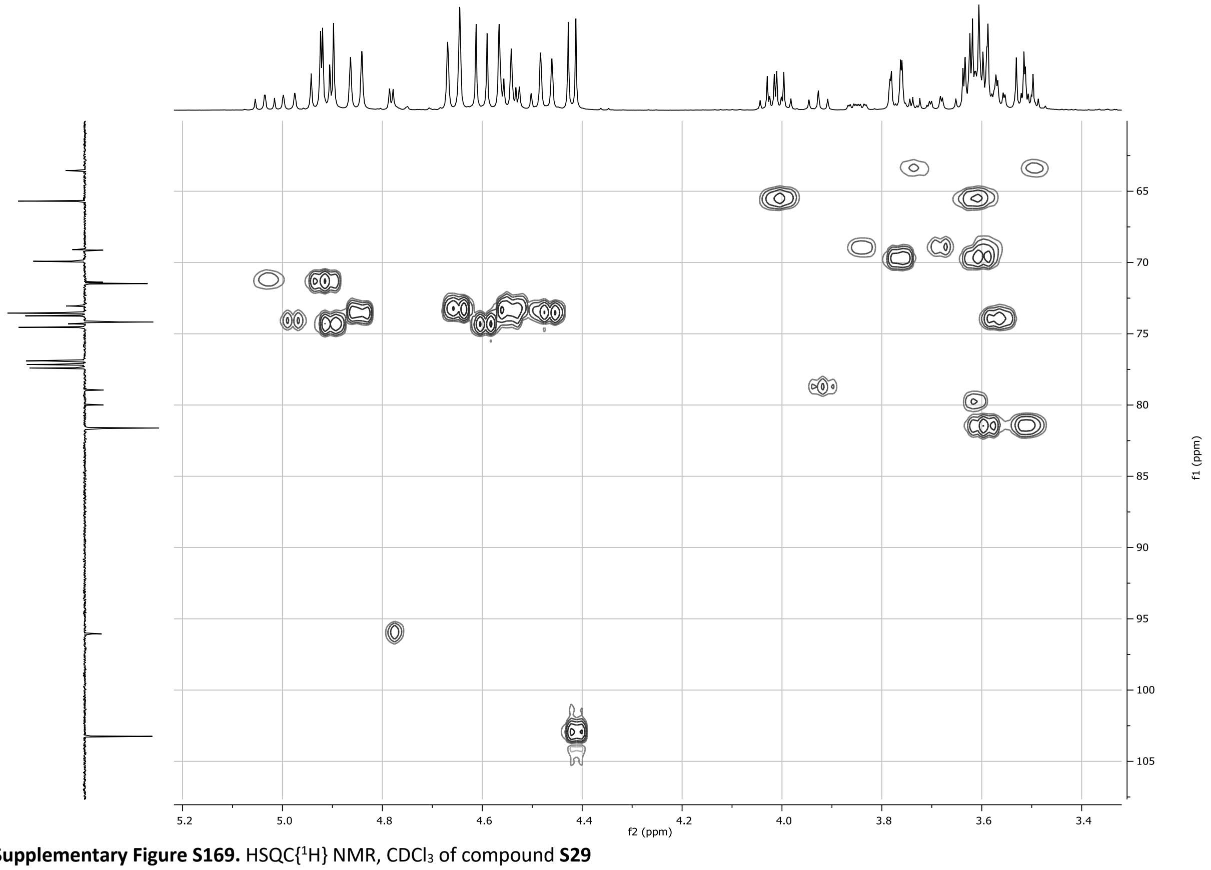

Supplementary Figure S169. HSQC{^{1}H} NMR, $CDCl_3$ of compound **S29**

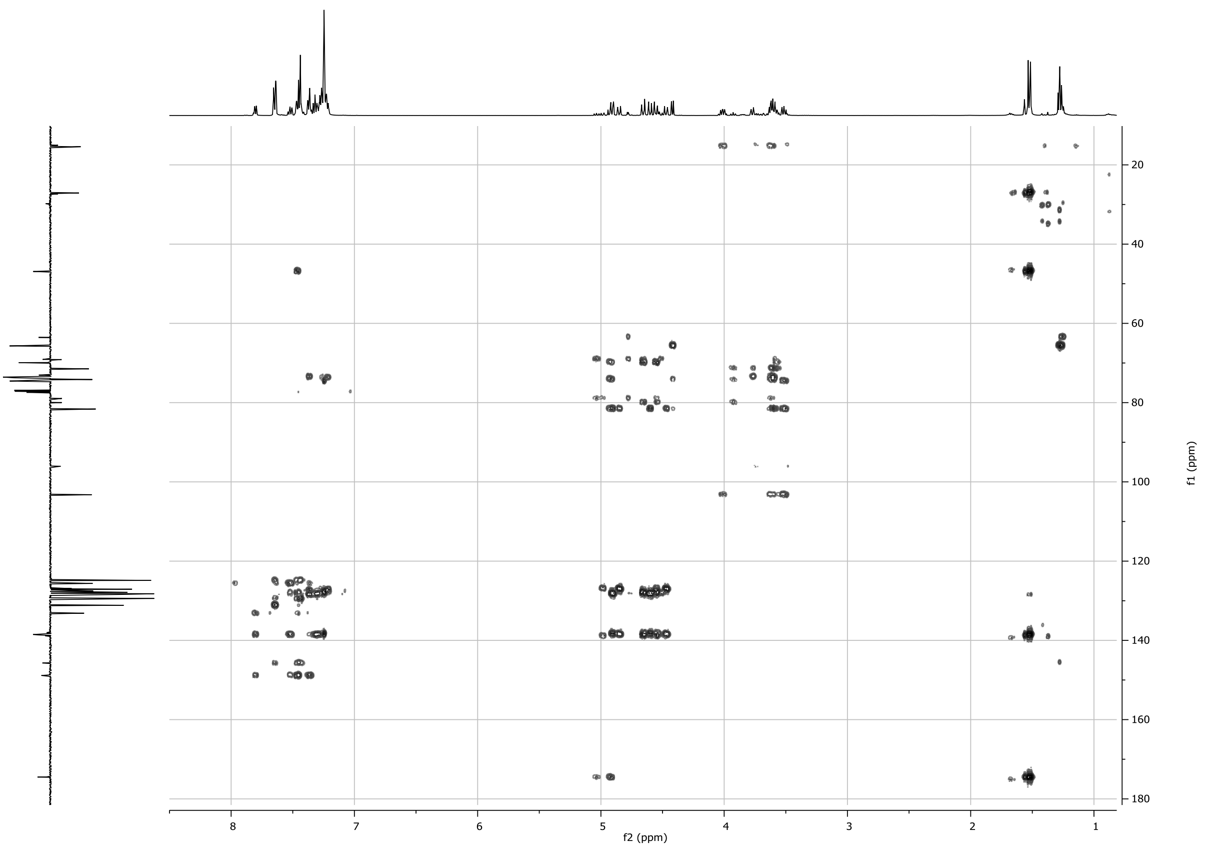

Supplementary Figure S170. HMBC{^{1}H} NMR, $CDCl_3$ of compound **S29**

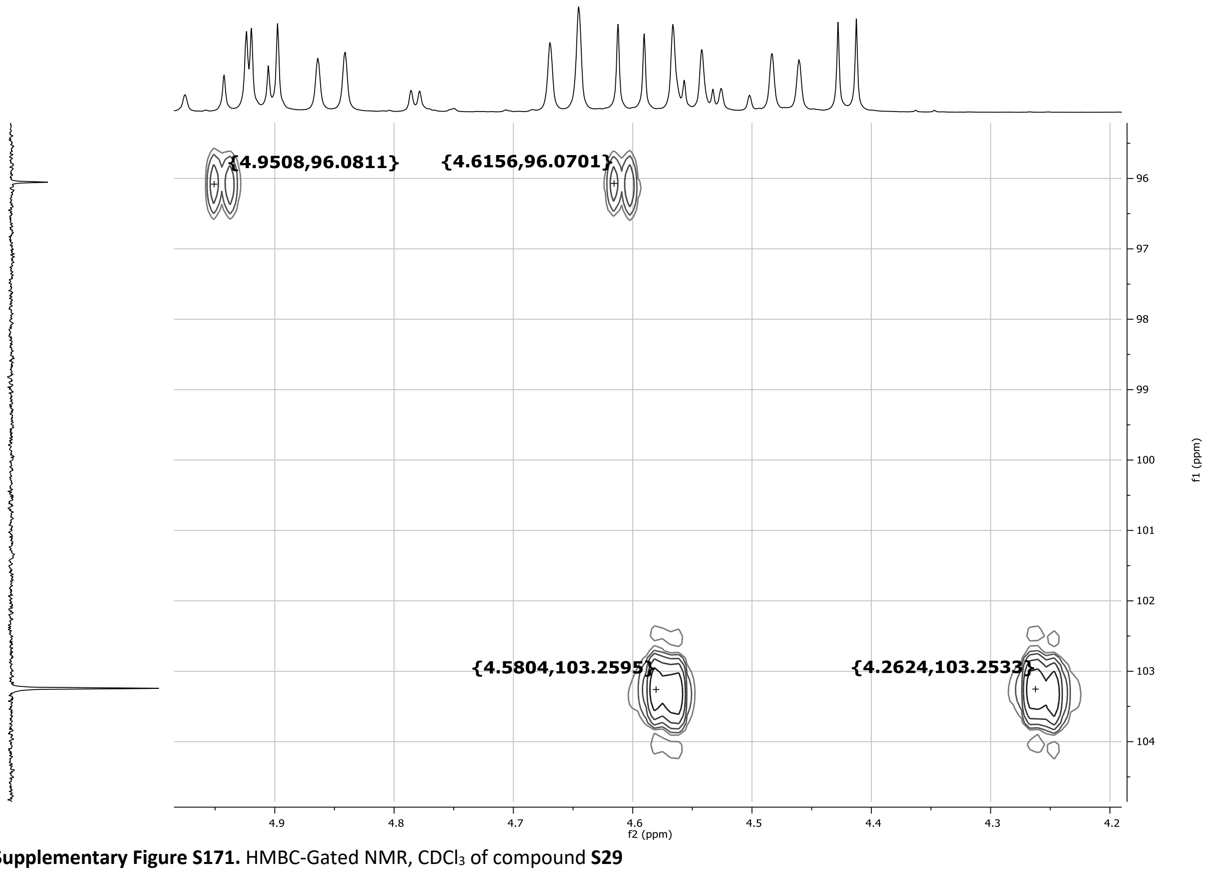

Supplementary Figure S171. HMBC-Gated NMR, $CDCl_3$ of compound **S29**

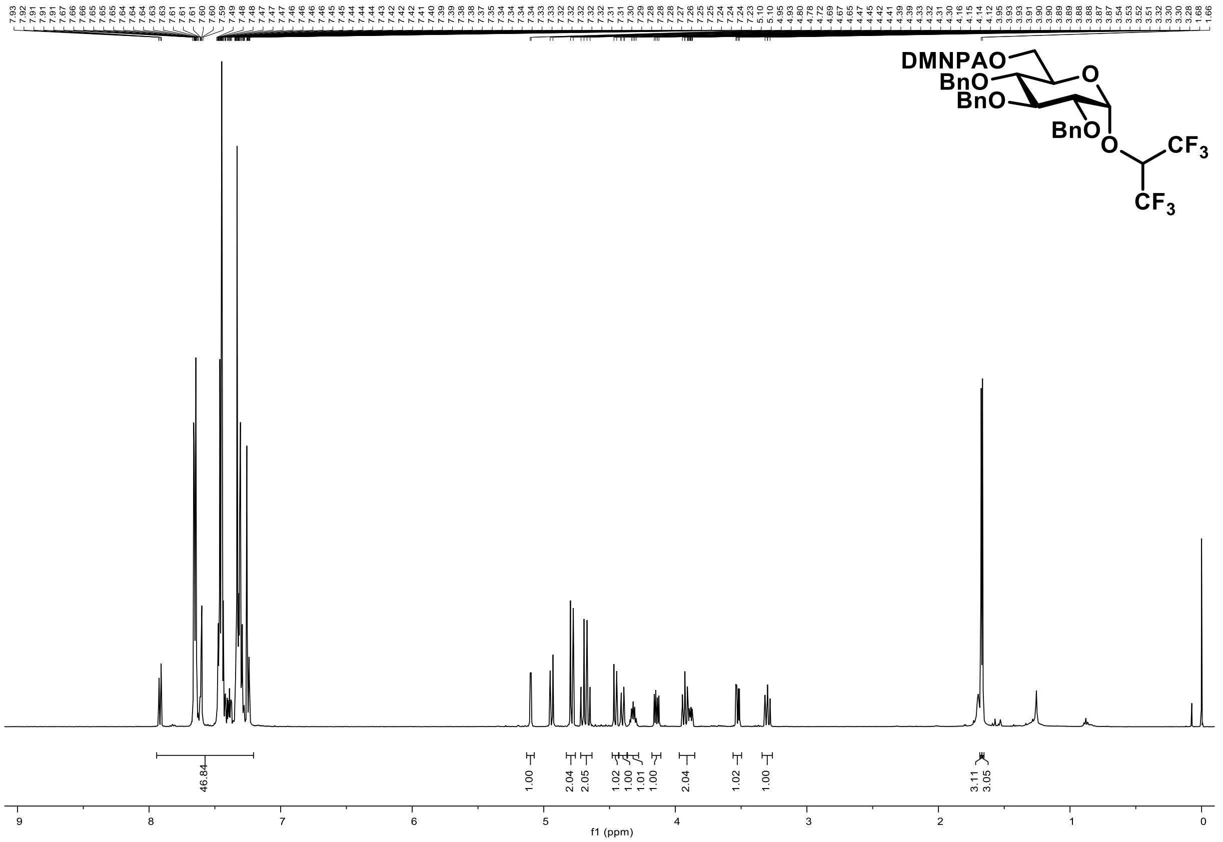

Supplementary Figure S172. ^{1}H NMR, 400 MHz, $CDCl_3$ of compound **S30**

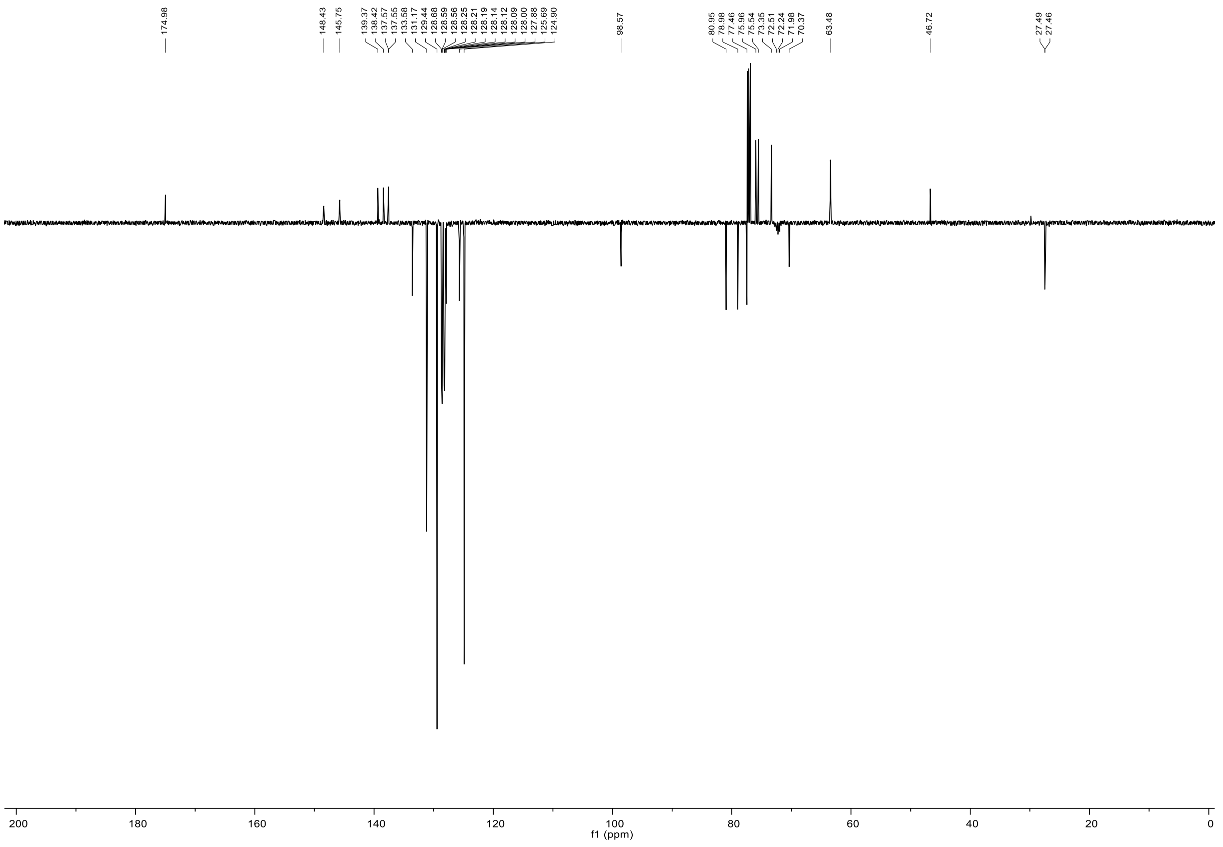

Supplementary Figure S173. $^{13}C\{^{1}H\}$ NMR, 101 MHz, $CDCl_3$ of compound **S30**

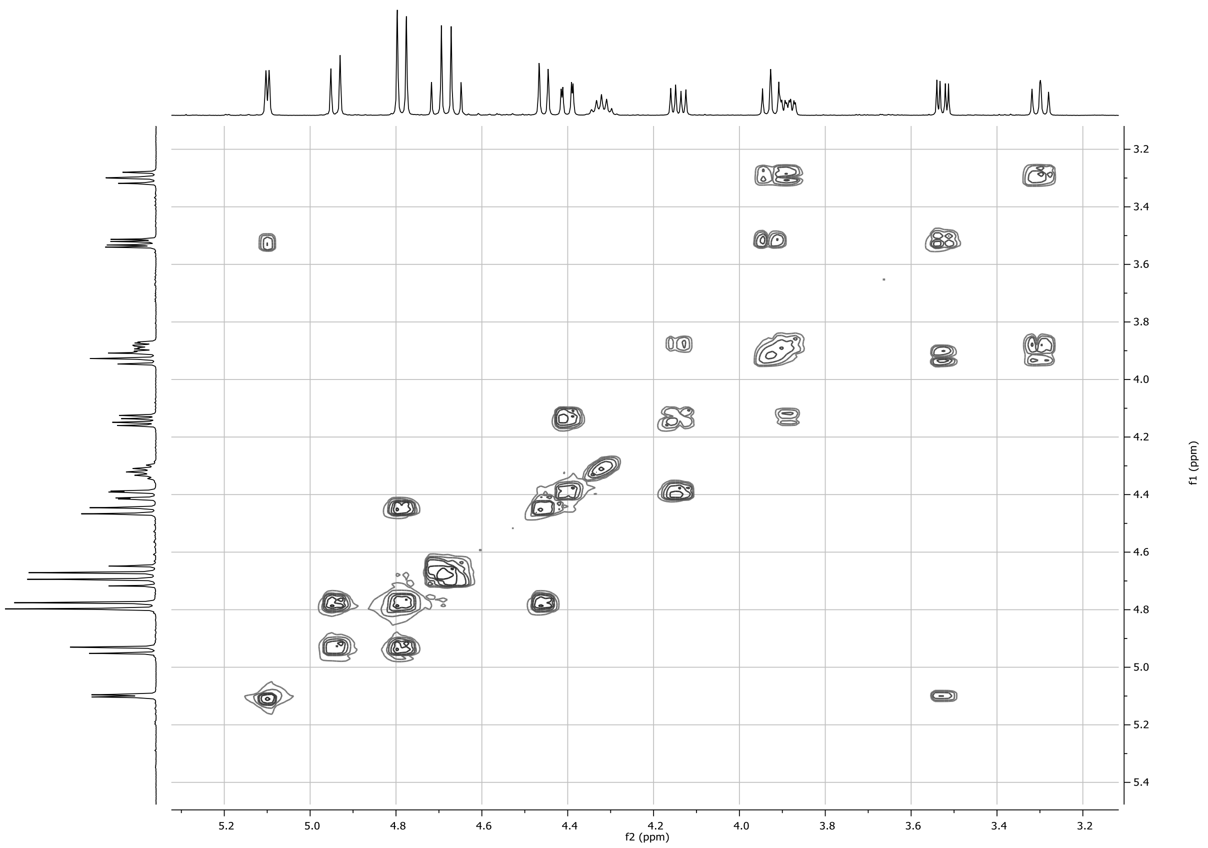

Supplementary Figure S174. HH-COSY NMR, $CDCl_3$ of compound **S30**

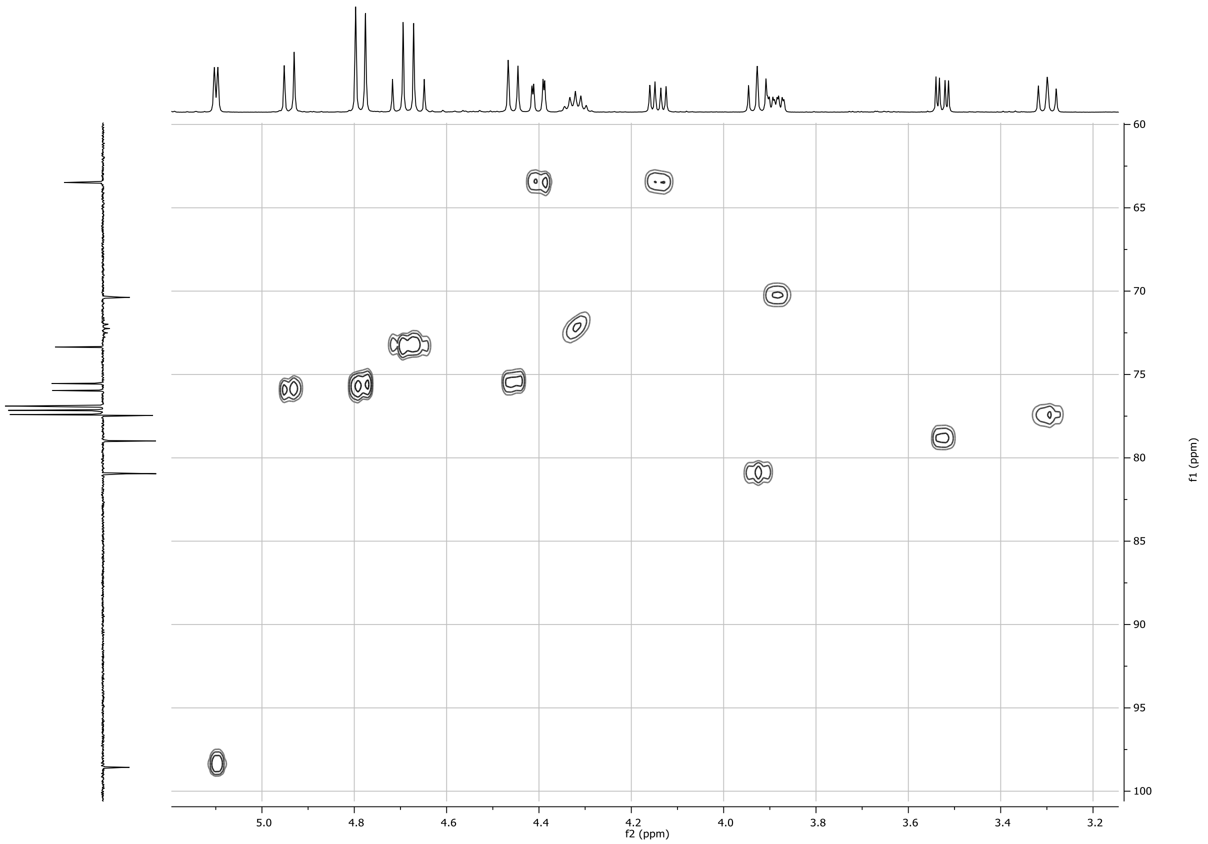

Supplementary Figure S175. HSQC{^{1}H} NMR, $CDCl_3$ of compound **S30**

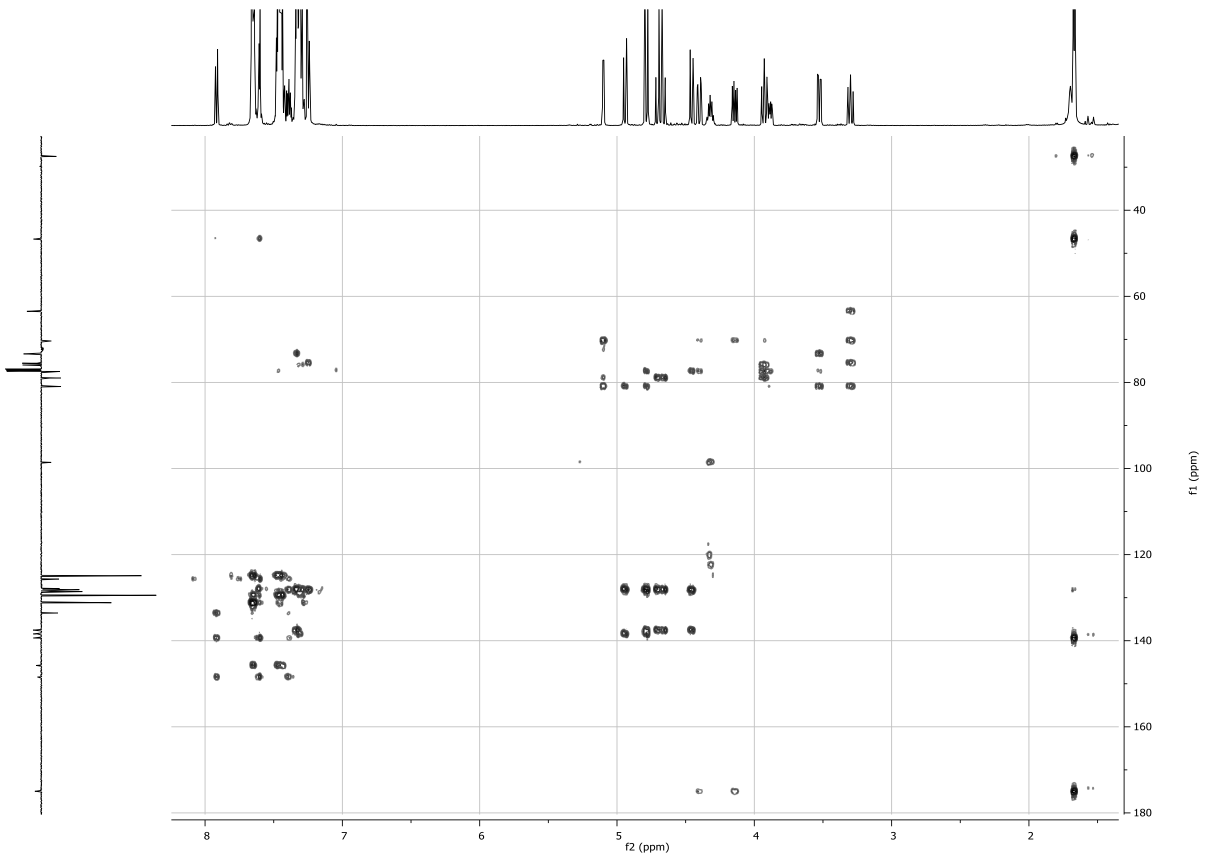

Supplementary Figure S176. HMBC{^{1}H} NMR, $CDCl_3$ of compound **S30**

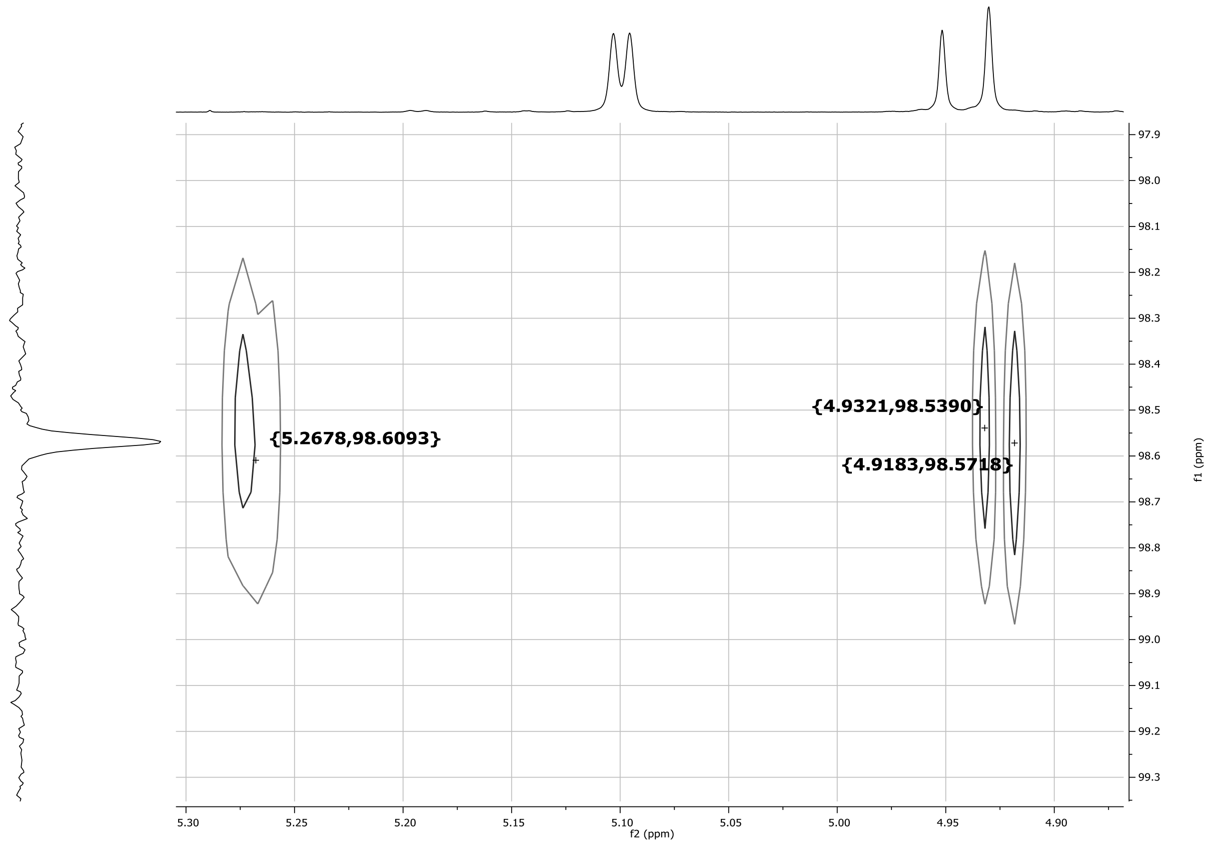

Supplementary Figure S177. HMBC-Gated NMR, $CDCl_3$ of compound **S30**

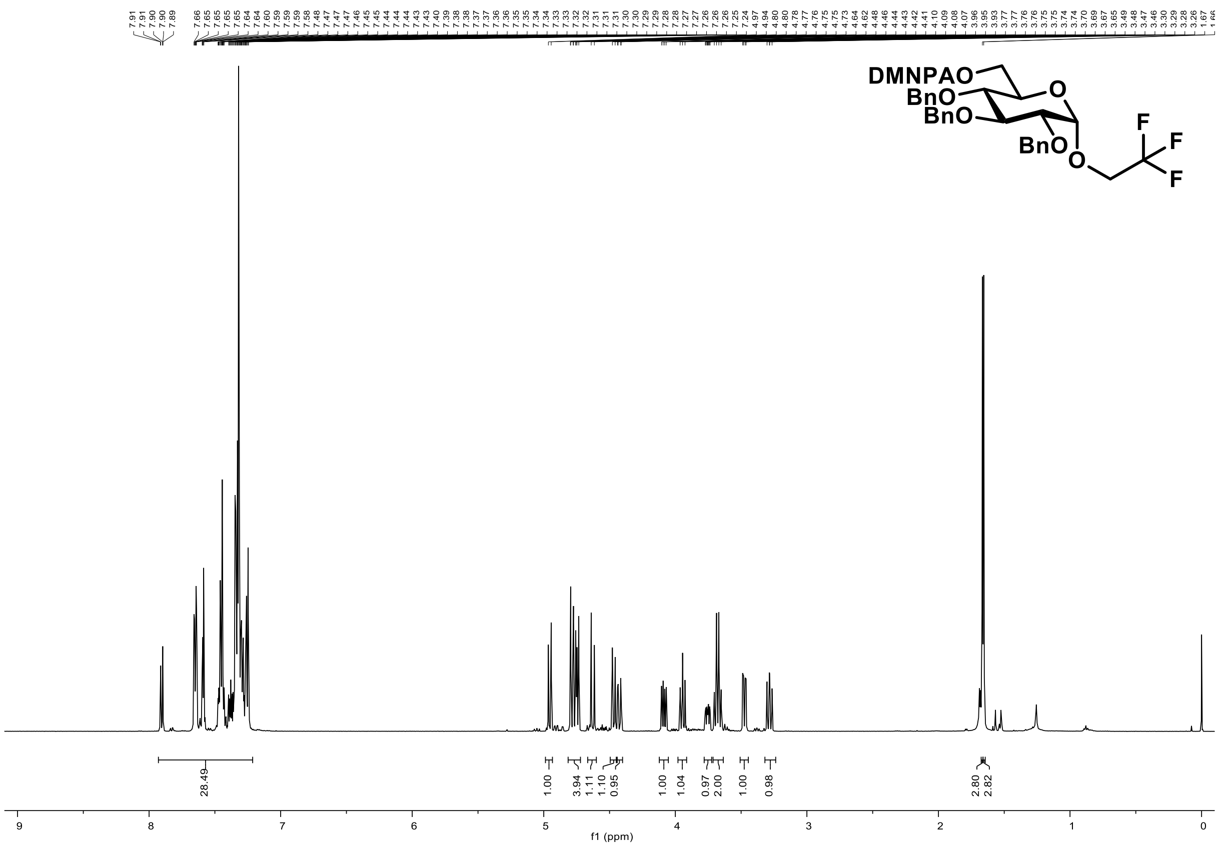

Supplementary Figure S178. ^{1}H NMR, 500 MHz, $CDCl_3$ of compound **S31**

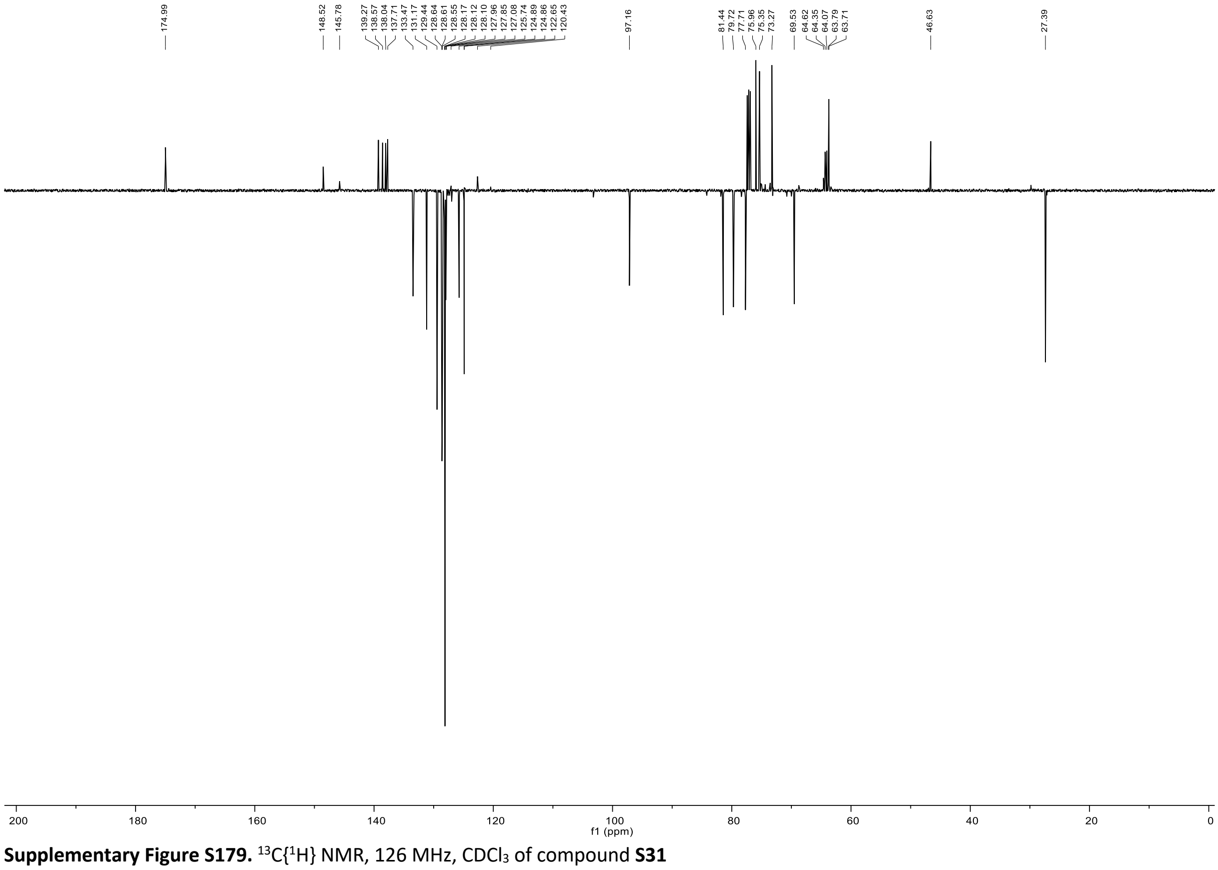

Supplementary Figure S179. $^{13}C\{^{1}H\}$ NMR, 126 MHz, $CDCl_3$ of compound **S31**

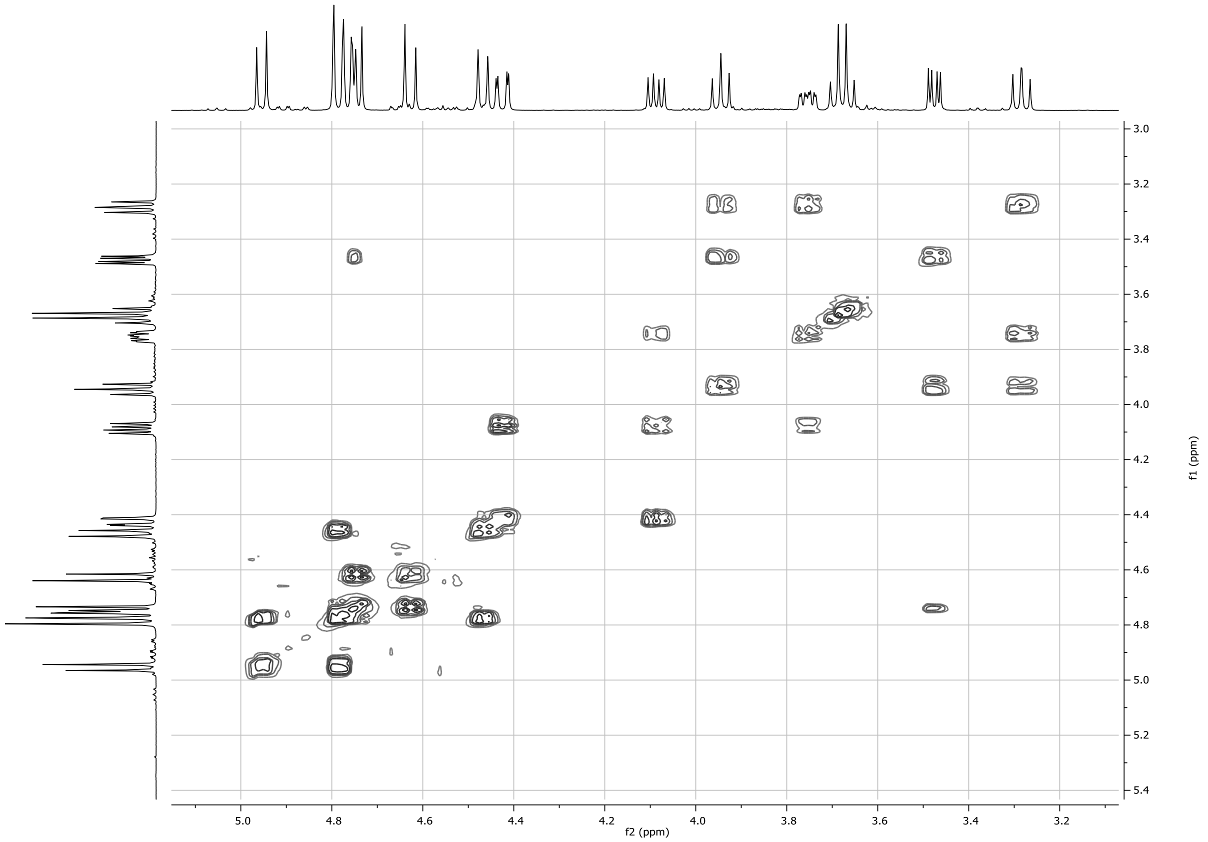

Supplementary Figure S180. HH-COSY NMR, $CDCl_3$ of compound **S31**

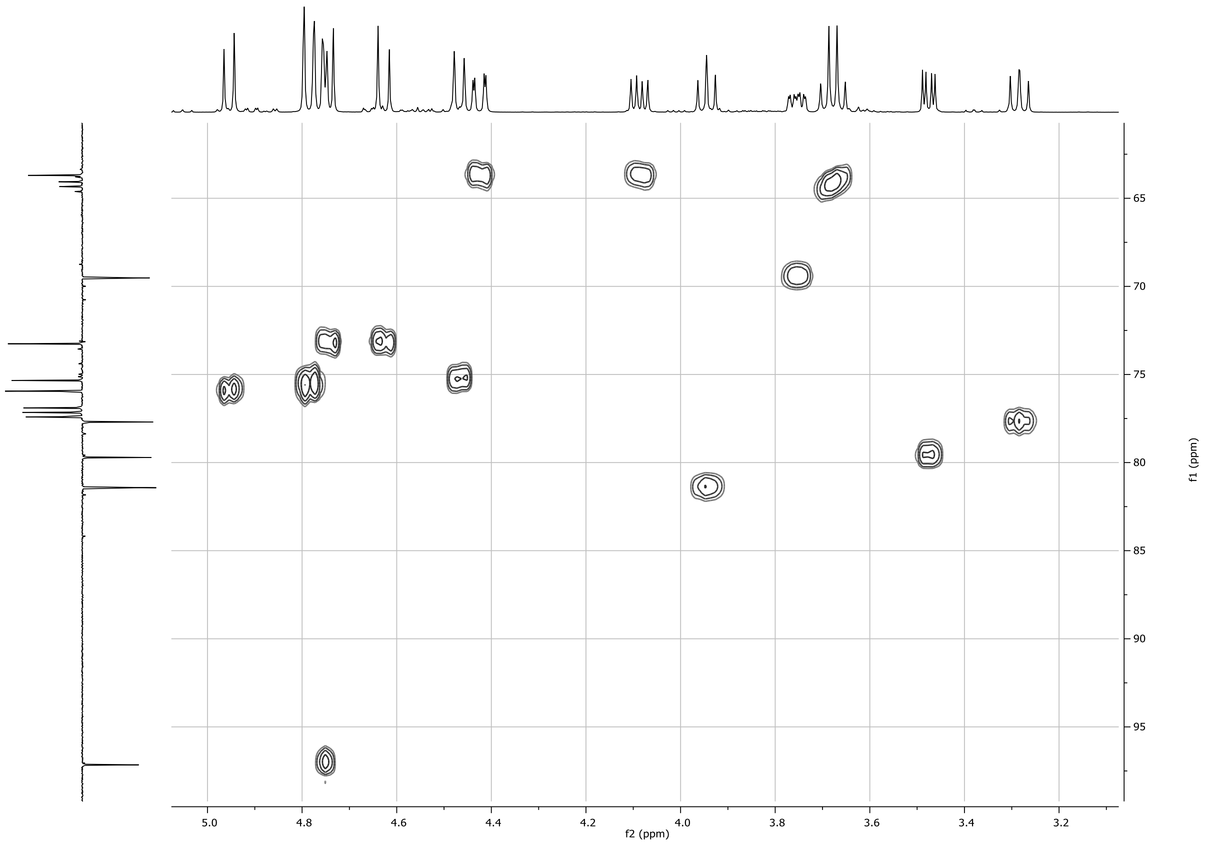

Supplementary Figure S181. HSQC{^{1}H} NMR, $CDCl_3$ of compound **S31**

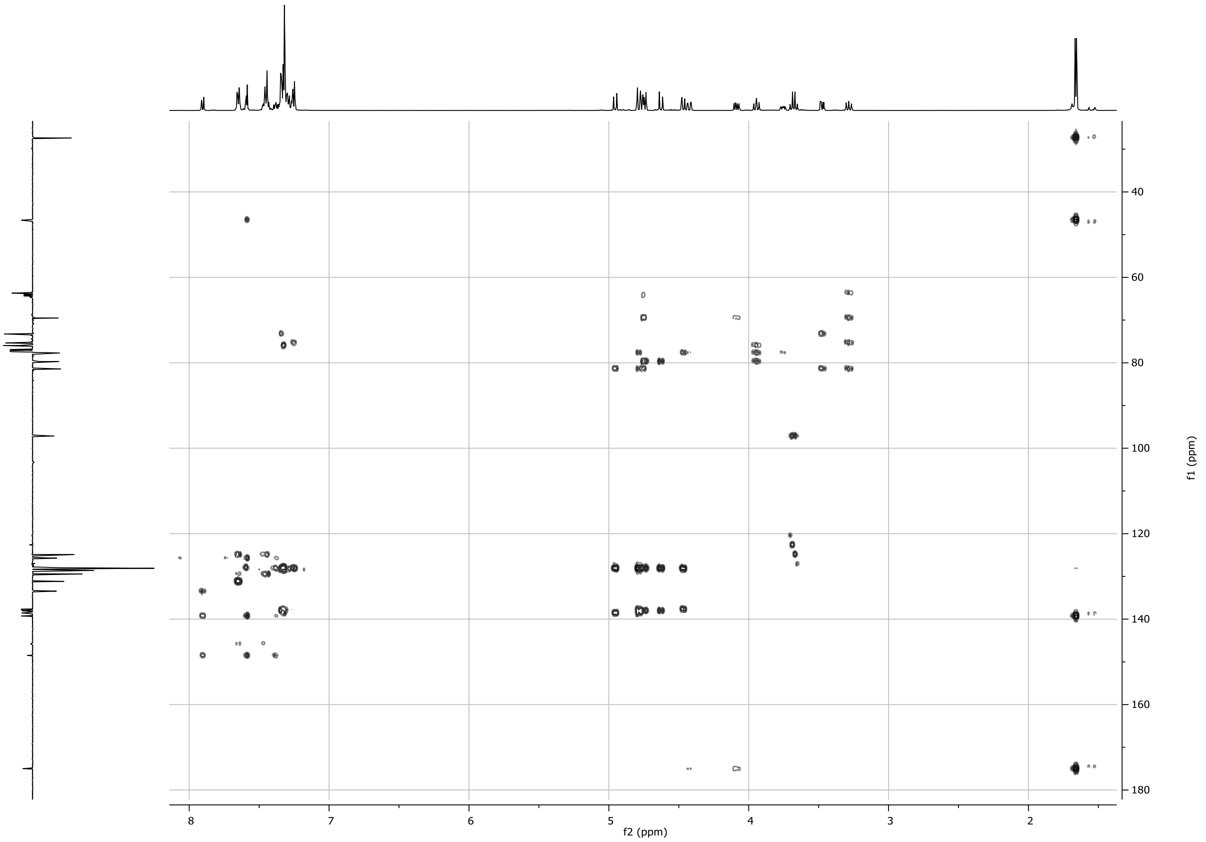

Supplementary Figure S182. HMBC{^{1}H} NMR, $CDCl_3$ of compound **S31**

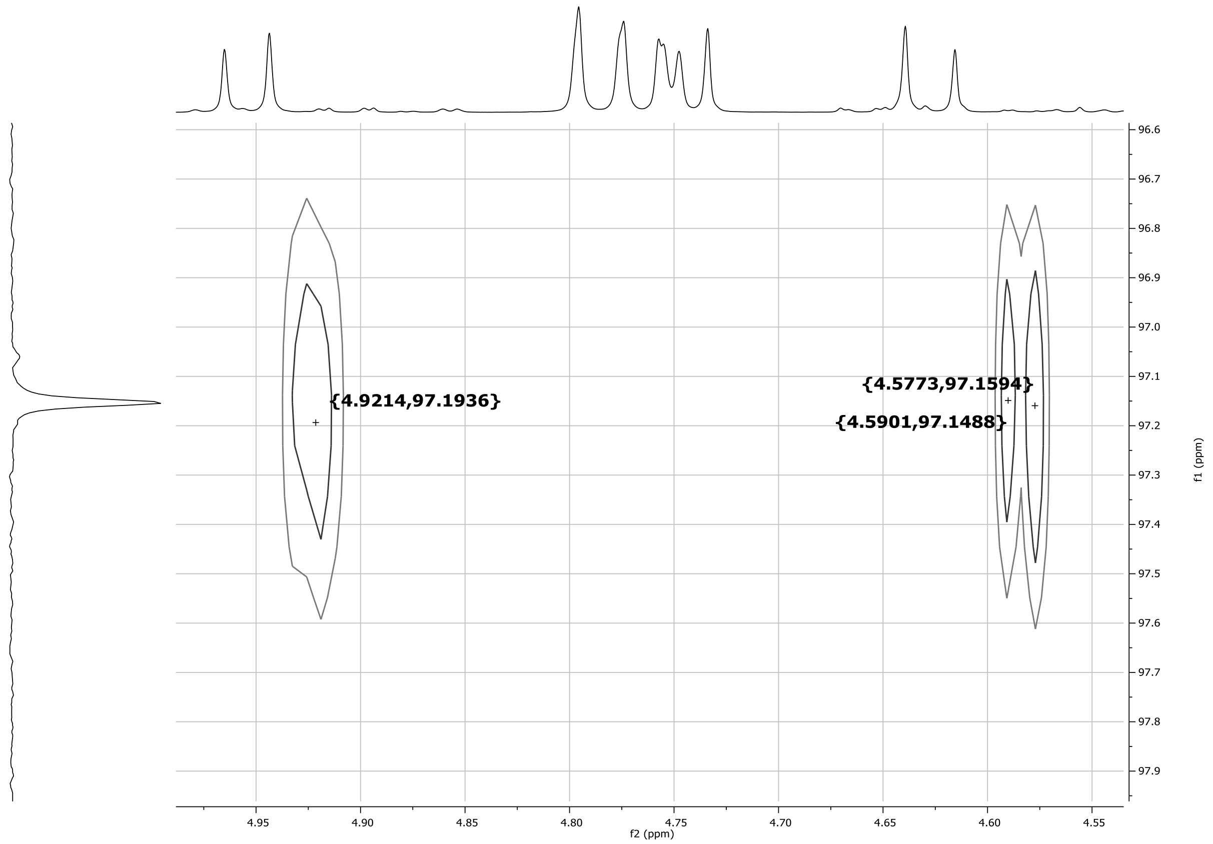

Supplementary Figure S183. HMBC-Gated NMR, $CDCl_3$ of compound **S31**

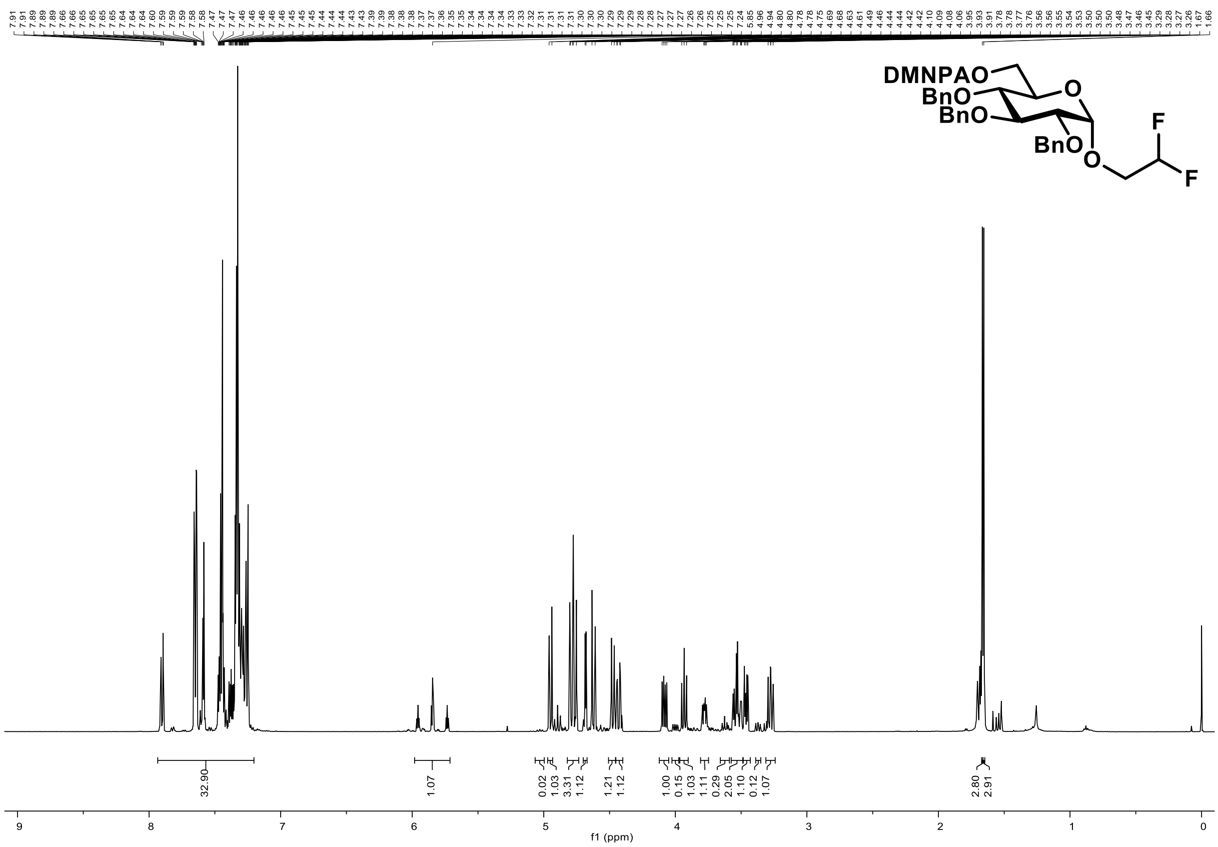

Supplementary Figure S184. ^{1}H NMR, 500 MHz, $CDCl_3$ of compound **S32**

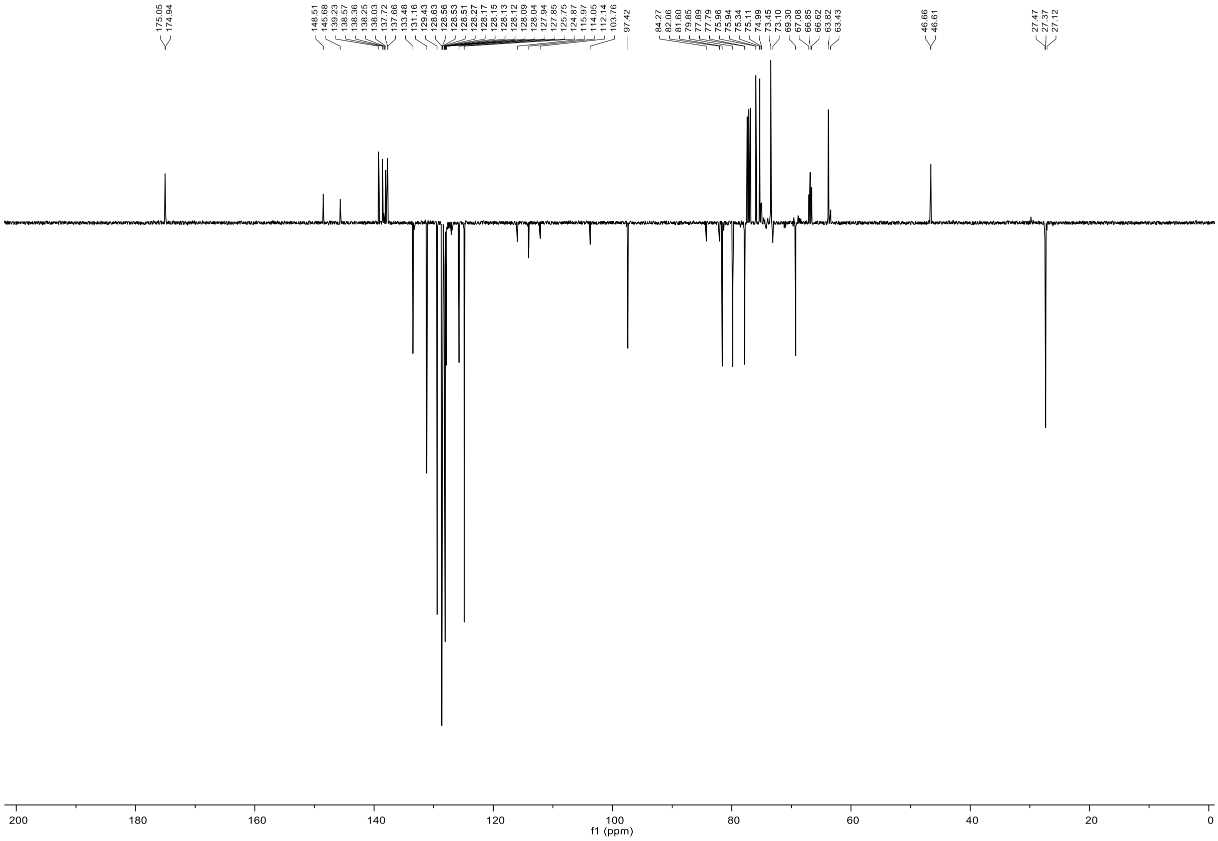

Supplementary Figure S185. $^{13}C\{^{1}H\}$ NMR, 126 MHz, $CDCl_3$ of compound **S32**

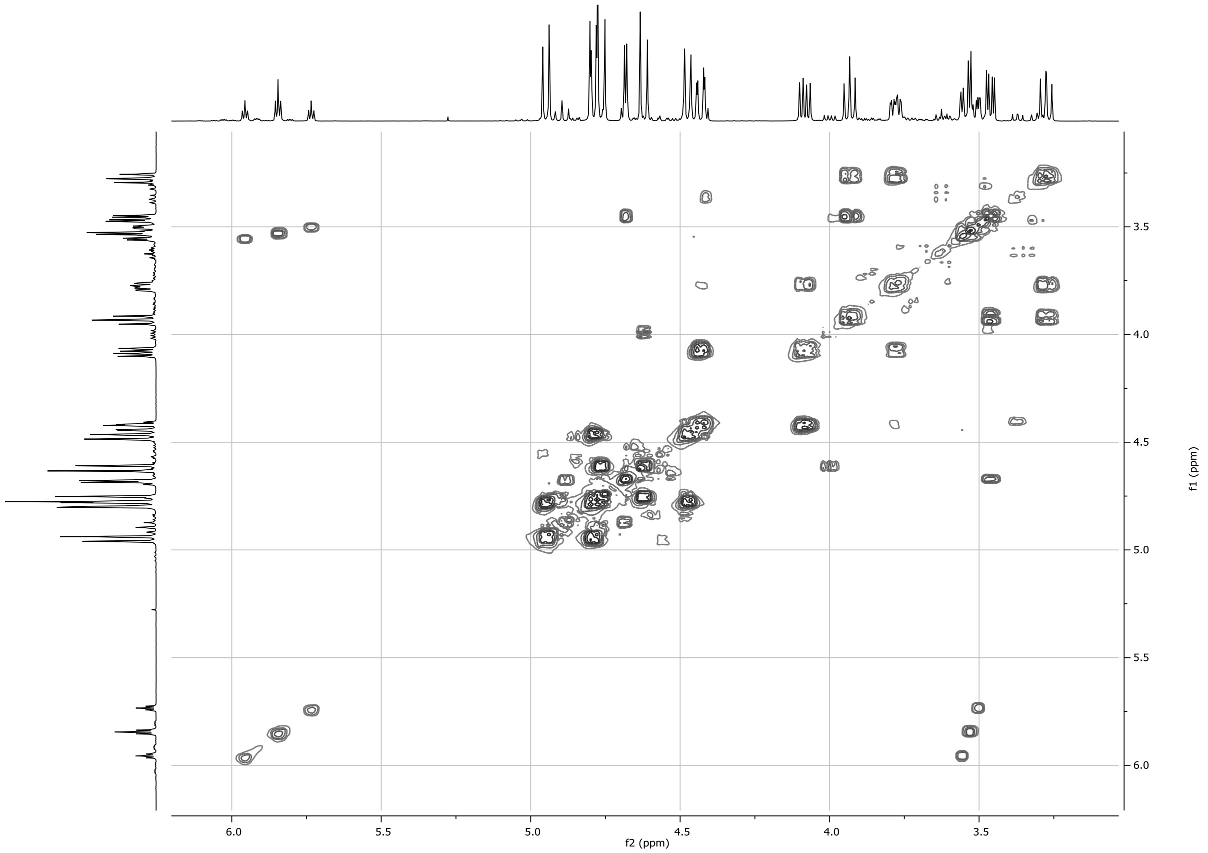

Supplementary Figure S186. HH-COSY NMR, $CDCl_3$ of compound **S32**

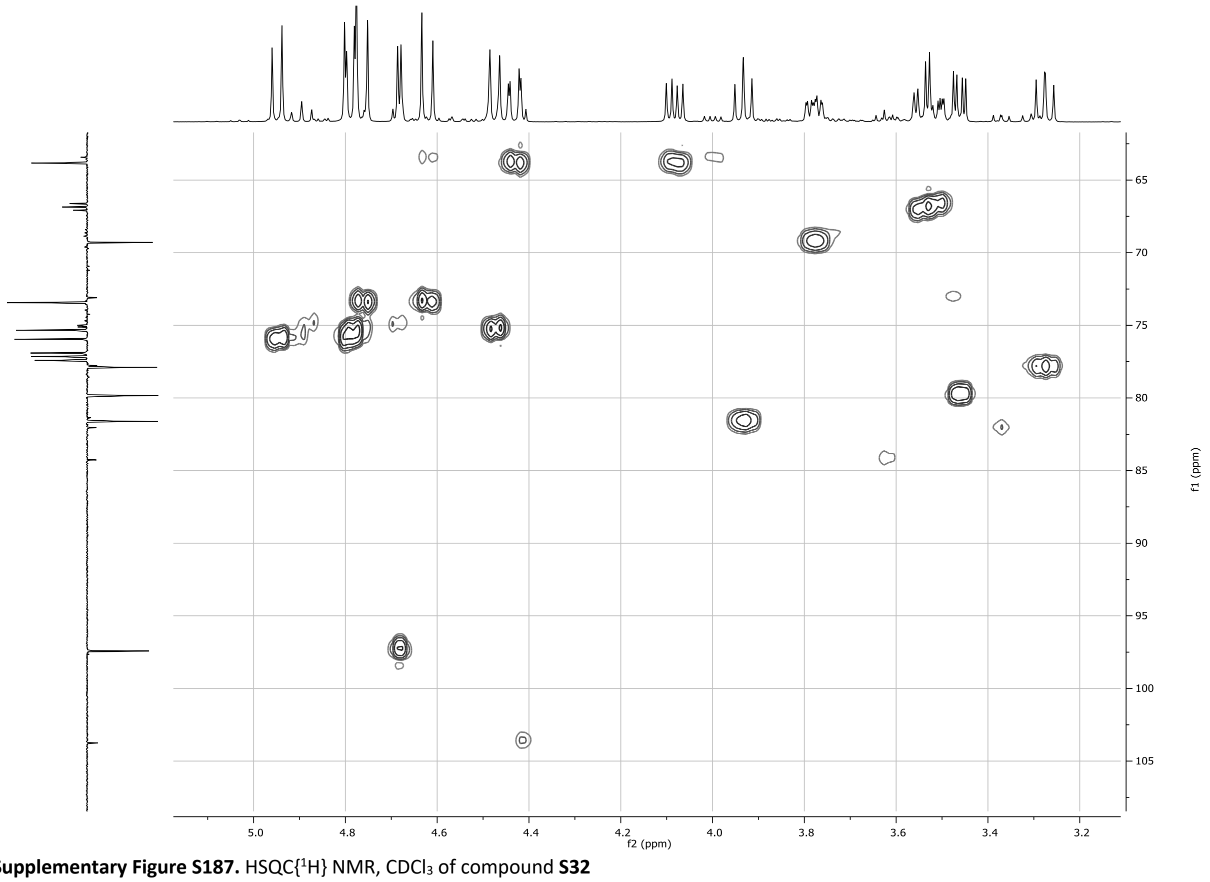

Supplementary Figure S187. HSQC{^{1}H} NMR, $CDCl_3$ of compound **S32**

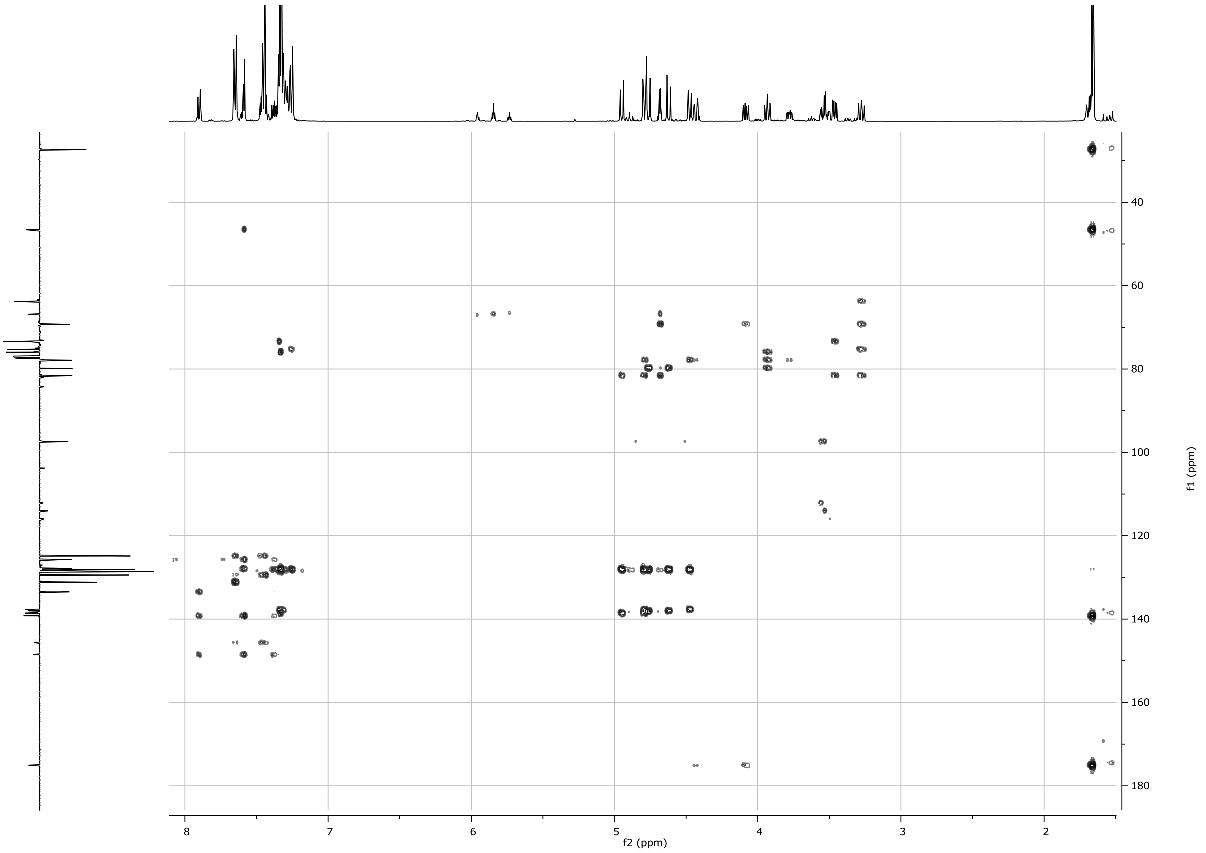

Supplementary Figure S188. HMBC{¹H} NMR, $CDCl_3$ of compound **S32**

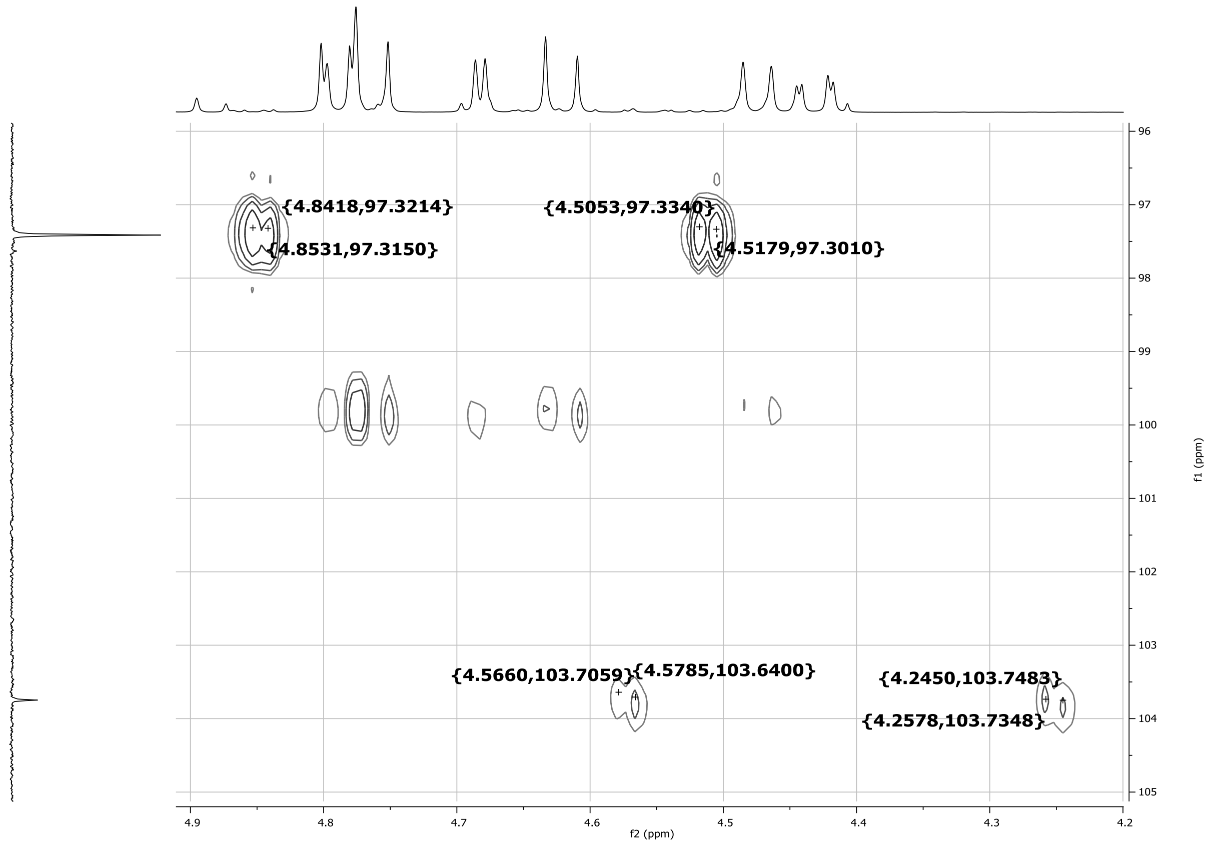

Supplementary Figure S189. HMBC-Gated NMR, $CDCl_3$ of compound **S32**

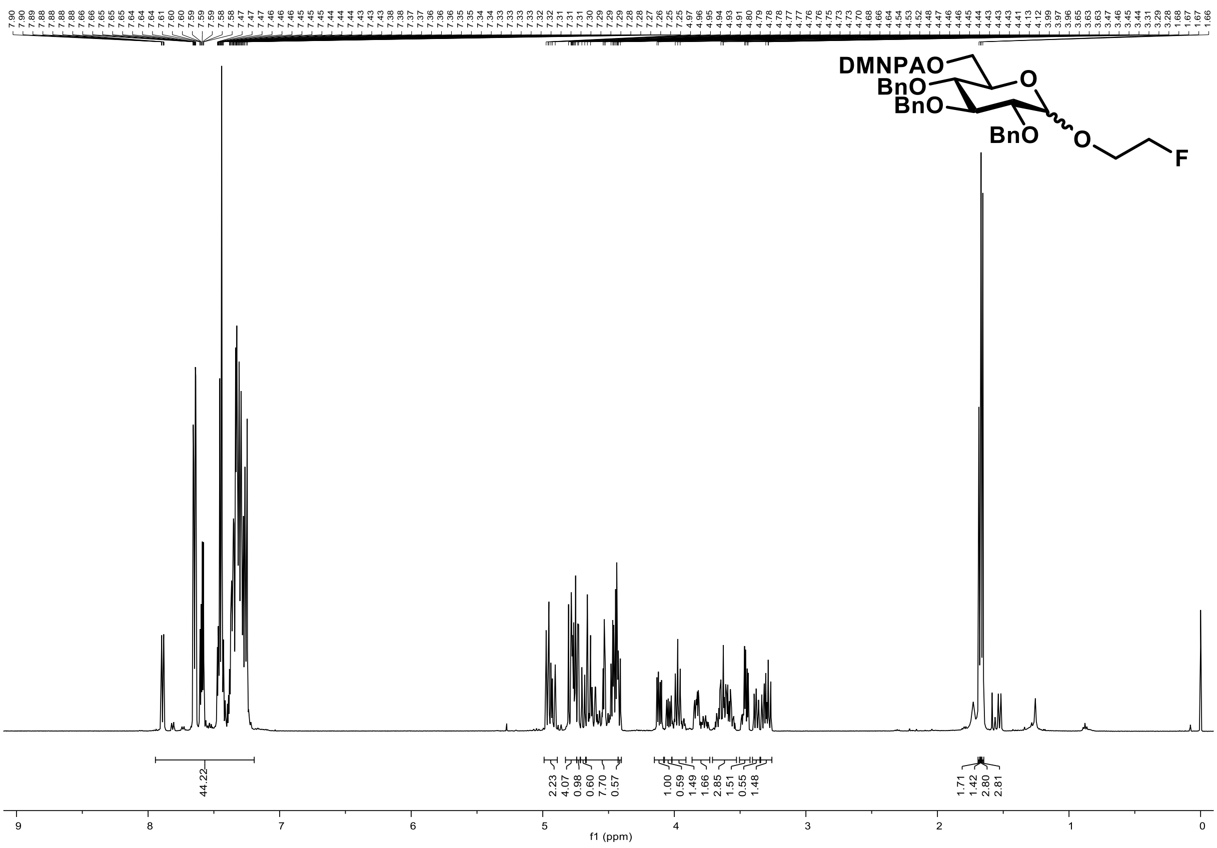

Supplementary Figure S190. ^{1}H NMR, 500 MHz, $CDCl_3$ of compound **S33**

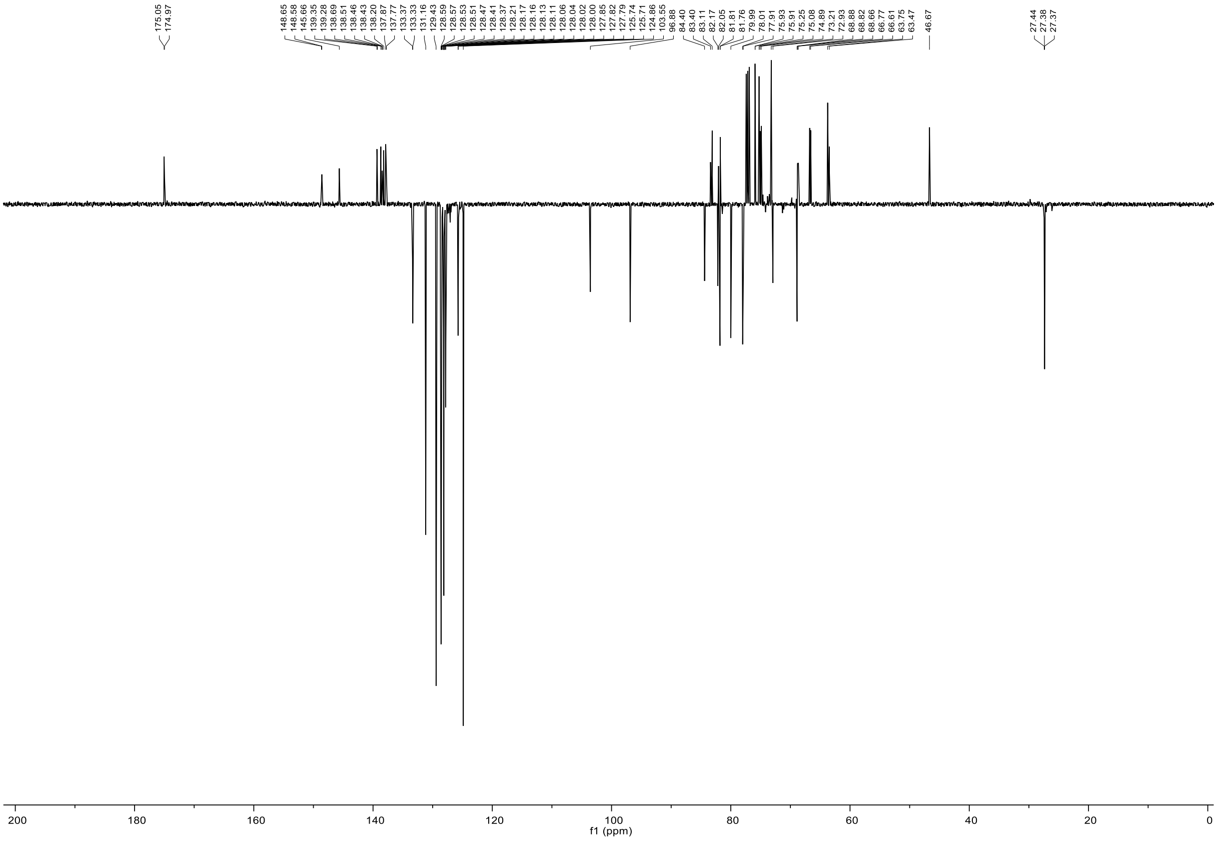

Supplementary Figure S191. $^{13}C\{^{1}H\}$ NMR, 126 MHz, $CDCl_3$ of compound **S33**

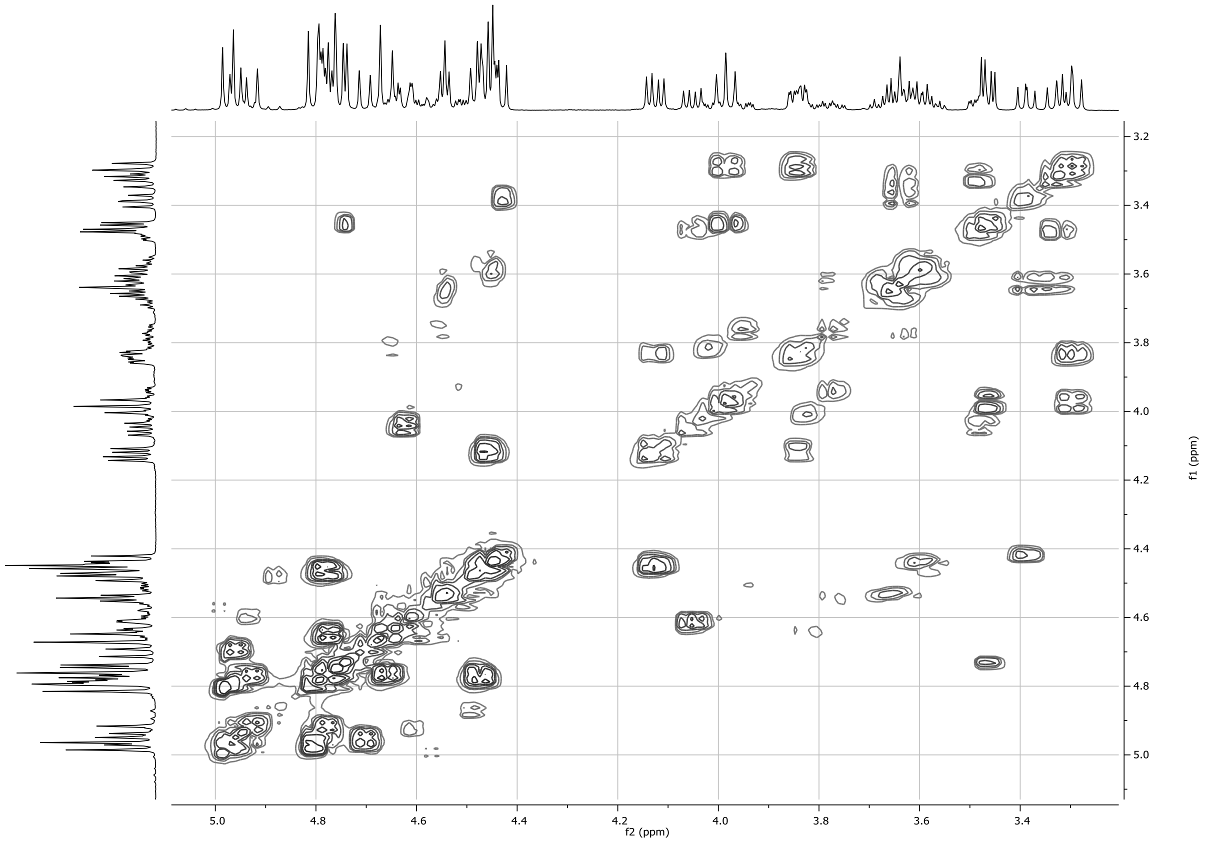

Supplementary Figure S192. HH-COSY NMR, $CDCl_3$ of compound **S33**

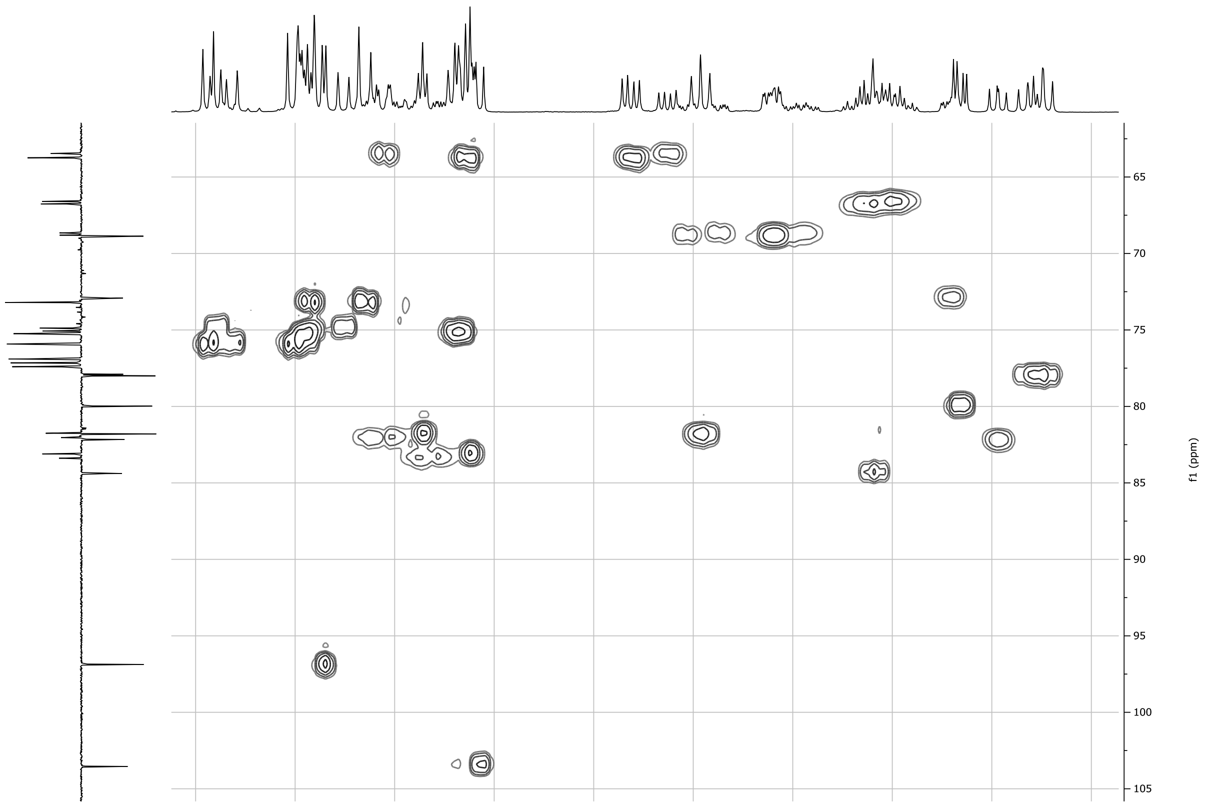

Supplementary Figure S193. HSQC{^{1}H} NMR, $CDCl_3$ of compound **S33**

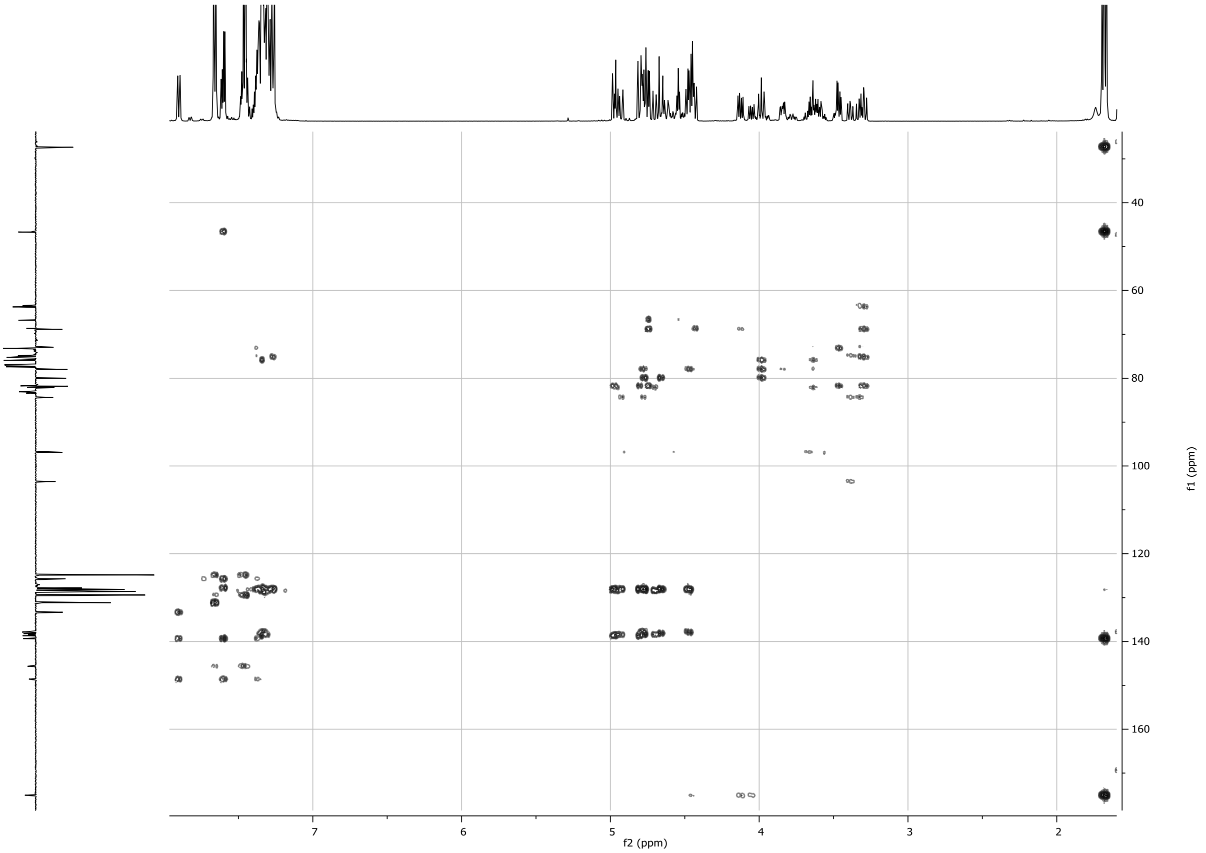

Supplementary Figure S194. HMBC{^{1}H} NMR, $CDCl_3$ of compound **S33**

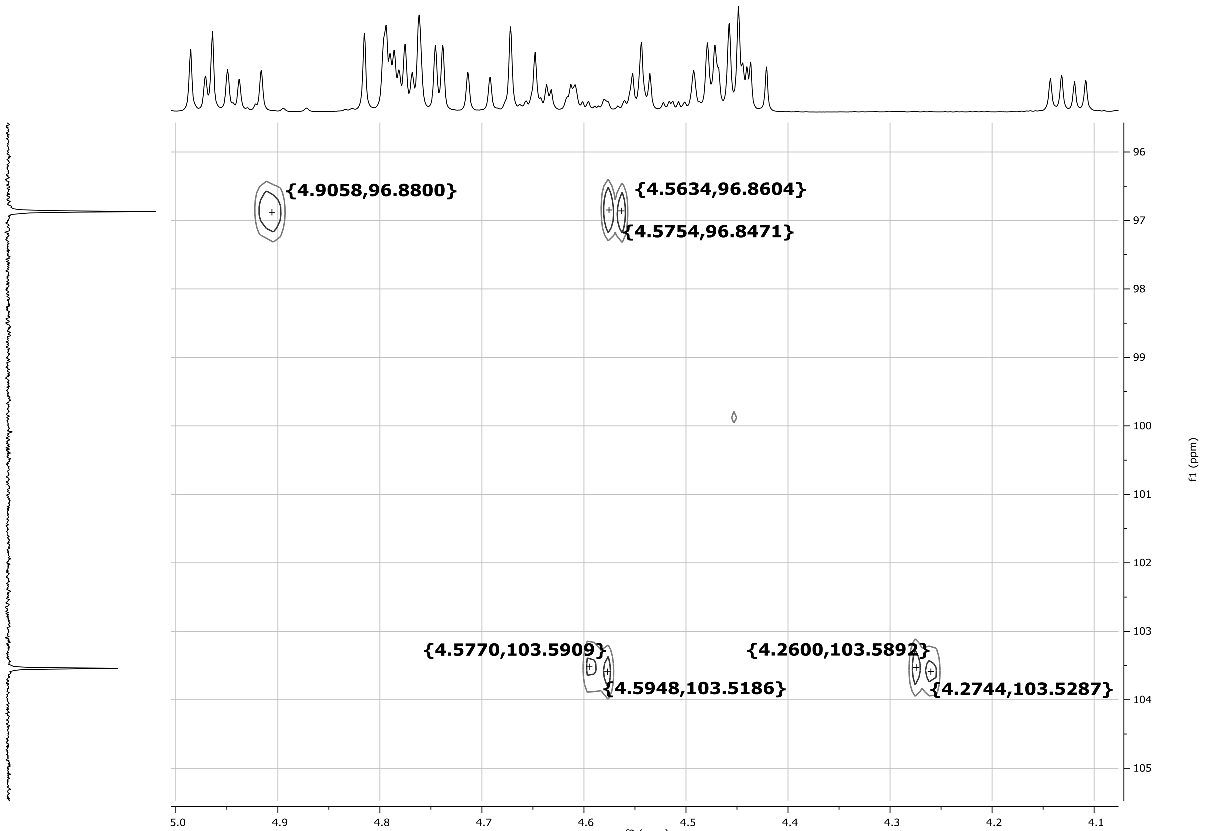

Supplementary Figure S195. HMBC-Gated NMR, $CDCl_3$ of compound **S33**

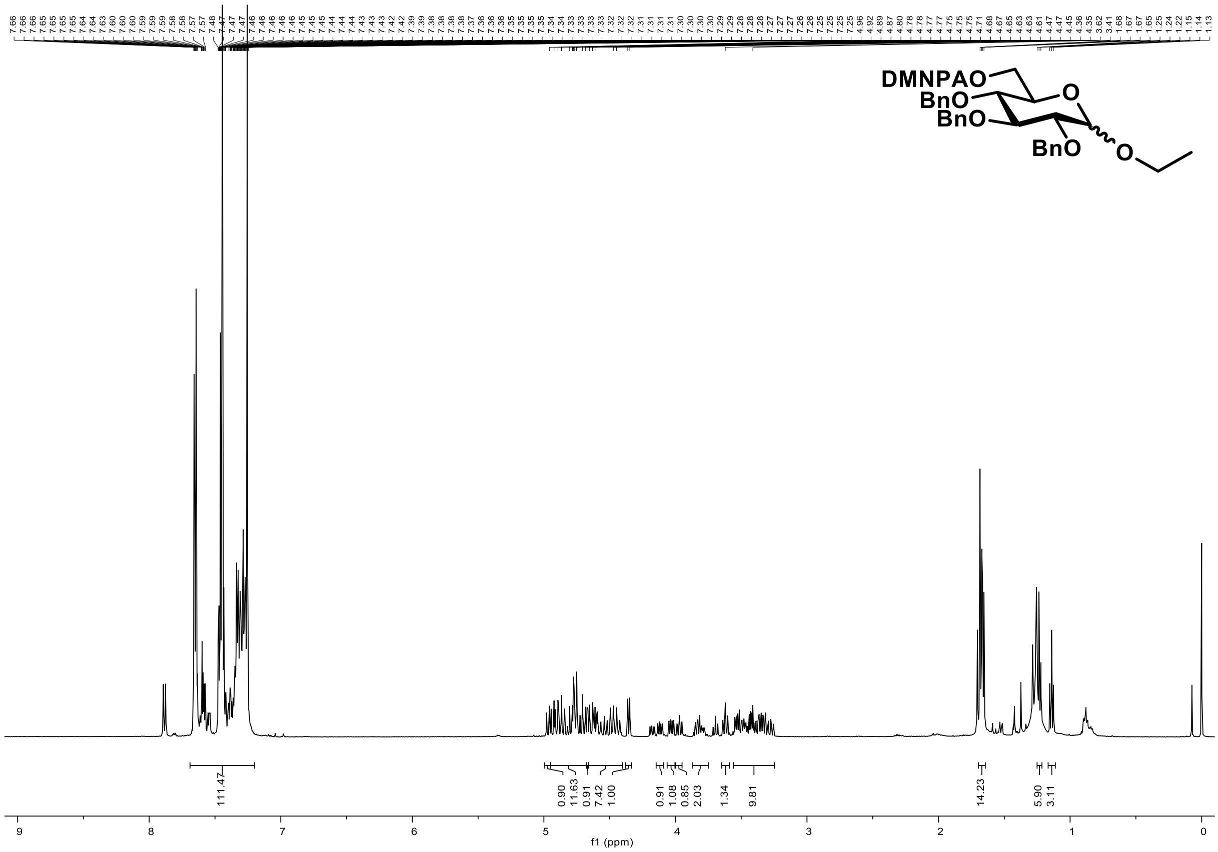

Supplementary Figure S196. ^{1}H NMR, 400 MHz, $CDCl_3$ of compound **S34**

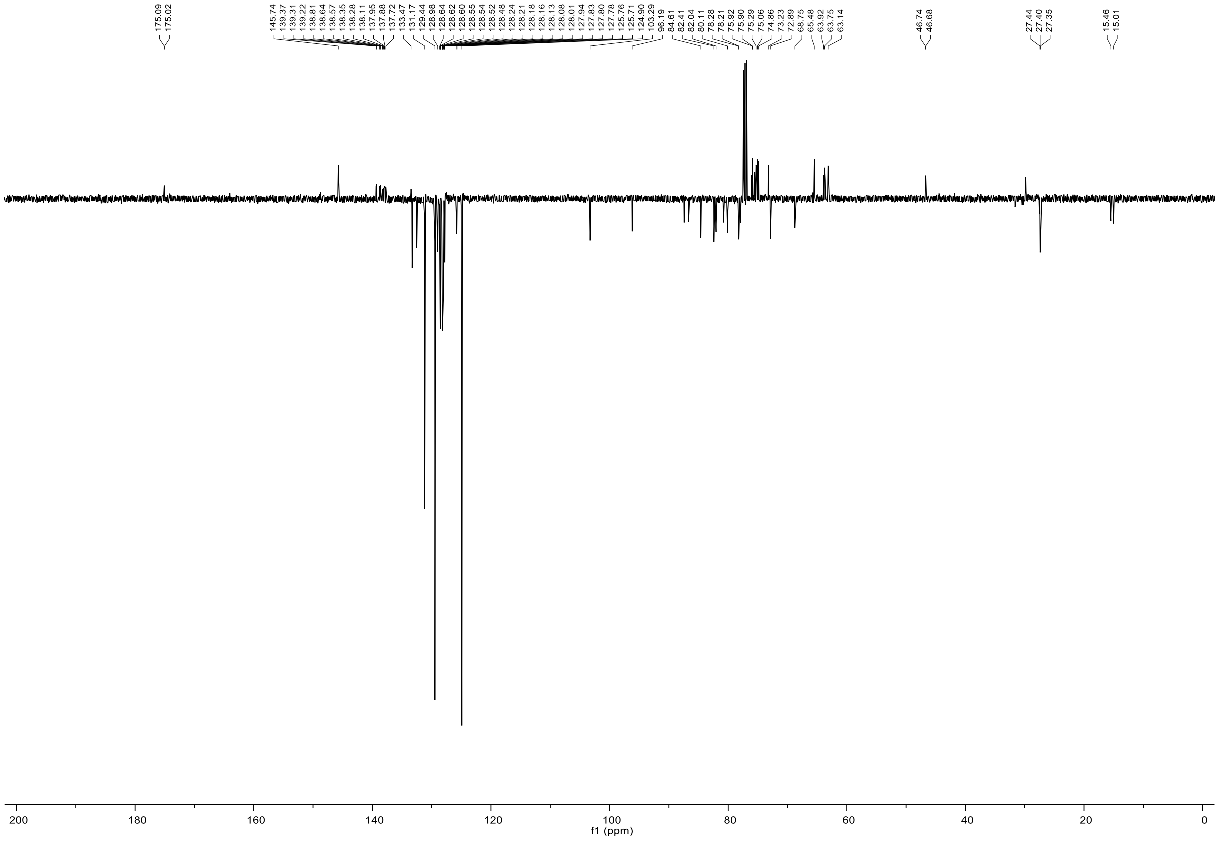

Supplementary Figure S197. $^{13}C\{^{1}H\}$ NMR, 101 MHz, $CDCl_3$ of compound **S34**

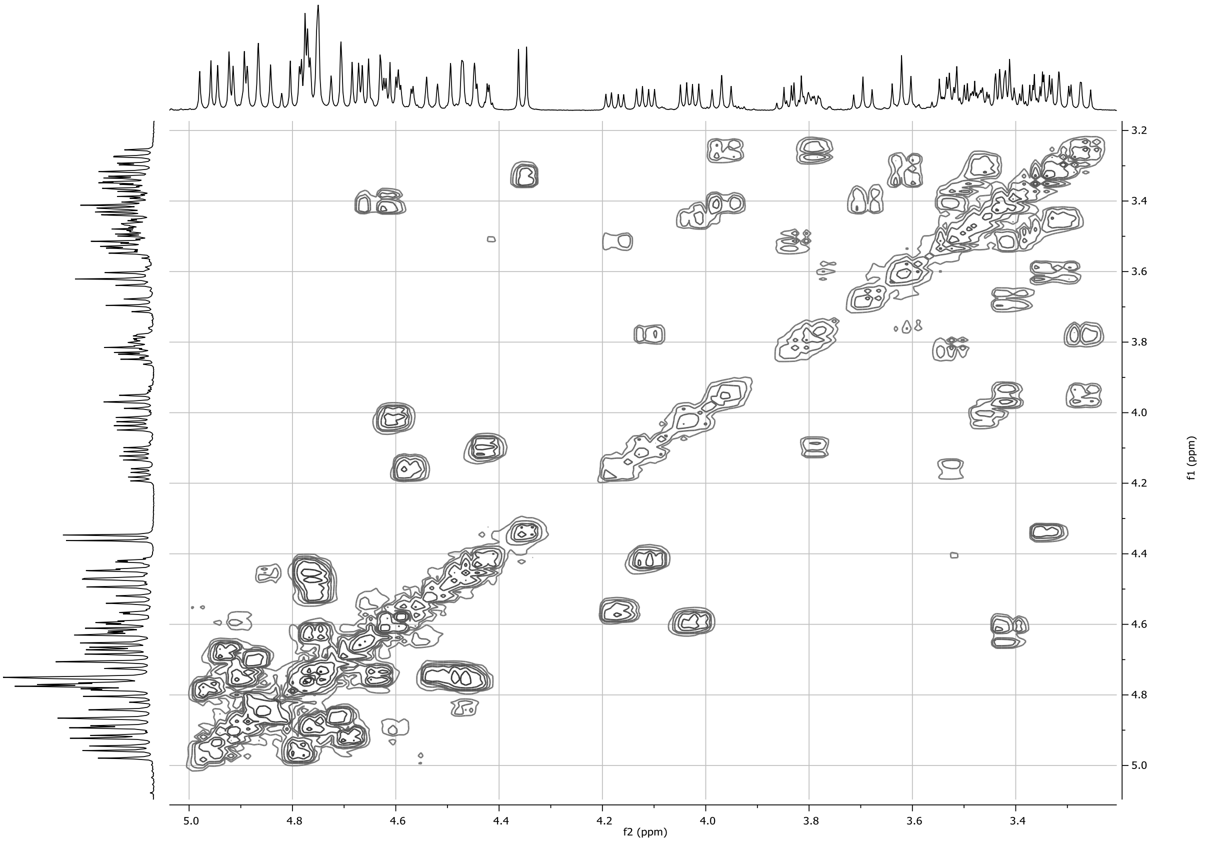

Supplementary Figure S198. HH-COSY NMR, $CDCl_3$ of compound **S34**

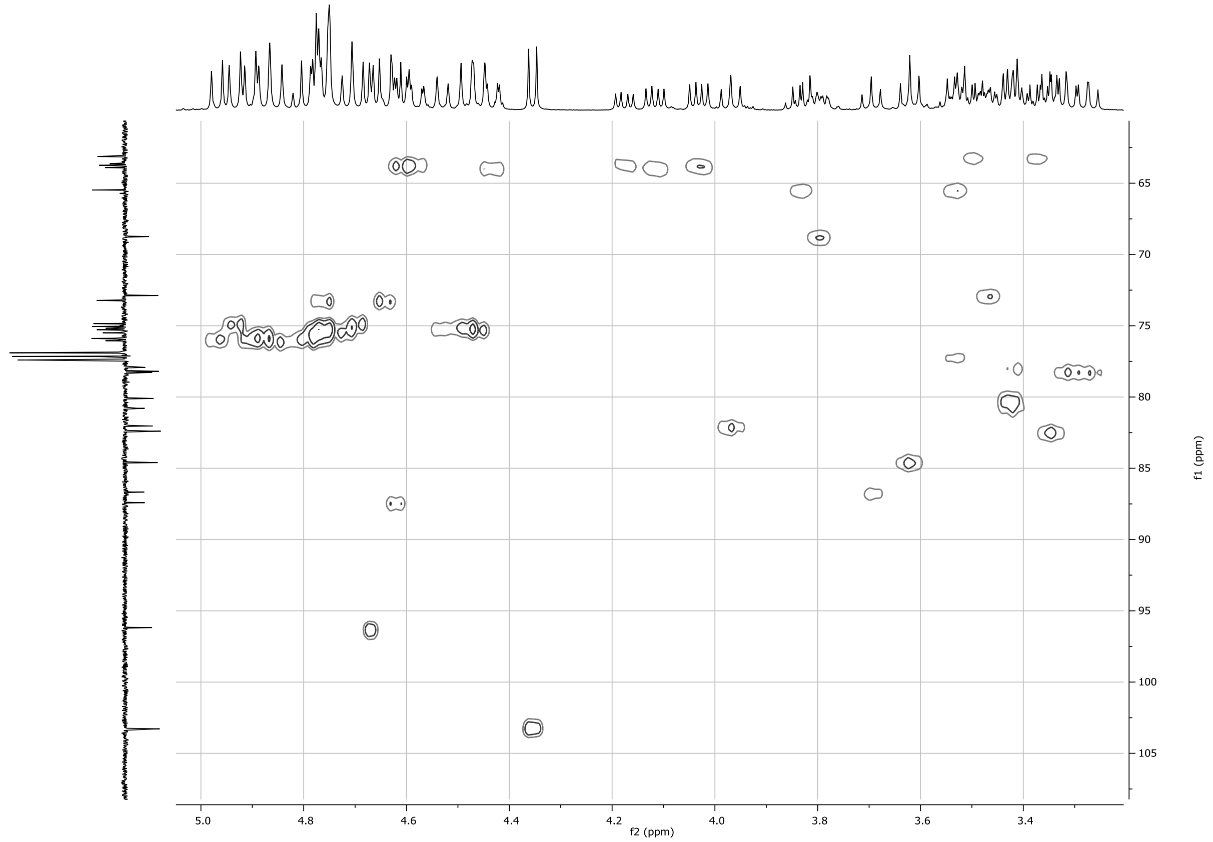

Supplementary Figure S199. HSQC{^{1}H} NMR, $CDCl_3$ of compound **S34**

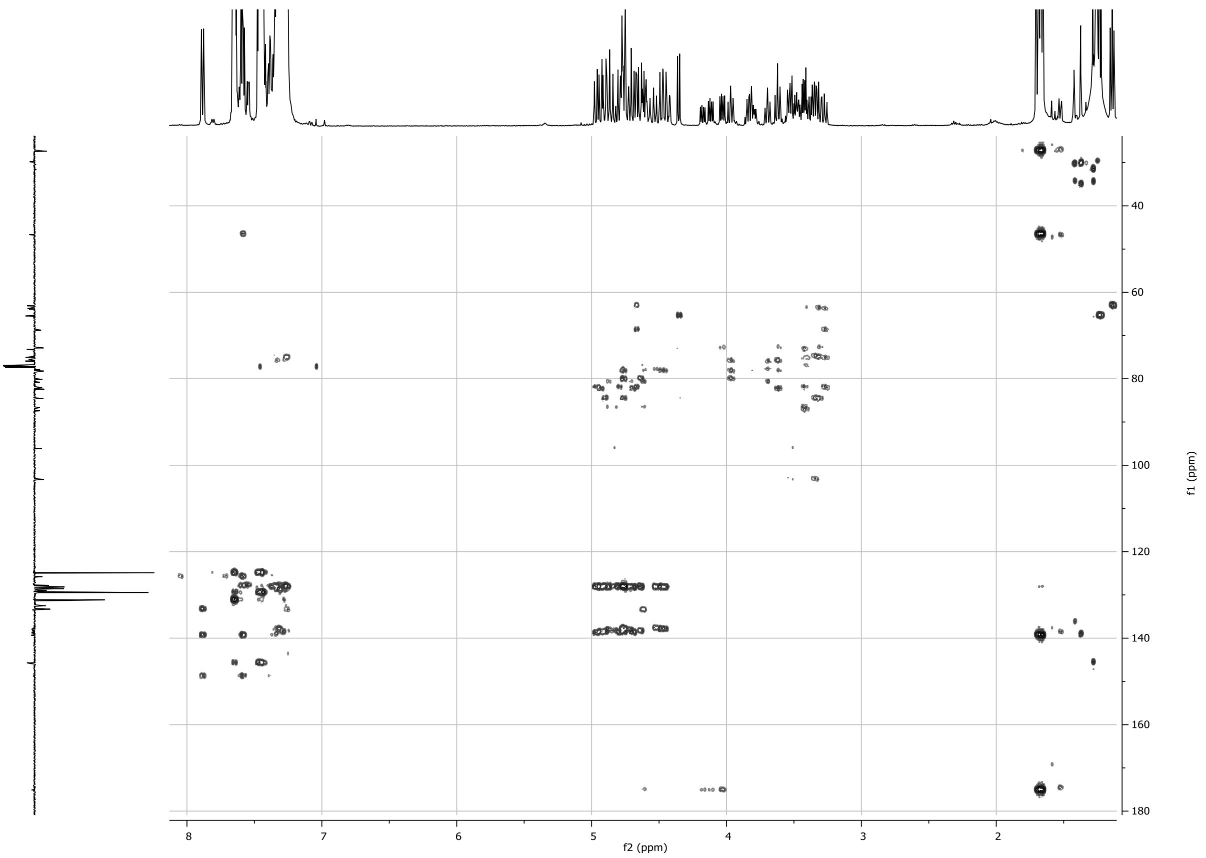

Supplementary Figure S200. HMBC{^{1}H} NMR, $CDCl_3$ of compound **S34**

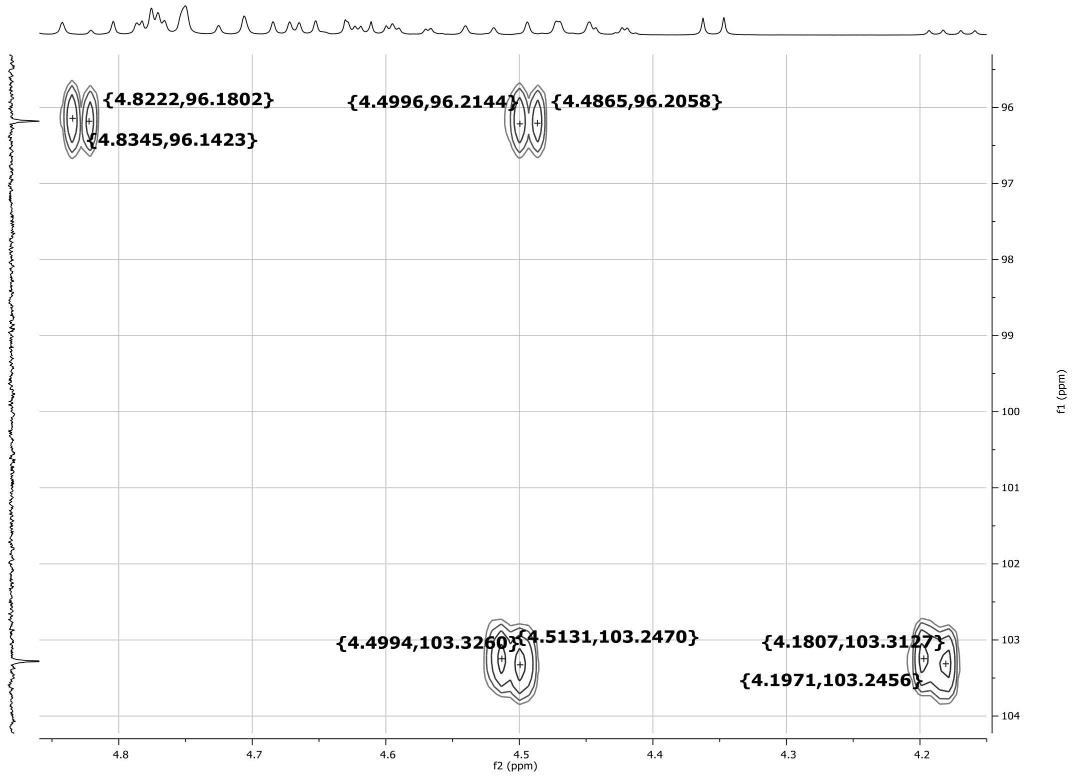

Supplementary Figure S201. HMBC-Gated NMR, $CDCl_3$ of compound **S34**

Supplementary References

1 van Outersterp, R.E., Houthuijs, K.J., Berden, G., Engelke, U.F., Kluijtmans, L.A.J., Wevers, R.A., Coene, K.L.M., Oomens, J., Martens, J., and Molecular Spectroscopy (HIMS, FNWI), Reference-standard free metabolite identification using infrared ion spectroscopy, *Int. J. Mass Spectrom.*, 2019, **443**, 77–85.

2 G. Landrum, P. Tosco, B. Kelley, Ric, sriniker, gedeck, R. Vianello, NadineSchneider, E. Kawashima, A. Dalke, D. N, B. Cole, D. Cosgrove, M. Swain, S. Turk, AlexanderSavelyev, G. Jones, A. Vaucher, M. Wójcikowski, D. Probst, V. F. Scalfani, guillaume godin, A. Pahl, F. Berenger, JLVarjo, K. Ujihara, strets123, JP, DoliathGavid and G. Sforna, *rdkit/rdkit: 2021_09_4 (Q3 2021) Release*, Zenodo, 2022.

3 P. Tosco, N. Stiefl and G. Landrum, Bringing the MMFF force field to the RDKit: implementation and validation, *J. Cheminformatics*, 2014, **6**, 37.

4 J.-P. Ebejer, G. M. Morris and C. M. Deane, Freely Available Conformer Generation Methods: How Good Are They?, *J. Chem. Inf. Model.*, 2012, **52**, 1146–1158.

5 J. J. P. Stewart, Optimization of parameters for semiempirical methods V: Modification of NDDO approximations and application to 70 elements, *J. Mol. Model.*, 2007, **13**, 1173–1213.

6 M. J. Frisch, G. W. Trucks, H. B. Schlegel, G. E. Scuseria, M. A. Robb, J. R. Cheeseman, G. Scalmani, V. Barone, G. A. Petersson, H. Nakatsuji, X. Li, M. Caricato, A. V. Marenich, J. Bloino, B. G. Janesko, R. Gomperts, B. Mennucci, H. P. Hratchian, J. V. Ortiz, A. F. Izmaylov, J. L. Sonnenberg, Williams, F. Ding, F. Lipparini, F. Egidi, J. Goings, B. Peng, A. Petrone, T. Henderson, D. Ranasinghe, V. G. Zakrzewski, J. Gao, N. Rega, G. Zheng, W. Liang, M. Hada, M. Ehara, K. Toyota, R. Fukuda, J. Hasegawa, M. Ishida, T. Nakajima, Y. Honda, O. Kitao, H. Nakai, T. Vreven, K. Throssell, J. A. Montgomery Jr., J. E. Peralta, F. Ogliaro, M. J. Bearpark, J. J. Heyd, E. N. Brothers, K. N. Kudin, V. N. Staroverov, T. A. Keith, R. Kobayashi, J. Normand, K. Raghavachari, A. P. Rendell, J. C. Burant, S. S. Iyengar, J. Tomasi, M. Cossi, J. M. Millam, M. Klene, C. Adamo, R. Cammi, J. W. Ochterski, R. L. Martin, K. Morokuma, O. Farkas, J. B. Foresman and D. J. Fox, *Gaussian 16 Rev. C.01*, Wallingford, CT, 2016.

7 A. D. Becke, Density-functional thermochemistry. III. The role of exact exchange, *J. Chem. Phys.*, 1993, **98**, 5648–5652.

8 null Lee, null Yang and null Parr, Development of the Colle-Salvetti correlation-energy formula into a functional of the electron density, *Phys. Rev. B Condens. Matter*, 1988, **37**, 785–789.

9 P. J. Stephens, F. J. Devlin, C. F. Chabalowski and M. J. Frisch, Ab Initio Calculation of Vibrational Absorption and Circular Dichroism Spectra Using Density Functional Force Fields, *J. Phys. Chem.*, 1994, **98**, 11623–11627.

10 M. J. Frisch, J. A. Pople and J. S. Binkley, Self-consistent molecular orbital methods 25. Supplementary functions for Gaussian basis sets, *J. Chem. Phys.*, 1984, **80**, 3265–3269.

11 C. Møller and M. Plesset, Note on an Approximation Treatment for Many-Electron Systems, , DOI:10.1103/PHYSREV.46.618.

12 S. Grimme, A. Hansen, J. G. Brandenburg and C. Bannwarth, Dispersion-Corrected Mean-Field Electronic Structure Methods, *Chem. Rev.*, 2016, **116**, 5105–5154.

13 H. Liu, S.-Y. Zhou, G.-E. Wen, X.-X. Liu, D.-Y. Liu, Q.-J. Zhang, R. R. Schmidt and J.-S. Sun, The 2,2-Dimethyl-2-(ortho-nitrophenyl)acetyl (DMNPA) Group: A Novel Protecting Group in Carbohydrate Chemistry, *Org. Lett.*, 2019, **21**, 8049–8052.

14 S. Tani, S. Sawadi, M. Kojima, S. Akai and K. Sato, A novel method for regioselective ring-opening reduction of 4,6-O-benzylidene hexopyranoside derivatives using CoCl2 and BH3·THF, *Tetrahedron Lett.*, 2007, **48**, 3103–3104.

15 J. Lv, T. Luo, D. Zou and H. Dong, Using DMF as Both a Catalyst and Cosolvent for the Regioselective Silylation of Polyols and Diols, *Eur. J. Org. Chem.*, 2019, **2019**, 6383–6395.
